# Composition and structural characteristics of lipids in yak, buffalo, and cow colostrum based on untargeted lipidomics

**DOI:** 10.1038/s41538-025-00406-x

**Published:** 2025-03-23

**Authors:** Yuzhuo Wang, Changhui Li, Jiaxiang Huang, Qingkun Zeng, Ling Li, Pan Yang, Pengjie Wang, Min Chu, Jie Luo, Fazheng Ren, Hao Zhang

**Affiliations:** 1 College of Food Science and Nutritional Engineering, China Agricultural University, Beijing, China; 2 Guangxi Buffalo Research Institute, Chinese Academy of Agricultural Sciences, Nanning, China; 3 Beijing Laboratory of Food Quality and Safety, Department of Nutrition and Health, China Agricultural University, Beijing, China; 4 Lanzhou Institute of Husbandry and Pharmaceutical Sciences, Chinese Academy of Agricultural Science, Lanzhou, China; 5 College of Food Science and Technology, Hunan Agricultural University, Changsha, China; 6 Food Laboratory of Zhongyuan, Luohe, China

**Keywords:** Biomarkers, Agriculture

## Abstract

Lipids play pivotal roles in supplying energy and promoting gut health. While yak and buffalo milk are known for their high nutritional values, the lipid compositions of yak colostrum (YC) and buffalo colostrum (BC) remain poorly explored. Here, untargeted lipidomics were applied to analyze YC, BC, and cow colostrum (CC). 546, 353, and 417 differential lipids were identified in the comparisons of YC-CC, BC-CC, and YC-BC, respectively. Compared to CC, YC exhibited a higher content of C18:2, while BC was marked by lower levels of saturated fatty acids. Additionally, specific lipid biomarkers were identified: triacylglycerol (TG) (16:0_10:0_22:6), TG (4:0_12:3_16:0), TG (4:0_8:0_18:2), TG (6:0_6:0_22:6), TG (6:0_8:0_22:6), and TG (6:0_8:0_8:0) were more for YC, while ceramide (Cer) (d19:1_24:1), diacylglycerol (DG) (36:2), hexosyl ceramide (Hex1Cer) (d37:1), TG (40:2e), TG (4:0_12:0_18:2), and zymosteryl (ZyE) (24:7) were biomarkers for BC. These findings provide a theoretical basis for optimizing the use of colostrum in various applications.

## Introduction

Colostrum, produced within the first 72 h postpartum, is a nutrient-rich milk that contains high concentrations of antimicrobial agents, immunomodulators, and growth factors. Its unique composition offers a range of biological functions, not only supplying essential nutrients for newborns but also enhancing immune defense and preventing infections^1^. Additionally, research involving both animal models and human studies have revealed its therapeutic potential in conditions such as gastrointestinal disorders^2^, skin inflammation^3^, diabetes^4^, hypercholesterolemia^5^, and nonalcoholic fatty liver disease^6^. Colostrum is nutritionally beneficial across all age groups with an excellent safety profile^7^, showing great promise in food innovation and development.

Lipids, accounting for 3–5% of milk’s total composition, serve as a major energy source and play a pivotal role in cellular membrane formation and signal transduction, supporting brain and visual development^8^. Milk lipids are secreted by mammary epithelial cells in the form of fat globules, which are enveloped by membranes composed of proteins, glycolipids, and phospholipids, ensuring stability and aiding digestion and absorption^9^. Based on their chemical structure, milk lipids can be classified into fatty acids (FA), glycerolipids (GL), glycerophospholipids (GP), sphingolipids (SP), sterol lipids (ST), prenolipids (PR), glycolipids (SL), polyketides (PK), and others. Research has shown that lipid composition changes throughout lactation. For example, Li et al. observed shifts in lipid metabolic pathways such as glycerophospholipid metabolism, sphingolipid metabolism, glycerolipid metabolism and arachidonic acid metabolism in bovine colostrum compared to mature milk^10^. Similarly, Xiong et al. found that yak (*Bos grunniens*) colostrum (YC) contains higher levels of essential amino acids (EAA), phosphatidylcholines (PC), phosphatidylglycerols (PG), glycerols, phosphatidylserines (PS), lyso-phosphatidylcholines (LPC), lyso-phosphatidylglycerols (LPG), and other functional lipids than yak mature milk^11^. As colostrum contains several health-promoting components distinct from those in mature milk, further investigation into its lipid composition is warranted.

The lipid composition of milk varies significantly across species^12^. Yaks, which inhabit high-altitude plateaus at elevations of 3000–5000 m, exhibit unique characteristics in their gene families, gastrointestinal microbiota, and metabolic adaptations to hypoxia and energy stress due to harsh environmental conditions^13^. As a result of the combined effects of external dietary factors and inherent physiological traits, yak milk has notably higher levels of protein, fat, lactose, and ash compared to milk from other animals^14^. The fat content in yak milk, ranging between 5.3% and 8.8%, is almost double that of Holstein milk^15^. Rich in didecapentaenoic acid, docosahexaenoic acid, arachidonic acid, and α-linolenic acid, yak milk demonstrates physiological functions such as antioxidant, anticancer, and anti-inflammatory properties^16^. Buffalo (*Bubalus bubalis*), predominantly found in tropical and subtropical regions, thrives in warm and humid environments. Buffalo milk accounts for approximately 13% of global milk production, making it the second largest contributor worldwide^17^. Buffalo milk is particularly rich in functional fatty acids, including arachidonic acid, eicosapentaenoic acid, and docosahexaenoic acid, which are known for their antioxidant activities^18^. Despite these insights into the nutritional benefits of yak and buffalo milk, the corresponding differences in colostrum composition remain unexplored. Therefore, it is crucial to thoroughly examine the lipid profiles of YC, buffalo colostrum (BC), and cow (*Bos taurus*) colostrum (CC).

The advancement of lipidomics, characterized by high throughput, precision, and accuracy, has become a powerful tool for elucidating the roles of lipids in biological processes. This technology is particularly valuable in the analysis of dairy products, aiding in composition analysis, quality control, and source identification^19^. For instance, Liu et al. employed UPLC-Q-Exactive Orbitrap mass spectrometry to examine goat milk from various lactation stages and geographical origins, identifying 38 and 19 potential lipid biomarkers, respectively^20^. Similarly, England et al. utilized MALDI-TOF mass spectrometry to distinguish bovine from non-dairy milk, offering a rapid, accurate, and cost-effective method for detecting milk adulteration^21^.

In this study, untargeted lipidomics was employed to analyze the lipid profiles of YC, BC, and CC, and to compare the lipid differences among YC-CC, BC-CC, and YC-BC. Moreover, lipid biomarkers unique to YC and BC were identified. This research provides a comprehensive understanding of the lipid composition in colostrum from different species, offering a theoretical basis for future colostrum applications.

## Results

### Validation of analytical methods

To systematically assess potential errors introduced by the sample extraction and analysis procedures, QC samples were prepared by pooling equal quantities of all collected samples. The PCA score plot indicated that the QC samples clustered tightly within a 95% confidence interval (See Supplementary Fig. 1), confirming the high reproducibility of the sample preparation process and the reliability and stability of the analytical method employed in this study.

### Lipid identification of YC, BC, and CC

**Fig. 1:**
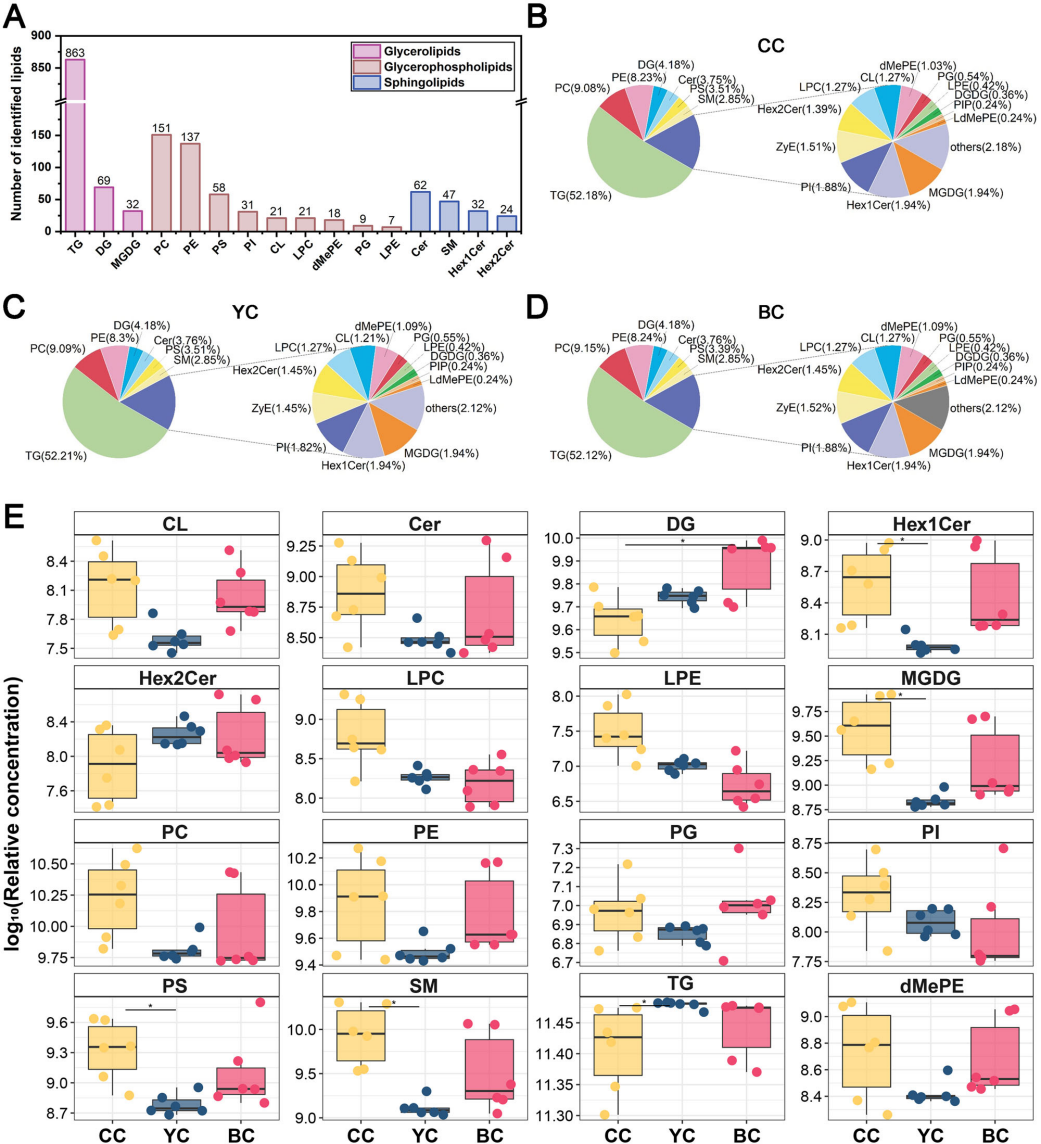
Identified lipid subclasses in yak colostrum (YC), buffalo colostrum (BC), and cow colostrum (CC). **A** Total number of lipids in all samples. **B** Percentages of lipid subclasses numbers in CC. **C** Percentages of lipid subclasses numbers in YC. **D** Percentages of lipid subclasses numbers in BC. **E** Comparative analysis of relative lipid subclass content in YC, BC, and CC.

A total of 1657 lipids were identified across the YC, BC, and CC groups (See Supplementary Table 1), which were categorized into 41 subclasses. The 16 most abundant lipid categories were further grouped into three principal classes: (1) GL, comprising 863 triacylglycerols (TG), 69 diacylglycerols (DG), and 32 monogalactosyl diacylglycerols (MGDG); (2) GP, including 151 PC, 137 phosphatidylethanolamines (PE), 58 PS, 31 phosphatidylinositols (PI), 21 cardiolipins (CL), 21 lysophosphatidylcholines (LPC), 18 dimethylphosphatidylethanolamines (dMePE), 9 PG, and 7 lysophosphatidylethanolamines (LPE); and (3) SP, comprising 62 ceramides (Cer), 47 sphingomyelins (SM), 32 hexosyl ceramides (Hex1Cer), and 24 dihexosyl ceramides (Hex2Cer) (Fig. 1A). The overall lipid composition was consistent across the three groups, with TG representing 52% of total lipids, followed by PC (9%), PE (8%), DG (4%), and Cer (3.7%) (Fig. 1B–D). The total amount of each subclass was calculated by summing the quantities of all detected lipids within that subclass. The results indicated a significant increase in TG levels in YC compared to CC (p < 0.05), while MGDG, PS, and SM levels were notably lower in YC than in CC (p < 0.05). Additionally, DG content in BC was significantly elevated compared to CC (p < 0.05). Although other lipid subclasses exhibited variations across the YC, BC, and CC groups, these differences were not statistically significant (Fig. 1E).

### Multivariate statistical analysis of lipids among YC, BC, and CC

**Fig. 2:**
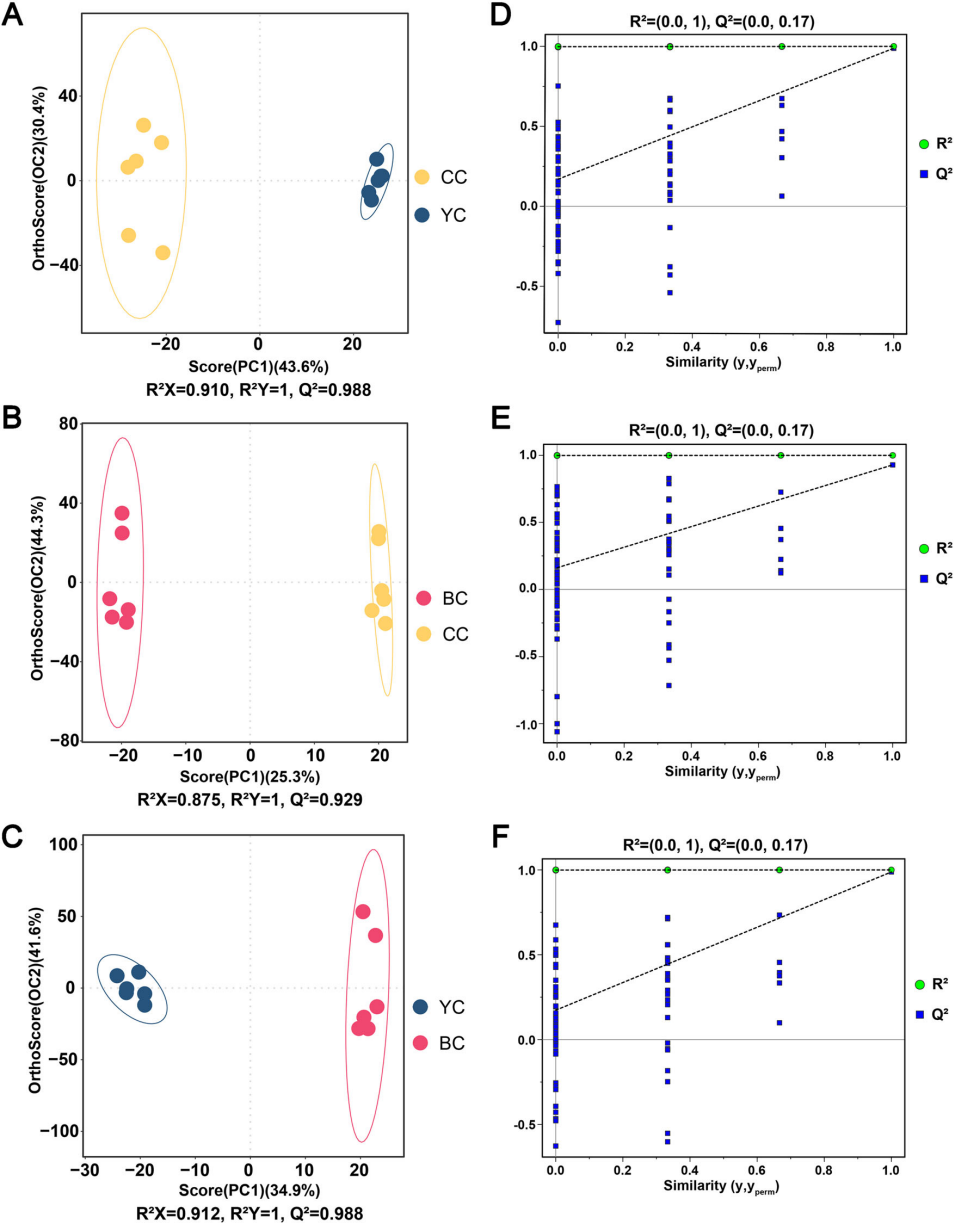
Multivariate statistical analysis of yak colostrum (YC), buffalo colostrum (BC), and cow colostrum (CC). Orthogonal partial least squares discriminant analysis (OPLS-DA) score plots for (**A**) YC versus CC, (**B**) BC versus CC, and (**C**) YC versus BC. OPLS-DA permutation test plots for (**D**) YC versus CC, (**E**) BC versus CC, and (**F**) YC versus BC.

To further investigate the lipid differences among YC, BC, and CC, pairwise multivariate statistical analyses and alignment tests were conducted. The analysis revealed clear clustering patterns and significant differences between the groups. The OPLS-DA analysis yielded the following model parameters: for YC vs. CC, R^2^X = 0.910, R^2^Y = 1, Q^2^ = 0.988 (Fig. 2A); for BC vs. CC, R^2^X = 0.875, R^2^Y = 1, and Q^2^ = 0.929 (Fig. 2B); and for YC vs. BC, R^2^X = 0.912, R^2^Y = 1, and Q^2^ = 0.988 (Fig. 2C). These results suggest a strong fit and predictive capacity of the data. To mitigate the risk of overfitting, permutation tests were performed, confirming that the models were robust and reliable (Fig. 2D–F). Consequently, the VIP values obtained from the OPLS-DA model can be utilized for further analysis of DLs.

### Identification of DLs between YC and CC

**Fig. 3:**
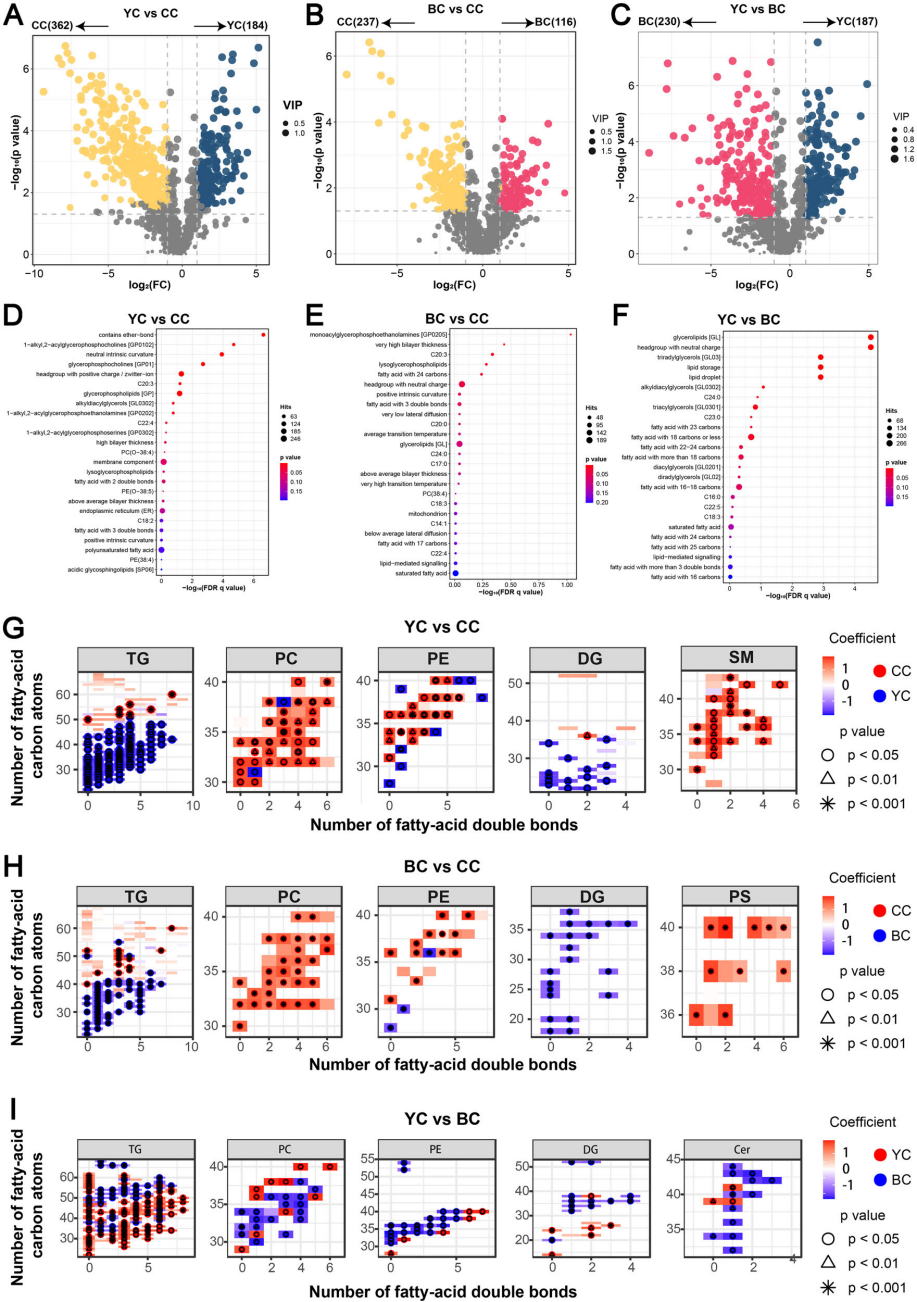
Lipid profile differences in pairwise comparisons. Volcano plots of differential lipids (DLs) for **A** yak colostrum (YC) versus cow colostrum (CC), **B** buffalo colostrum (BC) versus CC, and **C** YC versus BC. LION/web lipid ontology enrichment analysis for DLs between **D** YC and CC, **E** BC and CC, and **F** YC and BC. Heat maps of structural characteristics of DLs between **G** YC versus CC, **H** BC versus CC, and **I** YC versus BC.

To further elucidate the differences between YC and CC lipids, a comparative analysis of 546 DLs was performed (VIP > 1, *p* < 0.05, FC > 2 or FC < 0.5), and the results were visualized in a volcano plot (Fig. 3A). In comparison to CC, 184 lipids were significantly upregulated in YC, including 137 TG, 14 DG, 12 PE, 8 Hex2Cer, 4 Cer, and 3 PC. Conversely, 362 lipids were significantly downregulated, including 75 TG, 70 PC, 46 PE, 31 SM, 22 PS, 21 MGDG, 18 Hex1Cer, 16 Cer, 10 LPC, 6 CL, 6 dMePE, and 6 PI (See Supplementary Table 2). To deepen the understanding of DLs characteristics, the LION/web lipid ontology database was utilized (Fig. 3D). The results indicated that most DLs between YC and CC were concentrated in membrane components and the endoplasmic reticulum, which may be attributed to the selective lipid transport mechanisms of membrane components during milk synthesis in yaks and Holstein cows, as well as differences in lipid synthesis pathways within the endoplasmic reticulum^22^. YC notably enriched in polyunsaturated fatty acids (PUFAs), particularly C20:3, C22:4, and C18:2. Previous studies, such as that by Wang et al.^23^ reported a C18:2 content of 0.74% in Holstein milk, compared to 2.57% in yak milk, a trend consistent with our findings. Research has highlighted the health benefits of C18:2 in preventing and treating obesity, cancer, diabetes, and cardiovascular diseases^24^, positioning YC as a potential functional food supplement riched in C18:2. Further structural analysis of the identified DLs was conducted, focusing on saturation and carbon chain length in the five most significantly altered lipid subclasses. A heat map was used to interpret this information (Fig. 3G). Compared to CC, TG in YC exhibited a broader distribution of fatty acid chains with total carbon numbers ranging from 20 to 50 and unsaturation levels between 0 and 8, while TG with total carbon numbers between 50 and 60 were less prevalent. For DG in YC, carbon atoms were distributed between 20 and 35, with unsaturation levels between 0 and 3. In contrast, PC, PE, and SM with carbon atom numbers between 20 and 40 were notably reduced in YC. Although PC, PE, and SM—key components of the milk fat globule membrane (MFGM)—are found in higher concentrations in mature yak milk compared to cow milk^25^, our findings indicated lower levels of these lipids in YC relative to CC. This disparity is likely linked to the differences in milk secretion stages. As yak calves age and require more polar lipids, the concentrations of PC, PE, and SM in mature yak milk increase significantly compared to YC^26^. The unique FA composition of YC, particularly the higher levels of PUFAs, may be attributed to the natural grazing environment of yaks, which is characterized by high-altitude pastures rich in specific plant species with significant amounts of PUFAs^27^.

### Identification of DLs difference between BC and CC

A total of 353 DLs were identified between BC and CC (VIP > 1, *p* < 0.05, FC > 2 or FC < 0.5), which were visualized through a volcano plot (Fig. 3B). Compared to CC, 116 lipids were significantly upregulated in BC, including 74 TG, 20 DG, 9 Hex2Cer, 4 Cer, 3 Hex1Cer, and 3 PE. Meanwhile, 237 lipids were significantly downregulated, including of 86 TG, 52 PC, 24 PE, 18 PS, 14 LPC, 9 SM, 8 MGDG, 6 Hex1Cer, 5 PI, 4 LPE, 3 Cer, and 2 CL (See Supplementary Table 3). Enrichment analysis using the LION/web lipid ontology database revealed that the DLs were primarily localized in mitochondria, suggesting a connection to lipid metabolism (Fig. 3E). Differences in feeding environments and distinct enzymatic or metabolic pathways between buffaloes and cows contributed to the observed variations in the fatty acid composition of BC and CC. Notably, the saturated fatty acids (SFAs) content in BC, including C17:0, C20:0, and C24:0, was significantly lower than in CC. Regarding unsaturated fatty acids, BC exhibited higher levels of C14:1 but lower levels of C20:3 and C22:4 compared to CC. The elevated C14:1 content in BC may play a vital role in the growth and immune protection of newborns^28^, while the lower levels of C20:3 and C22:4 likely reflect a balanced fatty acid composition specific to buffalo milk. Additionally, this study analyzed the top five lipid subclasses with the most significant DLs. The results indicated that BC contained higher levels of TG with carbon atom numbers distributed between 20 and 40 and carbon-carbon double bonds between 0 and 7, while TG with carbon atom numbers between 40 and 60 were less prevalent. Similarly, BC showed a relatively higher abundance of DG with carbon atom numbers ranging from 20 to 40. However, the concentrations of polar lipids such as PC, PE, and PS were lower in BC compared to CC (Fig. 3H).

### Identification of DLs between YC and BC

A total of 417 DLs were identified between YC and BC (VIP > 1, p < 0.05, FC > 2 or FC < 0.5), as illustrated in Fig. 3C. Among these, 187 lipids showed higher expression in YC, including 132 TG, 11 PC, 11 PE, 8 DG, 6 Cer, 6 PS, 3 LPC, 2 Hex2Cer, 2 PI, and 2 zymosteryl (ZyE). Conversely, 230 lipids were more highly expressed in BC, including 60 TG, 34 PE, 27 PC, 16 PS, 15 Hex1Cer, 13 DG, 12 Cer, 12 MGDG, 12 SM, and 6 CL (See Supplementary Table 4). Enrichment analysis using the LION/web lipid ontology database revealed that the majority of DLs between YC and BC contained fatty acids with 6–18 or 22–25 carbons, as well as those with more than three double bonds (Fig. 3F). In particular, YC exhibited higher levels of C18:3 (α-linolenic acid) and C22:5 (docosapentaenoic acid) compared to BC. C18:3, a precursor of docosahexaenoic acid, has been associated with various health benefits, including antiplatelet, anti-thrombotic, anti-inflammatory, antioxidant effects and promoting brain and nervous system development^26^. C22:5 is known for its ability to lower blood cholesterol and triglyceride levels, promote the metabolism of SFAs, and help prevent cardiovascular diseases such as atherosclerosis, cerebral thrombosis, and hypertension^29^. Consequently, due to its higher content of lipids such as LPC (18:3), PC (16:0_18:3), PC (20:0_18:3), PE (16:0_18:3), PE (18:1_18:3), TG (10:0_18:1_18:3), TG (10:0_18:3_18:3), TG (18:0_18:3_24:0), TG (18:3_11:1_18:2), TG (8:0_18:3_22:5), TG (16:0_10:0_22:5), TG (16:0_8:0_22:5), TG (17:0_18:1_22:5), TG (18:0_6:0_22:5), TG (4:0_17:0_22:5), TG (6:0_14:0_22:5), TG (6:0_8:0_22:5), TG (8:0_18:1_22:5), and MGDG (18:1_22:5), YC is potentially more advantageous than BC in promoting cardiovascular health, reducing cancer risk, and supporting neurodevelopment. Furthermore, YC exhibited a higher concentration of TG with 20–50 carbon atoms and fewer DG with 30–40 carbon atoms and 1–4 carbon-carbon double bonds. In contrast, BC had a greater abundance of PC with 30–36 carbon atoms and 0–4 double bonds, more PE with 30–40 carbon atoms and 0–5 double bonds, and more Cer with 30–45 carbon atoms and 1–2 double bonds (Fig. 3I). Notably, the natural grazing environment of yaks, with access to fresh pasture grass rich in unsaturated fatty acids, contrasts with the alfalfa-based feed for buffalo, potentially contributing to the lipid differences observed^27^.

### Characteristic lipid biomarkers of YC

**Fig. 4:**
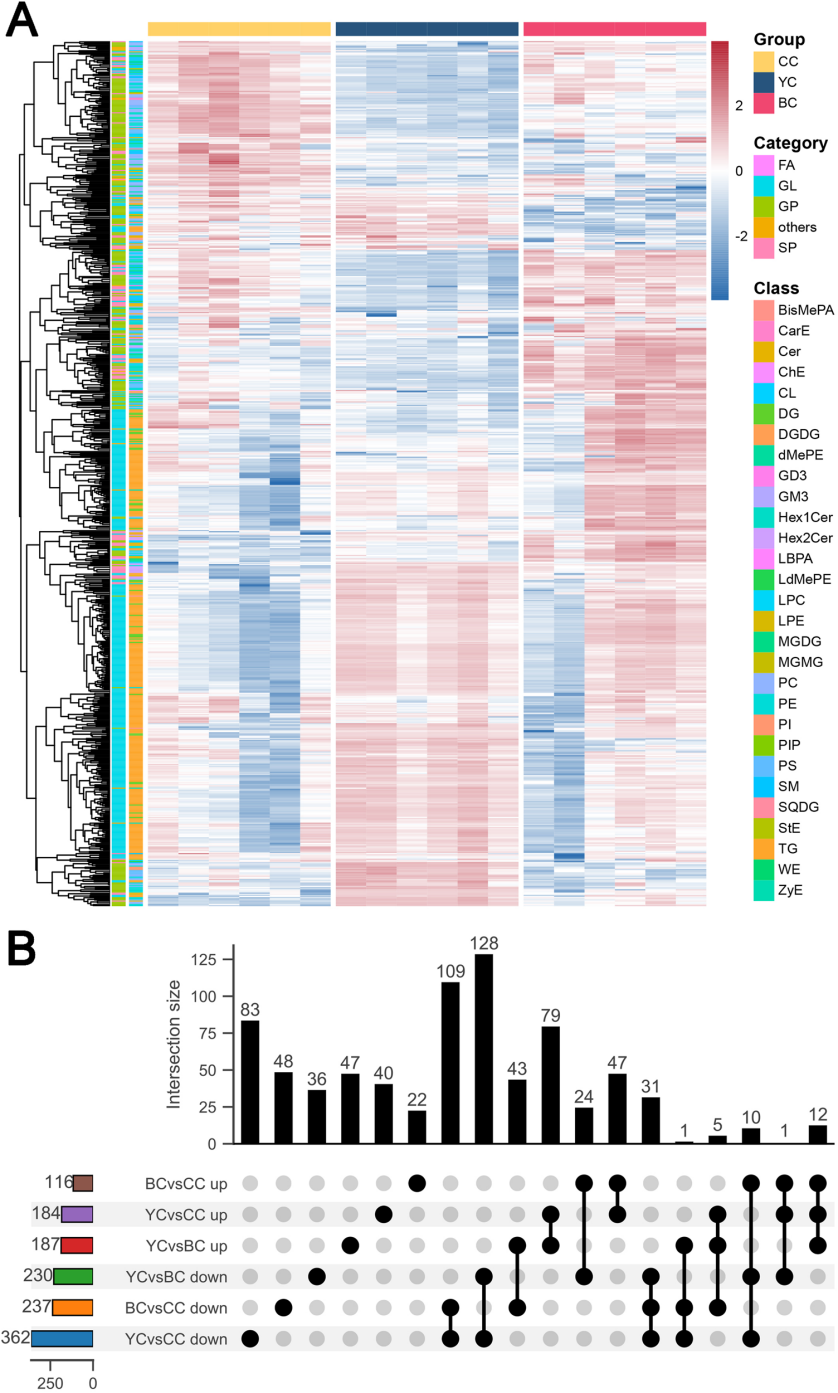
Clustering and distribution of all differential lipids (DLs) from pairwise comparisons of yak colostrum (YC), buffalo colostrum (BC), and cow colostrum (CC). **A** Heatmap showing all DLs across the three groups. **B** Upset plot displaying the unique and shared DLs in YC, BC, and CC.

**Fig. 5:**
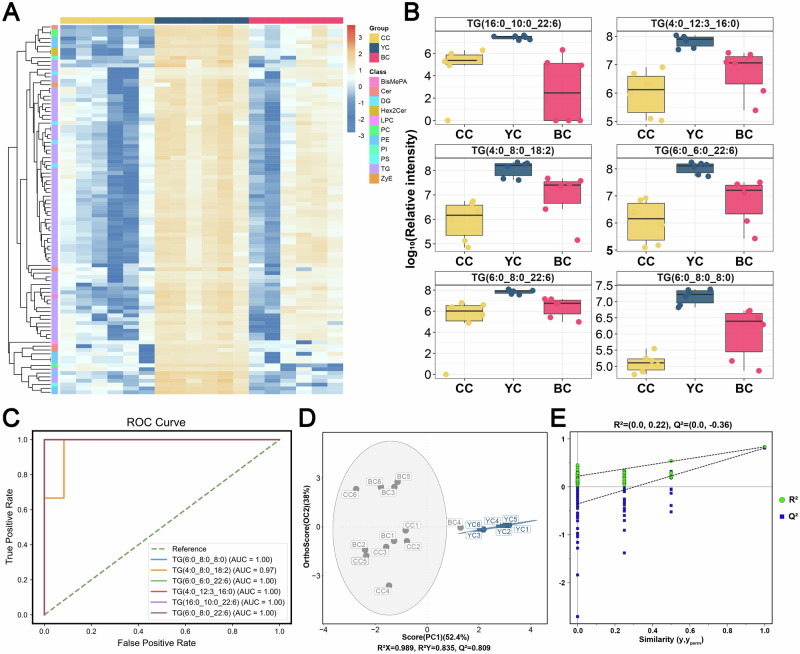
Lipid biomarkers of yak colostrum (YC). **A** Heatmap of lipids with significantly higher expression in YC compared to buffalo colostrum (BC) and cow colostrum (CC). **B** Comparison of the levels of six potential lipid biomarkers in YC, BC, and CC. **C** Receiver operating characteristic (ROC) curves for the six potential lipid biomarkers. **D** Orthogonal partial least squares discriminant analysis (OPLS-DA) score plot based on potential lipids across the three groups, with (**E**) corresponding OPLS-DA validation.

A total of 766 DLs were identified from the comparison of YC, BC, and CC, visualized as heat maps (Fig. 4A). The analysis revealed a significant increase in TG levels in the YC group. Classification showed 96 lipids specifically upregulated in YC and 169 downregulated compared to BC and CC (Fig. 4B). To identify potential biomarkers for YC, 96 significantly upregulated DLs were screened (Fig. 5A), including 69 TG, 9 PE, 4 DG, 4 Cer, 3 PC, 2 Hex2Cer, 1 LPC, 1 PI, 1 PS, 1 Bis(monoacylglycero)phosphate (BisMePA), and 1 ZyE. The DLs were ranked by FC to highlight the top six lipids: TG (16:0_10:0_22:6) [YC vs. CC, FC = 12.94; YC vs. BC, FC = 11.63], TG (4:0_12:3_16:0) [YC vs. CC, FC = 15.04; YC vs. BC, FC = 8.27], TG (4:0_8:0_18:2) [YC vs. CC, FC = 32.17; YC vs. BC, FC = 7.95], TG (6:0_6:0_22:6) [YC vs. CC, FC = 21.16; YC vs. BC, FC = 10.52], TG (6:0_8:0_22:6) [YC vs. CC, FC = 10.05; YC vs. BC, FC = 16.98], and TG (6:0_8:0_8:0) [YC vs. CC, FC = 35.48; YC vs. BC, FC = 8.37] (Fig. 5B). The AUC values for these lipid biomarkers in ROC curve analysis ranged from 0.97 to 1, indicating strong classification performance (Fig. 5C). The OPLS-DA model also showed clear separation of YC from other groups based on these lipid biomarkers (Fig. 5D), with permutation tests further confirming the model’s strong fit and predictive accuracy (Fig. 5E).

### Characteristic lipid biomarkers of BC

**Fig. 6:**
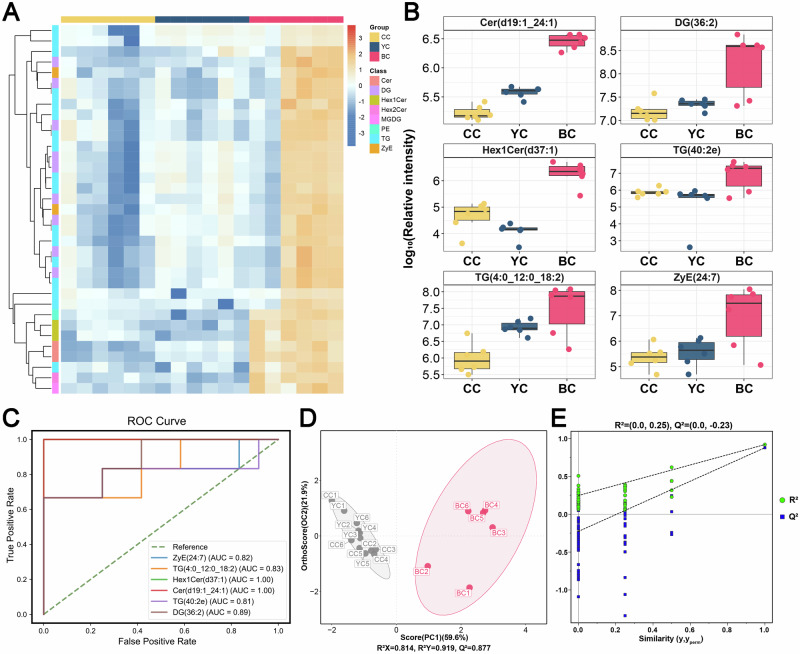
Lipid biomarkers of buffalo colostrum (BC). **A** Heatmap of lipids with significantly higher expression in BC compared to yak colostrum (YC) and cow colostrum (CC). **B** Comparison of the levels of six potential lipid biomarkers in BC, YC, and CC. **C** Receiver operating characteristic (ROC) curves for the six potential lipid biomarkers. **D** Orthogonal partial least squares discriminant analysis (OPLS-DA) score plot based on potential lipids across the three groups, with (**E**) corresponding OPLS-DA validation.

Compared to YC and CC, BC exhibited 35 lipids with significantly elevated expression and 49 with lower expression (Fig. 4B). To identify potential biomarkers for BC, 35 upregulated DLs were visualized (Fig. 6A), which included 17 TG, 9 DG, 2 Cer, 2 Hex1Cer, 2 ZyE, 1 Hex2Cer, 1 PE, and 1 MGDG. Using FC values, the top six lipids were identified: Cer (d19:1_24:1) [BC vs. CC, FC = 8.01; YC vs. BC, FC = 12.50], DG (36:2) [BC vs. CC, FC = 4.97; YC vs. BC, FC = 10.00], Hex1Cer (d37:1) [BC vs. CC, FC = 12.46; YC vs. BC, FC = 0.00], TG (40:2e) [BC vs. CC, FC = 5.22; YC vs. BC, FC = 25.00], TG (4:0_12:0_18:2) [BC vs. CC, FC = 14.12; YC vs. BC, FC = 0.20], and ZyE (24:7) [BC vs. CC, FC = 27.39; YC vs. BC, FC = 50.00] (Fig. 6B). Both Cer and Hex1Cer, which belong to the SP class, are involved in key physiological processes such as cell proliferation, differentiation, apoptosis, and inflammation. These lipids have been linked to positive effects on infant brain development and cognitive function^30^. The ROC curve analysis showed that the AUC values for Cer (d19:1_24:1), DG (36:2), Hex1Cer (d37:1), TG (40:2e), TG (4:0_12:0_18:2), and ZyE (24:7) were 1.00, 0.89, 1.00, 0.81, 0.83 and 0.82, respectively (Fig. 6C), indicating that these lipid biomarkers are highly representative. The OPLS-DA model demonstrated clear separation of the BC group from the other groups, confirming that the model was well-fitted and reliable, with no overfitting (Fig. 6D, E).

## Discussion

This study analyzed the differences in lipid composition and structural characteristics among YC, BC, and CC using untargeted lipidomics. A total of 1657 lipids from 41 subclasses were identified. Based on the criteria of VIP > 1, p < 0.05, and FC > 2 or FC < 0.5, 546, 353, and 417 DLs were identified in the comparisons between YC and CC, BC and CC, and YC and BC, respectively.

In terms of fatty acid composition, YC exhibited characteristics of multiple short-chain fatty acids (SCFAs) and PUFAs. SCFAs are typically digested more rapidly^31^. During gastrointestinal digestion, these hydrophilic molecules exhibit rapid phase separation forming micelles that enhance lipase accessibility to TG substrates. This kinetic advantage is attributable to the lower critical micelle temperature and stronger electrostatic interactions with bile salts of SCFAs, which collectively facilitate efficient emulsification and hydrolysis processes^32,33^. The elevated SCFAs may serve dual functions: providing rapid metabolic energy in cold climates while inhibiting cholesterol absorption through bile acid conjugation, collectively contributing to the species’ survival strategies under extreme environmental pressures. Additionally, the high PUFAs content, particularly C18:2 and C22:6, enhances YC’s potential to boost immunity, promote growth and development, and lower cholesterol levels^24^. This lipid profile likely reflects the adaptation of yaks to their high-altitude habitats, where the availability of forage and the metabolic requirements of the animals may play a significant role in shaping the lipid composition of their milk^34^. We speculate that YC can be developed into functional dairy products, including intestinal health drinks with prebiotic and immunomodulatory properties, infant nutrition formula to promote neurodevelopment, and high-altitude adapted foods for heat production and oxidative stress relief.

Compared with YC, the difference between BC and CC was smaller. While according to the phylogenetic trend, the buffalo (*Bubalus*) is more distantly related to the other two species (*Bos*), it is important to note that *Bos* and *Bubalis* are indeed very closely related within the broader evolutionary context. The differences observed in our study may reflect the greater influence of growing environment and dietary factors on colostrum lipid composition than phylogenetic trend. The content of SFAs in BC were significantly lower. Previous studies have demonstrated that long-chain SFAs (≥14 carbons) are particularly associated with elevated low-density lipoprotein cholesterol (LDL-C) and endothelial dysfunction, with a 1% increase in dietary SFAs corresponding to a 2–3% rise in coronary heart disease risk^35^. The metabolic advantage of BC may derive from its unique rumen microbial fermentation, which preferentially hydrolyzes long-chain fatty acids into medium-chain triglycerides (MCTs) and unsaturated derivatives through β-oxidation pathways^36^. This lipid remodeling mechanism not only lowers SFAs bioavailability but also generates conjugated linoleic acid (CLA) isomers with anti-atherogenic properties. Therefore, it is reasonable to infer that BC may be a more suitable dairy option for individuals at higher risk of cardiovascular disease. These findings emphasize the unique nutritional profiles of YC and BC.

Furthermore, following screening and validation, several lipid biomarkers were identified for each type of colostrum. For YC, TG (16:0_10:0_22:6), TG (4:0_12:3_16:0), TG (4:0_8:0_18:2), TG (6:0_6:0_22:6), TG (6:0_8:0_22:6), and TG (6:0_8:0_8:0) were identified as potential biomarkers. For BC, biomarkers included Cer (d19:1_24:1), DG (36:2), Hex1Cer (d37:1), TG (40:2e), TG (4:0_12:0_18:2), and ZyE (24:7)). Future research can be further explored from the aspects of standardizing feed formulations, precisely controlling sampling times, and increasing the sample size. In conclusion, the lipid composition of colostrum varies significantly across species. This study provides valuable theoretical insight into colostrum identification and supports the development of related functional foods.

## Methods

### Chemicals and reagents

Isopropyl alcohol, methanol, and acetonitrile were sourced from Fisher Scientific (Loughborough, UK), while ammonium formate was obtained from Sigma-Aldrich (Darmstadt, Germany). Chloroform was supplied by Sinopharm (Shanghai, China), and formic acid by TCI (Shanghai, China). Ultrapure water was generated using a Milli-Q system (Millipore, Bedford, USA).

### Collection of colostrum samples

We manually collected all samples from healthy animals confirmed to be disease-free through screening in this study. All samples were collected within the first 24–48 h postpartum. YC samples were obtained from yaks on individual farms in Gannan Tibetan Autonomous Prefecture, Gansu Province (n = 6; 5–6 years old; 1–3 days postpartum; weight: 210 ± 20 kg; grazing naturally on the same pasture: the grassland is the subalpine meadows, and the vegetation is dominated by *Cyperaceae* and *Gramineae*). BC samples were collected from Nili-Ravi buffaloes bred collaboratively by the Chinese Academy of Agricultural Sciences and the Buffalo Research Institute of Guangxi Zhuang Autonomous Region (n = 6; 5–6 years old; 2–4 parities; 1–3 days postpartum; weight: 520 ± 20 kg; consistent diet and environmental conditions: alfalfa as the main feed). CC samples were provided by Holstein cows from Daxing, Beijing (n = 6; 3–7 years old; 2–3 parities; 1–3 days postpartum; weight: 700 ± 50 kg; uniform diet and environmental conditions: alfalfa as the main feed). Each sample (50 mL) was manually collected into a sterile tube prior to feeding, promptly transferred under −20 °C cold chain conditions, and stored at −80 °C to ensure long-term stability and integrity for subsequent research.

### Lipid extraction of colostrum samples

Lipid extraction from colostrum was adapted from the method of Liu et al. with modifications^20^. First, 50 μL of the sample was mixed with 750 μL of extraction solution (chloroform: methanol = 2:1, v/v), vortexed, and placed in an ice bath for 40 min. Then, 190 μL of H_2_O was added, followed by vortexing and another 10-min incubation on ice. After centrifugation at 12,000 rpm for 5 min at room temperature, 300 μL of the organic layer was collected and combined with 500 μL of lipid extraction solution (chloroform: methanol = 2:1, v/v). The mixture was centrifuged again at 12,000 rpm for 5 min at room temperature. Subsequently, 400 μL of the organic layer was transferred to a centrifugal vacuum evaporator (Eppendorf China Ltd., Shanghai, China) and dried under vacuum. Finally, the dried extract was redissolved in 200 μL of isopropyl alcohol, filtered through a Costar SpinX 0.22 μm pore nylon filter (Corning, New York, USA), and prepared for analysis. For quality control (QC), filtrates from all samples were pooled in equal proportions for machine testing.

### Lipid analysis

Lipid isolation and identification were performed using ultra-high performance liquid chromatography-electrospray ionization tandem mass spectrometry (UHPLC-ESI-MS/MS)^37,38^. Chromatographic separation was carried out on a Vanquish UHPLC System (Thermo Fisher Scientific, Waltham, MA, USA) equipped with an ACQUITY UPLC BEH C18 column (2.1 × 100 mm, 1.7 µm; Waters, Milford, MA, USA) maintained at 50 °C. The mobile phase A consisted of acetonitrile/water (60:40, v/v) with 0.1% formic acid and 10 mM ammonium formate, while mobile phase B comprised isopropyl alcohol/acetonitrile (9:1, v/v) with 0.1% formic acid and 10 mM ammonium formate. A 2 μL sample injection volume was used at 8 °C, with a flow rate of 0.25 mL/min for gradient elution. The mobile phase gradient was as follows: 0–5 min, 70–57% A; 5–5.1 min, 57–50% A; 5.1–14 min, 50–30% A; 14–14.1 min, 30% A; 14.1–21 min, 30–1% A; 21–24 min, 1% A; 24–24.1 min, 1–70% A; 24.1–28 min, 70% A. Mass spectrometry analysis was conducted on a Q Exactive mass spectrometer (Thermo Fisher Scientific, Waltham, MA, USA) in both positive and negative ion modes, scanning m/z 150-2000 with a resolution of 35,000. Spray voltages were set at 3.50 kV for the positive mode and 2.50 kV for the negative mode, with sheath gas and auxiliary gas flows of 30 arb and 10 arb, respectively. The capillary temperature was maintained at 325 °C, and higher-energy collisional dissociation (HCD) was employed for MS/MS experiments, using a collision energy of 30 eV. Dynamic exclusion was applied to filter out redundant signals.

### Lipid data processing and statistical analyses

Raw data processing, including peak alignment, filtering, and lipid annotation, was performed using LipidSearch software version 4.2.28 (Thermo Fisher Scientific, Waltham, MA, USA). Sum peak normalization was employed to adjust the data, and compounds with a relative standard deviation (RSD) > 30% in QC samples were excluded. Unsupervised and supervised multivariate statistical analyses, such as principal component analysis (PCA) and orthogonal partial least squares discriminant analysis (OPLS-DA), were conducted using the Ropls package. Significant differences were identified using paired t-tests and one-way analysis of variance (ANOVA) with Tukey’s HSD. Lipids with variable importance for the projection (VIP) > 1, p < 0.05, and fold change (FC) > 2 or FC < 0.5 were classified as differential lipids (DLs). Receiver operating characteristic (ROC) curves were generated for the biomarkers, and areas under the curve (AUC) were calculated. Lipid structures were analyzed using lipidomeR software (V0.1.2), and functional enrichment of lipids was conducted using the LION lipid ontology database, encompassing classifications of lipid properties, functions, and subcellular components^39^.

## Supplementary information

Supplementary Information

## Data Availability

Data available under request to the corresponding author.
