# Supplementary material for: Composition and structural characteristics of lipids in yak, buffalo, and cow colostrum based on untargeted lipidomics
Source: NPJ Sci Food. 2025 Mar 23;9:37. doi: 10.1038/s41538-025-00406-x (PMC11931005; doi:10.1038/s41538-025-00406-x)
Supplement: Supplementary file 1 — Supplementary Information [file 41538_2025_406_MOESM1_ESM.pdf]

Supplementary information

Supplementary Figure 1. Principal component analysis (PCA) score plots of yak colostrum (YC), buffalo colostrum (BC), cow colostrum (CC), and quality control (QC).

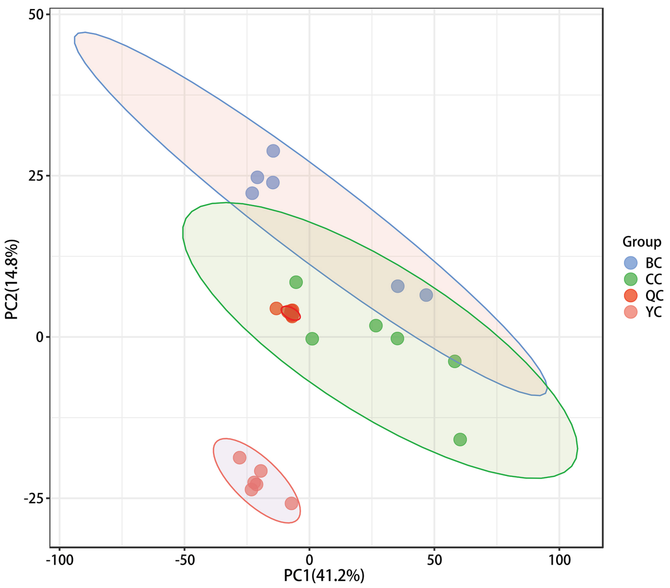

Supplementary Table 1. All lipids were identified in yak colostrum (YC), buffalo colostrum (BC), and cow colostrum (CC).

name	Class	MainIon	mz	rtmin	IonFormula
AcCa(22:0)	AcCa	M+H	484.4360355	8.63	C29 H58 O4 N1
BiotinylPE(31:1)	BiotinylPE	M+H	902.5687835	12.003	C46 H85 O10 N3 S1 P1
BisMePA(38:8e)	BisMePA	M+NH4	748.5275685	12.776	C43 H75 O7 N1 P1
BisMePA(4:0_22:0)	BisMePA	M+Na	615.3996295	11.936	C31 H61 O8 N0 P1 Na1
BisMePA(4:0_24:0)	BisMePA	M+Na	643.4309295	13.381	C33 H65 O8 N0 P1 Na1
CL(70:1)	CL	M+H	1436.057757	14.48457951	C79 H153 O17 P2
CL(70:2)	CL	M+H	1434.042107	12.60191525	C79 H151 O17 P2
CL(70:3)	CL	M+H	1432.026457	13.339458	C79 H149 O17 P2
CL(72:2)	CL	M-H	1460.058855	14.50924136	C81 H153 O17 P2
CL(72:4)	CL	M+H	1458.042107	13.3722847	C81 H151 O17 P2
CL(72:6)	CL	M+H	1454.010807	13.338	C81 H147 O17 P2
CL(74:1)	CL	M+H	1492.120357	15.80449915	C83 H161 O17 P2
CL(74:2)	CL	M+H	1490.104707	14.06750016	C83 H159 O17 P2
CL(74:3)	CL	M-H	1486.074505	14.53173067	C83 H155 O17 P2
CL(74:5)	CL	M+H	1484.057757	13.39798753	C83 H153 O17 P2
CL(76:4)	CL	M-H	1512.090155	15.79990866	C85 H157 O17 P2
CL(76:6)	CL	M-H	1508.058855	14.60103688	C85 H153 O17 P2
CL(76:8)	CL	M-H	1504.027555	13.39056092	C85 H149 O17 P2
CL(78:11)	CL	M+H	1528.026457	13.41	C87 H149 O17 P2
CL(78:9)	CL	M+H	1532.057757	14.54743777	C87 H153 O17 P2
CL(80:5)	CL	M+H	1568.151657	14.15668942	C89 H165 O17 P2
CL(80:7)	CL	M+H	1564.120357	13.01692097	C89 H161 O17 P2

CL(82:4)	CL	M+H	1598.198607	15.46364022	C91 H171 O17 P2
CL(82:6)	CL	M+H	1594.167307	14.32292853	C91 H167 O17 P2
CL(82:8)	CL	M+H	1590.136007	13.043	C91 H163 O17 P2
CL(83:10)	CL	M+H	1600.120357	14.707	C92 H161 O17 P2
CarE(18:0)	CarE	M+H	428.3734355	4.771009067	C25 H50 O4 N1
CarE(20:0)	CarE	M+H	456.4047355	6.852596299	C27 H54 O4 N1
Cer(d16:0_23:0)	Cer	M-H	608.5987175	17.70916947	C39 H78 O3 N1
Cer(d16:1_16:0)	Cer	M+HCOO	554.4789975	12.59048963	C33 H64 O5 N1
Cer(d16:1_22:0)	Cer	M+HCOO	638.5728975	16.81106118	C39 H76 O5 N1
Cer(d16:1_23:0)	Cer	M+HCOO	652.5885475	17.37211059	C40 H78 O5 N1
Cer(d16:1_24:1)	Cer	M+HCOO	664.5885475	16.6960172	C41 H78 O5 N1
Cer(d17:1_16:0)	Cer	M+HCOO	568.4946475	13.34470245	C34 H66 O5 N1
Cer(d18:0_16:0)	Cer	M+HCOO	584.5259475	14.58567941	C35 H70 O5 N1
Cer(d18:0_18:0)	Cer	M+HCOO	612.5572475	15.985	C37 H74 O5 N1
Cer(d18:1_16:0)	Cer	M+HCOO	582.5102975	14.1008785	C35 H68 O5 N1
Cer(d18:1_18:0)	Cer	M+HCOO	610.5415975	15.51701362	C37 H72 O5 N1
Cer(d18:1_18:1)	Cer	M+HCOO	608.5259475	14.188	C37 H70 O5 N1
Cer(d18:1_22:0)	Cer	M+HCOO	666.6041975	17.85812088	C41 H80 O5 N1
Cer(d18:1_23:0)	Cer	M-H	634.6143675	18.24835481	C41 H80 O3 N1
Cer(d18:1_24:0)	Cer	M-H	648.6300175	18.5688733	C42 H82 O3 N1
Cer(d18:1_24:1)	Cer	M+HCOO	692.6198475	17.78150544	C43 H82 O5 N1
Cer(d18:1_24:2)	Cer	M+HCOO	690.6041975	16.7902952	C43 H80 O5 N1
Cer(d18:2_23:0)	Cer	M+HCOO	678.6041975	17.28137856	C42 H80 O5 N1
Cer(d19:1_24:0)	Cer	M+HCOO	708.6511475	18.89714397	C44 H86 O5 N1
Cer(d19:1_24:1)	Cer	M+HCOO	706.6354975	18.03138471	C44 H84 O5 N1

Cer(d20:1_24:0)	Cer	M+HCOO	722.6667975	19.37542817	C45 H88 O5 N1
Cer(d23:0_19:0)	Cer	M+H	652.6602205	21.247	C42 H86 O3 N1
Cer(d24:0_17:0)	Cer	M+H	638.6445705	18.56859168	C41 H84 O3 N1
Cer(d24:0_18:0)	Cer	M+H	652.6602205	18.9502695	C42 H86 O3 N1
Cer(d32:0)	Cer	M+HCOO	556.4946475	13.0814563	C33 H66 O5 N1
Cer(d34:2)	Cer	M+HCOO	580.4946475	12.69858091	C35 H66 O5 N1
Cer(d38:0)	Cer	M+HCOO	640.5885475	17.20630964	C39 H78 O5 N1
Cer(d39:0)	Cer	M+HCOO	654.6041975	17.70916947	C40 H80 O5 N1
Cer(d39:1+O)	Cer	M+HCOO	668.5834625	16.908	C40 H78 O6 N1
Cer(d40:0)	Cer	M+HCOO	668.6198475	18.16911678	C41 H82 O5 N1
Cer(d40:1+O)	Cer	M+HCOO	682.5991125	17.462	C41 H80 O6 N1
Cer(d41:1+O)	Cer	M+HCOO	696.6147625	17.811	C42 H82 O6 N1
Cer(d42:1)	Cer	M+HCOO	694.6354975	18.84600685	C43 H84 O5 N1
Cer(d44:2+O)	Cer	M+Na	714.6370805	15.57325705	C44 H85 O4 N1 Na1
Cer(m19:0_22:0)	Cer	M+H	622.6496555	17.583	C41 H84 O2 N1
Cer(m19:0_23:0+O)	Cer	M+H	652.6602205	18.947	C42 H86 O3 N1
Cer(m20:0_17:0)	Cer	M+H	566.5870555	10.50183301	C37 H76 O2 N1
Cer(m20:0_21:0)	Cer	M+H	622.6496555	13.93090518	C41 H84 O2 N1
Cer(m30:1)	Cer	M+H	466.4618555	10.028	C30 H60 O2 N1
Cer(m36:0)	Cer	M+H	552.5714055	7.982424699	C36 H74 O2 N1
Cer(m37:0)	Cer	M+H	566.5870555	9.448741678	C37 H76 O2 N1
Cer(m39:0)	Cer	M+H	594.6183555	12.551	C39 H80 O2 N1
Cer(m40:0)	Cer	M+H	608.6340055	9.689	C40 H82 O2 N1
Cer(m41:0)	Cer	M+H	622.6496555	10.6740091	C41 H84 O2 N1
Cer(m43:0)	Cer	M+H	650.6809555	15.24913088	C43 H88 O2 N1

Cer(m45:0)	Cer	M+H	678.7122555	12.32438803	C45 H92 O2 N1
Cer(t16:0_22:0)	Cer	M+CH3COO	670.5991125	16.84114229	C40 H80 O6 N1
Cer(t17:0_22:0)	Cer	M+CH3COO	684.6147625	17.3042246	C41 H82 O6 N1
Cer(t17:0_23:0)	Cer	M+HCOO	684.6147625	17.30992473	C41 H82 O6 N1
Cer(t17:1_23:0)	Cer	M+HCOO	682.5991125	17.43772623	C41 H80 O6 N1
Cer(t18:0_23:0)	Cer	M+HCOO	698.6304125	17.82705688	C42 H84 O6 N1
Cer(t18:0_24:0)	Cer	M+HCOO	712.6460625	18.23786197	C43 H86 O6 N1
Cer(t18:0_24:0+O)	Cer	M+HCOO	728.6409775	17.94325608	C43 H86 O7 N1
Cer(t18:0_25:2)	Cer	M+CH3COO	736.6460625	15.38	C45 H86 O6 N1
Cer(t18:1_23:0)	Cer	M+HCOO	696.6147625	17.89075646	C42 H82 O6 N1
Cer(t18:1_24:0)	Cer	M+HCOO	710.6304125	18.26164416	C43 H84 O6 N1
Cer(t18:1_24:0+O)	Cer	M+HCOO	726.6253275	16.80904411	C43 H84 O7 N1
Cer(t18:1_26:0+O)	Cer	M+HCOO	754.6566275	17.83435406	C45 H88 O7 N1
Cer(t39:1)	Cer	M+HCOO	668.5834625	16.908	C40 H78 O6 N1
Cer(t41:1)	Cer	M-H	650.6092825	17.89075646	C41 H80 O4 N1
Cer(t43:1+O)	Cer	M+HCOO	740.6409775	17.369	C44 H86 O7 N1
Cer(t43:2)	Cer	M+CH3COO	736.6460625	15.76353827	C45 H86 O6 N1
Cer(t44:2)	Cer	M+HCOO	736.6460625	15.76298475	C45 H86 O6 N1
ChE()	ChE	M+NH4	404.3886905	12.024	C27 H50 O1 N1
ChE(20:4)	ChE	M+H	673.5918065	21.281	C47 H77 O2
CmE(28:6)	CmE	M+NH4	812.7279055	18.327	C56 H94 O2 N1
CmE(30:6)	CmE	M+H	823.7326565	18.88451123	C58 H95 O2
DG(14:0e)	DG	M+Na	325.2349315	12.765	C17 H34 O4 Na1
DG(14:1)	DG	M+Na	337.1985465	3.256949916	C17 H30 O5 Na1
DG(16:0e)	DG	M+Na	353.2662315	4.387197202	C19 H38 O4 Na1

DG(16:1e)	DG	M+Na	351.2505815	4.382764542	C19 H36 O4 Na1
DG(18:0e)	DG	M+Na	381.2975315	15.55594237	C21 H42 O4 Na1
DG(18:1_18:1)	DG	M+Na	643.5271965	16.40330816	C39 H72 O5 Na1
DG(18:1e)	DG	M+Na	379.2818815	15.99346226	C21 H40 O4 Na1
DG(18:2_18:2)	DG	M+NH4	634.5405005	14.331	C39 H72 O5 N1
DG(20:0e)	DG	M+Na	409.3288315	17.86672131	C23 H46 O4 Na1
DG(20:1e)	DG	M+Na	407.3131815	17.04164304	C23 H44 O4 Na1
DG(20:2)	DG	M+H	397.2948515	13.102	C23 H41 O5
DG(22:0e)	DG	M+Na	437.3601315	18.54187851	C25 H50 O4 Na1
DG(22:1e)	DG	M+Na	435.3444815	16.83410457	C25 H48 O4 Na1
DG(22:2e)	DG	M+H	411.3468865	18.55548416	C25 H47 O4
DG(23:0e)	DG	M+Na	451.3757815	18.85131049	C26 H52 O4 Na1
DG(24:0)	DG	M+Na	479.3706965	10.55743436	C27 H52 O5 Na1
DG(24:0e)	DG	M+Na	465.3914315	12.83324504	C27 H54 O4 Na1
DG(24:1e)	DG	M+Na	463.3757815	11.601	C27 H52 O4 Na1
DG(24:3e)	DG	M+H	437.3625365	11.50429588	C27 H49 O4
DG(25:0e)	DG	M+Na	479.4070815	13.48833545	C28 H56 O4 Na1
DG(25:2e)	DG	M+H	453.3938365	18.80273283	C28 H53 O4
DG(26:0e)	DG	M+Na	493.4227315	14.20664856	C29 H58 O4 Na1
DG(26:1e)	DG	M+Na	491.4070815	19.22538979	C29 H56 O4 Na1
DG(26:3)	DG	M+H	479.3731015	10.652	C29 H51 O5
DG(26:3e)	DG	M+Na	487.3757815	5.449220033	C29 H52 O4 Na1
DG(27:2e)	DG	M+H	481.4251365	19.438	C30 H57 O4
DG(27:3e)	DG	M+H	479.4094865	13.502	C30 H55 O4
DG(28:0e)	DG	M+Na	521.4540315	15.562	C31 H62 O4 Na1

DG(28:1e)	DG	M+Na	519.4383815	19.77228368	C31 H60 O4 Na1
DG(28:3e)	DG	M+H	493.4251365	14.21062839	C31 H57 O4
DG(28:4e)	DG	M+H	491.4094865	19.22566473	C31 H55 O4
DG(30:0)	DG	M+NH4	558.5092005	15.02697641	C33 H68 O5 N1
DG(30:1e)	DG	M+Na	547.4696815	15.73764374	C33 H64 O4 Na1
DG(30:2e)	DG	M+Na	545.4540315	19.8294247	C33 H62 O4 Na1
DG(30:3e)	DG	M+H	521.4564365	20.20516688	C33 H61 O4
DG(30:4e)	DG	M+H	519.4407865	19.77801166	C33 H59 O4
DG(31:0)	DG	M+Na	577.4802465	15.69439737	C34 H66 O5 Na1
DG(32:0)	DG	M+NH4	586.5405005	16.36834604	C35 H72 O5 N1
DG(32:0e)	DG	M+Na	577.5166315	17.87	C35 H70 O4 Na1
DG(32:1)	DG	M+NH4	584.5248505	15.05152446	C35 H70 O5 N1
DG(32:2)	DG	M+NH4	582.5092005	13.907	C35 H68 O5 N1
DG(32:2e)	DG	M+Na	573.4853315	20.345	C35 H66 O4 Na1
DG(32:3)	DG	M+NH4	580.4935505	5.257608174	C35 H66 O5 N1
DG(32:3e)	DG	M+H	549.4877365	25.748	C35 H65 O4
DG(34:0)	DG	M+NH4	614.5718005	17.51773501	C37 H76 O5 N1
DG(34:0e)	DG	M+Na	605.5479315	19.25442007	C37 H74 O4 Na1
DG(34:1)	DG	M+NH4	612.5561505	16.38206017	C37 H74 O5 N1
DG(34:1e)	DG	M+Na	603.5322815	14.51865128	C37 H72 O4 Na1
DG(34:2)	DG	M+NH4	610.5405005	15.277	C37 H72 O5 N1
DG(34:2e)	DG	M+Na	601.5166315	17.011	C37 H70 O4 Na1
DG(34:3e)	DG	M+Na	599.5009815	20.323	C37 H68 O4 Na1
DG(34:4e)	DG	M+H	575.5033865	12.044	C37 H67 O4
DG(35:2)	DG	M+NH4	624.5561505	15.778	C38 H74 O5 N1

DG(35:3e)	DG	M+H	591.5346865	18.906	C38 H71 O4
DG(36:0)	DG	M+NH4	642.6031005	18.407	C39 H80 O5 N1
DG(36:1)	DG	M+NH4	640.5874505	17.52400012	C39 H78 O5 N1
DG(36:2)	DG	M+NH4	638.5718005	16.40330816	C39 H76 O5 N1
DG(36:3)	DG	M+NH4	636.5561505	15.33215755	C39 H74 O5 N1
DG(36:4e)	DG	M+H	603.5346865	17.974	C39 H71 O4
DG(37:3e)	DG	M+H	619.5659865	21.58406186	C40 H75 O4
DG(38:1)	DG	M+NH4	668.6187505	18.83511775	C41 H82 O5 N1
DG(38:2e)	DG	M+H	635.5972865	19.953	C41 H79 O4
DG(38:4)	DG	M+NH4	662.5718005	16.292	C41 H76 O5 N1
DG(38:7e)	DG	M+H	625.5190365	20.527	C41 H69 O4
DG(40:3e)	DG	M+H	661.6129365	22.025	C43 H81 O4
DG(40:6)	DG	M+Na	691.5271965	18.32568084	C43 H72 O5 Na1
DG(52:1)	DG	M+NH4	864.8378505	21.89305025	C55 H110 O5 N1
DG(52:2)	DG	M+NH4	862.8222005	21.544	C55 H108 O5 N1
DG(8:0_8:0)	DG	M+Na	367.2454965	2.398	C19 H36 O5 Na1
DGDG(16:0_18:3)	DGDG	M+Na	937.5858965	11.98590032	C49 H86 O15 Na1
DGDG(18:3_18:3)	DGDG	M+H	937.5883015	11.997	C51 H85 O15
DGDG(30:5)	DGDG	M-H	853.4954985	15.16328412	C45 H73 O15
DGDG(32:5)	DGDG	M-H	881.5267985	16.43456442	C47 H77 O15
DGDG(32:6)	DGDG	M-H	879.5111485	15.35983845	C47 H75 O15
DGDG(33:0)	DGDG	M-H	905.6206985	18.695	C48 H89 O15
DLCL(29:5)	DLCL	M-H	823.3804245	13.25	C38 H65 O15 P2
GD3(d42:1)	GD3	M-H	1554.926506	16.145	C76 H136 O29 N3
GM3(d40:1)	GM3	M-H	1235.799787	15.26729949	C63 H115 O21 N2

GM3(d41:1)	GM3	M-H	1249.815437	15.856	C64 H117 O21 N2
GM3(d42:1)	GM3	M-H	1263.831087	16.43725379	C65 H119 O21 N2
Hex1Cer(d14:0_22:4)	Hex1Cer	M+HCOO	766.5474725	13.87280408	C43 H76 O10 N1
Hex1Cer(d15:0_18:1)	Hex1Cer	M+HCOO	730.5474725	12.31466684	C40 H76 O10 N1
Hex1Cer(d16:0_22:4)	Hex1Cer	M+HCOO	794.5787725	15.37434695	C45 H80 O10 N1
Hex1Cer(d16:0_22:5)	Hex1Cer	M+HCOO	792.5631225	14.47	C45 H78 O10 N1
Hex1Cer(d16:1_20:4)	Hex1Cer	M+HCOO	764.5318225	13.09002015	C43 H74 O10 N1
Hex1Cer(d16:1_22:5)	Hex1Cer	M+HCOO	790.5474725	13.11519791	C45 H76 O10 N1
Hex1Cer(d18:1_16:0)	Hex1Cer	M+HCOO	744.5631225	13.07571464	C41 H78 O10 N1
Hex1Cer(d23:1_18:3)	Hex1Cer	M+H	792.6347955	16.505	C47 H86 O8 N1
Hex1Cer(d32:0)	Hex1Cer	M+H	674.5565455	12.065	C38 H76 O8 N1
Hex1Cer(d32:1)	Hex1Cer	M+HCOO	716.5318225	11.52975808	C39 H74 O10 N1
Hex1Cer(d34:1)	Hex1Cer	M+HCOO	744.5631225	14.12148371	C41 H78 O10 N1
Hex1Cer(d35:1)	Hex1Cer	M+HCOO	758.5787725	13.525	C42 H80 O10 N1
Hex1Cer(d36:1)	Hex1Cer	M+HCOO	772.5944225	14.54315502	C43 H82 O10 N1
Hex1Cer(d36:2)	Hex1Cer	M+HCOO	770.5787725	13.34680441	C43 H80 O10 N1
Hex1Cer(d36:3)	Hex1Cer	M+HCOO	768.5631225	15.02346191	C43 H78 O10 N1
Hex1Cer(d37:1)	Hex1Cer	M+HCOO	786.6100725	15.03137604	C44 H84 O10 N1
Hex1Cer(d38:1)	Hex1Cer	M+HCOO	800.6257225	15.88307247	C45 H86 O10 N1
Hex1Cer(d39:1)	Hex1Cer	M+HCOO	814.6413725	16.51036504	C46 H88 O10 N1
Hex1Cer(d40:2)	Hex1Cer	M+HCOO	826.6413725	15.77771568	C47 H88 O10 N1
Hex1Cer(d42:0+O)	Hex1Cer	M+H	830.7079605	17.56	C48 H96 O9 N1
Hex1Cer(m20:1_18:2)	Hex1Cer	M+H	736.6085805	13.60225783	C44 H82 O7 N1
Hex1Cer(m32:0+O)	Hex1Cer	M+H	674.5565455	12.06879018	C38 H76 O8 N1
Hex1Cer(m39:0+O)	Hex1Cer	M+H	772.6660955	16.906	C45 H90 O8 N1

Hex1Cer(m41:0+O)	Hex1Cer	M+H	800.6973955	17.92877949	C47 H94 O8 N1
Hex1Cer(t17:0_20:5)	Hex1Cer	M-H	748.5369075	14.109	C43 H74 O9 N1
Hex1Cer(t18:0_20:4)	Hex1Cer	M+HCOO	810.5736875	13.3008572	C45 H80 O11 N1
Hex1Cer(t20:0_20:4)	Hex1Cer	M+HCOO	838.6049875	14.708	C47 H84 O11 N1
Hex1Cer(t32:0)	Hex1Cer	M+HCOO	734.5423875	13.08223786	C39 H76 O11 N1
Hex1Cer(t34:0)	Hex1Cer	M+HCOO	762.5736875	14.502	C41 H80 O11 N1
Hex1Cer(t36:0)	Hex1Cer	M+HCOO	790.6049875	14.91420732	C43 H84 O11 N1
Hex1Cer(t36:1)	Hex1Cer	M+CH3COO	802.6049875	15.189	C44 H84 O11 N1
Hex1Cer(t38:1)	Hex1Cer	M+HCOO	816.6206375	14.9783659	C45 H86 O11 N1
Hex2Cer(d34:0)	Hex2Cer	M+H	864.6406705	13.203	C46 H90 O13 N1
Hex2Cer(d37:1)	Hex2Cer	M+HCOO	948.6628975	14.56397056	C50 H94 O15 N1
Hex2Cer(d38:0)	Hex2Cer	M+H	920.7032705	15.928	C50 H98 O13 N1
Hex2Cer(d39:0)	Hex2Cer	M+H	934.7189205	16.5378844	C51 H100 O13 N1
Hex2Cer(d40:0)	Hex2Cer	M+H	948.7345705	17.11422111	C52 H102 O13 N1
Hex2Cer(d40:0+O)	Hex2Cer	M+H	964.7294855	16.12923647	C52 H102 O14 N1
Hex2Cer(d41:0)	Hex2Cer	M+H	962.7502205	17.62212918	C53 H104 O13 N1
Hex2Cer(d42:0)	Hex2Cer	M+H	976.7658705	18.06	C54 H106 O13 N1
Hex2Cer(d42:1)	Hex2Cer	M+H	974.7502205	17.03936869	C54 H104 O13 N1
Hex2Cer(d42:2)	Hex2Cer	M+HCOO	1016.725498	16.394	C55 H102 O15 N1
Hex2Cer(m32:0+O)	Hex2Cer	M+H	836.6093705	11.67453828	C44 H86 O13 N1
Hex2Cer(m34:0+O)	Hex2Cer	M+H	864.6406705	13.209116	C46 H90 O13 N1
Hex2Cer(m36:0+O)	Hex2Cer	M+H	892.6719705	14.64327852	C48 H94 O13 N1
Hex2Cer(m38:0+O)	Hex2Cer	M+H	920.7032705	15.9219417	C50 H98 O13 N1
Hex2Cer(m39:0+O)	Hex2Cer	M+H	934.7189205	16.53685645	C51 H100 O13 N1
Hex2Cer(m40:0+O)	Hex2Cer	M+H	948.7345705	17.10807406	C52 H102 O13 N1

Hex2Cer(m41:0+O)	Hex2Cer	M+H	962.7502205	17.6006577	C53 H104 O13 N1
Hex2Cer(m42:0+O)	Hex2Cer	M+H	976.7658705	18.04031366	C54 H106 O13 N1
Hex2Cer(m43:0+O)	Hex2Cer	M+H	990.7815205	18.448	C55 H108 O13 N1
Hex2Cer(t34:0)	Hex2Cer	M+H	880.6355855	11.97102783	C46 H90 O14 N1
Hex2Cer(t34:2)	Hex2Cer	M-H	874.5897325	14.59153315	C46 H84 O14 N1
Hex2Cer(t40:0)	Hex2Cer	M+H	964.7294855	16.10885808	C52 H102 O14 N1
Hex2Cer(t41:0)	Hex2Cer	M+H	978.7451355	16.71	C53 H104 O14 N1
Hex2Cer(t42:0)	Hex2Cer	M+H	992.7607855	17.04852963	C54 H106 O14 N1
Hex3Cer(d34:2)	Hex3Cer	M+HCOO	1066.653123	13.702	C53 H96 O20 N1
LBPA(16:0_16:0)	LBPA	M-H	721.5025115	13.095	C38 H74 O10 P1
LBPA(16:0_18:0)	LBPA	M-H	749.5338115	14.44403181	C40 H78 O10 P1
LPC(12:0)	LPC	M+H	440.2771685	1.517045381	C20 H43 O7 N1 P1
LPC(14:0)	LPC	M+H	468.3084685	2.121303294	C22 H47 O7 N1 P1
LPC(15:0)	LPC	M+HCOO	526.3150455	2.431025424	C24 H49 O9 N1 P1
LPC(16:0e)	LPC	M+H	482.3605035	3.912836583	C24 H53 O6 N1 P1
LPC(16:1)	LPC	M+H	494.3241185	2.293961544	C24 H49 O7 N1 P1
LPC(16:1e)	LPC	M+H	480.3448535	3.732941794	C24 H51 O6 N1 P1
LPC(17:0)	LPC	M+H	510.3554185	4.036969901	C25 H53 O7 N1 P1
LPC(17:1)	LPC	M+H	508.3397685	2.81014016	C25 H51 O7 N1 P1
LPC(18:0)	LPC	M+H	524.3710685	4.977370114	C26 H55 O7 N1 P1
LPC(18:0e)	LPC	M+H	510.3918035	5.898070703	C26 H57 O6 N1 P1
LPC(18:1)	LPC	M+Na	544.3373635	3.470075226	C26 H52 O7 N1 P1 Na1
LPC(18:1e)	LPC	M+H	508.3761535	4.120647318	C26 H55 O6 N1 P1
LPC(18:2)	LPC	M+H	520.3397685	2.351307905	C26 H51 O7 N1 P1
LPC(18:3)	LPC	M+H	518.3241185	1.96395333	C26 H49 O7 N1 P1

LPC(20:0)	LPC	M+H	552.4023685	5.369470166	C28 H59 O7 N1 P1
LPC(20:1)	LPC	M+H	550.3867185	5.172561945	C28 H57 O7 N1 P1
LPC(20:3)	LPC	M+H	546.3554185	2.673483274	C28 H53 O7 N1 P1
LPC(20:4)	LPC	M+H	544.3397685	3.465773862	C28 H51 O7 N1 P1
LPC(22:5)	LPC	M+H	570.3554185	2.571	C30 H53 O7 N1 P1
LPC(30:0)	LPC	M+Na	714.5408135	13.487	C38 H78 O7 N1 P1 Na1
LPC(32:1)	LPC	M+Na	740.5564635	14.72806383	C40 H80 O7 N1 P1 Na1
LPE(16:0)	LPE	M-H	452.2782655	3.423030295	C21 H43 O7 N1 P1
LPE(18:0)	LPE	M+Na	504.3060635	2.825	C23 H48 O7 N1 P1 Na1
LPE(18:1)	LPE	M+Na	502.2904135	3.627976557	C23 H46 O7 N1 P1 Na1
LPE(18:2)	LPE	M-H	476.2782655	2.651862016	C23 H43 O7 N1 P1
LPE(20:2)	LPE	M+Na	528.3060635	2.502401741	C25 H48 O7 N1 P1 Na1
LPE(20:3)	LPE	M-H	502.2939155	2.827877749	C25 H45 O7 N1 P1
LPE(20:4)	LPE	M-H	500.2782655	2.378432756	C25 H43 O7 N1 P1
LdMePE(16:0)	LdMePE	M-H	480.3095655	3.255145433	C23 H47 O7 N1 P1
LdMePE(18:0)	LdMePE	M-H	508.3408655	4.974749271	C25 H51 O7 N1 P1
LdMePE(18:1)	LdMePE	M-H	506.3252155	3.194	C25 H49 O7 N1 P1
LdMePE(18:2)	LdMePE	M-H	504.3095655	2.358	C25 H47 O7 N1 P1
MG(32:0)	MG	M+Na	577.5166315	24.604	C35 H70 O4 Na1
MGDG(14:0e_23:0)	MGDG	M+HCOO	831.6566885	17.23576052	C47 H91 O11
MGDG(16:0_16:0)	MGDG	M+HCOO	775.5577035	14.944	C42 H79 O12
MGDG(18:1_20:3)	MGDG	M-H	805.5835235	14.12343083	C47 H81 O10
MGDG(18:1_22:4)	MGDG	M-H	831.5991735	14.17656011	C49 H83 O10
MGDG(18:1_22:5)	MGDG	M-H	829.5835235	13.04363008	C49 H81 O10
MGDG(29:0e)	MGDG	M+CH3COO	733.5471385	11.60815075	C40 H77 O11

MGDG(30:0e)	MGDG	M+CH3COO	747.5627885	12.65151881	C41 H79 O11
MGDG(31:0e)	MGDG	M+CH3COO	761.5784385	13.402	C42 H81 O11
MGDG(32:0e)	MGDG	M+CH3COO	775.5940885	14.13495743	C43 H83 O11
MGDG(32:1e)	MGDG	M+CH3COO	773.5784385	12.79222615	C43 H81 O11
MGDG(33:0e)	MGDG	M+CH3COO	789.6097385	14.85254897	C44 H85 O11
MGDG(34:0e)	MGDG	M+CH3COO	803.6253885	15.39474549	C45 H87 O11
MGDG(34:1e)	MGDG	M+CH3COO	801.6097385	14.28972625	C45 H85 O11
MGDG(34:3)	MGDG	M-H	751.5365735	12.599	C43 H75 O10
MGDG(35:0e)	MGDG	M+CH3COO	817.6410385	15.95252567	C46 H89 O11
MGDG(35:1e)	MGDG	M+CH3COO	815.6253885	14.73830483	C46 H87 O11
MGDG(36:0e)	MGDG	M+CH3COO	831.6566885	16.77447497	C47 H91 O11
MGDG(36:1e)	MGDG	M+CH3COO	829.6410385	15.40122356	C47 H89 O11
MGDG(36:2e)	MGDG	M+CH3COO	827.6253885	14.19493561	C47 H87 O11
MGDG(36:3)	MGDG	M-H	779.5678735	14.053	C45 H79 O10
MGDG(37:1e)	MGDG	M+CH3COO	843.6566885	16.28222063	C48 H91 O11
MGDG(38:0e)	MGDG	M+CH3COO	859.6879885	17.66804221	C49 H95 O11
MGDG(38:1e)	MGDG	M+CH3COO	857.6723385	16.47585519	C49 H93 O11
MGDG(38:2e)	MGDG	M+CH3COO	855.6566885	15.54196131	C49 H91 O11
MGDG(38:3)	MGDG	M-H	807.5991735	15.41344277	C47 H83 O10
MGDG(38:5)	MGDG	M-H	803.5678735	12.98310503	C47 H79 O10
MGDG(39:0e)	MGDG	M+CH3COO	873.7036385	18.12770254	C50 H97 O11
MGDG(40:0e)	MGDG	M+CH3COO	887.7192885	18.48878382	C51 H99 O11
MGDG(40:1e)	MGDG	M+CH3COO	885.7036385	17.76025842	C51 H97 O11
MGDG(40:4)	MGDG	M-H	833.6148235	15.46575914	C49 H85 O10
MGDG(40:5)	MGDG	M-H	831.5991735	14.476	C49 H83 O10

MGDG(42:3)	MGDG	M-H	863.6617735	16.4684854	C51 H91 O10
MGMG(35:0)	MGMG	M+CH3COO	817.6410385	16.17448054	C46 H89 O11
MGMG(36:0)	MGMG	M+CH3COO	831.6566885	16.80862986	C47 H91 O11
MGMG(37:0)	MGMG	M+CH3COO	845.6723385	17.16305624	C48 H93 O11
PA(18:0_18:1)	PA	M-H	701.5126815	23.496	C39 H74 O8 N0 P1
PA(18:0_18:2)	PA	M-H	699.4970315	23.3327116	C39 H72 O8 N0 P1
PC(12:0_12:0)	PC	M+HCOO	666.4351605	8.026206845	C33 H65 O10 N1 P1
PC(12:0_14:0)	PC	M+HCOO	694.4664605	9.581271838	C35 H69 O10 N1 P1
PC(12:1e_20:3)	PC	M+H	712.5275685	13.34752552	C40 H75 O7 N1 P1
PC(14:0_14:0)	PC	M+Na	700.4887785	11.08568557	C36 H72 O8 N1 P1 Na1
PC(14:0_18:2)	PC	M+Na	752.5200785	11.52058634	C40 H76 O8 N1 P1 Na1
PC(14:0_18:3)	PC	M+HCOO	772.5134105	12.644	C41 H75 O10 N1 P1
PC(14:1e_16:0)	PC	M+HCOO	734.5341455	13.13217992	C39 H77 O9 N1 P1
PC(14:1e_20:4)	PC	M+H	738.5432185	14.15645083	C42 H77 O7 N1 P1
PC(15:0_14:0)	PC	M+Na	714.5044285	11.8888053	C37 H74 O8 N1 P1 Na1
PC(15:0_16:0)	PC	M+Na	742.5357285	13.07854514	C39 H78 O8 N1 P1 Na1
PC(15:0_18:1)	PC	M+Na	768.5513785	13.13494094	C41 H80 O8 N1 P1 Na1
PC(15:0_18:2)	PC	M+HCOO	788.5447105	12.25789051	C42 H79 O10 N1 P1
PC(15:0_20:4)	PC	M+Na	790.5357285	11.99321562	C43 H78 O8 N1 P1 Na1
PC(16:0_14:0)	PC	M+HCOO	750.5290605	12.59690403	C39 H77 O10 N1 P1
PC(16:0_14:4)	PC	M+CH3COO	756.4821105	13.05346753	C40 H71 O10 N1 P1
PC(16:0_16:0)	PC	M+HCOO	778.5603605	14.05554953	C41 H81 O10 N1 P1
PC(16:0_16:1)	PC	M+Na	754.5357285	12.97686529	C40 H78 O8 N1 P1 Na1
PC(16:0_17:0)	PC	M+Na	770.5670285	14.48303112	C41 H82 O8 N1 P1 Na1
PC(16:0_18:1)	PC	M+HCOO	804.5760105	14.1257297	C43 H83 O10 N1 P1

PC(16:0_18:3)	PC	M+Na	778.5357285	11.96160954	C42 H78 O8 N1 P1 Na1
PC(16:0_20:4)	PC	M+HCOO	826.5603605	12.71260151	C45 H81 O10 N1 P1
PC(16:0_20:5)	PC	M+HCOO	824.5447105	11.71214611	C45 H79 O10 N1 P1
PC(16:0_22:4)	PC	M+Na	832.5826785	13.69625808	C46 H84 O8 N1 P1 Na1
PC(16:0_22:6)	PC	M+HCOO	850.5603605	12.26015983	C47 H81 O10 N1 P1
PC(16:0e_16:0)	PC	M+HCOO	764.5810955	14.92033309	C41 H83 O9 N1 P1
PC(16:0e_18:2)	PC	M+HCOO	788.5810955	13.84956302	C43 H83 O9 N1 P1
PC(16:0e_20:3)	PC	M+HCOO	814.5967455	14.18094172	C45 H85 O9 N1 P1
PC(16:1_18:2)	PC	M+HCOO	800.5447105	11.62081411	C43 H79 O10 N1 P1
PC(16:1e_18:0)	PC	M+HCOO	790.5967455	15.88482998	C43 H85 O9 N1 P1
PC(16:1e_18:1)	PC	M+HCOO	788.5810955	14.77563162	C43 H83 O9 N1 P1
PC(17:0_18:1)	PC	M+HCOO	818.5916605	14.80560364	C44 H85 O10 N1 P1
PC(17:0_18:2)	PC	M+Na	794.5670285	13.46696855	C43 H82 O8 N1 P1 Na1
PC(17:0_18:3)	PC	M+HCOO	814.5603605	14.72418591	C44 H81 O10 N1 P1
PC(17:0_20:4)	PC	M+HCOO	840.5760105	13.42249968	C46 H83 O10 N1 P1
PC(17:1_16:0)	PC	M+Na	768.5513785	14.44879456	C41 H80 O8 N1 P1 Na1
PC(17:1_18:1)	PC	M+HCOO	816.5760105	15.73749575	C44 H83 O10 N1 P1
PC(17:1_20:5)	PC	M+Na	814.5357285	15.033	C45 H78 O8 N1 P1 Na1
PC(18:0_14:4)	PC	M+CH3COO	784.5134105	14.461	C42 H75 O10 N1 P1
PC(18:0_15:0)	PC	M+HCOO	792.5760105	14.7522829	C42 H83 O10 N1 P1
PC(18:0_16:0)	PC	M+HCOO	806.5916605	15.41790644	C43 H85 O10 N1 P1
PC(18:0_17:0)	PC	M+HCOO	820.6073105	15.87238445	C44 H87 O10 N1 P1
PC(18:0_18:0)	PC	M+HCOO	834.6229605	16.67121778	C45 H89 O10 N1 P1
PC(18:0_18:1)	PC	M+CH3COO	846.6229605	16.08	C46 H89 O10 N1 P1
PC(18:0_18:2)	PC	M+HCOO	830.5916605	14.474	C45 H85 O10 N1 P1

PC(18:0_18:3)	PC	M+Na	806.5670285	13.35462676	C44 H82 O8 N1 P1 Na1
PC(18:0_20:3)	PC	M+Na	834.5983285	14.70060128	C46 H86 O8 N1 P1 Na1
PC(18:0_20:4)	PC	M+HCOO	854.5916605	14.10625683	C47 H85 O10 N1 P1
PC(18:0_20:5)	PC	M+Na	830.5670285	13.14464217	C46 H82 O8 N1 P1 Na1
PC(18:0_22:4)	PC	M+HCOO	882.6229605	15.04837606	C49 H89 O10 N1 P1
PC(18:0_22:5)	PC	M+HCOO	880.6073105	14.09950911	C49 H87 O10 N1 P1
PC(18:0_22:6)	PC	M+HCOO	878.5916605	13.67159285	C49 H85 O10 N1 P1
PC(18:0e_16:0)	PC	M+HCOO	792.6123955	16.22921511	C43 H87 O9 N1 P1
PC(18:0e_18:2)	PC	M+HCOO	816.6123955	15.21342645	C45 H87 O9 N1 P1
PC(18:0e_20:4)	PC	M+HCOO	840.6123955	14.92939188	C47 H87 O9 N1 P1
PC(18:1_12:0)	PC	M+Na	726.5044285	11.20688248	C38 H74 O8 N1 P1 Na1
PC(18:1_14:0)	PC	M+HCOO	776.5447105	12.70166228	C41 H79 O10 N1 P1
PC(18:1_18:1)	PC	M+Na	808.5826785	14.19903553	C44 H84 O8 N1 P1 Na1
PC(18:1_18:2)	PC	M+Na	806.5670285	13.05200056	C44 H82 O8 N1 P1 Na1
PC(18:1_18:3)	PC	M+CH3COO	840.5760105	14.554	C46 H83 O10 N1 P1
PC(18:1_20:3)	PC	M+Na	832.5826785	13.37769907	C46 H84 O8 N1 P1 Na1
PC(18:1_20:4)	PC	M+Na	830.5670285	12.73233503	C46 H82 O8 N1 P1 Na1
PC(18:1_22:4)	PC	M+HCOO	880.6073105	13.744	C49 H87 O10 N1 P1
PC(18:1_22:5)	PC	M+HCOO	878.5916605	12.78227074	C49 H85 O10 N1 P1
PC(18:1e_16:0)	PC	M+CH3COO	804.6123955	16.49489984	C44 H87 O9 N1 P1
PC(18:1e_18:0)	PC	M+CH3COO	832.6436955	17.60174598	C46 H91 O9 N1 P1
PC(18:1e_18:2)	PC	M+HCOO	814.5967455	13.85632021	C45 H85 O9 N1 P1
PC(18:2_18:2)	PC	M+Na	804.5513785	11.92599654	C44 H80 O8 N1 P1 Na1
PC(18:2_20:4)	PC	M+HCOO	850.5603605	11.59442082	C47 H81 O10 N1 P1
PC(18:2e_15:0)	PC	M+CH3COO	788.5810955	14.77250421	C43 H83 O9 N1 P1

PC(18:3e_16:0)	PC	M+CH3COO	800.5810955	14.069	C44 H83 O9 N1 P1
PC(19:0_18:1)	PC	M+HCOO	846.6229605	16.1043035	C46 H89 O10 N1 P1
PC(19:1_18:1)	PC	M+HCOO	844.6073105	14.83407228	C46 H87 O10 N1 P1
PC(20:0_14:4)	PC	M+CH3COO	812.5447105	15.79451687	C44 H79 O10 N1 P1
PC(20:0_18:1)	PC	M+HCOO	860.6386105	15.61229982	C47 H91 O10 N1 P1
PC(20:0_18:2)	PC	M+Na	836.6139785	14.57135842	C46 H88 O8 N1 P1 Na1
PC(20:0_18:3)	PC	M+HCOO	856.6073105	13.57027991	C47 H87 O10 N1 P1
PC(20:0_20:4)	PC	M+HCOO	882.6229605	14.26706541	C49 H89 O10 N1 P1
PC(20:1_18:1)	PC	M+HCOO	858.6229605	15.54397871	C47 H89 O10 N1 P1
PC(20:3_18:2)	PC	M+HCOO	852.5760105	12.2271279	C47 H83 O10 N1 P1
PC(20:4e_18:0)	PC	M+H	796.6214685	15.303269	C46 H87 O7 N1 P1
PC(22:0_14:2)	PC	M+CH3COO	844.6073105	16.91	C46 H87 O10 N1 P1
PC(25:0)	PC	M+Na	658.4418285	8.792	C33 H66 O8 N1 P1 Na1
PC(28:0)	PC	M+H	678.5068335	12.835	C36 H73 O8 N1 P1
PC(29:0)	PC	M+Na	714.5044285	11.58466674	C37 H74 O8 N1 P1 Na1
PC(29:1)	PC	M+H	690.5068335	13.06697699	C37 H73 O8 N1 P1
PC(29:2)	PC	M+H	688.4911835	11.995	C37 H71 O8 N1 P1
PC(30:0)	PC	M+Na	728.5200785	13.015	C38 H76 O8 N1 P1 Na1
PC(30:0e)	PC	M+Na	714.5408135	13.491	C38 H78 O7 N1 P1 Na1
PC(31:0)	PC	M+Na	742.5357285	13.38856044	C39 H78 O8 N1 P1 Na1
PC(31:0e)	PC	M+Na	728.5564635	14.23874598	C39 H80 O7 N1 P1 Na1
PC(31:1)	PC	M+Na	740.5200785	12.373	C39 H76 O8 N1 P1 Na1
PC(31:2)	PC	M+H	716.5224835	13.30260326	C39 H75 O8 N1 P1
PC(31:3)	PC	M+H	714.5068335	11.99324244	C39 H73 O8 N1 P1
PC(32:0)	PC	M+H	734.5694335	26.7	C40 H81 O8 N1 P1

PC(32:0e)	PC	M+Na	742.5721135	14.92033309	C40 H82 O7 N1 P1 Na1
PC(32:1)	PC	M+H	732.5537835	14.91175916	C40 H79 O8 N1 P1
PC(32:2)	PC	M+H	730.5381335	13.84145396	C40 H77 O8 N1 P1
PC(32:2e)	PC	M+CH3COO	774.5654455	13.79194088	C42 H81 O9 N1 P1
PC(32:3e)	PC	M+CH3COO	772.5497955	12.62016409	C42 H79 O9 N1 P1
PC(32:5e)	PC	M+H	710.5119185	12.723	C40 H73 O7 N1 P1
PC(32:6e)	PC	M+H	708.4962685	11.99	C40 H71 O7 N1 P1
PC(33:1e)	PC	M+H	732.5901685	15.14800004	C41 H83 O7 N1 P1
PC(33:2)	PC	M+H	744.5537835	14.52664763	C41 H79 O8 N1 P1
PC(33:2e)	PC	M+H	730.5745185	12.91	C41 H81 O7 N1 P1
PC(33:3)	PC	M+H	742.5381335	13.74073184	C41 H77 O8 N1 P1
PC(33:4)	PC	M+H	740.5224835	12.35783777	C41 H75 O8 N1 P1
PC(33:4e)	PC	M+H	726.5432185	14.093	C41 H77 O7 N1 P1
PC(33:7)	PC	M+H	734.4755335	14.887	C41 H69 O8 N1 P1
PC(34:0)	PC	M+Na	784.5826785	15.41790644	C42 H84 O8 N1 P1 Na1
PC(34:0e)	PC	M+Na	770.6034135	16.22921511	C42 H86 O7 N1 P1 Na1
PC(34:1e)	PC	M+Na	768.5877635	14.91477468	C42 H84 O7 N1 P1 Na1
PC(34:3e)	PC	M+H	742.5745185	13.63305866	C42 H81 O7 N1 P1
PC(34:4e)	PC	M+H	740.5588685	14.709	C42 H79 O7 N1 P1
PC(35:1)	PC	M+Na	796.5826785	14.80560364	C43 H84 O8 N1 P1 Na1
PC(35:2)	PC	M+Na	794.5670285	14.44462974	C43 H82 O8 N1 P1 Na1
PC(35:2e)	PC	M+H	758.6058185	15.2	C43 H85 O7 N1 P1
PC(35:3)	PC	M+H	770.5694335	15.03899978	C43 H81 O8 N1 P1
PC(35:3e)	PC	M+Na	778.5721135	15.068	C43 H82 O7 N1 P1 Na1
PC(35:4)	PC	M+H	768.5537835	11.99321562	C43 H79 O8 N1 P1

PC(35:4e)	PC	M+Na	776.5564635	12.95651666	C43 H80 O7 N1 P1 Na1
PC(35:5)	PC	M+H	766.5381335	13.108	C43 H77 O8 N1 P1
PC(36:1)	PC	M+H	788.6163835	15.46311233	C44 H87 O8 N1 P1
PC(36:2e)	PC	M+Na	794.6034135	15.21342645	C44 H86 O7 N1 P1 Na1
PC(36:3)	PC	M+H	784.5850835	15.43545749	C44 H83 O8 N1 P1
PC(36:4)	PC	M+H	782.5694335	14.113	C44 H81 O8 N1 P1
PC(36:4e)	PC	M+H	768.5901685	13.569	C44 H83 O7 N1 P1
PC(36:5)	PC	M+Na	802.5357285	10.852	C44 H78 O8 N1 P1 Na1
PC(36:5e)	PC	M+H	766.5745185	13.33147706	C44 H81 O7 N1 P1
PC(37:3)	PC	M+H	798.6007335	14.0430143	C45 H85 O8 N1 P1
PC(37:4)	PC	M+H	796.5850835	14.801	C45 H83 O8 N1 P1
PC(37:4e)	PC	M+H	782.6058185	14.386	C45 H85 O7 N1 P1
PC(37:5)	PC	M+H	794.5694335	14.10242337	C45 H81 O8 N1 P1
PC(37:5e)	PC	M+H	780.5901685	16.0002371	C45 H83 O7 N1 P1
PC(37:6)	PC	M+H	792.5537835	13.142	C45 H79 O8 N1 P1
PC(38:2)	PC	M+H	814.6320335	14.57135842	C46 H89 O8 N1 P1
PC(38:3)	PC	M+Na	834.5983285	14.36582133	C46 H86 O8 N1 P1 Na1
PC(38:3e)	PC	M+Na	820.6190635	15.51225995	C46 H88 O7 N1 P1 Na1
PC(38:4)	PC	M+H	810.6007335	15.463	C46 H85 O8 N1 P1
PC(38:4e)	PC	M+H	796.6214685	14.92939188	C46 H87 O7 N1 P1
PC(38:5e)	PC	M+H	794.6058185	13.56693396	C46 H85 O7 N1 P1
PC(38:6)	PC	M+Na	828.5513785	12.26015983	C46 H80 O8 N1 P1 Na1
PC(38:6e)	PC	M+H	792.5901685	13.32817729	C46 H83 O7 N1 P1
PC(39:4)	PC	M+H	824.6163835	14.784	C47 H87 O8 N1 P1
PC(39:5e)	PC	M+H	808.6214685	14.719	C47 H87 O7 N1 P1

PC(40:4e)	PC	M+Na	846.6347135	15.821	C48 H90 O7 N1 P1 Na1
PC(40:5e)	PC	M+Na	844.6190635	14.925	C48 H88 O7 N1 P1 Na1
PC(40:6e)	PC	M+H	820.6214685	13.542	C48 H87 O7 N1 P1
PC(40:7)	PC	M+Na	854.5670285	11.903	C48 H82 O8 N1 P1 Na1
PC(44:11)	PC	M+H	880.5850835	12.989	C52 H83 O8 N1 P1
PC(51:2)	PC	M+H	996.8354835	22.308	C59 H115 O8 N1 P1
PC(8:0e_24:1)	PC	M+Na	740.5564635	14.70962401	C40 H80 O7 N1 P1 Na1
PE(14:0_18:2)	PE	M-H	686.4766305	11.82355944	C37 H69 O8 N1 P1
PE(14:0_20:4)	PE	M-H	710.4766305	11.56891943	C39 H69 O8 N1 P1
PE(14:1e_18:1)	PE	M-H	672.4973655	13.75447611	C37 H71 O7 N1 P1
PE(14:1e_18:2)	PE	M-H	670.4817155	12.55619674	C37 H69 O7 N1 P1
PE(14:1e_20:4)	PE	M-H	694.4817155	12.09966853	C39 H69 O7 N1 P1
PE(15:0_16:0)	PE	M-H	676.4922805	13.42385526	C36 H71 O8 N1 P1
PE(15:0_18:1)	PE	M-H	702.5079305	13.77696445	C38 H73 O8 N1 P1
PE(15:0_18:2)	PE	M-H	700.4922805	12.6080077	C38 H71 O8 N1 P1
PE(16:0_12:0)	PE	M+H	636.4598835	11.46091841	C33 H67 O8 N1 P1
PE(16:0_14:0)	PE	M-H	662.4766305	12.96724237	C35 H69 O8 N1 P1
PE(16:0_16:0)	PE	M-H	690.5079305	14.41815682	C37 H73 O8 N1 P1
PE(16:0_18:1)	PE	M+H	718.5381335	14.48144738	C39 H77 O8 N1 P1
PE(16:0_18:2)	PE	M+Na	738.5044285	13.32034624	C39 H74 O8 N1 P1 Na1
PE(16:0_18:3)	PE	M-H	712.4922805	12.29784271	C39 H71 O8 N1 P1
PE(16:0_20:4)	PE	M-H	738.5079305	13.04771352	C41 H73 O8 N1 P1
PE(16:0_20:5)	PE	M+H	738.5068335	12.04532661	C41 H73 O8 N1 P1
PE(16:0_22:6)	PE	M-H	762.5079305	12.59587871	C43 H73 O8 N1 P1
PE(16:0e_18:1)	PE	M+Na	726.5408135	14.088	C39 H78 O7 N1 P1 Na1

PE(16:0e_18:2)	PE	M-H	700.5286655	14.227	C39 H75 O7 N1 P1
PE(16:1_18:2)	PE	M-H	712.4922805	11.99017816	C39 H71 O8 N1 P1
PE(16:1e_16:0)	PE	M-H	674.5130155	15.12333864	C37 H73 O7 N1 P1
PE(16:1e_18:1)	PE	M-H	700.5286655	15.15239837	C39 H75 O7 N1 P1
PE(16:1e_18:2)	PE	M-H	698.5130155	14.02239775	C39 H73 O7 N1 P1
PE(16:1e_18:3)	PE	M-H	696.4973655	12.98235842	C39 H71 O7 N1 P1
PE(16:1e_20:3)	PE	M-H	724.5286655	14.77101956	C41 H75 O7 N1 P1
PE(16:1e_20:4)	PE	M-H	722.5130155	13.67682893	C41 H73 O7 N1 P1
PE(16:1e_20:5)	PE	M-H	720.4973655	12.69523069	C41 H71 O7 N1 P1
PE(16:1e_22:4)	PE	M-H	750.5443155	14.68515763	C43 H77 O7 N1 P1
PE(16:1e_22:5)	PE	M-H	748.5286655	13.71949674	C43 H75 O7 N1 P1
PE(17:0_18:1)	PE	M+H	732.5537835	14.9834673	C40 H79 O8 N1 P1
PE(17:0_18:2)	PE	M-H	728.5235805	13.83611025	C40 H75 O8 N1 P1
PE(17:0_20:3)	PE	M-H	754.5392305	14.36	C42 H77 O8 N1 P1
PE(17:0_20:4)	PE	M-H	752.5235805	13.74105276	C42 H75 O8 N1 P1
PE(17:1_18:2)	PE	M-H	726.5079305	12.66929523	C40 H73 O8 N1 P1
PE(18:0_15:0)	PE	M-H	704.5235805	14.81203046	C38 H75 O8 N1 P1
PE(18:0_16:0)	PE	M-H	718.5392305	15.75655619	C39 H77 O8 N1 P1
PE(18:0_18:0)	PE	M-H	746.5705305	16.9748115	C41 H81 O8 N1 P1
PE(18:0_18:1)	PE	M+H	746.5694335	15.8018813	C41 H81 O8 N1 P1
PE(18:0_18:2)	PE	M-H	742.5392305	14.815	C41 H77 O8 N1 P1
PE(18:0_18:3)	PE	M-H	740.5235805	13.969	C41 H75 O8 N1 P1
PE(18:0_20:3)	PE	M-H	768.5548805	15.03330531	C43 H79 O8 N1 P1
PE(18:0_20:4)	PE	M-H	766.5392305	14.44656771	C43 H77 O8 N1 P1
PE(18:0_20:5)	PE	M-H	764.5235805	13.48046834	C43 H75 O8 N1 P1

PE(18:0_21:1)	PE	M-H	786.6018305	17.497	C44 H85 O8 N1 P1
PE(18:0_22:4)	PE	M-H	794.5705305	15.37561565	C45 H81 O8 N1 P1
PE(18:0_22:5)	PE	M+H	794.5694335	14.43905426	C45 H81 O8 N1 P1
PE(18:0_22:6)	PE	M-H	790.5392305	14.00376941	C45 H77 O8 N1 P1
PE(18:0e_18:1)	PE	M-H	730.5756155	16.63248521	C41 H81 O7 N1 P1
PE(18:0e_18:2)	PE	M-H	728.5599655	15.58977073	C41 H79 O7 N1 P1
PE(18:0e_20:3)	PE	M-H	754.5756155	15.871	C43 H81 O7 N1 P1
PE(18:1_12:0)	PE	M+H	662.4755335	11.53571783	C35 H69 O8 N1 P1
PE(18:1_14:0)	PE	M-H	688.4922805	13.05879581	C37 H71 O8 N1 P1
PE(18:1_18:1)	PE	M+H	744.5537835	14.52415986	C41 H79 O8 N1 P1
PE(18:1_18:2)	PE	M+H	742.5381335	13.39687283	C41 H77 O8 N1 P1
PE(18:1_18:3)	PE	M+H	740.5224835	12.35944279	C41 H75 O8 N1 P1
PE(18:1_20:3)	PE	M-H	766.5392305	13.71667516	C43 H77 O8 N1 P1
PE(18:1_20:4)	PE	M-H	764.5235805	13.09869023	C43 H75 O8 N1 P1
PE(18:1_20:5)	PE	M-H	762.5079305	12.03156745	C43 H73 O8 N1 P1
PE(18:1_22:0)	PE	M-H	800.6174805	17.93928158	C45 H87 O8 N1 P1
PE(18:1_22:4)	PE	M-H	792.5548805	14.0806587	C45 H79 O8 N1 P1
PE(18:1_22:5)	PE	M+H	792.5537835	13.12403746	C45 H79 O8 N1 P1
PE(18:1_22:6)	PE	M+H	790.5381335	12.66484157	C45 H77 O8 N1 P1
PE(18:1e_16:0)	PE	M-H	702.5443155	16.428677	C39 H77 O7 N1 P1
PE(18:1e_17:1)	PE	M-H	714.5443155	15.80909996	C40 H77 O7 N1 P1
PE(18:1e_18:1)	PE	M-H	728.5599655	16.45276776	C41 H79 O7 N1 P1
PE(18:1e_18:2)	PE	M-H	726.5443155	15.484	C41 H77 O7 N1 P1
PE(18:1e_20:1)	PE	M-H	756.5912655	17.53080802	C43 H83 O7 N1 P1
PE(18:1e_20:3)	PE	M-H	752.5599655	15.66315726	C43 H79 O7 N1 P1

PE(18:1e_20:4)	PE	M-H	750.5443155	15.08079313	C43 H77 O7 N1 P1
PE(18:1e_20:5)	PE	M-H	748.5286655	14.13256293	C43 H75 O7 N1 P1
PE(18:1e_22:4)	PE	M-H	778.5756155	15.98254555	C45 H81 O7 N1 P1
PE(18:1e_22:5)	PE	M-H	776.5599655	15.06360223	C45 H79 O7 N1 P1
PE(18:1e_22:6)	PE	M-H	774.5443155	14.61507328	C45 H77 O7 N1 P1
PE(18:2e_18:2)	PE	M-H	724.5286655	14.07581094	C41 H75 O7 N1 P1
PE(18:2e_18:3)	PE	M-H	722.5130155	13.056411	C41 H73 O7 N1 P1
PE(18:2e_20:3)	PE	M-H	750.5443155	14.39295237	C43 H77 O7 N1 P1
PE(18:2e_22:4)	PE	M-H	776.5599655	14.733	C45 H79 O7 N1 P1
PE(18:2e_22:5)	PE	M-H	774.5443155	13.75795754	C45 H77 O7 N1 P1
PE(18:3_18:2)	PE	M-H	736.4922805	11.19464895	C41 H71 O8 N1 P1
PE(19:0_18:1)	PE	M-H	758.5705305	16.41702057	C42 H81 O8 N1 P1
PE(19:1_15:0)	PE	M+Na	740.5200785	13.042	C39 H76 O8 N1 P1 Na1
PE(19:1_18:1)	PE	M-H	756.5548805	15.17743009	C42 H79 O8 N1 P1
PE(20:0_18:1)	PE	M+H	774.6007335	16.99239841	C43 H85 O8 N1 P1
PE(20:1_18:1)	PE	M+H	772.5850835	15.81636376	C43 H83 O8 N1 P1
PE(20:1_18:2)	PE	M-H	768.5548805	14.683	C43 H79 O8 N1 P1
PE(20:2_18:2)	PE	M+Na	790.5357285	11.99140193	C43 H78 O8 N1 P1 Na1
PE(20:3_18:2)	PE	M-H	764.5235805	12.642	C43 H75 O8 N1 P1
PE(20:3e_18:2)	PE	M-H	750.5443155	11.858	C43 H77 O7 N1 P1
PE(22:5_18:2)	PE	M+H	790.5381335	11.9985302	C45 H77 O8 N1 P1
PE(28:1)	PE	M+H	634.4442335	10.03958941	C33 H65 O8 N1 P1
PE(29:0)	PE	M+H	650.4755335	11.99444478	C34 H69 O8 N1 P1
PE(30:0)	PE	M+H	664.4911835	12.96724237	C35 H71 O8 N1 P1
PE(31:0e)	PE	M+Na	686.5095135	13.04907718	C36 H74 O7 N1 P1 Na1

PE(31:1)	PE	M+H	676.4911835	12.023	C36 H71 O8 N1 P1
PE(32:0)	PE	M+Na	714.5044285	12.00221151	C37 H74 O8 N1 P1 Na1
PE(32:1)	PE	M+Na	712.4887785	10.92396747	C37 H72 O8 N1 P1 Na1
PE(32:1p)	PE	M+H	674.5119185	13.779	C37 H73 O7 N1 P1
PE(32:3e)	PE	M+Na	694.4782135	12.55619674	C37 H70 O7 N1 P1 Na1
PE(33:0e)	PE	M+Na	714.5408135	14.482	C38 H78 O7 N1 P1 Na1
PE(33:1)	PE	M+H	704.5224835	13.77696445	C38 H75 O8 N1 P1
PE(33:2e)	PE	M+H	688.5275685	14.215	C38 H75 O7 N1 P1
PE(34:0)	PE	M+Na	742.5357285	13.398	C39 H78 O8 N1 P1 Na1
PE(34:0e)	PE	M+Na	728.5564635	15.398	C39 H80 O7 N1 P1 Na1
PE(34:1)	PE	M+Na	740.5200785	14.48144738	C39 H76 O8 N1 P1 Na1
PE(34:1e)	PE	M-H	702.5443155	15.369	C39 H77 O7 N1 P1
PE(34:1p)	PE	M+H	702.5432185	15.162	C39 H77 O7 N1 P1
PE(34:2)	PE	M+Na	738.5044285	12.038	C39 H74 O8 N1 P1 Na1
PE(34:2p)	PE	M+H	700.5275685	13.88079686	C39 H75 O7 N1 P1
PE(34:3)	PE	M+H	714.5068335	11.99017816	C39 H73 O8 N1 P1
PE(34:3p)	PE	M+H	698.5119185	12.975	C39 H73 O7 N1 P1
PE(34:4)	PE	M+H	712.4911835	10.92143007	C39 H71 O8 N1 P1
PE(34:4e)	PE	M-H	696.4973655	12.65941993	C39 H71 O7 N1 P1
PE(35:2)	PE	M+H	730.5381335	13.83611025	C40 H77 O8 N1 P1
PE(36:0)	PE	M+Na	770.5670285	15.03357983	C41 H82 O8 N1 P1 Na1
PE(36:1)	PE	M+Na	768.5513785	15.8018813	C41 H80 O8 N1 P1 Na1
PE(36:2)	PE	M+Na	766.5357285	14.52415986	C41 H78 O8 N1 P1 Na1
PE(36:2e)	PE	M+Na	752.5564635	15.58977073	C41 H80 O7 N1 P1 Na1
PE(36:3)	PE	M+Na	764.5200785	11.973	C41 H76 O8 N1 P1 Na1

PE(36:3e)	PE	M+H	728.5588685	15.20587593	C41 H79 O7 N1 P1
PE(36:3p)	PE	M+H	726.5432185	14.3344624	C41 H77 O7 N1 P1
PE(36:4)	PE	M+H	740.5224835	13.04771352	C41 H75 O8 N1 P1
PE(36:5e)	PE	M+Na	746.5095135	13.67682893	C41 H74 O7 N1 P1 Na1
PE(36:6)	PE	M+H	736.4911835	10.157	C41 H71 O8 N1 P1
PE(37:2e)	PE	M+Na	766.5721135	13.306	C42 H82 O7 N1 P1 Na1
PE(38:2)	PE	M+Na	794.5670285	14.426	C43 H82 O8 N1 P1 Na1
PE(38:3)	PE	M+Na	792.5513785	13.12780867	C43 H80 O8 N1 P1 Na1
PE(38:3p)	PE	M+H	754.5745185	16.094	C43 H81 O7 N1 P1
PE(38:4)	PE	M+H	768.5537835	14.44656771	C43 H79 O8 N1 P1
PE(38:5)	PE	M+H	766.5381335	13.09869023	C43 H77 O8 N1 P1
PE(38:5e)	PE	M+Na	774.5408135	15.08079313	C43 H78 O7 N1 P1 Na1
PE(38:8e)	PE	M+Na	768.4938635	16.077	C43 H72 O7 N1 P1 Na1
PE(40:4p)	PE	M+H	780.5901685	16.003	C45 H83 O7 N1 P1
PE(40:6)	PE	M+Na	814.5357285	15.024	C45 H78 O8 N1 P1 Na1
PE(44:6e)	PE	M+Na	856.6190635	19.738	C49 H88 O7 N1 P1 Na1
PE(52:1)	PE	M+Na	992.8017785	21.848	C57 H112 O8 N1 P1 Na1
PE(54:1)	PE	M+Na	1020.833078	22.074	C59 H116 O8 N1 P1 Na1
PE(58:2)	PE	M+H	1052.898083	22.716	C63 H123 O8 N1 P1
PG(16:0_14:0)	PG	M-H	693.4712115	11.65768478	C36 H70 O10 N0 P1
PG(16:0_16:0)	PG	M-H	721.5025115	13.089	C38 H74 O10 N0 P1
PG(16:0_18:1)	PG	M-H	747.5181615	13.16776315	C40 H76 O10 N0 P1
PG(18:0_18:1)	PG	M-H	775.5494615	14.51719499	C42 H80 O10 N0 P1
PG(18:1_14:0)	PG	M-H	719.4868615	11.74748918	C38 H72 O10 N0 P1
PG(18:1_18:1)	PG	M-H	773.5338115	13.30904218	C42 H78 O10 N0 P1

PG(35:0)	PG	M+Na	787.5459595	18.288	C41 H81 O10 N0 P1 Na1
PG(44:0)	PG	M+H	891.7048645	26.809	C50 H100 O10 N0 P1
PG(52:2)	PG	M+H	999.7987645	22.057	C58 H112 O10 N0 P1
PI(16:0_16:0)	PI	M-H	809.5185565	12.79627562	C41 H78 O13 N0 P1
PI(16:0_16:1)	PI	M-H	807.5029065	11.56553927	C41 H76 O13 N0 P1
PI(16:0_18:1)	PI	M-H	835.5342065	12.90099577	C43 H80 O13 N0 P1
PI(16:0_18:2)	PI	M-H	833.5185565	11.75273569	C43 H78 O13 N0 P1
PI(16:0_19:0)	PI	M-H	851.5655065	11.582	C44 H84 O13 N0 P1
PI(16:0_20:4)	PI	M-H	857.5185565	11.54021352	C45 H78 O13 N0 P1
PI(17:0_18:1)	PI	M-H	849.5498565	13.5752526	C44 H82 O13 N0 P1
PI(18:0_16:0)	PI	M-H	837.5498565	14.20050971	C43 H82 O13 N0 P1
PI(18:0_18:0)	PI	M-H	865.5811565	15.56868766	C45 H86 O13 N0 P1
PI(18:0_18:1)	PI	M-H	863.5655065	14.25498383	C45 H84 O13 N0 P1
PI(18:0_18:2)	PI	M-H	861.5498565	13.304	C45 H82 O13 N0 P1
PI(18:0_20:1)	PI	M-H	891.5968065	15.50887669	C47 H88 O13 N0 P1
PI(18:0_20:2)	PI	M-H	889.5811565	14.35269378	C47 H86 O13 N0 P1
PI(18:0_20:3)	PI	M-H	887.5655065	13.95747668	C47 H84 O13 N0 P1
PI(18:0_20:4)	PI	M+NH4	904.5909585	12.93926274	C47 H87 O13 N1 P1
PI(18:0_20:5)	PI	M-H	883.5342065	11.99295526	C47 H80 O13 N0 P1
PI(18:0_22:4)	PI	M-H	913.5811565	13.85184274	C49 H86 O13 N0 P1
PI(18:0_22:5)	PI	M-H	911.5655065	12.9263093	C49 H84 O13 N0 P1
PI(18:1_18:1)	PI	M+NH4	880.5909585	13.02149152	C45 H87 O13 N1 P1
PI(18:1_18:2)	PI	M+NH4	878.5753085	11.96562149	C45 H85 O13 N1 P1
PI(18:1_18:3)	PI	M-H	857.5185565	11.038	C45 H78 O13 N0 P1
PI(18:1_20:3)	PI	M-H	885.5498565	12.1998786	C47 H82 O13 N0 P1

PI(18:1_20:4)	PI	M+Na	907.5307045	11.61786884	C47 H81 O13 N0 P1 Na1
PI(19:1_18:0)	PI	M-H	877.5811565	14.88360741	C46 H86 O13 N0 P1
PI(34:0)	PI	M+Na	861.5463545	12.00913766	C43 H83 O13 N0 P1 Na1
PI(34:1)	PI	M+NH4	854.5753085	12.90099577	C43 H85 O13 N1 P1
PI(34:2)	PI	M+NH4	852.5596585	11.75273569	C43 H83 O13 N1 P1
PI(36:1)	PI	M+Na	887.5620045	12.93329638	C45 H85 O13 N0 P1 Na1
PI(36:3)	PI	M+H	861.5487595	11.96562149	C45 H82 O13 N0 P1
PI(38:4)	PI	M+H	887.5644095	12.93926274	C47 H84 O13 N0 P1
PI(38:5)	PI	M+NH4	902.5753085	11.61786884	C47 H85 O13 N1 P1
PIP(16:2)	PIP	M+Na	685.1996875	18.045	C25 H44 O16 N0 P2 Na1
PIP(19:0_18:2)	PIP	M-H	955.5318395	13.006	C46 H85 O16 N0 P2
PIP(38:5)	PIP	M-H	963.5005395	14.54526791	C47 H81 O16 N0 P2
PIP(42:8e)	PIP	M-H	999.5369245	14.25798455	C51 H85 O15 N0 P2
PMe(38:4e)	PMe	M-H	723.5334165	15.90435947	C42 H76 O7 N0 P1
PMe(40:6e)	PMe	M-H	747.5334165	15.21052176	C44 H76 O7 N0 P1
PS(16:0_18:1)	PS	M-H	760.5134105	13.08683011	C40 H75 O10 N1 P1
PS(16:0_18:2)	PS	M+H	760.5123135	11.98993453	C40 H75 O10 N1 P1
PS(18:0_16:0)	PS	M+Na	786.5255585	12.3552375	C40 H78 O10 N1 P1 Na1
PS(18:0_18:0)	PS	M+Na	814.5568585	13.68676004	C42 H82 O10 N1 P1 Na1
PS(18:0_18:1)	PS	M-H	788.5447105	14.55178467	C42 H79 O10 N1 P1
PS(18:0_18:2)	PS	M-H	786.5290605	13.54939427	C42 H77 O10 N1 P1
PS(18:0_18:3)	PS	M-H	784.5134105	12.3624123	C42 H75 O10 N1 P1
PS(18:0_20:2)	PS	M+Na	838.5568585	13.10483123	C44 H82 O10 N1 P1 Na1
PS(18:0_20:3)	PS	M-H	812.5447105	13.69130831	C44 H79 O10 N1 P1
PS(18:0_20:4)	PS	M-H	810.5290605	13.09988846	C44 H77 O10 N1 P1

PS(18:0_22:4)	PS	M+H	840.5749135	14.04797062	C46 H83 O10 N1 P1
PS(18:0_22:5)	PS	M-H	836.5447105	13.63270461	C46 H79 O10 N1 P1
PS(18:0_22:6)	PS	M+H	836.5436135	12.67493594	C46 H79 O10 N1 P1
PS(18:1_18:1)	PS	M-H	786.5290605	13.34585124	C42 H77 O10 N1 P1
PS(18:1_18:2)	PS	M+H	786.5279635	12.0244189	C42 H77 O10 N1 P1
PS(18:1_18:3)	PS	M-H	782.4977605	13.20163504	C42 H73 O10 N1 P1
PS(18:1_20:3)	PS	M-H	810.5290605	14.52433791	C44 H77 O10 N1 P1
PS(18:1_20:4)	PS	M-H	808.5134105	13.38930751	C44 H75 O10 N1 P1
PS(18:1_20:5)	PS	M-H	806.4977605	12.36189571	C44 H73 O10 N1 P1
PS(18:1_22:0)	PS	M-H	844.6073105	16.89671605	C46 H87 O10 N1 P1
PS(18:1_23:0)	PS	M+H	860.6375135	17.415	C47 H91 O10 N1 P1
PS(18:2_20:4)	PS	M-H	806.4977605	13.04533447	C44 H73 O10 N1 P1
PS(18:3_18:2)	PS	M-H	780.4821105	12.29343594	C42 H71 O10 N1 P1
PS(19:0_18:1)	PS	M-H	802.5603605	15.08959421	C43 H81 O10 N1 P1
PS(20:0_18:1)	PS	M-H	816.5760105	15.72818526	C44 H83 O10 N1 P1
PS(20:1_18:1)	PS	M-H	814.5603605	14.69474057	C44 H81 O10 N1 P1
PS(20:3_18:2)	PS	M-H	808.5134105	13.70954788	C44 H75 O10 N1 P1
PS(20:3_20:3)	PS	M-H	834.5290605	13.69617905	C46 H77 O10 N1 P1
PS(22:0_18:2)	PS	M+H	844.6062135	15.93657804	C46 H87 O10 N1 P1
PS(22:4_18:2)	PS	M-H	834.5290605	14.44968284	C46 H77 O10 N1 P1
PS(22:5_18:2)	PS	M-H	832.5134105	13.09805822	C46 H75 O10 N1 P1
PS(34:0)	PS	M+Na	786.5255585	12.02680985	C40 H78 O10 N1 P1 Na1
PS(34:3)	PS	M-H	756.4821105	13.05	C40 H71 O10 N1 P1
PS(35:1)	PS	M-H	774.5290605	13.75814012	C41 H77 O10 N1 P1
PS(36:1)	PS	M+Na	812.5412085	13.09959587	C42 H80 O10 N1 P1 Na1

PS(36:2)	PS	M+H	788.5436135	13.54939427	C42 H79 O10 N1 P1
PS(36:2e)	PS	M-H	772.5497955	12.61618764	C42 H79 O9 N1 P1
PS(36:3)	PS	M-H	784.5134105	14.46939048	C42 H75 O10 N1 P1
PS(36:4)	PS	M+H	784.5123135	11.03655946	C42 H75 O10 N1 P1
PS(36:4e)	PS	M-H	768.5184955	15.15562663	C42 H75 O9 N1 P1
PS(36:5e)	PS	M-H	766.5028455	13.99868728	C42 H73 O9 N1 P1
PS(37:3)	PS	M-H	798.5290605	15.14359952	C43 H77 O10 N1 P1
PS(38:2)	PS	M+Na	838.5568585	13.62784467	C44 H82 O10 N1 P1 Na1
PS(38:3)	PS	M+Na	836.5412085	11.99357559	C44 H80 O10 N1 P1 Na1
PS(38:4)	PS	M+H	812.5436135	13.09988846	C44 H79 O10 N1 P1
PS(38:4e)	PS	M-H	796.5497955	16.44880986	C44 H79 O9 N1 P1
PS(38:5)	PS	M+H	810.5279635	11.92127613	C44 H77 O10 N1 P1
PS(38:5e)	PS	M-H	794.5341455	15.19798862	C44 H77 O9 N1 P1
PS(38:6e)	PS	M-H	792.5184955	14.31813243	C44 H75 O9 N1 P1
PS(38:7e)	PS	M-H	790.5028455	13.70291835	C44 H73 O9 N1 P1
PS(39:1)	PS	M+H	832.6062135	16.33797015	C45 H87 O10 N1 P1
PS(40:3)	PS	M+H	842.5905635	15.029	C46 H85 O10 N1 P1
PS(40:5)	PS	M+H	838.5592635	13.096	C46 H81 O10 N1 P1
PS(40:6)	PS	M+H	836.5436135	11.98607375	C46 H79 O10 N1 P1
PS(40:6e)	PS	M-H	820.5497955	15.66	C46 H79 O9 N1 P1
PS(40:7)	PS	M+H	834.5279635	11.53559557	C46 H77 O10 N1 P1
PS(40:7e)	PS	M-H	818.5341455	15.07883524	C46 H77 O9 N1 P1
PS(40:8e)	PS	M-H	816.5184955	13.74454984	C46 H75 O9 N1 P1
SM(d19:0_19:1)	SM	M+Na	781.6193975	15.53899762	C43 H87 O6 N2 P1 Na1
SM(d19:1_23:0)	SM	M+Na	837.6819975	17.71769797	C47 H95 O6 N2 P1 Na1

SM(d28:1)	SM	M+HCOO	663.4718795	7.843357546	C34 H68 O8 N2 P1
SM(d30:0)	SM	M+HCOO	693.5188295	10.006	C36 H74 O8 N2 P1
SM(d30:1)	SM	M+HCOO	691.5031795	9.43649806	C36 H72 O8 N2 P1
SM(d31:1)	SM	M+HCOO	705.5188295	10.24893363	C37 H74 O8 N2 P1
SM(d32:0)	SM	M+Na	699.5411475	11.63692337	C37 H77 O6 N2 P1 Na1
SM(d32:1)	SM	M+HCOO	719.5344795	11.06357303	C38 H76 O8 N2 P1
SM(d33:1)	SM	M+HCOO	733.5501295	11.85953967	C39 H78 O8 N2 P1
SM(d34:0)	SM	M+Na	727.5724475	13.22995387	C39 H81 O6 N2 P1 Na1
SM(d34:1)	SM	M+Na	725.5567975	12.67122124	C39 H79 O6 N2 P1 Na1
SM(d34:2)	SM	M+Na	723.5411475	11.23590074	C39 H77 O6 N2 P1 Na1
SM(d34:4)	SM	M+H	697.5279025	11.05885355	C39 H74 O6 N2 P1
SM(d35:0)	SM	M+HCOO	763.5970795	13.989	C41 H84 O8 N2 P1
SM(d35:1)	SM	M+HCOO	761.5814295	13.42458871	C41 H82 O8 N2 P1
SM(d35:4)	SM	M+H	711.5435525	11.615	C40 H76 O6 N2 P1
SM(d36:0)	SM	M+HCOO	777.6127295	14.71442847	C42 H86 O8 N2 P1
SM(d36:1)	SM	M+HCOO	775.5970795	14.1815447	C42 H84 O8 N2 P1
SM(d36:2)	SM	M+Na	751.5724475	12.77716234	C41 H81 O6 N2 P1 Na1
SM(d36:3)	SM	M+H	727.5748525	13.215	C41 H80 O6 N2 P1
SM(d36:4)	SM	M+H	725.5592025	12.63	C41 H78 O6 N2 P1
SM(d37:1)	SM	M+H	745.6218025	14.86	C42 H86 O6 N2 P1
SM(d37:4)	SM	M+H	739.5748525	13.431	C42 H80 O6 N2 P1
SM(d38:0)	SM	M+HCOO	805.6440295	16.01126248	C44 H90 O8 N2 P1
SM(d38:2)	SM	M+Na	779.6037475	14.28563722	C43 H85 O6 N2 P1 Na1
SM(d38:3)	SM	M+H	755.6061525	14.715	C43 H84 O6 N2 P1
SM(d39:0)	SM	M+HCOO	819.6596795	16.66141087	C45 H92 O8 N2 P1

SM(d39:1)	SM	M+HCOO	817.6440295	16.17210616	C45 H90 O8 N2 P1
SM(d39:2)	SM	M+H	771.6374525	14.75125573	C44 H88 O6 N2 P1
SM(d40:0)	SM	M+HCOO	833.6753295	17.21208272	C46 H94 O8 N2 P1
SM(d40:1)	SM	M+HCOO	831.6596795	16.80614089	C46 H92 O8 N2 P1
SM(d40:2)	SM	M+Na	807.6350475	15.42147751	C45 H89 O6 N2 P1 Na1
SM(d40:3)	SM	M+Na	805.6193975	14.21886102	C45 H87 O6 N2 P1 Na1
SM(d40:4)	SM	M+H	781.6218025	15.35535045	C45 H86 O6 N2 P1
SM(d41:0)	SM	M+Na	825.6819975	17.715	C46 H95 O6 N2 P1 Na1
SM(d41:1)	SM	M+HCOO	845.6753295	17.2734654	C47 H94 O8 N2 P1
SM(d41:2)	SM	M+HCOO	843.6596795	16.06648827	C47 H92 O8 N2 P1
SM(d41:3)	SM	M+H	797.6531025	16.656	C46 H90 O6 N2 P1
SM(d42:2)	SM	M+Na	835.6663475	16.4879513	C47 H93 O6 N2 P1 Na1
SM(d42:3)	SM	M+HCOO	855.6596795	15.54906795	C48 H92 O8 N2 P1
SM(d42:5)	SM	M+H	807.6374525	15.43	C47 H88 O6 N2 P1
SM(d43:1)	SM	M+HCOO	873.7066295	18.11930097	C49 H98 O8 N2 P1
SM(d43:2)	SM	M+HCOO	871.6909795	17.12186755	C49 H96 O8 N2 P1
SM(d43:4)	SM	M+H	823.6687525	17.166	C48 H92 O6 N2 P1
SM(d44:1)	SM	M+Na	865.7132975	18.652	C49 H99 O6 N2 P1 Na1
SM(d44:2)	SM	M+HCOO	885.7066295	17.737	C50 H98 O8 N2 P1
SM(t42:0)	SM	M+Na	855.6925625	17.323	C47 H97 O7 N2 P1 Na1
SPH(d21:0)	SPH	M+Na	366.3342505	7.679	C21 H45 O2 N1 Na1
SQDG(31:1)	SQDG	M+HCOO	823.4883055	13.04364544	C41 H75 O14 S1
SiE(20:2)	SiE	M+H	705.6544065	3.432	C49 H85 O2
StE(24:7)	StE	M+NH4	766.6496555	17.101	C53 H84 O2 N1
StE(25:6)	StE	M+NH4	782.6809555	17.62379681	C54 H88 O2 N1

TG(10:0_10:0_11:2)	TG	M+NH4	582.4728155	12.38893819	C34 H64 O6 N1
TG(10:0_10:0_12:1)	TG	M+Na	603.4595115	14.86727088	C35 H64 O6 Na1
TG(10:0_10:0_14:0)	TG	M+NH4	628.5510655	12.49612149	C37 H74 O6 N1
TG(10:0_10:0_20:3)	TG	M+H	689.5714665	20.381	C43 H77 O6
TG(10:0_10:0_21:0)	TG	M+NH4	726.6606155	21.89499138	C44 H88 O6 N1
TG(10:0_10:0_23:0)	TG	M+NH4	754.6919155	26.22666494	C46 H92 O6 N1
TG(10:0_10:0_24:1)	TG	M+NH4	766.6919155	26.67248293	C47 H92 O6 N1
TG(10:0_12:0_12:0)	TG	M+Na	633.5064615	16.5555264	C37 H70 O6 Na1
TG(10:0_12:0_14:0)	TG	M+NH4	656.5823655	23.21264716	C39 H78 O6 N1
TG(10:0_12:2_12:3)	TG	M+H	601.4462665	13.017	C37 H61 O6
TG(10:0_12:3_14:0)	TG	M+H	633.5088665	16.81741614	C39 H69 O6
TG(10:0_12:3_21:1)	TG	M+H	729.6027665	18.86455338	C46 H81 O6
TG(10:0_14:0_18:1)	TG	M+NH4	738.6606155	23.58797827	C45 H88 O6 N1
TG(10:0_17:1_18:2)	TG	M+NH4	776.6762655	19.422	C48 H90 O6 N1
TG(10:0_17:1_20:4)	TG	M+H	783.6497165	19.46362403	C50 H87 O6
TG(10:0_18:1_18:2)	TG	M+NH4	790.6919155	19.23491023	C49 H92 O6 N1
TG(10:0_18:1_18:3)	TG	M+NH4	788.6762655	18.66021266	C49 H90 O6 N1
TG(10:0_18:1_20:4)	TG	M+H	797.6653665	19.70534671	C51 H89 O6
TG(10:0_18:3_18:3)	TG	M+H	767.6184165	18.65030813	C49 H83 O6
TG(10:0_18:3_20:4)	TG	M+H	793.6340665	18.99	C51 H85 O6
TG(10:0_20:5_23:1)	TG	M+NH4	882.7545155	19.96095343	C56 H100 O6 N1
TG(11:0_11:1_23:0)	TG	M+NH4	780.7075655	18.53692065	C48 H94 O6 N1
TG(11:0_15:0_15:0)	TG	M+NH4	726.6606155	24.52282036	C44 H88 O6 N1
TG(11:0_16:0_16:0)	TG	M+NH4	754.6919155	21.79695562	C46 H92 O6 N1
TG(11:0_18:2_18:2)	TG	M+NH4	802.6919155	19.03114084	C50 H92 O6 N1

TG(11:0_6:0_6:0)	TG	M+NH4	474.3789155	9.199856684	C26 H52 O6 N1
TG(11:0_8:0_12:3)	TG	M+H	563.4306165	13.433	C34 H59 O6
TG(12:0_12:0_12:0)	TG	M+NH4	656.5823655	20.92025744	C39 H78 O6 N1
TG(12:0_12:0_12:4)	TG	M+H	631.4932165	16.22199813	C39 H67 O6
TG(12:0_20:4_20:4)	TG	M+H	847.6810165	19.37152912	C55 H91 O6
TG(12:0_22:1_22:1)	TG	M+NH4	932.8640655	25.52	C59 H114 O6 N1
TG(12:0e_22:4_22:4)	TG	M+Na	911.7462965	21.233	C59 H100 O5 Na1
TG(12:0e_6:0_20:5)	TG	M+H	643.5296015	16.70065444	C41 H71 O5
TG(12:0e_6:0_6:0)	TG	M+Na	479.3706965	10.852	C27 H52 O5 Na1
TG(12:1e_10:0_18:3)	TG	M+H	673.5765515	18.855	C43 H77 O5
TG(12:1e_11:1_11:1)	TG	M+H	591.4983015	13.63294339	C37 H67 O5
TG(12:1e_6:0_18:3)	TG	M+H	617.5139515	24.874	C39 H69 O5
TG(14:0_10:1_14:0)	TG	M+NH4	682.5980155	5.042457615	C41 H80 O6 N1
TG(14:0_12:1_14:0)	TG	M+NH4	710.6293155	11.459	C43 H84 O6 N1
TG(14:0_14:0_14:0)	TG	M+NH4	740.6762655	25.323	C45 H90 O6 N1
TG(14:0_14:0_18:2)	TG	M+NH4	792.7075655	25.79984397	C49 H94 O6 N1
TG(14:0_14:0_20:4)	TG	M+H	799.6810165	20.20912682	C51 H91 O6
TG(14:0_14:0_20:5)	TG	M+NH4	814.6919155	19.12711585	C51 H92 O6 N1
TG(14:0_14:0_22:5)	TG	M+NH4	842.7232155	19.71194724	C53 H96 O6 N1
TG(14:0_14:0_23:1)	TG	M+Na	869.7568615	20.538	C54 H102 O6 Na1
TG(14:0_17:1_18:2)	TG	M+NH4	832.7388655	20.3645669	C52 H98 O6 N1
TG(14:0_18:2_18:3)	TG	M+NH4	842.7232155	19.38478678	C53 H96 O6 N1
TG(14:0e_16:0_16:0)	TG	M+Na	787.7149965	21.263	C49 H96 O5 Na1
TG(14:0e_18:0_18:0)	TG	M+Na	843.7775965	21.92530329	C53 H104 O5 Na1
TG(14:0e_18:1_18:1)	TG	M+NH4	834.7909005	21.20257393	C53 H104 O5 N1

TG(15:0_10:0_16:0)	TG	M+NH4	726.6606155	19.4334521	C44 H88 O6 N1
TG(15:0_10:0_20:4)	TG	M+H	757.6340665	19.43758196	C48 H85 O6
TG(15:0_10:2_15:0)	TG	M+NH4	708.6136655	23.167	C43 H82 O6 N1
TG(15:0_12:0_16:0)	TG	M+NH4	754.6919155	20.47913147	C46 H92 O6 N1
TG(15:0_12:0_20:4)	TG	M+H	785.6653665	19.96910386	C50 H89 O6
TG(15:0_12:1_18:1)	TG	M+NH4	778.6919155	19.43276052	C48 H92 O6 N1
TG(15:0_12:3_18:2)	TG	M+H	755.6184165	18.851	C48 H83 O6
TG(15:0_14:0_18:1)	TG	M+NH4	808.7388655	20.40596743	C50 H98 O6 N1
TG(15:0_14:0_18:3)	TG	M+H	787.6810165	20.465	C50 H91 O6
TG(15:0_14:0_20:4)	TG	M+H	813.6966665	20.449	C52 H93 O6
TG(15:0_14:0_20:5)	TG	M+H	811.6810165	19.97934881	C52 H91 O6
TG(15:0_14:1_20:5)	TG	M+H	809.6653665	19.49445058	C52 H89 O6
TG(15:0_14:2_18:1)	TG	M+NH4	804.7075655	19.7771293	C50 H94 O6 N1
TG(15:0_16:0_18:2)	TG	M+Na	839.7099115	20.43770574	C52 H96 O6 Na1
TG(15:0_17:0_17:0)	TG	M+NH4	838.7858155	23.93515044	C52 H104 O6 N1
TG(15:0_17:0_17:1)	TG	M+NH4	836.7701655	26.66560774	C52 H102 O6 N1
TG(15:0_17:0_18:3)	TG	M+H	829.7279665	21.09624118	C53 H97 O6
TG(15:0_18:1_18:2)	TG	M+NH4	860.7701655	20.48157514	C54 H102 O6 N1
TG(15:0_18:1_20:5)	TG	M+H	865.7279665	20.48226689	C56 H97 O6
TG(15:0_18:1_22:6)	TG	M+H	891.7436165	20.47293091	C58 H99 O6
TG(15:0_18:2_18:2)	TG	M+NH4	858.7545155	20.0947003	C54 H100 O6 N1
TG(15:0_18:2_18:3)	TG	M+Na	861.6942615	19.68408517	C54 H94 O6 Na1
TG(15:0_18:2_20:5)	TG	M+H	863.7123165	20.10221998	C56 H95 O6
TG(15:0_18:3_18:3)	TG	M+H	837.6966665	20.02223686	C54 H93 O6
TG(15:0_19:0_19:0)	TG	M+NH4	894.8484155	24.827	C56 H112 O6 N1

TG(15:0_6:0_14:3)	TG	M+H	619.4932165	15.9500343	C38 H67 O6
TG(15:0_6:0_15:0)	TG	M+NH4	656.5823655	12.012	C39 H78 O6 N1
TG(15:0_6:0_18:1)	TG	M+NH4	696.6136655	18.16867069	C42 H82 O6 N1
TG(15:0_6:0_18:2)	TG	M+Na	699.5534115	17.39342273	C42 H76 O6 Na1
TG(15:0_6:0_18:3)	TG	M+H	675.5558165	18.31344201	C42 H75 O6
TG(15:0_6:0_20:3)	TG	M+H	703.5871165	19.05104279	C44 H79 O6
TG(15:0_6:0_20:5)	TG	M+H	699.5558165	17.41012714	C44 H75 O6
TG(15:0_6:0_6:0)	TG	M+NH4	530.4415155	11.73757545	C30 H60 O6 N1
TG(15:0_6:0_8:0)	TG	M+NH4	558.4728155	13.30584171	C32 H64 O6 N1
TG(15:0_8:0_16:0)	TG	M+NH4	698.6293155	19.07180168	C42 H84 O6 N1
TG(15:0_8:0_20:3)	TG	M+NH4	748.6449655	18.27099512	C46 H86 O6 N1
TG(15:0_8:0_22:5)	TG	M+H	755.6184165	19.455	C48 H83 O6
TG(15:0_8:0_8:0)	TG	M+NH4	586.5041155	10.904	C34 H68 O6 N1
TG(16:0_10:0_10:0)	TG	M+NH4	656.5823655	25.20067577	C39 H78 O6 N1
TG(16:0_10:0_17:0)	TG	M+Na	759.6473115	19.89672078	C46 H88 O6 Na1
TG(16:0_10:0_20:3)	TG	M+H	773.6653665	25.79	C49 H89 O6
TG(16:0_10:0_22:5)	TG	M+NH4	814.6919155	18.81049851	C51 H92 O6 N1
TG(16:0_10:0_22:6)	TG	M+NH4	812.6762655	18.69123359	C51 H90 O6 N1
TG(16:0_10:0_23:1)	TG	M+NH4	836.7701655	19.748	C52 H102 O6 N1
TG(16:0_10:1_16:0)	TG	M+NH4	738.6606155	24.58469417	C45 H88 O6 N1
TG(16:0_12:0_14:0)	TG	M+NH4	740.6762655	22.808	C45 H90 O6 N1
TG(16:0_12:0_16:1)	TG	M+Na	771.6473115	19.231	C47 H88 O6 Na1
TG(16:0_12:0_22:6)	TG	M+NH4	840.7075655	19.33122812	C53 H94 O6 N1
TG(16:0_12:3_14:0)	TG	M+H	717.6027665	19.15162451	C45 H81 O6
TG(16:0_12:3_16:0)	TG	M+H	745.6340665	19.74105493	C47 H85 O6

TG(16:0_13:0_14:0)	TG	M+NH4	754.6919155	22.36195816	C46 H92 O6 N1
TG(16:0_14:0_14:0)	TG	M+NH4	768.7075655	20.24235779	C47 H94 O6 N1
TG(16:0_14:0_17:0)	TG	M+NH4	810.7545155	20.80810811	C50 H100 O6 N1
TG(16:0_14:0_17:1)	TG	M+NH4	808.7388655	20.84183652	C50 H98 O6 N1
TG(16:0_14:0_18:2)	TG	M+NH4	820.7388655	24.99420426	C51 H98 O6 N1
TG(16:0_14:0_18:3)	TG	M+H	801.6966665	21.16	C51 H93 O6
TG(16:0_14:0_20:4)	TG	M+H	827.7123165	20.65493418	C53 H95 O6
TG(16:0_14:0_22:6)	TG	M+NH4	868.7388655	19.87803701	C55 H98 O6 N1
TG(16:0_14:1_18:1)	TG	M+NH4	820.7388655	20.8552233	C51 H98 O6 N1
TG(16:0_14:1_19:0)	TG	M+NH4	836.7701655	23.296	C52 H102 O6 N1
TG(16:0_16:0_20:1)	TG	M+NH4	878.8171155	24.73574252	C55 H108 O6 N1
TG(16:0_16:0_20:3)	TG	M+H	857.7592665	21.46242716	C55 H101 O6
TG(16:0_16:0_22:5)	TG	M+H	881.7592665	26.30301808	C57 H101 O6
TG(16:0_16:0_23:0)	TG	M+NH4	922.8797155	22.18863176	C58 H116 O6 N1
TG(16:0_16:0_23:1)	TG	M+NH4	920.8640655	21.88849906	C58 H114 O6 N1
TG(16:0_16:0_24:0)	TG	M+NH4	936.8953655	22.32037713	C59 H118 O6 N1
TG(16:0_16:1_16:1)	TG	M+NH4	820.7388655	27.227	C51 H98 O6 N1
TG(16:0_16:1_17:0)	TG	M+Na	841.7255615	20.511	C52 H98 O6 Na1
TG(16:0_16:1_18:2)	TG	M+Na	851.7099115	26.6740086	C53 H96 O6 Na1
TG(16:0_16:1_18:3)	TG	M+NH4	844.7388655	27.218	C53 H98 O6 N1
TG(16:0_16:1_20:2)	TG	M+NH4	874.7858155	27.32556656	C55 H104 O6 N1
TG(16:0_16:1_20:4)	TG	M+H	853.7279665	27.2212257	C55 H97 O6
TG(16:0_16:1_20:5)	TG	M+H	851.7123165	26.68314087	C55 H95 O6
TG(16:0_17:0_18:3)	TG	M+H	843.7436165	21.202	C54 H99 O6
TG(16:0_17:0_20:4)	TG	M+H	869.7592665	21.264	C56 H101 O6

TG(16:0_17:1_18:1)	TG	M+Na	867.7412115	20.85213996	C54 H100 O6 Na1
TG(16:0_17:1_18:2)	TG	M+NH4	860.7701655	20.85266423	C54 H102 O6 N1
TG(16:0_17:1_20:5)	TG	M+NH4	882.7545155	21.0159017	C56 H100 O6 N1
TG(16:0_18:1_20:1)	TG	M+NH4	904.8327655	20.183	C57 H110 O6 N1
TG(16:0_18:1_22:6)	TG	M+H	905.7592665	27.397	C59 H101 O6
TG(16:0_18:1_24:0)	TG	M+Na	967.8664115	22.29582096	C61 H116 O6 Na1
TG(16:0_18:2_18:3)	TG	M+NH4	870.7545155	19.93291638	C55 H100 O6 N1
TG(16:0_18:2_20:4)	TG	M+H	879.7436165	20.63698134	C57 H99 O6
TG(16:0_18:3_23:0)	TG	M+H	927.8375165	22.18623045	C60 H111 O6
TG(16:0_18:3_23:1)	TG	M+H	925.8218665	21.88921378	C60 H109 O6
TG(16:0_19:0_20:3)	TG	M+H	899.8062165	21.91490268	C58 H107 O6
TG(16:0_20:2_20:2)	TG	M+Na	933.7881615	26.72776172	C59 H106 O6 Na1
TG(16:0_20:2_20:4)	TG	M+H	907.7749165	21.286	C59 H103 O6
TG(16:0_20:4_20:4)	TG	M+H	903.7436165	20.53800829	C59 H99 O6
TG(16:0_20:4_22:5)	TG	M+NH4	946.7858155	19.93727096	C61 H104 O6 N1
TG(16:0_20:4_23:0)	TG	M+H	953.8531665	22.16241496	C62 H113 O6
TG(16:0_22:4_22:4)	TG	M+NH4	976.8327655	20.48297162	C63 H110 O6 N1
TG(16:0_22:5_24:0)	TG	M+NH4	1010.911015	21.90179111	C65 H120 O6 N1
TG(16:0_6:0_10:0)	TG	M+NH4	600.5197655	15.53061153	C35 H70 O6 N1
TG(16:0_6:0_12:3)	TG	M+H	605.4775665	15.756	C37 H65 O6
TG(16:0_6:0_17:0)	TG	M+NH4	698.6293155	19.48223822	C42 H84 O6 N1
TG(16:0_6:0_18:2)	TG	M+NH4	708.6136655	19.05754221	C43 H82 O6 N1
TG(16:0_6:0_22:4)	TG	M+NH4	760.6449655	18.37075956	C47 H86 O6 N1
TG(16:0_6:0_22:5)	TG	M+NH4	758.6293155	17.92380334	C47 H84 O6 N1
TG(16:0_6:0_22:6)	TG	M+Na	761.5690615	17.14401809	C47 H78 O6 Na1

TG(16:0_6:0_6:0)	TG	M+NH4	544.4571655	15.47870302	C31 H62 O6 N1
TG(16:0_8:0_10:0)	TG	M+NH4	628.5510655	23.78157187	C37 H74 O6 N1
TG(16:0_8:0_11:2)	TG	M+H	621.5088665	14.5245478	C38 H69 O6
TG(16:0_8:0_14:0)	TG	M+NH4	684.6136655	18.53792754	C41 H82 O6 N1
TG(16:0_8:0_16:1)	TG	M+NH4	710.6293155	18.69511109	C43 H84 O6 N1
TG(16:0_8:0_17:0)	TG	M+NH4	726.6606155	24.12667741	C44 H88 O6 N1
TG(16:0_8:0_20:4)	TG	M+H	743.6184165	26.65528663	C47 H83 O6
TG(16:0_8:0_22:5)	TG	M+NH4	786.6606155	18.0634224	C49 H88 O6 N1
TG(16:0e_10:1_10:1)	TG	M+NH4	638.5718005	26.64812978	C39 H76 O5 N1
TG(16:0e_16:0_20:4)	TG	M+H	841.7643515	21.57708602	C55 H101 O5
TG(16:0e_6:0_20:4)	TG	M+H	701.6078515	19.52	C45 H81 O5
TG(16:1_10:0_10:3)	TG	M+H	631.4932165	15.32485251	C39 H67 O6
TG(16:1_10:0_20:4)	TG	M+H	769.6340665	19.17432238	C49 H85 O6
TG(16:1_12:0_12:3)	TG	M+H	687.5558165	17.69173844	C43 H75 O6
TG(16:1_14:1_18:1)	TG	M+NH4	818.7232155	19.79962861	C51 H96 O6 N1
TG(16:1_17:0_18:3)	TG	M+H	841.7279665	20.788	C54 H97 O6
TG(16:1_17:0_20:4)	TG	M+H	867.7436165	20.84392937	C56 H99 O6
TG(16:1_17:1_18:2)	TG	M+NH4	858.7545155	20.34541081	C54 H100 O6 N1
TG(16:1_18:1_18:1)	TG	M+NH4	874.7858155	20.70087445	C55 H104 O6 N1
TG(16:1_18:1_18:3)	TG	M+H	853.7279665	26.68072686	C55 H97 O6
TG(16:1_18:1_19:0)	TG	M+NH4	890.8171155	24.537	C56 H108 O6 N1
TG(16:1_18:2_18:2)	TG	M+NH4	870.7545155	21.271	C55 H100 O6 N1
TG(16:1_18:2_18:3)	TG	M+NH4	868.7388655	19.60516201	C55 H98 O6 N1
TG(16:1_18:3_18:3)	TG	M+H	849.6966665	19.86052945	C55 H93 O6
TG(16:1_19:0_19:0)	TG	M+NH4	906.8484155	23.40848528	C57 H112 O6 N1

TG(16:1_20:1_20:1)	TG	M+NH4	930.8484155	26.082	C59 H112 O6 N1
TG(16:1_20:4_23:0)	TG	M+H	951.8375165	21.857	C62 H111 O6
TG(16:1_20:5_21:1)	TG	M+NH4	936.8014655	20.40069128	C60 H106 O6 N1
TG(16:1_6:0_12:0)	TG	M+NH4	626.5354155	24.46496583	C37 H72 O6 N1
TG(16:1_6:0_14:0)	TG	M+NH4	654.5667155	26.64992014	C39 H76 O6 N1
TG(16:1_6:0_14:4)	TG	M+H	629.4775665	15.484	C39 H65 O6
TG(16:1_6:0_16:1)	TG	M+NH4	680.5823655	18.274	C41 H78 O6 N1
TG(16:1_6:0_18:1)	TG	M+NH4	708.6136655	24.72217027	C43 H82 O6 N1
TG(16:1_6:0_18:2)	TG	M+NH4	706.5980155	17.03093439	C43 H80 O6 N1
TG(16:1_6:0_20:5)	TG	M+H	711.5558165	17.02913237	C45 H75 O6
TG(16:1_6:0_6:0)	TG	M+Na	547.3969115	11.49192099	C31 H56 O6 Na1
TG(16:1_8:0_10:0)	TG	M+Na	631.4908115	15.41437103	C37 H68 O6 Na1
TG(16:1_8:0_16:1)	TG	M+NH4	708.6136655	17.019	C43 H82 O6 N1
TG(16:1e_16:0_16:0)	TG	M+NH4	808.7752505	21.22112627	C51 H102 O5 N1
TG(17:0_10:0_14:0)	TG	M+NH4	726.6606155	21.32769433	C44 H88 O6 N1
TG(17:0_10:0_14:3)	TG	M+H	703.5871165	18.79509661	C44 H79 O6
TG(17:0_12:0_14:0)	TG	M+NH4	754.6919155	20.96766273	C46 H92 O6 N1
TG(17:0_12:0_14:3)	TG	M+H	731.6184165	19.33765541	C46 H83 O6
TG(17:0_13:0_17:0)	TG	M+NH4	810.7545155	19.45334009	C50 H100 O6 N1
TG(17:0_14:0_14:0)	TG	M+NH4	782.7232155	22.42626311	C48 H96 O6 N1
TG(17:0_14:0_18:1)	TG	M+NH4	836.7701655	23.582	C52 H102 O6 N1
TG(17:0_14:0_18:3)	TG	M+H	815.7123165	20.82	C52 H95 O6
TG(17:0_17:0_17:1)	TG	M+NH4	864.8014655	23.96879359	C54 H106 O6 N1
TG(17:0_17:0_18:1)	TG	M+NH4	878.8171155	25.64225143	C55 H108 O6 N1
TG(17:0_17:0_19:0)	TG	M+NH4	894.8484155	24.38248231	C56 H112 O6 N1

TG(17:0_17:1_17:1)	TG	M+NH4	862.7858155	26.679	C54 H104 O6 N1
TG(17:0_17:1_19:0)	TG	M+NH4	892.8327655	26.11042074	C56 H110 O6 N1
TG(17:0_18:1_19:0)	TG	M+NH4	906.8484155	24.95371306	C57 H112 O6 N1
TG(17:0_18:1_20:3)	TG	M+H	897.7905665	21.58438161	C58 H105 O6
TG(17:0_18:1_20:5)	TG	M+NH4	910.7858155	20.30093718	C58 H104 O6 N1
TG(17:0_18:1_22:5)	TG	M+NH4	938.8171155	20.60433994	C60 H108 O6 N1
TG(17:0_6:0_14:3)	TG	M+H	647.5245165	16.953	C40 H71 O6
TG(17:0_6:0_17:0)	TG	M+NH4	712.6449655	16.83119915	C43 H86 O6 N1
TG(17:0_6:0_18:3)	TG	M+Na	725.5690615	17.46593198	C44 H78 O6 Na1
TG(17:0_6:0_22:5)	TG	M+NH4	772.6449655	17.80478115	C48 H86 O6 N1
TG(17:0_8:0_18:1)	TG	M+NH4	752.6762655	19.43812351	C46 H90 O6 N1
TG(18:0_10:0_10:0)	TG	M+NH4	684.6136655	15.529	C41 H82 O6 N1
TG(18:0_10:0_14:0)	TG	M+NH4	740.6762655	23.19128834	C45 H90 O6 N1
TG(18:0_10:0_16:0)	TG	M+NH4	768.7075655	24.34136047	C47 H94 O6 N1
TG(18:0_10:0_17:1)	TG	M+NH4	780.7075655	20.392	C48 H94 O6 N1
TG(18:0_10:0_18:0)	TG	M+NH4	796.7388655	19.30414149	C49 H98 O6 N1
TG(18:0_10:1_14:0)	TG	M+NH4	738.6606155	18.516	C45 H88 O6 N1
TG(18:0_10:1_18:0)	TG	M+NH4	794.7232155	18.775	C49 H96 O6 N1
TG(18:0_10:2_18:0)	TG	M+NH4	792.7075655	18.670314	C49 H94 O6 N1
TG(18:0_12:0_16:0)	TG	M+NH4	796.7388655	26.19854368	C49 H98 O6 N1
TG(18:0_12:0_18:1)	TG	M+NH4	822.7545155	19.18729721	C51 H100 O6 N1
TG(18:0_12:2_18:0)	TG	M+NH4	820.7388655	19.77949236	C51 H98 O6 N1
TG(18:0_13:0_21:0)	TG	M+Na	885.7881615	19.51906431	C55 H106 O6 Na1
TG(18:0_14:0_14:2)	TG	M+NH4	792.7075655	19.21415485	C49 H94 O6 N1
TG(18:0_14:0_16:1)	TG	M+Na	827.7099115	26.33725014	C51 H96 O6 Na1

TG(18:0_14:0_17:0)	TG	M+NH4	838.7858155	23.7312057	C52 H104 O6 N1
TG(18:0_14:0_18:2)	TG	M+NH4	848.7701655	22.49733566	C53 H102 O6 N1
TG(18:0_14:0_20:0)	TG	M+NH4	880.8327655	23.275	C55 H110 O6 N1
TG(18:0_14:0_20:2)	TG	M+NH4	876.8014655	20.68	C55 H106 O6 N1
TG(18:0_14:0_20:5)	TG	M+NH4	870.7545155	20.20721311	C55 H100 O6 N1
TG(18:0_14:0_23:0)	TG	M+NH4	922.8797155	23.336	C58 H116 O6 N1
TG(18:0_14:1_18:0)	TG	M+NH4	850.7858155	19.74420047	C53 H104 O6 N1
TG(18:0_14:3_16:0)	TG	M+NH4	818.7232155	20.32857276	C51 H96 O6 N1
TG(18:0_15:0_18:1)	TG	M+NH4	864.8014655	22.67952058	C54 H106 O6 N1
TG(18:0_16:0_16:1)	TG	M+NH4	850.7858155	21.54498298	C53 H104 O6 N1
TG(18:0_16:0_17:0)	TG	M+Na	871.7725115	21.59640988	C54 H104 O6 Na1
TG(18:0_16:0_18:3)	TG	M+H	857.7592665	22.885	C55 H101 O6
TG(18:0_16:0_19:0)	TG	M+NH4	894.8484155	22.16995596	C56 H112 O6 N1
TG(18:0_16:0_20:2)	TG	M+NH4	904.8327655	15.722	C57 H110 O6 N1
TG(18:0_16:0_21:0)	TG	M+NH4	922.8797155	23.038	C58 H116 O6 N1
TG(18:0_16:0_23:0)	TG	M+NH4	950.9110155	22.44085443	C60 H120 O6 N1
TG(18:0_16:0_23:1)	TG	M+NH4	948.8953655	22.1642638	C60 H118 O6 N1
TG(18:0_16:0_24:0)	TG	M+NH4	964.9266655	22.56362098	C61 H122 O6 N1
TG(18:0_16:1_19:0)	TG	M+NH4	892.8327655	25.79	C56 H110 O6 N1
TG(18:0_16:1_20:1)	TG	M+NH4	904.8327655	26.46356677	C57 H110 O6 N1
TG(18:0_16:1_20:3)	TG	M+H	883.7749165	22.15486718	C57 H103 O6
TG(18:0_17:1_18:0)	TG	M+NH4	892.8327655	21.89975154	C56 H110 O6 N1
TG(18:0_17:1_18:1)	TG	M+NH4	890.8171155	21.56318457	C56 H108 O6 N1
TG(18:0_18:0_18:3)	TG	M+NH4	902.8171155	12.057	C57 H108 O6 N1
TG(18:0_18:0_20:0)	TG	M+NH4	936.8953655	22.88884404	C59 H118 O6 N1

TG(18:0_18:0_20:1)	TG	M+NH4	934.8797155	21.739	C59 H116 O6 N1
TG(18:0_18:0_22:0)	TG	M+NH4	964.9266655	22.82233	C61 H122 O6 N1
TG(18:0_18:0_22:4)	TG	M+NH4	956.8640655	21.64759397	C61 H114 O6 N1
TG(18:0_18:0_22:6)	TG	M+NH4	952.8327655	20.85775562	C61 H110 O6 N1
TG(18:0_18:0_23:1)	TG	M+NH4	976.9266655	22.4182848	C62 H122 O6 N1
TG(18:0_18:0_24:0)	TG	M+NH4	992.9579655	22.78719764	C63 H126 O6 N1
TG(18:0_18:1_18:1)	TG	M+NH4	904.8327655	21.39191362	C57 H110 O6 N1
TG(18:0_18:1_18:2)	TG	M+NH4	902.8171155	25.07108501	C57 H108 O6 N1
TG(18:0_18:1_18:3)	TG	M+NH4	900.8014655	23.18448142	C57 H106 O6 N1
TG(18:0_18:1_20:0)	TG	M+Na	939.8351115	22.02954241	C59 H112 O6 Na1
TG(18:0_18:1_20:3)	TG	M+NH4	928.8327655	21.13721018	C59 H110 O6 N1
TG(18:0_18:1_20:4)	TG	M+H	909.7905665	21.37606785	C59 H105 O6
TG(18:0_18:1_20:5)	TG	M+H	907.7749165	25.91242846	C59 H103 O6
TG(18:0_18:1_22:4)	TG	M+NH4	954.8484155	21.19545147	C61 H112 O6 N1
TG(18:0_18:1_22:6)	TG	M+NH4	950.8171155	20.76928976	C61 H108 O6 N1
TG(18:0_18:1_24:0)	TG	M+NH4	990.9423155	22.5411028	C63 H124 O6 N1
TG(18:0_18:2_18:3)	TG	M+H	881.7592665	13.03460528	C57 H101 O6
TG(18:0_18:2_20:5)	TG	M+H	905.7592665	20.75196992	C59 H101 O6
TG(18:0_18:2_23:1)	TG	M+NH4	972.8953655	21.85314211	C62 H118 O6 N1
TG(18:0_18:3_23:0)	TG	M+H	955.8688165	22.441	C62 H115 O6
TG(18:0_18:3_24:0)	TG	M+H	969.8844665	22.5612132	C63 H117 O6
TG(18:0_20:0_22:5)	TG	M+NH4	982.8797155	21.56982335	C63 H116 O6 N1
TG(18:0_20:1_22:5)	TG	M+NH4	980.8640655	21.2345603	C63 H114 O6 N1
TG(18:0_20:3_20:5)	TG	M+H	931.7749165	20.93227162	C61 H103 O6
TG(18:0_20:3_23:0)	TG	M+H	983.9001165	22.673	C64 H119 O6

TG(18:0_20:4_24:0)	TG	M+H	995.9001165	22.534	C65 H119 O6
TG(18:0_23:1_24:0)	TG	M+NH4	1061.020565	23.06717239	C68 H134 O6 N1
TG(18:0_6:0_15:0)	TG	M+NH4	698.6293155	20.59129958	C42 H84 O6 N1
TG(18:0_6:0_16:1)	TG	M+NH4	710.6293155	15.59315693	C43 H84 O6 N1
TG(18:0_6:0_18:1)	TG	M+NH4	738.6606155	25.20649844	C45 H88 O6 N1
TG(18:0_6:0_18:3)	TG	M+NH4	734.6293155	18.15359787	C45 H84 O6 N1
TG(18:0_6:0_20:4)	TG	M+H	743.6184165	19.18370544	C47 H83 O6
TG(18:0_6:0_22:5)	TG	M+Na	791.6160115	18.35326628	C49 H84 O6 Na1
TG(18:0_6:0_22:6)	TG	M+NH4	784.6449655	17.95220013	C49 H86 O6 N1
TG(18:0_6:0_23:1)	TG	M+NH4	808.7388655	19.21899195	C50 H98 O6 N1
TG(18:0_6:0_8:0)	TG	M+NH4	600.5197655	18.78141723	C35 H70 O6 N1
TG(18:0_8:0_10:0)	TG	M+NH4	656.5823655	12.67452801	C39 H78 O6 N1
TG(18:0_8:0_12:3)	TG	M+H	661.5401665	17.58919149	C41 H73 O6
TG(18:0_8:0_12:4)	TG	M+H	659.5245165	17.39747518	C41 H71 O6
TG(18:0_8:0_18:0)	TG	M+NH4	768.7075655	18.5940974	C47 H94 O6 N1
TG(18:0_8:0_18:1)	TG	M+NH4	766.6919155	19.72650661	C47 H92 O6 N1
TG(18:0_8:0_20:1)	TG	M+NH4	794.7232155	19.306	C49 H96 O6 N1
TG(18:0_8:0_20:4)	TG	M+H	771.6497165	19.70319938	C49 H87 O6
TG(18:0e_18:2_18:2)	TG	M+H	869.7956515	19.2	C57 H105 O5
TG(18:0e_6:0_10:1)	TG	M+NH4	612.5561505	16.37869404	C37 H74 O5 N1
TG(18:1_10:4_20:4)	TG	M+H	789.6027665	17.51309182	C51 H81 O6
TG(18:1_12:0_17:1)	TG	M+NH4	806.7232155	19.98444786	C50 H96 O6 N1
TG(18:1_12:0_18:3)	TG	M+NH4	816.7075655	19.3161047	C51 H94 O6 N1
TG(18:1_12:1_18:2)	TG	M+NH4	816.7075655	19.58629616	C51 H94 O6 N1
TG(18:1_12:3_20:4)	TG	M+H	819.6497165	18.93725498	C53 H87 O6

TG(18:1_13:0_18:2)	TG	M+NH4	832.7388655	20.02352885	C52 H98 O6 N1
TG(18:1_14:0_14:2)	TG	M+NH4	790.6919155	19.61817966	C49 H92 O6 N1
TG(18:1_14:0_17:1)	TG	M+NH4	834.7545155	20.82088622	C52 H100 O6 N1
TG(18:1_14:1_18:1)	TG	M+NH4	846.7545155	23.229	C53 H100 O6 N1
TG(18:1_14:3_18:1)	TG	M+H	825.6966665	20.18297414	C53 H93 O6
TG(18:1_14:4_18:1)	TG	M+NH4	840.7075655	19.00754232	C53 H94 O6 N1
TG(18:1_17:1_18:1)	TG	M+Na	893.7568615	20.8463042	C56 H102 O6 Na1
TG(18:1_17:1_18:2)	TG	M+NH4	886.7858155	21.8	C56 H104 O6 N1
TG(18:1_17:1_20:4)	TG	M+H	893.7592665	20.81540655	C58 H101 O6
TG(18:1_17:1_20:5)	TG	M+NH4	908.7701655	19.94139954	C58 H102 O6 N1
TG(18:1_18:1_18:3)	TG	M+H	881.7592665	22.952	C57 H101 O6
TG(18:1_18:1_20:3)	TG	M+NH4	926.8171155	20.91121182	C59 H108 O6 N1
TG(18:1_18:1_20:5)	TG	M+Na	927.7412115	20.12100418	C59 H100 O6 Na1
TG(18:1_18:1_21:0)	TG	M+NH4	946.8797155	21.86459455	C60 H116 O6 N1
TG(18:1_18:1_22:0)	TG	M+NH4	960.8953655	22.00277323	C61 H118 O6 N1
TG(18:1_18:1_22:2)	TG	M+NH4	956.8640655	21.34548847	C61 H114 O6 N1
TG(18:1_18:1_22:4)	TG	M+H	935.8062165	21.339	C61 H107 O6
TG(18:1_18:1_22:5)	TG	M+NH4	950.8171155	20.49144842	C61 H108 O6 N1
TG(18:1_18:1_22:6)	TG	M+NH4	948.8014655	20.21374877	C61 H106 O6 N1
TG(18:1_18:1_23:0)	TG	M+NH4	974.9110155	22.13620339	C62 H120 O6 N1
TG(18:1_18:1_24:0)	TG	M+NH4	988.9266655	22.27122558	C63 H122 O6 N1
TG(18:1_18:1_24:1)	TG	M+NH4	986.9110155	21.97223346	C63 H120 O6 N1
TG(18:1_18:2_18:3)	TG	M+Na	901.7255615	19.89424719	C57 H98 O6 Na1
TG(18:1_18:2_20:4)	TG	M+H	905.7592665	26.67627861	C59 H101 O6
TG(18:1_18:2_21:0)	TG	M+NH4	944.8640655	21.56215452	C60 H114 O6 N1

TG(18:1_18:2_21:1)	TG	M+NH4	942.8484155	21.30946253	C60 H112 O6 N1
TG(18:1_18:2_23:1)	TG	M+NH4	970.8797155	21.57199471	C62 H116 O6 N1
TG(18:1_18:3_18:3)	TG	M+NH4	894.7545155	19.76863976	C57 H100 O6 N1
TG(18:1_18:3_22:5)	TG	M+H	929.7592665	20.51491106	C61 H101 O6
TG(18:1_18:3_23:1)	TG	M+NH4	968.8640655	21.22578336	C62 H114 O6 N1
TG(18:1_18:3_24:1)	TG	M+H	965.8531665	21.99729784	C63 H113 O6
TG(18:1_20:3_20:3)	TG	M+H	933.7905665	21.31	C61 H105 O6
TG(18:1_20:4_22:1)	TG	M+H	963.8375165	21.679	C63 H111 O6
TG(18:1_20:4_23:0)	TG	M+H	979.8688165	22.13023817	C64 H115 O6
TG(18:1e_14:0_14:0)	TG	M+NH4	780.7439505	20.83547335	C49 H98 O5 N1
TG(18:1e_14:2_14:2)	TG	M+H	755.6548015	17.738	C49 H87 O5
TG(18:1e_16:0_16:0)	TG	M+NH4	836.8065505	21.57710157	C53 H106 O5 N1
TG(18:1e_18:1_18:1)	TG	M+NH4	888.8378505	21.52317026	C57 H110 O5 N1
TG(18:2_12:3_18:2)	TG	M+H	793.6340665	18.65878455	C51 H85 O6
TG(18:2_14:1_17:1)	TG	M+NH4	830.7232155	19.67029384	C52 H96 O6 N1
TG(18:2_17:1_17:1)	TG	M+H	855.7436165	14.743	C55 H99 O6
TG(18:2_17:1_18:2)	TG	M+NH4	884.7701655	21.43419678	C56 H102 O6 N1
TG(18:2_18:2_18:2)	TG	M+H	879.7436165	26.67183709	C57 H99 O6
TG(18:2e_6:0_6:0)	TG	M+NH4	554.4779005	13.58094811	C33 H64 O5 N1
TG(18:2e_6:0_8:0)	TG	M+H	565.4826515	13.56894033	C35 H65 O5
TG(18:2e_8:0_10:1)	TG	M+NH4	636.5561505	15.33049649	C39 H74 O5 N1
TG(18:3_11:1_18:2)	TG	M+H	781.6340665	18.94232323	C50 H85 O6
TG(18:3_18:2_18:2)	TG	M+H	877.7279665	20.33732245	C57 H97 O6
TG(18:3_18:2_18:3)	TG	M+H	875.7123165	19.92092392	C57 H95 O6
TG(18:3_18:3_20:2)	TG	M+NH4	920.7701655	19.72498101	C59 H102 O6 N1

TG(18:4_12:0_12:0)	TG	M+H	715.5871165	18.64104172	C45 H79 O6
TG(18:4_14:0_18:2)	TG	M+H	823.6810165	19.83109198	C53 H91 O6
TG(18:4_14:1_18:2)	TG	M+H	821.6653665	19.33782023	C53 H89 O6
TG(18:4_15:0_18:1)	TG	M+H	839.7123165	20.42293812	C54 H95 O6
TG(18:4_16:0_16:0)	TG	M+H	827.7123165	26.70171985	C53 H95 O6
TG(18:4_18:1_18:1)	TG	M+H	879.7436165	27.34976513	C57 H99 O6
TG(18:4_6:0_14:0)	TG	M+H	659.5245165	16.88606022	C41 H71 O6
TG(18:4_6:0_14:1)	TG	M+H	657.5088665	15.76680475	C41 H69 O6
TG(18:4_6:0_15:0)	TG	M+H	673.5401665	17.92255667	C42 H73 O6
TG(18:4_6:0_17:0)	TG	M+H	701.5714665	18.16888844	C44 H77 O6
TG(18:4_6:0_18:3)	TG	M+H	709.5401665	15.996	C45 H73 O6
TG(18:4_6:0_18:4)	TG	M+H	707.5245165	15.38159156	C45 H71 O6
TG(18:4_6:0_24:0)	TG	M+H	799.6810165	20.51780252	C51 H91 O6
TG(18:4_6:0_6:0)	TG	M+NH4	564.4258655	9.853142888	C33 H58 O6 N1
TG(18:4_6:0_9:0)	TG	M+H	589.4462665	13.509	C36 H61 O6
TG(18:4_8:0_14:0)	TG	M+H	687.5558165	18.367	C43 H75 O6
TG(18:4_8:0_19:0)	TG	M+H	757.6340665	19.914	C48 H85 O6
TG(19:0_14:0_18:1)	TG	M+NH4	864.8014655	25.35902087	C54 H106 O6 N1
TG(19:0_14:2_19:0)	TG	M+NH4	876.8014655	15.64	C55 H106 O6 N1
TG(19:0_18:1_18:1)	TG	M+NH4	918.8484155	21.56665931	C58 H112 O6 N1
TG(19:0_6:0_6:0)	TG	M+NH4	586.5041155	14.60622031	C34 H68 O6 N1
TG(19:0_9:0_21:0)	TG	M+NH4	838.7858155	20.01068841	C52 H104 O6 N1
TG(19:1_10:1_18:1)	TG	M+NH4	804.7075655	19.49192838	C50 H94 O6 N1
TG(19:1_15:0_17:0)	TG	M+NH4	864.8014655	24.8802225	C54 H106 O6 N1
TG(19:1_16:0_16:0)	TG	M+NH4	864.8014655	21.68859771	C54 H106 O6 N1

TG(19:1_16:0_18:1)	TG	M+NH4	890.8171155	22.01916116	C56 H108 O6 N1
TG(19:1_17:0_17:1)	TG	M+NH4	890.8171155	26.28382219	C56 H108 O6 N1
TG(19:1_17:1_17:1)	TG	M+NH4	888.8014655	21.794	C56 H106 O6 N1
TG(19:1_18:0_18:0)	TG	M+NH4	920.8640655	22.18182703	C58 H114 O6 N1
TG(19:1_18:0_18:1)	TG	M+NH4	918.8484155	21.88381576	C58 H112 O6 N1
TG(19:1_18:0_20:4)	TG	M+H	923.8062165	21.5647208	C60 H107 O6
TG(19:1_18:1_18:1)	TG	M+NH4	916.8327655	21.23782308	C58 H110 O6 N1
TG(19:1_18:1_18:2)	TG	M+NH4	914.8171155	20.93636693	C58 H108 O6 N1
TG(19:1_18:1_20:3)	TG	M+NH4	940.8327655	20.96697999	C60 H110 O6 N1
TG(19:1_18:1_20:4)	TG	M+H	921.7905665	21.28556964	C60 H105 O6
TG(19:1_18:2_18:2)	TG	M+NH4	912.8014655	20.58420164	C58 H106 O6 N1
TG(19:1_6:0_14:3)	TG	M+NH4	690.5667155	15.47652846	C42 H76 O6 N1
TG(19:1_6:0_18:1)	TG	M+NH4	750.6606155	18.8842899	C46 H88 O6 N1
TG(19:1_6:0_20:5)	TG	M+H	753.6027665	18.304	C48 H81 O6
TG(19:1_8:0_14:0)	TG	M+NH4	724.6449655	19.27836796	C44 H86 O6 N1
TG(19:1_8:0_16:0)	TG	M+NH4	752.6762655	19.91589631	C46 H90 O6 N1
TG(19:1_8:0_16:1)	TG	M+NH4	750.6606155	19.36814404	C46 H88 O6 N1
TG(20:0_10:1_20:0)	TG	M+NH4	850.7858155	20.69	C53 H104 O6 N1
TG(20:0_10:3_10:3)	TG	M+H	683.5245165	15.82881208	C43 H71 O6
TG(20:0_13:0_20:0)	TG	M+NH4	894.8484155	25.419	C56 H112 O6 N1
TG(20:0_16:0_20:5)	TG	M+H	909.7905665	26.25616127	C59 H105 O6
TG(20:0_16:0_22:4)	TG	M+H	939.8375165	22.02346712	C61 H111 O6
TG(20:0_18:1_18:1)	TG	M+Na	937.8194615	21.70787813	C59 H110 O6 Na1
TG(20:0_18:1_20:4)	TG	M+H	937.8218665	21.70411184	C61 H109 O6
TG(20:0_20:4_20:4)	TG	M+H	959.8062165	21.271	C63 H107 O6

TG(20:0_6:0_10:0)	TG	M+NH4	656.5823655	15.557	C39 H78 O6 N1
TG(20:0_9:0_9:0)	TG	M+NH4	684.6136655	15.25	C41 H82 O6 N1
TG(20:0e_6:0_6:0)	TG	M+NH4	586.5405005	16.35908579	C35 H72 O5 N1
TG(20:0e_6:0_8:0)	TG	M+NH4	614.5718005	17.502	C37 H76 O5 N1
TG(20:1_12:0_20:1)	TG	M+NH4	876.8014655	19.674	C55 H106 O6 N1
TG(20:1_14:0_14:0)	TG	M+NH4	822.7545155	26.90806995	C51 H100 O6 N1
TG(20:1_14:0_18:1)	TG	M+NH4	876.8014655	20.018	C55 H106 O6 N1
TG(20:1_18:1_18:2)	TG	M+NH4	928.8327655	25.66013534	C59 H110 O6 N1
TG(20:1_20:3_20:3)	TG	M+NH4	978.8484155	20.90627215	C63 H112 O6 N1
TG(20:1_20:4_20:4)	TG	M+NH4	974.8171155	20.289	C63 H108 O6 N1
TG(20:1_6:0_10:3)	TG	M+H	631.4932165	26.665	C39 H67 O6
TG(20:1_6:0_6:0)	TG	M+Na	603.4595115	14.10931634	C35 H64 O6 Na1
TG(20:1_8:0_10:4)	TG	M+H	657.5088665	16.264	C41 H69 O6
TG(20:1_8:0_8:0)	TG	M+NH4	654.5667155	14.916	C39 H76 O6 N1
TG(20:1e_16:0_16:0)	TG	M+NH4	864.8378505	21.89384428	C55 H110 O5 N1
TG(20:1e_6:0_6:0)	TG	M+NH4	584.5248505	15.05463011	C35 H70 O5 N1
TG(20:1e_6:0_8:0)	TG	M+Na	617.5115465	26.68084654	C37 H70 O5 Na1
TG(20:1e_8:0_8:0)	TG	M+NH4	640.5874505	17.52369789	C39 H78 O5 N1
TG(20:2_20:4_20:4)	TG	M+NH4	972.8014655	19.91913188	C63 H106 O6 N1
TG(20:2e_16:0_16:0)	TG	M+NH4	862.8222005	21.54842994	C55 H108 O5 N1
TG(20:2e_6:0_10:0)	TG	M+H	621.5452515	16.40525145	C39 H73 O5
TG(20:2e_6:0_8:0)	TG	M+NH4	610.5405005	15.2903805	C37 H72 O5 N1
TG(20:3_17:1_17:1)	TG	M+H	881.7592665	14.32290099	C57 H101 O6
TG(20:3_18:2_18:2)	TG	M+H	905.7592665	27.006	C59 H101 O6
TG(20:3_20:3_20:3)	TG	M+H	957.7905665	20.91735258	C63 H105 O6

TG(20:3e_16:0_16:0)	TG	M+NH4	860.8065505	21.22806721	C55 H106 O5 N1
TG(20:3e_6:0_6:0)	TG	M+NH4	580.4935505	5.273	C35 H66 O5 N1
TG(20:4_14:1_20:4)	TG	M+H	873.6966665	19.58534853	C57 H93 O6
TG(20:4e_8:0_8:0)	TG	M+H	617.5139515	26.65064333	C39 H69 O5
TG(20:5_18:2_18:2)	TG	M+H	901.7279665	19.883	C59 H97 O6
TG(21:0_23:0_23:0)	TG	M+NH4	1091.067515	23.47376587	C70 H140 O6 N1
TG(22:0_22:0_24:0)	TG	M+NH4	1105.083165	23.57793285	C71 H142 O6 N1
TG(22:1_10:3_10:3)	TG	M+NH4	726.5667155	14.665	C45 H76 O6 N1
TG(22:1_14:1_22:1)	TG	M+NH4	958.8797155	21.69318817	C61 H116 O6 N1
TG(22:2_14:1_14:1)	TG	M+H	827.7123165	18.446	C53 H95 O6
TG(22:3_11:2_11:2)	TG	M+NH4	754.5980155	15.67706878	C47 H80 O6 N1
TG(23:0_11:1_23:0)	TG	M+NH4	948.8953655	21.78291748	C60 H118 O6 N1
TG(24:0_10:1_10:1)	TG	M+NH4	764.6762655	16.911	C47 H90 O6 N1
TG(24:0_13:0_13:0)	TG	M+Na	857.7568615	19.00886413	C53 H102 O6 Na1
TG(24:0_18:2_20:3)	TG	M+H	993.8844665	22.288	C65 H117 O6
TG(24:1_12:2_24:1)	TG	M+NH4	984.8953655	21.69176744	C63 H118 O6 N1
TG(25:0_16:0_18:0)	TG	M+NH4	978.9423155	22.67563187	C62 H124 O6 N1
TG(25:0_18:0_18:0)	TG	M+NH4	1006.973615	22.89019272	C64 H128 O6 N1
TG(25:0_18:1_18:1)	TG	M+NH4	1002.942315	22.39384527	C64 H124 O6 N1
TG(25:0_18:1_24:0)	TG	M+NH4	1089.051865	23.255	C70 H138 O6 N1
TG(25:0_18:2_18:2)	TG	M+NH4	998.9110155	21.85234091	C64 H120 O6 N1
TG(25:0_6:0_10:0)	TG	M+NH4	726.6606155	18.651	C44 H88 O6 N1
TG(25:0_6:0_18:4)	TG	M+H	813.6966665	20.83042607	C52 H93 O6
TG(25:0_6:0_6:0)	TG	M+NH4	670.5980155	23.14928431	C40 H80 O6 N1
TG(25:0_6:0_8:0)	TG	M+NH4	698.6293155	23.11991814	C42 H84 O6 N1

TG(25:1_18:0_18:0)	TG	M+NH4	1004.957965	22.65180372	C64 H126 O6 N1
TG(25:1_18:1_18:1)	TG	M+NH4	1000.926665	22.10827184	C64 H122 O6 N1
TG(25:1_6:0_10:1)	TG	M+NH4	722.6293155	19.256	C44 H84 O6 N1
TG(25:1_6:0_6:0)	TG	M+NH4	668.5823655	16.17877625	C40 H78 O6 N1
TG(25:1_9:0_9:0)	TG	M+NH4	752.6762655	17.83297185	C46 H90 O6 N1
TG(26:0_16:0_20:3)	TG	M+H	997.9157665	22.7901601	C65 H121 O6
TG(26:0_16:0_23:0)	TG	M+NH4	1063.036215	23.29118414	C68 H136 O6 N1
TG(26:0_18:0_18:0)	TG	M+NH4	1020.989265	23.00095359	C65 H130 O6 N1
TG(26:0_18:0_18:1)	TG	M+NH4	1018.973615	22.76567875	C65 H128 O6 N1
TG(26:0_18:0_20:4)	TG	M+H	1023.931416	22.75992127	C67 H123 O6
TG(26:0_18:1_18:1)	TG	M+NH4	1016.957965	22.52018984	C65 H126 O6 N1
TG(26:0_18:3_22:1)	TG	M+H	1051.962716	22.982	C69 H127 O6
TG(26:0_6:0_20:4)	TG	M+H	855.7436165	21.316	C55 H99 O6
TG(26:0_6:0_8:0)	TG	M+NH4	712.6449655	19.2662021	C43 H86 O6 N1
TG(26:1_10:0_10:0)	TG	M+NH4	794.7232155	19.747	C49 H96 O6 N1
TG(26:1_10:3_10:3)	TG	M+H	765.6027665	17.968	C49 H81 O6
TG(26:1_12:0_12:0)	TG	M+Na	855.7412115	22.554	C53 H100 O6 Na1
TG(26:1_18:1_18:1)	TG	M+NH4	1014.942315	22.24328509	C65 H124 O6 N1
TG(26:1_18:1_18:2)	TG	M+NH4	1012.926665	22.00767595	C65 H122 O6 N1
TG(26:1_6:0_20:5)	TG	M+H	851.7123165	26.74512017	C55 H95 O6
TG(26:1_6:0_8:0)	TG	M+NH4	710.6293155	20.322	C43 H84 O6 N1
TG(27:0_18:0_18:1)	TG	M+NH4	1032.989265	22.86989951	C66 H130 O6 N1
TG(27:0_6:0_8:0)	TG	M+NH4	726.6606155	23.54133591	C44 H88 O6 N1
TG(27:1)	TG	M+NH4	528.4258655	11.03065858	C30 H58 O6 N1
TG(27:1_16:0_16:0)	TG	M+NH4	976.9266655	22.13971762	C62 H122 O6 N1

TG(27:1_18:0_18:1)	TG	M+NH4	1030.973615	22.62478511	C66 H128 O6 N1
TG(27:1_18:1_18:1)	TG	M+NH4	1028.957965	22.37655083	C66 H126 O6 N1
TG(27:1_6:0_6:0)	TG	M+NH4	696.6136655	17.395	C42 H82 O6 N1
TG(27:1_6:0_8:0)	TG	M+NH4	724.6449655	18.139	C44 H86 O6 N1
TG(28:0_17:0_18:0)	TG	M+NH4	1035.004915	23.09585411	C66 H132 O6 N1
TG(28:0_18:0_18:1)	TG	M+NH4	1047.004915	22.98249951	C67 H132 O6 N1
TG(28:0_6:0_22:6)	TG	M+H	907.7749165	22.17215082	C59 H103 O6
TG(28:0_6:0_6:0)	TG	M+NH4	712.6449655	18.56404132	C43 H86 O6 N1
TG(28:0_8:0_8:0)	TG	M+NH4	768.7075655	19.65156121	C47 H94 O6 N1
TG(28:1_18:0_18:1)	TG	M+NH4	1044.989265	22.75214323	C67 H130 O6 N1
TG(28:1_18:1_18:1)	TG	M+NH4	1042.973615	22.52383639	C67 H128 O6 N1
TG(28:1_6:0_20:3)	TG	M+H	883.7749165	19.023	C57 H103 O6
TG(28:1_6:0_20:5)	TG	M+H	879.7436165	26.20275393	C57 H99 O6
TG(28:1_6:0_8:0)	TG	M+NH4	738.6606155	26.944	C45 H88 O6 N1
TG(29:0_10:1_10:1)	TG	M+NH4	834.7545155	21.089	C52 H100 O6 N1
TG(29:0_18:1_18:1)	TG	M+NH4	1059.004915	22.85031194	C68 H132 O6 N1
TG(29:0_6:0_6:0)	TG	M+NH4	726.6606155	25.305	C44 H88 O6 N1
TG(29:0_6:0_8:0)	TG	M+NH4	754.6919155	18.136	C46 H92 O6 N1
TG(29:1_12:0_12:0)	TG	M+NH4	892.8327655	23.923	C56 H110 O6 N1
TG(29:2)	TG	M+NH4	554.4415155	10.992	C32 H60 O6 N1
TG(29:3e)	TG	M+Na	543.4019965	11.289	C32 H56 O5 Na1
TG(30:0_16:0_18:0)	TG	M+NH4	1049.020565	23.20377085	C67 H134 O6 N1
TG(30:0_18:0_18:0)	TG	M+NH4	1077.051865	23.39296257	C69 H138 O6 N1
TG(30:0_18:0_18:1)	TG	M+NH4	1075.036215	23.1882614	C69 H136 O6 N1
TG(30:0_18:1_18:1)	TG	M+NH4	1073.020565	22.96312208	C69 H134 O6 N1

TG(30:0_6:0_18:1)	TG	M+NH4	906.8484155	26.06543876	C57 H112 O6 N1
TG(30:0_6:0_6:0)	TG	M+NH4	740.6762655	18.20013609	C45 H90 O6 N1
TG(30:0e)	TG	M+NH4	558.5092005	15.0147757	C33 H68 O5 N1
TG(30:1_18:1_18:1)	TG	M+NH4	1071.004915	22.68773353	C69 H132 O6 N1
TG(30:1_18:1_19:0)	TG	M+NH4	1087.036215	23.079	C70 H136 O6 N1
TG(30:1_19:0_19:0)	TG	M+NH4	1103.067515	23.37982379	C71 H140 O6 N1
TG(30:1_6:0_6:0)	TG	M+NH4	738.6606155	16.84970642	C45 H88 O6 N1
TG(30:1_6:0_8:0)	TG	M+NH4	766.6919155	17.538	C47 H92 O6 N1
TG(30:5)	TG	M+NH4	562.4102155	8.863513145	C33 H56 O6 N1
TG(31:0)	TG	M+NH4	586.5041155	14.85985404	C34 H68 O6 N1
TG(31:0e)	TG	M+Na	577.4802465	15.692	C34 H66 O5 Na1
TG(32:0)	TG	M+NH4	600.5197655	11.848	C35 H70 O6 N1
TG(32:1)	TG	M+NH4	598.5041155	25.182	C35 H68 O6 N1
TG(32:3)	TG	M+Na	599.4282115	10.223	C35 H60 O6 Na1
TG(33:1)	TG	M+NH4	612.5197655	15.04	C36 H70 O6 N1
TG(34:3)	TG	M+NH4	622.5041155	14.686	C37 H68 O6 N1
TG(35:0)	TG	M+NH4	642.5667155	26.786	C38 H76 O6 N1
TG(35:1)	TG	M+Na	645.5064615	17.08508669	C38 H70 O6 Na1
TG(35:2)	TG	M+Na	643.4908115	12.477	C38 H68 O6 Na1
TG(35:3)	TG	M+H	619.4932165	13.216	C38 H67 O6
TG(36:0)	TG	M+NH4	656.5823655	15.273	C39 H78 O6 N1
TG(36:0e)	TG	M+NH4	642.6031005	18.41345956	C39 H80 O5 N1
TG(36:2)	TG	M+Na	657.5064615	15.222	C39 H70 O6 Na1
TG(36:3e)	TG	M+NH4	636.5561505	15.595	C39 H74 O5 N1
TG(36:4e)	TG	M+NH4	634.5405005	14.18159338	C39 H72 O5 N1

TG(36:5)	TG	M+NH4	646.5041155	12.976	C39 H68 O6 N1
TG(37:0)	TG	M+NH4	670.5980155	18.708	C40 H80 O6 N1
TG(37:2)	TG	M+NH4	666.5667155	17.085	C40 H76 O6 N1
TG(37:4)	TG	M+H	645.5088665	13.481	C40 H69 O6
TG(37:7e)	TG	M+Na	647.4645965	15.34558954	C40 H64 O5 Na1
TG(37:8e)	TG	M+Na	645.4489465	14.409	C40 H62 O5 Na1
TG(38:0)	TG	M+NH4	684.6136655	5.139	C41 H82 O6 N1
TG(38:0e)	TG	M+NH4	670.6344005	19.537	C41 H84 O5 N1
TG(38:10)	TG	M+NH4	664.4571655	16.9033623	C41 H62 O6 N1
TG(38:1e)	TG	M+NH4	668.6187505	18.83380066	C41 H82 O5 N1
TG(38:3)	TG	M+H	661.5401665	16.08942567	C41 H73 O6
TG(38:4e)	TG	M+Na	667.5271965	16.277	C41 H72 O5 Na1
TG(39:0)	TG	M+NH4	698.6293155	26.258	C42 H84 O6 N1
TG(39:6)	TG	M+Na	691.4908115	14.916	C42 H68 O6 Na1
TG(39:7e)	TG	M+Na	675.4958965	16.60198744	C42 H68 O5 Na1
TG(39:8)	TG	M+NH4	682.5041155	15.38	C42 H68 O6 N1
TG(39:8e)	TG	M+Na	673.4802465	15.73531911	C42 H66 O5 Na1
TG(40:1e)	TG	M+NH4	696.6500505	19.42126168	C43 H86 O5 N1
TG(40:2e)	TG	M+NH4	694.6344005	18.823	C43 H84 O5 N1
TG(40:3)	TG	M+H	689.5714665	13.41083476	C43 H77 O6
TG(40:3e)	TG	M+Na	697.5741465	12.807	C43 H78 O5 Na1
TG(40:6e)	TG	M+Na	691.5271965	18.14775961	C43 H72 O5 Na1
TG(40:7e)	TG	M+Na	689.5115465	17.18828254	C43 H70 O5 Na1
TG(40:8e)	TG	M+Na	687.4958965	16.31968385	C43 H68 O5 Na1
TG(41:1)	TG	M+NH4	724.6449655	16.958	C44 H86 O6 N1

TG(41:3)	TG	M+NH4	720.6136655	16.47	C44 H82 O6 N1
TG(41:6)	TG	M+Na	719.5221115	16.226	C44 H72 O6 Na1
TG(41:8e)	TG	M+Na	701.5115465	16.997	C44 H70 O5 Na1
TG(42:0)	TG	M+NH4	740.6762655	23.877	C45 H90 O6 N1
TG(42:0e)	TG	M+NH4	726.6970005	20.419	C45 H92 O5 N1
TG(42:1)	TG	M+NH4	738.6606155	17.39899785	C45 H88 O6 N1
TG(42:2)	TG	M+NH4	736.6449655	18.0060694	C45 H86 O6 N1
TG(42:3)	TG	M+NH4	734.6293155	17.067	C45 H84 O6 N1
TG(42:4)	TG	M+NH4	732.6136655	16.268	C45 H82 O6 N1
TG(42:5)	TG	M+H	713.5714665	18.959	C45 H77 O6
TG(42:6e)	TG	M+Na	719.5584965	18.86517289	C45 H76 O5 Na1
TG(42:7e)	TG	M+Na	717.5428465	18.19488441	C45 H74 O5 Na1
TG(43:0)	TG	M+NH4	754.6919155	19.38828026	C46 H92 O6 N1
TG(43:2)	TG	M+NH4	750.6606155	17.07	C46 H88 O6 N1
TG(43:2e)	TG	M+Na	741.6367465	16.63	C46 H86 O5 Na1
TG(44:1)	TG	M+Na	771.6473115	5.444471585	C47 H88 O6 Na1
TG(44:2)	TG	M+NH4	764.6762655	18.516	C47 H90 O6 N1
TG(44:3)	TG	M+NH4	762.6606155	17.79420201	C47 H88 O6 N1
TG(44:4)	TG	M+NH4	760.6449655	16.674	C47 H86 O6 N1
TG(44:5e)	TG	M+H	727.6235015	16.23538688	C47 H83 O5
TG(44:6)	TG	M+NH4	756.6136655	17.14401809	C47 H82 O6 N1
TG(44:6e)	TG	M+H	725.6078515	16.24	C47 H81 O5
TG(44:7e)	TG	M+Na	745.5741465	18.90636732	C47 H78 O5 Na1
TG(45:2)	TG	M+NH4	778.6919155	19.902	C48 H92 O6 N1
TG(46:4)	TG	M+H	771.6497165	5.450452328	C49 H87 O6

TG(46:5)	TG	M+NH4	786.6606155	17.518	C49 H88 O6 N1
TG(46:6e)	TG	M+H	753.6391515	17.00486755	C49 H85 O5
TG(46:7)	TG	M+NH4	782.6293155	16.683	C49 H84 O6 N1
TG(46:7e)	TG	M+Na	773.6054465	19.466	C49 H82 O5 Na1
TG(47:1)	TG	M+NH4	808.7388655	25.996	C50 H98 O6 N1
TG(47:2)	TG	M+NH4	806.7232155	20.696	C50 H96 O6 N1
TG(47:4)	TG	M+NH4	802.6919155	19.305	C50 H92 O6 N1
TG(47:5)	TG	M+NH4	800.6762655	18.49	C50 H90 O6 N1
TG(47:6e)	TG	M+H	767.6548015	17.337	C50 H87 O5
TG(48:0e)	TG	M+NH4	810.7909005	21.60954212	C51 H104 O5 N1
TG(48:6e)	TG	M+H	781.6704515	17.621	C51 H89 O5
TG(48:7e)	TG	M+Na	801.6367465	19.98550053	C51 H86 O5 Na1
TG(49:0)	TG	M+Na	843.7412115	20.483	C52 H100 O6 Na1
TG(49:1)	TG	M+NH4	836.7701655	22.999	C52 H102 O6 N1
TG(4:0_10:0_16:0)	TG	M+Na	577.4438615	11.95	C33 H62 O6 Na1
TG(4:0_10:0_18:1)	TG	M+Na	603.4595115	14.39457483	C35 H64 O6 Na1
TG(4:0_10:0_20:1)	TG	M+NH4	626.5354155	25.16631592	C37 H72 O6 N1
TG(4:0_10:0_20:4)	TG	M+H	603.4619165	14.87615538	C37 H63 O6
TG(4:0_10:1_18:3)	TG	M+NH4	592.4571655	10.87888835	C35 H62 O6 N1
TG(4:0_10:1_24:0)	TG	M+NH4	682.5980155	25.10250536	C41 H80 O6 N1
TG(4:0_10:2_12:0)	TG	M+NH4	512.3945655	9.402	C29 H54 O6 N1
TG(4:0_10:3_17:1)	TG	M+H	561.4149665	12.30453394	C34 H57 O6
TG(4:0_10:3_20:4)	TG	M+H	597.4149665	10.84827613	C37 H57 O6
TG(4:0_11:0_19:0)	TG	M+NH4	628.5510655	10.136	C37 H74 O6 N1
TG(4:0_12:0_17:0)	TG	M+NH4	614.5354155	16.3555729	C36 H72 O6 N1

TG(4:0_12:0_18:1)	TG	M+Na	631.4908115	16.21600581	C37 H68 O6 Na1
TG(4:0_12:0_18:2)	TG	M+NH4	624.5197655	14.9215651	C37 H70 O6 N1
TG(4:0_12:0_18:3)	TG	M+NH4	622.5041155	13.59571237	C37 H68 O6 N1
TG(4:0_12:0_20:1)	TG	M+NH4	654.5667155	17.99074057	C39 H76 O6 N1
TG(4:0_12:0_20:3)	TG	M+H	633.5088665	16.58273671	C39 H69 O6
TG(4:0_12:0_21:0)	TG	M+NH4	670.5980155	15.54153381	C40 H80 O6 N1
TG(4:0_12:0_22:1)	TG	M+Na	687.5534115	15.99080368	C41 H76 O6 Na1
TG(4:0_12:3_16:0)	TG	M+Na	599.4282115	12.00167364	C35 H60 O6 Na1
TG(4:0_13:0_18:2)	TG	M+NH4	638.5354155	15.07841186	C38 H72 O6 N1
TG(4:0_13:0_18:3)	TG	M+NH4	636.5197655	14.08	C38 H70 O6 N1
TG(4:0_13:0_20:3)	TG	M+NH4	664.5510655	15.581	C40 H74 O6 N1
TG(4:0_13:0_20:5)	TG	M+H	643.4932165	15.06132042	C40 H67 O6
TG(4:0_14:0_17:0)	TG	M+NH4	642.5667155	17.67456268	C38 H76 O6 N1
TG(4:0_14:0_17:1)	TG	M+Na	645.5064615	16.03646544	C38 H70 O6 Na1
TG(4:0_14:0_18:0)	TG	M+NH4	656.5823655	11.44946457	C39 H78 O6 N1
TG(4:0_14:0_18:2)	TG	M+NH4	652.5510655	16.764	C39 H74 O6 N1
TG(4:0_14:0_18:3)	TG	M+Na	655.4908115	14.99625301	C39 H68 O6 Na1
TG(4:0_14:0_19:1)	TG	M+NH4	668.5823655	18.09978929	C40 H78 O6 N1
TG(4:0_14:0_20:1)	TG	M+NH4	682.5980155	18.84348946	C41 H80 O6 N1
TG(4:0_14:0_20:3)	TG	M+H	661.5401665	17.88607819	C41 H73 O6
TG(4:0_14:0_20:4)	TG	M+Na	681.5064615	15.03927175	C41 H70 O6 Na1
TG(4:0_14:0_20:5)	TG	M+NH4	674.5354155	14.40510275	C41 H72 O6 N1
TG(4:0_14:0_22:5)	TG	M+Na	707.5221115	15.38119413	C43 H72 O6 Na1
TG(4:0_14:0_22:6)	TG	M+NH4	700.5510655	14.8745613	C43 H74 O6 N1
TG(4:0_14:1_16:0)	TG	M+Na	631.4908115	15.72851221	C37 H68 O6 Na1

TG(4:0_14:1_17:0)	TG	M+NH4	640.5510655	16.36295518	C38 H74 O6 N1
TG(4:0_14:1_18:0)	TG	M+NH4	654.5667155	17.36765928	C39 H76 O6 N1
TG(4:0_14:1_18:1)	TG	M+NH4	652.5510655	16.24558449	C39 H74 O6 N1
TG(4:0_14:1_18:2)	TG	M+Na	655.4908115	15.76077964	C39 H68 O6 Na1
TG(4:0_14:1_18:3)	TG	M+H	631.4932165	15.59767355	C39 H67 O6
TG(4:0_14:2_18:1)	TG	M+NH4	650.5354155	15.42435861	C39 H72 O6 N1
TG(4:0_14:2_20:4)	TG	M+H	655.4932165	14.99619478	C41 H67 O6
TG(4:0_14:3_16:0)	TG	M+H	605.4775665	15.32588752	C37 H65 O6
TG(4:0_14:3_18:2)	TG	M+H	629.4775665	14.38590826	C39 H65 O6
TG(4:0_14:4_16:0)	TG	M+H	603.4619165	14.238	C37 H63 O6
TG(4:0_14:4_18:1)	TG	M+H	629.4775665	14.943	C39 H65 O6
TG(4:0_15:0_18:0)	TG	M+NH4	670.5980155	19.12041272	C40 H80 O6 N1
TG(4:0_15:0_18:1)	TG	M+NH4	668.5823655	17.759	C40 H78 O6 N1
TG(4:0_15:0_18:3)	TG	M+H	647.5245165	17.388	C40 H71 O6
TG(4:0_15:0_18:4)	TG	M+H	645.5088665	15.99158386	C40 H69 O6
TG(4:0_15:0_19:1)	TG	M+NH4	682.5980155	14.33	C41 H80 O6 N1
TG(4:0_15:0_20:4)	TG	M+Na	695.5221115	16.01743419	C42 H72 O6 Na1
TG(4:0_15:0_22:5)	TG	M+NH4	716.5823655	16.03019223	C44 H78 O6 N1
TG(4:0_16:0_17:1)	TG	M+NH4	668.5823655	17.49409039	C40 H78 O6 N1
TG(4:0_16:0_18:2)	TG	M+Na	685.5377615	17.01385828	C41 H74 O6 Na1
TG(4:0_16:0_19:1)	TG	M+NH4	696.6136655	18.30696195	C42 H82 O6 N1
TG(4:0_16:0_20:1)	TG	M+NH4	710.6293155	18.98520709	C43 H84 O6 N1
TG(4:0_16:0_20:3)	TG	M+NH4	706.5980155	16.03922682	C43 H80 O6 N1
TG(4:0_16:0_20:4)	TG	M+NH4	704.5823655	16.65165127	C43 H78 O6 N1
TG(4:0_16:0_20:5)	TG	M+NH4	702.5667155	15.7489534	C43 H76 O6 N1

TG(4:0_16:0_22:6)	TG	M+NH4	728.5823655	16.15868825	C45 H78 O6 N1
TG(4:0_16:0_23:0)	TG	M+NH4	754.6919155	20.03395792	C46 H92 O6 N1
TG(4:0_16:1_16:1)	TG	M+Na	657.5064615	15.78321518	C39 H70 O6 Na1
TG(4:0_16:1_17:1)	TG	M+NH4	666.5667155	16.38346435	C40 H76 O6 N1
TG(4:0_16:1_18:0)	TG	M+NH4	682.5980155	22.013	C41 H80 O6 N1
TG(4:0_16:1_18:1)	TG	M+NH4	680.5823655	26.76862115	C41 H78 O6 N1
TG(4:0_16:1_18:3)	TG	M+Na	681.5064615	14.85108578	C41 H70 O6 Na1
TG(4:0_16:1_20:5)	TG	M+NH4	700.5510655	14.01670082	C43 H74 O6 N1
TG(4:0_17:0_18:1)	TG	M+NH4	696.6136655	19.327	C42 H82 O6 N1
TG(4:0_17:0_18:4)	TG	M+NH4	690.5667155	15.6462827	C42 H76 O6 N1
TG(4:0_17:0_20:4)	TG	M+H	701.5714665	18.476	C44 H77 O6
TG(4:0_17:0_22:5)	TG	M+NH4	744.6136655	17.00266206	C46 H82 O6 N1
TG(4:0_17:1_18:1)	TG	M+NH4	694.5980155	18.09611569	C42 H80 O6 N1
TG(4:0_17:1_18:2)	TG	M+NH4	692.5823655	17.25814558	C42 H78 O6 N1
TG(4:0_17:1_18:3)	TG	M+H	673.5401665	17.38	C42 H73 O6
TG(4:0_17:1_18:4)	TG	M+H	671.5245165	16.37640804	C42 H71 O6
TG(4:0_17:1_20:5)	TG	M+H	697.5401665	16.482	C44 H73 O6
TG(4:0_18:0_18:0)	TG	M+NH4	712.6449655	17.37685811	C43 H86 O6 N1
TG(4:0_18:0_18:1)	TG	M+NH4	710.6293155	6.799	C43 H84 O6 N1
TG(4:0_18:0_18:3)	TG	M+NH4	706.5980155	17.77924907	C43 H80 O6 N1
TG(4:0_18:0_20:3)	TG	M+H	717.6027665	26.25710243	C45 H81 O6
TG(4:0_18:0_20:4)	TG	M+Na	737.5690615	17.52939521	C45 H78 O6 Na1
TG(4:0_18:0_20:5)	TG	M+NH4	730.5980155	16.98915176	C45 H80 O6 N1
TG(4:0_18:1_18:2)	TG	M+NH4	706.5980155	17.01233647	C43 H80 O6 N1
TG(4:0_18:1_18:3)	TG	M+NH4	704.5823655	16.10742208	C43 H78 O6 N1

TG(4:0_18:1_20:2)	TG	M+NH4	734.6293155	18.40980736	C45 H84 O6 N1
TG(4:0_18:1_20:4)	TG	M+NH4	730.5980155	16.65022191	C45 H80 O6 N1
TG(4:0_18:1_22:5)	TG	M+NH4	756.6136655	16.66922786	C47 H82 O6 N1
TG(4:0_18:1_22:6)	TG	M+NH4	754.5980155	16.19931272	C47 H80 O6 N1
TG(4:0_18:2_18:2)	TG	M+Na	709.5377615	16.30041031	C43 H74 O6 Na1
TG(4:0_18:2_18:3)	TG	M+NH4	702.5667155	15.06288039	C43 H76 O6 N1
TG(4:0_18:2_19:0)	TG	M+NH4	722.6293155	18.46719429	C44 H84 O6 N1
TG(4:0_18:2_19:1)	TG	M+NH4	720.6136655	17.74926707	C44 H82 O6 N1
TG(4:0_18:2_20:4)	TG	M+NH4	728.5823655	15.63690287	C45 H78 O6 N1
TG(4:0_18:2_20:5)	TG	M+H	709.5401665	16.25840802	C45 H73 O6
TG(4:0_18:3_18:3)	TG	M+H	683.5245165	16.08993786	C43 H71 O6
TG(4:0_18:3_18:4)	TG	M+H	681.5088665	15.017	C43 H69 O6
TG(4:0_18:3_20:4)	TG	M+H	709.5401665	16.656	C45 H73 O6
TG(4:0_18:3_20:5)	TG	M+H	707.5245165	15.03	C45 H71 O6
TG(4:0_20:3_20:3)	TG	M+H	739.5871165	18.13187237	C47 H79 O6
TG(4:0_20:3_20:5)	TG	M+H	735.5558165	16.97065344	C47 H75 O6
TG(4:0_20:4_20:4)	TG	M+H	735.5558165	16.3902798	C47 H75 O6
TG(4:0_20:4_20:5)	TG	M+H	733.5401665	15.776	C47 H73 O6
TG(4:0_20:4_22:5)	TG	M+H	761.5714665	16.68538829	C49 H77 O6
TG(4:0_20:5_20:5)	TG	M+H	731.5245165	14.663	C47 H71 O6
TG(4:0_6:0_13:0)	TG	M+Na	479.3343115	8.916921999	C26 H48 O6 Na1
TG(4:0_6:0_14:1)	TG	M+NH4	486.3789155	8.972044995	C27 H52 O6 N1
TG(4:0_6:0_16:0)	TG	M+Na	521.3812615	9.216631904	C29 H54 O6 Na1
TG(4:0_6:0_16:1)	TG	M+NH4	514.4102155	10.44946105	C29 H56 O6 N1
TG(4:0_6:0_18:2)	TG	M+NH4	540.4258655	10.27058684	C31 H58 O6 N1

TG(4:0_6:0_18:3)	TG	M+Na	543.3656115	9.222029013	C31 H52 O6 Na1
TG(4:0_6:0_19:1)	TG	M+Na	561.4125615	12.09055437	C32 H58 O6 Na1
TG(4:0_6:0_20:0)	TG	M+NH4	572.4884655	12.785	C33 H66 O6 N1
TG(4:0_6:0_20:5)	TG	M+H	545.3836665	10.266	C33 H53 O6
TG(4:0_6:0_21:0)	TG	M+Na	591.4595115	14.85985404	C34 H64 O6 Na1
TG(4:0_6:0_22:0)	TG	M+NH4	600.5197655	16.32386526	C35 H70 O6 N1
TG(4:0_6:0_22:1)	TG	M+NH4	598.5041155	14.40547127	C35 H68 O6 N1
TG(4:0_6:0_22:2)	TG	M+Na	601.4438615	13.27139362	C35 H62 O6 Na1
TG(4:0_6:0_23:0)	TG	M+NH4	614.5354155	14.58440715	C36 H72 O6 N1
TG(4:0_6:0_23:1)	TG	M+NH4	612.5197655	15.35941529	C36 H70 O6 N1
TG(4:0_6:0_24:0)	TG	M+NH4	628.5510655	23.08455257	C37 H74 O6 N1
TG(4:0_8:0_18:1)	TG	M+NH4	570.4728155	12.83013161	C33 H64 O6 N1
TG(4:0_8:0_18:2)	TG	M+Na	573.4125615	11.95702103	C33 H58 O6 Na1
TG(4:0_8:0_20:4)	TG	M+H	575.4306165	12.83748314	C35 H59 O6
TG(4:0_8:0_20:5)	TG	M+H	573.4149665	12.139	C35 H57 O6
TG(4:0_8:0_23:1)	TG	M+NH4	640.5510655	17.08508669	C38 H74 O6 N1
TG(4:0_8:0_24:0)	TG	M+NH4	656.5823655	19.33094618	C39 H78 O6 N1
TG(4:0_8:0_24:1)	TG	M+NH4	654.5667155	18.312	C39 H76 O6 N1
TG(4:0_9:0_22:0)	TG	M+NH4	642.5667155	14.19	C38 H76 O6 N1
TG(50:0)	TG	M+NH4	852.8014655	23.804	C53 H106 O6 N1
TG(50:1)	TG	M+NH4	850.7858155	21.08349485	C53 H104 O6 N1
TG(50:2)	TG	M+NH4	848.7701655	26.67842949	C53 H102 O6 N1
TG(50:3e)	TG	M+Na	837.7306465	18.48498487	C53 H98 O5 Na1
TG(50:5e)	TG	M+H	811.7174015	18.54207005	C53 H95 O5
TG(52:0)	TG	M+NH4	880.8327655	20.714	C55 H110 O6 N1

TG(52:1)	TG	M+Na	883.7725115	18.999	C55 H104 O6 Na1
TG(52:3)	TG	M+NH4	874.7858155	19.375	C55 H104 O6 N1
TG(52:5e)	TG	M+H	839.7487015	19.07	C55 H99 O5
TG(52:6e)	TG	M+H	837.7330515	18.48265318	C55 H97 O5
TG(52:7e)	TG	M+Na	857.6993465	20.898	C55 H94 O5 Na1
TG(53:0)	TG	M+Na	899.8038115	26.929	C56 H108 O6 Na1
TG(53:1)	TG	M+NH4	892.8327655	25.068	C56 H110 O6 N1
TG(53:2)	TG	M+NH4	890.8171155	23.184	C56 H108 O6 N1
TG(54:1)	TG	M+NH4	906.8484155	22.579	C57 H112 O6 N1
TG(54:1e)	TG	M+NH4	892.8691505	22.188	C57 H114 O5 N1
TG(54:2)	TG	M+NH4	904.8327655	21.009	C57 H110 O6 N1
TG(54:2e)	TG	M+NH4	890.8535005	21.87247137	C57 H112 O5 N1
TG(54:3)	TG	M+NH4	902.8171155	26.391	C57 H108 O6 N1
TG(54:3e)	TG	M+Na	893.7932465	19.516	C57 H106 O5 Na1
TG(54:5)	TG	M+H	881.7592665	20.725	C57 H101 O6
TG(54:6)	TG	M+NH4	896.7701655	25.73440836	C57 H102 O6 N1
TG(54:6e)	TG	M+Na	887.7462965	26.834	C57 H100 O5 Na1
TG(54:7e)	TG	M+H	863.7487015	18.472	C57 H99 O5
TG(55:2)	TG	M+NH4	918.8484155	22.966	C58 H112 O6 N1
TG(55:8)	TG	M+NH4	906.7545155	20.959	C58 H100 O6 N1
TG(55:8e)	TG	M+Na	897.7306465	26.823	C58 H98 O5 Na1
TG(56:6e)	TG	M+Na	915.7775965	21.923	C59 H104 O5 Na1
TG(56:7)	TG	M+NH4	922.7858155	25.671	C59 H104 O6 N1
TG(59:2)	TG	M+NH4	974.9110155	21.707	C62 H120 O6 N1
TG(59:3)	TG	M+NH4	972.8953655	20.96602933	C62 H118 O6 N1

TG(62:1)	TG	M+Na	1023.929011	22.488	C65 H124 O6 Na1
TG(62:6)	TG	M+NH4	1008.895365	21.56327603	C65 H118 O6 N1
TG(63:2)	TG	M+NH4	1030.973615	21.88241948	C66 H128 O6 N1
TG(68:2)	TG	M+NH4	1101.051865	23.16333076	C71 H138 O6 N1
TG(68:3)	TG	M+NH4	1099.036215	22.91475116	C71 H136 O6 N1
TG(6:0_10:0_10:0)	TG	M+NH4	516.4258655	11.71884767	C29 H58 O6 N1
TG(6:0_10:0_18:2)	TG	M+NH4	624.5197655	13.09752418	C37 H70 O6 N1
TG(6:0_10:0_24:1)	TG	M+NH4	710.6293155	17.68	C43 H84 O6 N1
TG(6:0_10:1_10:1)	TG	M+NH4	512.3945655	9.039461182	C29 H54 O6 N1
TG(6:0_10:1_18:3)	TG	M+NH4	620.4884655	12.04597093	C37 H66 O6 N1
TG(6:0_12:0_12:0)	TG	M+NH4	572.4884655	22.0382763	C33 H66 O6 N1
TG(6:0_12:0_12:3)	TG	M+H	549.4149665	12.624	C33 H57 O6
TG(6:0_12:4_22:0)	TG	M+H	687.5558165	17.961	C43 H75 O6
TG(6:0_13:0_14:3)	TG	M+H	591.4619165	14.627	C36 H63 O6
TG(6:0_14:0_18:1)	TG	M+NH4	682.5980155	17.85015019	C41 H80 O6 N1
TG(6:0_14:0_20:3)	TG	M+H	689.5714665	19.5148914	C43 H77 O6
TG(6:0_14:0_20:4)	TG	M+H	687.5558165	26.718	C43 H75 O6
TG(6:0_14:0_22:5)	TG	M+NH4	730.5980155	16.17540891	C45 H80 O6 N1
TG(6:0_14:1_17:1)	TG	M+NH4	666.5667155	15.85533211	C40 H76 O6 N1
TG(6:0_14:1_20:4)	TG	M+H	685.5401665	16.728	C43 H73 O6
TG(6:0_17:1_17:1)	TG	M+NH4	708.6136655	17.8993219	C43 H82 O6 N1
TG(6:0_17:1_18:1)	TG	M+NH4	722.6293155	18.19820002	C44 H84 O6 N1
TG(6:0_17:1_18:2)	TG	M+NH4	720.6136655	18.067	C44 H82 O6 N1
TG(6:0_17:1_18:3)	TG	M+NH4	718.5980155	16.5036787	C44 H80 O6 N1
TG(6:0_17:1_20:4)	TG	M+H	727.5871165	18.22807973	C46 H79 O6

TG(6:0_18:1_18:2)	TG	M+NH4	734.6293155	17.8416461	C45 H84 O6 N1
TG(6:0_18:1_18:3)	TG	M+NH4	732.6136655	17.07152788	C45 H82 O6 N1
TG(6:0_18:1_20:4)	TG	M+H	741.6027665	18.59642221	C47 H81 O6
TG(6:0_18:1_22:5)	TG	M+NH4	784.6449655	17.50467284	C49 H86 O6 N1
TG(6:0_18:1_22:6)	TG	M+NH4	782.6293155	17.1201642	C49 H84 O6 N1
TG(6:0_18:2_18:2)	TG	M+Na	737.5690615	16.667	C45 H78 O6 Na1
TG(6:0_18:2_20:5)	TG	M+H	737.5714665	17.06364409	C47 H77 O6
TG(6:0_18:3_18:3)	TG	M+Na	733.5377615	15.08814461	C45 H74 O6 Na1
TG(6:0_18:3_20:4)	TG	M+H	737.5714665	17.375	C47 H77 O6
TG(6:0_18:3_22:5)	TG	M+H	763.5871165	17.48937892	C49 H79 O6
TG(6:0_6:0_10:0)	TG	M+NH4	460.3632655	8.839	C25 H50 O6 N1
TG(6:0_6:0_10:1)	TG	M+NH4	458.3476155	6.819112102	C25 H48 O6 N1
TG(6:0_6:0_12:0)	TG	M+NH4	488.3945655	10.15115424	C27 H54 O6 N1
TG(6:0_6:0_12:2)	TG	M+NH4	484.3632655	7.923	C27 H50 O6 N1
TG(6:0_6:0_13:0)	TG	M+Na	507.3656115	10.64782811	C28 H52 O6 Na1
TG(6:0_6:0_14:0)	TG	M+NH4	516.4258655	16.432	C29 H58 O6 N1
TG(6:0_6:0_14:1)	TG	M+Na	519.3656115	10.19514555	C29 H52 O6 Na1
TG(6:0_6:0_14:3)	TG	M+NH4	510.3789155	7.988	C29 H52 O6 N1
TG(6:0_6:0_17:1)	TG	M+NH4	556.4571655	12.54362289	C32 H62 O6 N1
TG(6:0_6:0_18:2)	TG	M+NH4	568.4571655	11.64741836	C33 H62 O6 N1
TG(6:0_6:0_18:3)	TG	M+NH4	566.4415155	10.556174	C33 H60 O6 N1
TG(6:0_6:0_20:2)	TG	M+NH4	596.4884655	13.04983026	C35 H66 O6 N1
TG(6:0_6:0_20:3)	TG	M+NH4	594.4728155	12.32829498	C35 H64 O6 N1
TG(6:0_6:0_20:5)	TG	M+H	573.4149665	11.733	C35 H57 O6
TG(6:0_6:0_21:1)	TG	M+Na	617.4751615	14.71297769	C36 H66 O6 Na1

TG(6:0_6:0_22:1)	TG	M+NH4	626.5354155	14.04971465	C37 H72 O6 N1
TG(6:0_6:0_22:2)	TG	M+Na	629.4751615	14.44445234	C37 H66 O6 Na1
TG(6:0_6:0_22:6)	TG	M+H	599.4306165	11.80615136	C37 H59 O6
TG(6:0_6:0_24:2)	TG	M+Na	657.5064615	26.767	C39 H70 O6 Na1
TG(6:0_6:0_8:0)	TG	M+NH4	432.3319655	6.879	C23 H46 O6 N1
TG(6:0_8:0_10:0)	TG	M+NH4	488.3945655	11.02	C27 H54 O6 N1
TG(6:0_8:0_10:1)	TG	M+NH4	486.3789155	8.511218152	C27 H52 O6 N1
TG(6:0_8:0_14:0)	TG	M+Na	549.4125615	12.67311831	C31 H58 O6 Na1
TG(6:0_8:0_17:1)	TG	M+NH4	584.4884655	13.48921648	C34 H66 O6 N1
TG(6:0_8:0_18:1)	TG	M+NH4	598.5041155	12.84825443	C35 H68 O6 N1
TG(6:0_8:0_18:3)	TG	M+NH4	594.4728155	11.82260617	C35 H64 O6 N1
TG(6:0_8:0_20:2)	TG	M+Na	629.4751615	15.40535844	C37 H66 O6 Na1
TG(6:0_8:0_20:4)	TG	M+NH4	620.4884655	12.3986433	C37 H66 O6 N1
TG(6:0_8:0_20:5)	TG	M+NH4	618.4728155	11.4259221	C37 H64 O6 N1
TG(6:0_8:0_21:0)	TG	M+NH4	642.5667155	17.1804205	C38 H76 O6 N1
TG(6:0_8:0_21:1)	TG	M+Na	645.5064615	13.47469738	C38 H70 O6 Na1
TG(6:0_8:0_22:0)	TG	M+NH4	656.5823655	7.178683518	C39 H78 O6 N1
TG(6:0_8:0_22:5)	TG	M+NH4	646.5041155	12.7084901	C39 H68 O6 N1
TG(6:0_8:0_22:6)	TG	M+H	627.4619165	13.14047589	C39 H63 O6
TG(6:0_8:0_8:0)	TG	M+NH4	460.3632655	8.09110604	C25 H50 O6 N1
TG(6:0_9:0_18:2)	TG	M+Na	615.4595115	13.65894016	C36 H64 O6 Na1
TG(70:2)	TG	M+NH4	1129.083165	23.356	C73 H142 O6 N1
TG(8:0_10:0_10:0)	TG	M+Na	549.4125615	12.96653725	C31 H58 O6 Na1
TG(8:0_10:0_12:0)	TG	M+NH4	572.4884655	16.95674363	C33 H66 O6 N1
TG(8:0_10:0_20:5)	TG	M+NH4	674.5354155	14.03695377	C41 H72 O6 N1

TG(8:0_10:3_17:1)	TG	M+H	617.4775665	14.68227317	C38 H65 O6
TG(8:0_10:4_22:1)	TG	M+H	685.5401665	17.45286669	C43 H73 O6
TG(8:0_11:2_18:1)	TG	M+H	647.5245165	14.60000072	C40 H71 O6
TG(8:0_12:0_12:3)	TG	M+H	577.4462665	14.169	C35 H61 O6
TG(8:0_12:3_18:1)	TG	M+H	659.5245165	16.59683329	C41 H71 O6
TG(8:0_14:0_14:0)	TG	M+NH4	656.5823655	14.13271132	C39 H78 O6 N1
TG(8:0_14:0_14:1)	TG	M+NH4	654.5667155	23.95233885	C39 H76 O6 N1
TG(8:0_14:0_17:1)	TG	M+NH4	696.6136655	18.78309273	C42 H82 O6 N1
TG(8:0_14:0_20:5)	TG	M+H	713.5714665	17.94771728	C45 H77 O6
TG(8:0_14:1_18:1)	TG	M+Na	713.5690615	17.92094007	C43 H78 O6 Na1
TG(8:0_14:3_14:3)	TG	M+H	627.4619165	13.58	C39 H63 O6
TG(8:0_14:4_18:2)	TG	M+H	683.5245165	16.705	C43 H71 O6
TG(8:0_17:1_18:3)	TG	M+NH4	746.6293155	17.52070122	C46 H84 O6 N1
TG(8:0_18:1_18:3)	TG	M+NH4	760.6449655	17.96667407	C47 H86 O6 N1
TG(8:0_18:1_22:5)	TG	M+NH4	812.6762655	18.33265101	C51 H90 O6 N1
TG(8:0_18:2_18:3)	TG	M+NH4	758.6293155	17.14462642	C47 H84 O6 N1
TG(8:0_18:3_22:5)	TG	M+H	791.6184165	18.258	C51 H83 O6
TG(8:0_20:3_20:3)	TG	M+H	795.6497165	19.24861006	C51 H87 O6
TG(8:0_8:0_10:0)	TG	M+NH4	516.4258655	11.429153	C29 H58 O6 N1
TG(8:0_8:0_11:1)	TG	M+NH4	528.4258655	10.76894608	C30 H58 O6 N1
TG(8:0_8:0_12:0)	TG	M+NH4	544.4571655	25.40862212	C31 H62 O6 N1
TG(8:0_8:0_14:0)	TG	M+NH4	572.4884655	20.082	C33 H66 O6 N1
TG(8:0_8:0_14:1)	TG	M+NH4	570.4728155	11.507	C33 H64 O6 N1
TG(8:0_8:0_18:1)	TG	M+NH4	626.5354155	16.854	C37 H72 O6 N1
TG(8:0_8:0_18:3)	TG	M+NH4	622.5041155	13.16205548	C37 H68 O6 N1

TG(8:0_8:0_20:3)	TG	M+Na	655.4908115	14.59262286	C39 H68 O6 Na1
TG(8:0_8:0_22:0)	TG	M+NH4	684.6136655	2.766	C41 H82 O6 N1
TG(8:0_8:0_22:6)	TG	M+H	655.4932165	14.5291876	C41 H67 O6
TG(8:0_8:0_24:1)	TG	M+NH4	710.6293155	17.966	C43 H84 O6 N1
TG(8:0_8:0_24:2)	TG	M+Na	713.5690615	26.66168969	C43 H78 O6 Na1
TG(8:0_8:0_8:0)	TG	M+NH4	488.3945655	9.869898355	C27 H54 O6 N1
TG(8:0_8:0_9:0)	TG	M+NH4	502.4102155	10.39997306	C28 H56 O6 N1
TG(9:0_18:1_18:2)	TG	M+NH4	776.6762655	18.89499186	C48 H90 O6 N1
TG(9:0_18:2_18:2)	TG	M+NH4	774.6606155	18.29275827	C48 H88 O6 N1
TG(9:0_23:0_23:0)	TG	M+NH4	922.8797155	22.777	C58 H116 O6 N1
TG(9:0_9:0_21:1)	TG	M+NH4	696.6136655	15.60480389	C42 H82 O6 N1
TG(9:0_9:0_23:0)	TG	M+NH4	726.6606155	17.66386249	C44 H88 O6 N1
TG(9:0_9:0_9:0)	TG	M+NH4	530.4415155	12.00917266	C30 H60 O6 N1
WE(13:0_20:3)	WE	M+NH4	506.4931555	12.56239285	H64 C33 O2 N1
WE(18:0_17:3)	WE	M+H	517.4979065	14.226	H65 C35 O2
WE(19:0_17:3)	WE	M+NH4	548.5401055	15.226	H70 C36 O2 N1
ZyE(18:0)	ZyE	M+NH4	668.6340055	21.27697633	C45 H82 O2 N1
ZyE(18:1)	ZyE	M+NH4	666.6183555	20.81411983	C45 H80 O2 N1
ZyE(18:2)	ZyE	M+H	647.5761565	18.252	C45 H75 O2
ZyE(20:3)	ZyE	M+NH4	690.6183555	20.53836481	C47 H80 O2 N1
ZyE(20:4)	ZyE	M+H	671.5761565	20.82152985	C47 H75 O2
ZyE(21:4)	ZyE	M+H	685.5918065	15.327	C48 H77 O2
ZyE(22:0)	ZyE	M+H	707.6700565	10.882	C49 H87 O2
ZyE(22:6)	ZyE	M+NH4	712.6027055	15.89563358	C49 H78 O2 N1
ZyE(23:1)	ZyE	M+NH4	736.6966055	15.593	C50 H90 O2 N1

ZyE(23:6)	ZyE	M+NH4	726.6183555	17.357	C50 H80 O2 N1
ZyE(24:6)	ZyE	M+NH4	740.6340055	17.07539183	C51 H82 O2 N1
ZyE(24:7)	ZyE	M+NH4	738.6183555	16.63891429	C51 H80 O2 N1
ZyE(25:5)	ZyE	M+H	739.6387565	16.359	C52 H83 O2
ZyE(25:6)	ZyE	M+NH4	754.6496555	17.009	C52 H84 O2 N1
ZyE(26:6)	ZyE	M+NH4	768.6653055	17.92684875	C53 H86 O2 N1
ZyE(30:6)	ZyE	M+NH4	824.7279055	18.074	C57 H94 O2 N1
ZyE(32:6)	ZyE	M+NH4	852.7592055	18.732	C59 H98 O2 N1
ZyE(33:5)	ZyE	M+NH4	868.7905055	19.08316628	C60 H102 O2 N1
ZyE(33:6)	ZyE	M+NH4	866.7748555	19.03856767	C60 H100 O2 N1
ZyE(34:5)	ZyE	M+NH4	882.8061555	19.693	C61 H104 O2 N1
ZyE(34:6)	ZyE	M+NH4	880.7905055	19.26659309	C61 H102 O2 N1
ZyE(35:6)	ZyE	M+NH4	894.8061555	19.535	C62 H104 O2 N1
ZyE(36:6)	ZyE	M+NH4	908.8218055	19.40228584	C63 H106 O2 N1
ZyE(37:5)	ZyE	M+H	907.8265565	20.348	C64 H107 O2
ZyE(37:6)	ZyE	M+NH4	922.8374555	20.32561718	C64 H108 O2 N1
dMePE(15:0_18:1)	dMePE	M-H	730.5392305	14.718	C40 H77 O8 N1 P1
dMePE(16:0_12:0)	dMePE	M-H	662.4766305	12.96946747	C35 H69 O8 N1 P1
dMePE(16:0_14:0)	dMePE	M-H	690.5079305	14.413	C37 H73 O8 N1 P1
dMePE(16:0_16:0)	dMePE	M-H	718.5392305	14.06082639	C39 H77 O8 N1 P1
dMePE(16:0_18:1)	dMePE	M-H	744.5548805	14.12170554	C41 H79 O8 N1 P1
dMePE(16:0_18:2)	dMePE	M-H	742.5392305	12.9797092	C41 H77 O8 N1 P1
dMePE(16:0_18:3)	dMePE	M-H	740.5235805	13.388	C41 H75 O8 N1 P1
dMePE(16:1_18:1)	dMePE	M-H	742.5392305	14.51838334	C41 H77 O8 N1 P1
dMePE(18:0_16:0)	dMePE	M-H	746.5705305	15.42489067	C41 H81 O8 N1 P1

dMePE(18:0_18:1)	dMePE	M-H	772.5861805	15.46529871	C43 H83 O8 N1 P1
dMePE(18:0_18:2)	dMePE	M-H	770.5705305	14.18433833	C43 H81 O8 N1 P1
dMePE(18:1_14:0)	dMePE	M-H	716.5235805	12.68478445	C39 H75 O8 N1 P1
dMePE(18:1_18:2)	dMePE	M-H	768.5548805	13.0434703	C43 H79 O8 N1 P1
dMePE(18:2_18:2)	dMePE	M-H	766.5392305	14.44748968	C43 H77 O8 N1 P1
dMePE(19:1_16:0)	dMePE	M-H	758.5705305	16.405	C42 H81 O8 N1 P1
dMePE(32:1_20:4)	dMePE	M-H	988.7739805	18.915	C59 H107 O8 N1 P1
dMePE(35:2)	dMePE	M-H	756.5548805	14.748	C42 H79 O8 N1 P1
dMePE(36:3)	dMePE	M-H	768.5548805	15.47011855	C43 H79 O8 N1 P1
phSM(d16:0_18:1)	phSM	M+HCOO	763.5606945	12.185	C40 H80 O9 N2 P1

Table S2. The differential lipids identified between yak colostrum (YC) and cow colostrum (CC) (VIP > 1，p value < 0.05, FC > 2 or FC < 0.5).

Accession	YC_Mean	CC_Mean	FC	log2FC	P.value	-log10(P.value)	FDR	VIP
BisMePA(4:0_24:0)	10607754.86	822275.44	12.9	3.69	2.33794E-05	4.63	0.000519022	1.392526822
Cer(d39:0)	8442504.3	1092331.12	7.73	2.95	0.0002167	3.66	0.002186698	1.403662918
Cer(t18:1_23:0)	1304298.16	459200.88	2.84	1.51	0.029001993	1.54	0.057392922	1.012149947
Cer(t39:1)	811763.38	151928.75	5.34	2.42	0.009483159	2.02	0.02490094	1.16884812
Cer(t41:1)	558732.26	143877.11	3.88	1.96	0.011097557	1.95	0.027766633	1.062836182
DG(22:1e)	22105094.39	6487103.08	3.41	1.77	0.000515515	3.29	0.003495686	1.281788183
DG(22:2e)	1602689902	675393113.4	2.37	1.25	0.00098835	3.01	0.005363444	1.346908259
DG(23:0e)	17998924.69	3891905.47	4.62	2.21	8.91658E-06	5.05	0.000322273	1.414268755
DG(24:0e)	48386927.3	12214850.42	3.96	1.99	0.000153568	3.81	0.001785781	1.334139361
DG(24:1e)	3418444.68	1123949.86	3.04	1.6	0.013601166	1.87	0.032435594	1.115904478
DG(24:3e)	35687967.01	7098037.75	5.03	2.33	0.001653152	2.78	0.00725104	1.304691865
DG(25:0e)	2936620.37	436142.04	6.73	2.75	0.003495234	2.46	0.01219446	1.251934637
DG(25:2e)	25997586.69	5072091.76	5.13	2.36	0.008850607	2.05	0.023803065	1.185476473
DG(26:0e)	61983623.84	18445333.7	3.36	1.75	0.000951508	3.02	0.005353874	1.249856935
DG(27:2e)	23990483.58	10255605.9	2.34	1.23	0.014878154	1.83	0.034668369	1.030651939
DG(28:3e)	61777450.33	18502983.64	3.34	1.74	0.001027589	2.99	0.005404529	1.245452011
DG(30:1e)	53737474.26	24829000.86	2.16	1.11	0.001701083	2.77	0.007313458	1.214495385
DG(34:0)	23812757.88	10800956.83	2.2	1.14	0.002117084	2.67	0.00844758	1.200895306
DG(35:3e)	33993085.09	7723153.45	4.4	2.14	0.007734293	2.11	0.021657147	1.179850254
Hex1Cer(m39:0+O)	3533011.3	917980.35	3.85	1.94	0.005091624	2.29	0.015679318	1.136300983
Hex2Cer(d38:0)	14983101.51	3558330.57	4.21	2.07	0.006524175	2.19	0.018966708	1.211962604
Hex2Cer(d39:0)	19198745.38	1863587.46	10.3	3.36	0.002765561	2.56	0.010294969	1.298822666

Accession	YC_Mean	CC_Mean	FC	log2FC	P.value	-log10(P.value)	FDR	VIP
Hex2Cer(d40:0)	33877165.38	12293392.76	2.76	1.46	0.01342982	1.87	0.032152569	1.041756832
Hex2Cer(d41:0)	12344602.35	3133528.94	3.94	1.98	0.017225494	1.76	0.038450326	1.10151261
Hex2Cer(m38:0+O)	14983101.51	3558330.57	4.21	2.07	0.006524175	2.19	0.018966708	1.211962604
Hex2Cer(m39:0+O)	19198745.38	1863587.46	10.3	3.36	0.002765561	2.56	0.010294969	1.298822666
Hex2Cer(m40:0+O)	33877165.38	12293392.76	2.76	1.46	0.01342982	1.87	0.032152569	1.041756832
Hex2Cer(m41:0+O)	12344602.35	3133528.94	3.94	1.98	0.017225494	1.76	0.038450326	1.10151261
LPC(20:0)	1486519.22	524258.97	2.84	1.5	0.003538656	2.45	0.012239941	1.163775994
PC(20:0_18:3)	926132.78	382892.22	2.42	1.27	0.011030609	1.96	0.027655799	1.142836797
PC(31:1)	80180171.08	15938725.87	5.03	2.33	0.000978991	3.01	0.005360305	1.351753441
PC(33:4)	80180171.08	15938725.87	5.03	2.33	0.000978991	3.01	0.005360305	1.351753441
PE(15:0_16:0)	978398.53	244032.09	4.01	2	0.017160517	1.77	0.038450326	1.115393584
PE(16:0_12:0)	7859105.51	1711571.74	4.59	2.2	0.007264393	2.14	0.020813177	1.211634833
PE(16:0_18:3)	7536840.22	2830737.56	2.66	1.41	0.01160847	1.94	0.028867499	1.154849992
PE(16:0_22:6)	2824586.59	989877.84	2.85	1.51	0.037316425	1.43	0.07064086	1.000384177
PE(18:0_21:1)	683455.73	264742.17	2.58	1.37	0.020939308	1.68	0.044681812	1.062165337
PE(18:0_22:6)	3096017.11	995687.4	3.11	1.64	0.008121099	2.09	0.022434076	1.195456047
PE(18:1_12:0)	5752927.67	1683683.23	3.42	1.77	0.020287776	1.69	0.04384314	1.100070698
PE(18:1_18:3)	81096367.79	29322778.44	2.77	1.47	0.015790262	1.8	0.036445955	1.127654669
PE(18:1_22:6)	7681272.82	626191.99	12.27	3.62	0.002067277	2.68	0.008330514	1.325354669
PE(32:1)	1485707.99	185751.17	8	3	0.00377699	2.42	0.012780926	1.281570529
PE(34:4)	1485707.99	185751.17	8	3	0.00377699	2.42	0.012780926	1.281570529
PE(38:8e)	6649650.96	1707533.88	3.89	1.96	0.001220736	2.91	0.005986018	1.355861846
PI(18:0_18:0)	992250.76	309679.24	3.2	1.68	0.024823011	1.61	0.050430776	1.049215585
PS(18:0_22:6)	17553295.68	8169276.47	2.15	1.1	0.033099045	1.48	0.06404744	1.019664247

Accession	YC_Mean	CC_Mean	FC	log2FC	P.value	-log10(P.value)	FDR	VIP
TG(10:0_10:0_11:2)	10229836.44	1507390.28	6.79	2.76	0.020266157	1.69	0.04384314	1.078664801
TG(10:0_12:0_12:0)	1470848021	646374242.2	2.28	1.19	0.002436462	2.61	0.009535	1.311386417
TG(10:0_12:2_12:3)	305877347.2	39397806.38	7.76	2.96	0.002052756	2.69	0.008299389	1.320441233
TG(10:0_18:1_18:3)	421267700.3	176370314.6	2.39	1.26	0.006471364	2.19	0.018903195	1.206990448
TG(11:0_18:2_18:2)	28740786.85	11364090.37	2.53	1.34	0.02171951	1.66	0.045961043	1.068381123
TG(11:0_8:0_12:3)	145447284.4	26043745.72	5.58	2.48	0.000259666	3.59	0.002476967	1.408059002
TG(15:0_10:0_16:0)	3642918631	1371051436	2.66	1.41	0.001660628	2.78	0.00725104	1.322067327
TG(15:0_12:1_18:1)	557571781.7	268595177	2.08	1.05	0.002618182	2.58	0.009990002	1.185404389
TG(15:0_18:2_18:2)	505700484.4	226802113.8	2.23	1.16	0.004758572	2.32	0.015249911	1.217406775
TG(15:0_6:0_14:3)	211736502.2	78559628.49	2.7	1.43	0.001677357	2.78	0.007288443	1.332732638
TG(15:0_6:0_18:1)	2385014756	832811716.3	2.86	1.52	0.002664529	2.57	0.010083296	1.184257333
TG(15:0_6:0_6:0)	38524989.32	3773326.37	10.21	3.35	0.018432735	1.73	0.040625216	1.107109706
TG(15:0_6:0_8:0)	358541105.6	53072525.62	6.76	2.76	4.9648E-06	5.3	0.000252584	1.42594109
TG(15:0_8:0_20:3)	163378148.4	35743106.64	4.57	2.19	0.003841229	2.42	0.012920497	1.256383683
TG(16:0_10:0_22:6)	26151856.93	2020535.77	12.94	3.69	0.000837197	3.08	0.004821779	1.377765748
TG(16:0_6:0_10:0)	5712713304	2344093429	2.44	1.29	0.006143755	2.21	0.018163499	1.195600564
TG(16:0_8:0_14:0)	18804189157	9088082983	2.07	1.05	0.006349993	2.2	0.01860978	1.115251044
TG(16:1_10:0_10:3)	347704891.1	148984529.2	2.33	1.22	0.004887627	2.31	0.015445809	1.250059734
TG(16:1_6:0_6:0)	147555717.7	47921245.33	3.08	1.62	0.000269547	3.57	0.002521614	1.390139738
TG(16:1_8:0_10:0)	347704891.1	148984529.2	2.33	1.22	0.004887627	2.31	0.015445809	1.250059734
TG(17:0_6:0_14:3)	65402344.99	17331514.17	3.77	1.92	0.000391364	3.41	0.003024402	1.391055269
TG(17:0_8:0_18:1)	2137419182	756101329.7	2.83	1.5	0.000142592	3.85	0.001758634	1.337061683
TG(18:2_12:3_18:2)	70049690.7	27330644.41	2.56	1.36	0.000324247	3.49	0.002768572	1.398089449
TG(18:3_11:1_18:2)	29047513.67	9987754.57	2.91	1.54	0.018574243	1.73	0.040716609	1.101288769

Accession	YC_Mean	CC_Mean	FC	log2FC	P.value	-log10(P.value)	FDR	VIP
TG(18:4_6:0_9:0)	69461618.68	14605964.61	4.76	2.25	6.29564E-06	5.2	0.000256233	1.421441463
TG(19:0_6:0_6:0)	462193173.3	60407983.95	7.65	2.94	2.46259E-05	4.61	0.000531116	1.391650431
TG(19:1_10:1_18:1)	187779375.2	76932796.02	2.44	1.29	0.002870953	2.54	0.010526324	1.178675697
TG(19:1_6:0_14:3)	33185793.45	2825595.82	11.74	3.55	0.001416846	2.85	0.006552915	1.329318744
TG(19:1_6:0_18:1)	589368479	171791673.6	3.43	1.78	0.000418003	3.38	0.003150506	1.292435796
TG(19:1_6:0_20:5)	29930244.28	7447289.4	4.02	2.01	0.01534939	1.81	0.035630429	1.139138774
TG(19:1_8:0_16:1)	29344352.25	11736204.21	2.5	1.32	0.010330854	1.99	0.02666802	1.149294304
TG(20:0_10:3_10:3)	219049039.8	105894682.4	2.07	1.05	0.003935366	2.41	0.013122767	1.155835837
TG(20:1_6:0_6:0)	840714032.8	276839009.9	3.04	1.6	0.004578781	2.34	0.014751165	1.263272484
TG(26:0_6:0_8:0)	25808774015	11710432933	2.2	1.14	0.019126716	1.72	0.041812232	1.109705375
TG(27:1)	3938978.36	1178407.3	3.34	1.74	0.013628117	1.87	0.032436512	1.140437034
TG(29:0_6:0_8:0)	15512865.82	6808541.59	2.28	1.19	0.036231019	1.44	0.069447527	1.006309358
TG(29:2)	4337947.25	150972.82	28.73	4.84	6.58186E-07	6.18	7.77996E-05	1.455898701
TG(31:0)	296267836.4	73327846.56	4.04	2.01	0.000188482	3.72	0.001995907	1.32670637
TG(33:1)	141263403.3	34252161.21	4.12	2.04	0.000295156	3.53	0.002592704	1.30776298
TG(34:3)	16792846.46	5260916.67	3.19	1.67	0.000141593	3.85	0.001758634	1.337455517
TG(38:3)	23005277.74	2871647.61	8.01	3	0.001004333	3	0.005385616	1.336194257
TG(44:6)	66369543.71	18669591.95	3.55	1.83	0.000137437	3.86	0.001748029	1.338673258
TG(4:0_10:0_16:0)	4095513.63	1361608.09	3.01	1.59	0.022517718	1.65	0.047078996	1.057914719
TG(4:0_10:0_18:1)	291563080	78535810.22	3.71	1.89	0.002675666	2.57	0.010083296	1.310031863
TG(4:0_10:1_18:3)	4712101.61	721303.62	6.53	2.71	0.007425542	2.13	0.020987083	1.208172188
TG(4:0_10:3_17:1)	11254628.8	1809926.4	6.22	2.64	0.000197369	3.7	0.002059722	1.324688
TG(4:0_12:0_17:0)	114653367.9	41287447.62	2.78	1.47	7.74026E-05	4.11	0.001204251	1.358438715
TG(4:0_12:0_18:2)	8609300.31	3110767.49	2.77	1.47	0.00129258	2.89	0.006164999	1.231094265

Accession	YC_Mean	CC_Mean	FC	log2FC	P.value	-log10(P.value)	FDR	VIP
TG(4:0_12:0_20:3)	1470848021	646374242.2	2.28	1.19	0.002436462	2.61	0.009535	1.311386417
TG(4:0_12:3_16:0)	68163431.28	4532056.94	15.04	3.91	0.000834272	3.08	0.004821779	1.368272294
TG(4:0_13:0_18:2)	55533181.79	11278620.91	4.92	2.3	0.022303411	1.65	0.047033618	1.064583076
TG(4:0_13:0_20:3)	85781323.35	15466636.7	5.55	2.47	0.004282899	2.37	0.014019893	1.253512257
TG(4:0_13:0_20:5)	28987679.77	8592326.71	3.37	1.75	0.023476335	1.63	0.048442068	1.075025706
TG(4:0_14:0_17:0)	346855978.6	151364982.5	2.29	1.2	0.003320163	2.48	0.011819007	1.267470805
TG(4:0_14:0_17:1)	63668742.59	26585241.47	2.39	1.26	0.002076134	2.68	0.008338683	1.309912439
TG(4:0_14:0_18:3)	72493777.39	14977785.37	4.84	2.28	0.00166281	2.78	0.00725104	1.324865179
TG(4:0_14:0_20:4)	55544728.19	19682530.02	2.82	1.5	0.0001242	3.91	0.00162647	1.342386805
TG(4:0_14:0_20:5)	17634452.88	3903649.83	4.52	2.18	0.022420878	1.65	0.047037614	1.075401291
TG(4:0_14:0_22:6)	11195925.71	1374665.01	8.14	3.03	0.000150753	3.82	0.001785781	1.335173167
TG(4:0_14:1_17:0)	257451998.6	115760425.1	2.22	1.15	0.013902343	1.86	0.032956652	1.036266056
TG(4:0_14:1_18:1)	64448685.76	21068353.53	3.06	1.61	0.001835167	2.74	0.007647139	1.292029997
TG(4:0_14:2_18:1)	13955492.36	3107608.32	4.49	2.17	0.002908444	2.54	0.010563193	1.262360746
TG(4:0_14:2_20:4)	72493777.39	14977785.37	4.84	2.28	0.00166281	2.78	0.00725104	1.324865179
TG(4:0_14:3_16:0)	738975590.1	239315128.2	3.09	1.63	0.000271239	3.57	0.002521614	1.311257475
TG(4:0_14:3_18:2)	242701402.4	107830239.9	2.25	1.17	0.010646614	1.97	0.027252654	1.172475201
TG(4:0_15:0_18:4)	65723217.31	26001747.05	2.53	1.34	0.00126494	2.9	0.006080677	1.339051451
TG(4:0_16:0_17:1)	2564115562	1059593292	2.42	1.27	0.012273666	1.91	0.030092664	1.134446559
TG(4:0_16:0_20:5)	98743909.03	46255306.42	2.13	1.09	0.016500538	1.78	0.037728759	1.019249522
TG(4:0_16:0_22:6)	98002384.11	16983656.69	5.77	2.53	0.000157072	3.8	0.001802439	1.333692943
TG(4:0_16:1_17:1)	648537478.5	131525556.9	4.93	2.3	0.000967023	3.01	0.005360305	1.333283025
TG(4:0_16:1_18:3)	32861370.55	8292489.22	3.96	1.99	0.003701039	2.43	0.012658177	1.26948039
TG(4:0_17:0_18:4)	36066453.41	5181722.37	6.96	2.8	0.005121698	2.29	0.015679318	1.234196462

Accession	YC_Mean	CC_Mean	FC	log2FC	P.value	-log10(P.value)	FDR	VIP
TG(4:0_18:1_18:3)	965138773.7	394599441.6	2.45	1.29	0.015470369	1.81	0.035843114	1.026915284
TG(4:0_18:1_22:6)	12304138.16	2808758.8	4.38	2.13	0.000640698	3.19	0.004095768	1.271511923
TG(4:0_18:2_18:2)	204607297.3	86769589.9	2.36	1.24	0.0078111	2.11	0.02177478	1.21223081
TG(4:0_18:2_18:3)	189158011.5	63873373.79	2.96	1.57	0.001772412	2.75	0.00750758	1.211560073
TG(4:0_18:2_20:5)	204607297.3	86406169.72	2.37	1.24	0.007610142	2.12	0.021360881	1.214912362
TG(4:0_18:3_18:3)	769637916.3	140663382	5.47	2.45	0.000151772	3.82	0.001785781	1.447179182
TG(4:0_18:3_18:4)	52775986.71	13623096.65	3.87	1.95	0.000125216	3.9	0.00162647	1.341415561
TG(4:0_18:3_20:5)	99041476	30437567.37	3.25	1.7	0.005719839	2.24	0.01703396	1.226199203
TG(4:0_6:0_14:1)	10521461.75	2800559.62	3.76	1.91	0.004963489	2.3	0.015539538	1.226509478
TG(4:0_6:0_16:1)	26382571.14	4775889.62	5.52	2.47	0.002915476	2.54	0.010563193	1.283948783
TG(4:0_6:0_18:2)	30425156.77	6946363.54	4.38	2.13	0.000804997	3.09	0.004702876	1.259511916
TG(4:0_6:0_18:3)	20675955.88	2182463.53	9.47	3.24	0.00286551	2.54	0.010526324	1.295565401
TG(4:0_6:0_19:1)	24128693.71	3258493.9	7.4	2.89	0.0002738	3.56	0.002521614	1.311839602
TG(4:0_6:0_20:0)	127761906.8	48406059.54	2.64	1.4	0.000231269	3.64	0.002277254	1.318382506
TG(4:0_6:0_20:5)	14887721.8	3201395.1	4.65	2.22	0.000340436	3.47	0.002842642	1.301930934
TG(4:0_6:0_21:0)	148128830.3	52443390.56	2.82	1.5	0.000402474	3.4	0.00308259	1.294497849
TG(4:0_6:0_22:1)	807708751.8	285614443.4	2.83	1.5	0.013932715	1.86	0.032956652	1.123135945
TG(4:0_6:0_22:2)	352126619.9	37361532.7	9.42	3.24	0.000629253	3.2	0.004043776	1.383357964
TG(4:0_8:0_18:1)	1130079430	259946796.7	4.35	2.12	3.90896E-05	4.41	0.000738632	1.378931917
TG(4:0_8:0_18:2)	124461726.7	3868823.42	32.17	5.01	2.08101E-05	4.68	0.000488637	1.39565378
TG(4:0_8:0_20:4)	390032966.5	134762937.7	2.89	1.53	0.001064963	2.97	0.005440667	1.350830938
TG(4:0_9:0_22:0)	9017252.02	1827404.63	4.93	2.3	0.001410432	2.85	0.006548052	1.32648107
TG(6:0_10:0_10:0)	86771760.75	20888335.05	4.15	2.05	0.000451308	3.35	0.00320412	1.288742637
TG(6:0_10:1_10:1)	11630204.18	3290480.61	3.53	1.82	0.008768541	2.06	0.023801062	1.1697759

Accession	YC_Mean	CC_Mean	FC	log2FC	P.value	-log10(P.value)	FDR	VIP
TG(6:0_12:0_12:3)	1064525377	131158261.4	8.12	3.02	8.91199E-05	4.05	0.001311029	1.448103987
TG(6:0_13:0_14:3)	130316447.8	39404952.03	3.31	1.73	0.00235197	2.63	0.009263727	1.300002224
TG(6:0_17:1_18:1)	838477933.2	316665246.7	2.65	1.4	0.007215955	2.14	0.020731013	1.190188346
TG(6:0_17:1_18:2)	12817785.18	5095837.15	2.52	1.33	0.030103521	1.52	0.059093889	1.00555144
TG(6:0_17:1_18:3)	16954471.11	2863895	5.92	2.57	2.0352E-05	4.69	0.000487251	1.395780294
TG(6:0_18:1_18:2)	2236437594	1048721956	2.13	1.09	0.013405991	1.87	0.032152569	1.141548597
TG(6:0_18:1_18:3)	844666460.9	224234168.7	3.77	1.91	0.003118348	2.51	0.011198536	1.269802605
TG(6:0_6:0_10:0)	14863710.6	1047704.7	14.19	3.83	6.7672E-05	4.17	0.001131884	1.432956191
TG(6:0_6:0_12:0)	103459105.4	15993197.8	6.47	2.69	4.192E-07	6.38	7.11336E-05	1.46084532
TG(6:0_6:0_13:0)	20651912.49	2622382.04	7.88	2.98	0.000371039	3.43	0.002941808	1.388162171
TG(6:0_6:0_14:1)	26758156.84	8901534.08	3.01	1.59	0.005354762	2.27	0.016223734	1.217036224
TG(6:0_6:0_14:3)	3688120.51	320191.56	11.52	3.53	0.000964243	3.02	0.005360305	1.353451029
TG(6:0_6:0_17:1)	4290523.13	326335.56	13.15	3.72	0.00051551	3.29	0.003495686	1.371922299
TG(6:0_6:0_18:2)	311994997.4	43092447.45	7.24	2.86	8.71647E-05	4.06	0.001297904	1.354603684
TG(6:0_6:0_18:3)	90934947.17	5935836.1	15.32	3.94	0.008825792	2.05	0.023801062	1.196917064
TG(6:0_6:0_20:2)	418201723.5	88209082.47	4.74	2.25	0.001069954	2.97	0.005443393	1.244585511
TG(6:0_6:0_20:5)	116216896.3	14300590.4	8.13	3.02	0.00042113	3.38	0.003154597	1.402855439
TG(6:0_6:0_21:1)	35577108.24	10957517.58	3.25	1.7	0.013072036	1.88	0.031543391	1.151222858
TG(6:0_6:0_22:6)	109105280.4	5157302.97	21.16	4.4	0.000509719	3.29	0.003495686	1.389671288
TG(6:0_8:0_10:1)	3686160.96	707653.86	5.21	2.38	2.23772E-05	4.65	0.000508816	1.393157251
TG(6:0_8:0_14:0)	1360068997	127380455.3	10.68	3.42	3.4044E-07	6.47	7.11336E-05	1.463275381
TG(6:0_8:0_17:1)	131179999	17475717.81	7.51	2.91	2.52291E-05	4.6	0.000531116	1.390814554
TG(6:0_8:0_20:4)	6985199.2	1321335.75	5.29	2.4	0.002574299	2.59	0.009915519	1.267691379
TG(6:0_8:0_20:5)	9790253.62	544530.39	17.98	4.17	0.002881858	2.54	0.010526324	1.297572263

Accession	YC_Mean	CC_Mean	FC	log2FC	P.value	-log10(P.value)	FDR	VIP
TG(6:0_8:0_21:0)	2146270815	749312139.2	2.86	1.52	0.000254312	3.59	0.002476967	1.314616313
TG(6:0_8:0_22:6)	71567950.33	7117747.76	10.05	3.33	0.003019163	2.52	0.010906503	1.293051831
TG(6:0_8:0_8:0)	14641208.51	412696.85	35.48	5.15	2.05181E-07	6.69	7.11336E-05	1.468076447
TG(8:0_10:0_10:0)	156596750.4	40764761.62	3.84	1.94	0.005688458	2.25	0.016990399	1.245643359
TG(8:0_10:3_17:1)	35577108.24	10957517.58	3.25	1.7	0.013072036	1.88	0.031543391	1.151222858
TG(8:0_12:0_12:3)	818604285.1	241095059.9	3.4	1.76	0.00199581	2.7	0.008150114	1.321537691
TG(8:0_17:1_18:3)	19249913.54	5996280.82	3.21	1.68	0.000201936	3.69	0.002089528	1.323273897
TG(8:0_18:1_18:3)	346465845.5	85187531.74	4.07	2.02	0.007402249	2.13	0.020987083	1.213818044
TG(8:0_8:0_10:0)	621710186.7	60321840.24	10.31	3.37	5.24326E-07	6.28	7.11336E-05	1.458516492
TG(8:0_8:0_11:1)	14987172.71	1425736.16	10.51	3.39	0.003535393	2.45	0.012239941	1.273469824
TG(8:0_8:0_14:1)	33727178.06	11196090.54	3.01	1.59	0.005273911	2.28	0.016058468	1.131571958
TG(8:0_8:0_18:3)	129152892.4	10993514.99	11.75	3.55	0.001537114	2.81	0.006977011	1.324048613
TG(8:0_8:0_20:3)	90596298.89	23628793.19	3.83	1.94	0.007751166	2.11	0.021657147	1.217999306
TG(8:0_8:0_8:0)	131195430.1	13355289.67	9.82	3.3	1.41378E-06	5.85	0.000130196	1.445886348
TG(8:0_8:0_9:0)	18467762.07	6638527.54	2.78	1.48	0.013849035	1.86	0.032898194	1.037699459
TG(9:0_18:1_18:2)	136406838	37914987.92	3.6	1.85	0.004711157	2.33	0.01513769	1.230415006
TG(9:0_9:0_9:0)	212532845.6	30447745.39	6.98	2.8	9.30972E-06	5.03	0.000322273	1.41369273
ZyE(32:6)	18198238.88	5257768.17	3.46	1.79	0.012032537	1.92	0.02974034	1.055107684
BisMePA(38:8e)	934832.2	5255638.48	0.18	-2.49	0.018470627	1.73	0.04063538	1.007116122
CL(70:2)	7625084.05	40398888.47	0.19	-2.41	0.00883037	2.05	0.023801062	1.165953529
CL(74:2)	5686276.66	48869471.7	0.12	-3.1	0.001457767	2.84	0.006691478	1.225107204
CL(74:5)	2171757.39	8714002.82	0.25	-2	0.018553664	1.73	0.040716609	1.073599828
CL(78:11)	728732.39	3363550.81	0.22	-2.21	0.008503903	2.07	0.023280865	1.088425118
CL(80:5)	2926558.02	33744057.87	0.09	-3.53	0.001840315	2.74	0.007647139	1.210619408

Accession	YC_Mean	CC_Mean	FC	log2FC	P.value	-log10(P.value)	FDR	VIP
CL(80:7)	447067.04	9131790.77	0.05	-4.35	0.000157953	3.8	0.001802439	1.332708318
CarE(18:0)	558482.52	76123584.84	0.01	-7.09	0.000213099	3.67	0.002168285	1.409133999
CarE(20:0)	77308.08	8802264.08	0.01	-6.83	1.01075E-05	5	0.000322273	1.412257801
Cer(d16:1_16:0)	967454.62	12807575.57	0.08	-3.73	0.001584181	2.8	0.007111344	1.327799442
Cer(d16:1_22:0)	10206423.87	29745821.19	0.34	-1.54	0.014989773	1.82	0.034861928	1.096839683
Cer(d16:1_24:1)	1265800.26	6366307.06	0.2	-2.33	0.020968614	1.68	0.044681812	1.076370585
Cer(d17:1_16:0)	816709.71	4788565.2	0.17	-2.55	0.012654682	1.9	0.030838981	1.142072975
Cer(d18:1_16:0)	9257030.37	69447870.21	0.13	-2.91	0.006312971	2.2	0.018575798	1.219772165
Cer(d18:1_18:0)	978799.97	15118995.66	0.06	-3.95	0.001320882	2.88	0.006251149	1.344746903
Cer(d18:1_24:1)	4132148.46	22767399.13	0.18	-2.46	0.011841867	1.93	0.029328437	1.149793505
Cer(d18:1_24:2)	306855.07	5139462.45	0.06	-4.07	0.001369652	2.86	0.006407452	1.228401618
Cer(d20:1_24:0)	466931	1481220.05	0.32	-1.67	0.009635407	2.02	0.02519236	1.154179795
Cer(d34:2)	218058.06	3259154.04	0.07	-3.9	0.002010164	2.7	0.008181366	1.296326128
Cer(d44:2+O)	56929098.91	282805440.3	0.2	-2.31	0.003425865	2.47	0.012089541	1.165553835
Cer(m19:0_22:0)	5937836.74	13550303.19	0.44	-1.19	0.010066384	2	0.026134657	1.072349564
Cer(m30:1)	1998084.89	9104606.75	0.22	-2.19	0.017662693	1.75	0.039140015	1.010891805
Cer(m37:0)	6278557.45	19856981.73	0.32	-1.66	0.013954619	1.86	0.032956652	1.037713847
Cer(t18:1_26:0+O)	650895.02	2015743.52	0.32	-1.63	0.02247219	1.65	0.047064399	1.062801894
Cer(t44:2)	1141897.86	3456822.6	0.33	-1.6	0.004840826	2.32	0.015432503	1.137613921
ChE()	1015009.67	4491074.77	0.23	-2.15	0.009320949	2.03	0.024687373	1.080800957
DG(18:1_18:1)	35013863.46	170607048	0.21	-2.28	9.8143E-05	4.01	0.001361734	1.350867751
DG(26:3e)	6750097.23	22212257.59	0.3	-1.72	0.01703788	1.77	0.038364472	1.0911791
DG(32:3)	1508223.94	4699292.34	0.32	-1.64	0.016979346	1.77	0.038364472	1.082626381
DG(34:4e)	9653296.37	32293349.05	0.3	-1.74	0.02360115	1.63	0.048442068	1.042360969

Accession	YC_Mean	CC_Mean	FC	log2FC	P.value	-log10(P.value)	FDR	VIP
DG(36:3)	12157423.37	27727576.88	0.44	-1.19	0.021439964	1.67	0.045448257	1.053432774
DG(38:1)	1867632.55	7842387.56	0.24	-2.07	0.027428763	1.56	0.054869737	1.016263774
DG(38:4)	1702362.8	9687810.54	0.18	-2.51	1.55324E-05	4.81	0.000412283	1.403187907
DG(52:1)	22885684.65	111364388.5	0.21	-2.28	0.001614267	2.79	0.007193503	1.325934508
DG(52:2)	18595634.97	101903217.5	0.18	-2.45	9.4825E-05	4.02	0.001331409	1.351949002
DGDG(30:5)	497131.32	2124966.64	0.23	-2.1	0.031622047	1.5	0.061383973	1.016214024
GD3(d42:1)	92834.01	2562346.14	0.04	-4.79	0.001728919	2.76	0.007407051	1.213354159
GM3(d40:1)	8511.09	2399552.74	0	-8.14	7.00898E-07	6.15	7.77996E-05	1.455336431
GM3(d41:1)	196779.31	1670619.08	0.12	-3.09	0.008195421	2.09	0.022555007	1.089446847
GM3(d42:1)	3619.94	2399428.27	0	-9.37	5.47015E-06	5.26	0.000256233	1.481744549
Hex1Cer(d15:0_18:1)	343917.79	3236985.69	0.11	-3.23	0.006913079	2.16	0.020002059	1.217956014
Hex1Cer(d16:0_22:4)	680980.68	17074105.51	0.04	-4.65	8.36993E-05	4.08	0.001261689	1.436434609
Hex1Cer(d18:1_16:0)	6229657.07	98334161.15	0.06	-3.98	0.001092414	2.96	0.005511723	1.35779761
Hex1Cer(d32:0)	1639333.81	12561766.87	0.13	-2.94	0.005343054	2.27	0.016223734	1.243038371
Hex1Cer(d32:1)	131711.55	5034513.62	0.03	-5.26	0.000388851	3.41	0.00302412	1.397960681
Hex1Cer(d35:1)	94077.23	586824.46	0.16	-2.64	0.007287183	2.14	0.020813177	1.103692962
Hex1Cer(d36:1)	384673.99	17390430.03	0.02	-5.5	0.000204079	3.69	0.002093953	1.422351639
Hex1Cer(d36:2)	6130.9	542469.37	0.01	-6.47	0.000518198	3.29	0.003495686	1.381609068
Hex1Cer(d36:3)	2093711.94	52156615.38	0.04	-4.64	1.02937E-05	4.99	0.000322273	1.411641292
Hex1Cer(d37:1)	14863.16	268589.39	0.06	-4.18	0.016968356	1.77	0.038364472	1.01538979
Hex1Cer(d38:1)	2739929.66	24436503.2	0.11	-3.16	0.002710475	2.57	0.010183047	1.303483468
Hex1Cer(d40:2)	131707.07	2723160.36	0.05	-4.37	0.000333409	3.48	0.002827031	1.30254121
Hex1Cer(d42:0+O)	1740844.8	17955661.62	0.1	-3.37	0.00065338	3.18	0.004147448	1.271876648
Hex1Cer(t18:0_20:4)	73409.75	5388953.26	0.01	-6.2	3.57085E-06	5.45	0.000218001	1.43197279

Accession	YC_Mean	CC_Mean	FC	log2FC	P.value	-log10(P.value)	FDR	VIP
Hex1Cer(t20:0_20:4)	23255	1501419.39	0.02	-6.01	0.000132886	3.88	0.001707939	1.339590665
Hex1Cer(t36:0)	1661796.23	30838042.74	0.05	-4.21	0.00108927	2.96	0.005511723	1.355101854
Hex1Cer(t36:1)	142912.39	1531636.38	0.09	-3.42	0.001525283	2.82	0.006949145	1.314837618
Hex1Cer(t38:1)	1316469.63	5764395.82	0.23	-2.13	0.009708687	2.01	0.025329716	1.168230306
Hex2Cer(t34:0)	231776.14	1550854.04	0.15	-2.74	0.005045539	2.3	0.01563605	1.135959817
LBPA(16:0_18:0)	58693.01	422354.77	0.14	-2.85	0.010929193	1.96	0.027655799	1.063096624
LPC(16:0e)	977671.3	24448650.32	0.04	-4.64	0.003614605	2.44	0.012467325	1.270877064
LPC(18:0e)	381612.98	32304068.16	0.01	-6.4	0.00266551	2.57	0.010083296	1.278540199
LPC(18:1)	2197292.43	12211813.73	0.18	-2.47	0.01314722	1.88	0.031662239	1.139708046
LPC(18:1e)	463964.6	28192639.74	0.02	-5.93	0.00510519	2.29	0.015679318	1.250378061
LPC(18:2)	6860926.18	299705736.9	0.02	-5.45	0.000441816	3.35	0.00320412	1.402185194
LPC(20:1)	316057.41	5097160.59	0.06	-4.01	0.004797567	2.32	0.015334633	1.237243504
LPC(20:3)	139529.88	45964058.79	0	-8.36	4.56582E-07	6.34	7.11336E-05	1.460149285
LPC(20:4)	2197292.43	13357309.31	0.16	-2.6	0.012383487	1.91	0.030240476	1.148082231
LPC(30:0)	1041026.96	25624912.79	0.04	-4.62	1.89118E-05	4.72	0.000471251	1.397768428
LPC(32:1)	632204.61	44364935.48	0.01	-6.13	1.70609E-06	5.77	0.000130196	1.443086481
LPE(18:0)	41281.4	8976335.76	0	-7.76	3.08996E-07	6.51	7.11336E-05	1.464228847
LPE(20:2)	1153530.83	4848814.46	0.24	-2.07	0.016549613	1.78	0.037770239	1.083411414
LPE(20:3)	30788.8	3538444.73	0.01	-6.84	7.51863E-06	5.12	0.000296137	1.417391188
LPE(20:4)	538754.4	13273660.72	0.04	-4.62	0.000454478	3.34	0.00320762	1.386321306
LdMePE(18:1)	169813.72	1637859.05	0.1	-3.27	0.004913043	2.31	0.015460891	1.243944482
MGDG(16:0_16:0)	346620.99	2649151.72	0.13	-2.93	0.023526565	1.63	0.048442068	1.070362799
MGDG(18:1_20:3)	102218761.2	379019412.2	0.27	-1.89	0.017990547	1.74	0.039794308	1.103053922
MGDG(18:1_22:5)	9881846.91	58739211.51	0.17	-2.57	0.000972154	3.01	0.005360305	1.340647262

Accession	YC_Mean	CC_Mean	FC	log2FC	P.value	-log10(P.value)	FDR	VIP
MGDG(29:0e)	3428992.96	13865276.19	0.25	-2.02	0.021331399	1.67	0.045375676	1.075617103
MGDG(30:0e)	112154709.2	1383331311	0.08	-3.62	0.000555699	3.26	0.003687546	1.383548359
MGDG(31:0e)	4230346.64	42894948.41	0.1	-3.34	0.001338438	2.87	0.006309781	1.337041423
MGDG(32:0e)	11320491.48	261047247	0.04	-4.53	0.00018552	3.73	0.001995907	1.423711111
MGDG(33:0e)	2630872.59	17336838.06	0.15	-2.72	0.000489844	3.31	0.003417711	1.285597087
MGDG(35:1e)	870084.12	4667919.78	0.19	-2.42	0.004559931	2.34	0.0147293	1.264455659
MGDG(36:0e)	152901927	1154325695	0.13	-2.92	0.001440383	2.84	0.006636634	1.336313266
MGDG(36:1e)	9847898.65	106132678.5	0.09	-3.43	0.001912503	2.72	0.007907986	1.320225384
MGDG(36:2e)	87984.65	9090588.59	0.01	-6.69	2.50525E-05	4.6	0.000531116	1.389873935
MGDG(36:3)	55673904.96	238819784.3	0.23	-2.1	0.011011224	1.96	0.027655799	1.159441181
MGDG(37:1e)	11406890.43	45121779.43	0.25	-1.98	0.010685185	1.97	0.027294166	1.158047845
MGDG(38:2e)	1481682.55	39145979	0.04	-4.72	0.000323996	3.49	0.002768572	1.405969401
MGDG(38:3)	18834070.23	70857267.49	0.27	-1.91	0.004465685	2.35	0.014540271	1.2420237
MGDG(38:5)	16094370.11	150000454.3	0.11	-3.22	0.000718355	3.14	0.004429855	1.360174603
MGDG(40:0e)	391857.23	1464872.1	0.27	-1.9	0.009211187	2.04	0.024503535	1.080596906
MGDG(40:1e)	251895.84	5641326.82	0.04	-4.49	1.96257E-05	4.71	0.000479258	1.396821475
MGDG(40:5)	4576786.99	164481221.3	0.03	-5.17	0.003136361	2.5	0.011230196	1.292703022
MGDG(42:3)	280506.56	2255179.5	0.12	-3.01	0.000427001	3.37	0.003159805	1.291545449
MGMG(35:0)	93591310.01	303957644.4	0.31	-1.7	0.015532412	1.81	0.035918703	1.118187871
MGMG(36:0)	156501555.8	1149000560	0.14	-2.88	0.001520773	2.82	0.006949145	1.331949163
PC(12:1e_20:3)	1771991.97	18219971.94	0.1	-3.36	0.000451358	3.35	0.00320412	1.287497354
PC(14:1e_16:0)	1018546.69	16363098.11	0.06	-4.01	0.000685696	3.16	0.00429351	1.357335755
PC(14:1e_20:4)	2628611.23	61076634.83	0.04	-4.54	0.002334153	2.63	0.009223305	1.193825397
PC(16:0_16:0)	122870974.9	498961310.6	0.25	-2.02	0.011420681	1.94	0.028458472	1.153304025

Accession	YC_Mean	CC_Mean	FC	log2FC	P.value	-log10(P.value)	FDR	VIP
PC(16:0_18:1)	221729510.2	815236029.7	0.27	-1.88	0.01706135	1.77	0.038364472	1.09553832
PC(16:0_20:4)	5188761.59	53935466.91	0.1	-3.38	0.003271822	2.49	0.01168098	1.273967798
PC(16:0_22:4)	226530.3	18145908.59	0.01	-6.32	5.26663E-05	4.28	0.000931964	1.371362917
PC(16:0e_16:0)	232585.08	17434239.03	0.01	-6.23	5.98707E-05	4.22	0.001015307	1.440993217
PC(16:0e_18:2)	118755.96	10913299.99	0.01	-6.52	9.48375E-05	4.02	0.001331409	1.443986942
PC(16:1e_18:0)	260172.48	2542432.54	0.1	-3.29	0.001782021	2.75	0.00750758	1.32563074
PC(16:1e_18:1)	742191.72	8030639.83	0.09	-3.44	0.000227331	3.64	0.002256673	1.319199277
PC(17:0_18:2)	8368164.6	28654698.33	0.29	-1.78	0.006355674	2.2	0.01860978	1.114159128
PC(17:0_18:3)	1866137.8	8711014.22	0.21	-2.22	0.000938965	3.03	0.005307758	1.250905442
PC(17:0_20:4)	194438.52	1318977.1	0.15	-2.76	0.007295692	2.14	0.020813177	1.206884913
PC(17:1_16:0)	58687011.66	453627010	0.13	-2.95	0.001627472	2.79	0.007199797	1.297595105
PC(18:0_16:0)	39346491.09	146385692.6	0.27	-1.9	0.004874779	2.31	0.015445809	1.231936933
PC(18:0_18:0)	3916561.37	10957400.05	0.36	-1.48	0.000408323	3.39	0.003096663	1.292877423
PC(18:0_18:2)	42282639.47	355741663.4	0.12	-3.07	4.81823E-05	4.32	0.000878068	1.373115103
PC(18:0_18:3)	50315623.2	222017304.3	0.23	-2.14	0.0037788	2.42	0.012780926	1.157583272
PC(18:0_20:3)	2410248.48	163116292.4	0.01	-6.08	3.70845E-07	6.43	7.11336E-05	1.461902268
PC(18:0_20:4)	5758617.41	54827405.12	0.11	-3.25	0.001000681	3	0.005385616	1.355135485
PC(18:0_22:4)	246611.57	7406596.11	0.03	-4.91	0.000109949	3.96	0.001475246	1.438357223
PC(18:0e_16:0)	196287.77	12942578.78	0.02	-6.04	0.000375865	3.42	0.002960844	1.385584951
PC(18:0e_18:2)	180347.43	18942378.06	0.01	-6.71	0.000441534	3.36	0.00320412	1.407893623
PC(18:0e_20:4)	78079.16	7348213.8	0.01	-6.56	0.000444267	3.35	0.00320412	1.402659269
PC(18:1_18:1)	103898658.5	379462092.1	0.27	-1.87	0.007563334	2.12	0.021278413	1.099091091
PC(18:1_18:2)	76902464.15	384725182.6	0.2	-2.32	0.001245287	2.9	0.006033499	1.23402387
PC(18:1_18:3)	78199.43	2691669.35	0.03	-5.11	0.00079089	3.1	0.004702876	1.259983165

Accession	YC_Mean	CC_Mean	FC	log2FC	P.value	-log10(P.value)	FDR	VIP
PC(18:1_20:3)	2093517.14	57590983.81	0.04	-4.78	8.86869E-06	5.05	0.000322273	1.41439209
PC(18:1_20:4)	12238979.15	66847774.93	0.18	-2.45	0.014235258	1.85	0.033554537	1.103328445
PC(18:1e_18:2)	79430.54	5387488.36	0.01	-6.08	0.000685363	3.16	0.00429351	1.376647517
PC(18:2e_15:0)	742191.72	8030639.83	0.09	-3.44	0.000227331	3.64	0.002256673	1.319199277
PC(20:0_18:2)	10377123.15	58745438.15	0.18	-2.5	0.000368398	3.43	0.002939959	1.297837197
PC(20:3_18:2)	192117.31	3656186.81	0.05	-4.25	3.79498E-06	5.42	0.000220651	1.430615206
PC(20:4e_18:0)	376738.83	15058422.78	0.03	-5.32	2.25029E-05	4.65	0.000508816	1.393633061
PC(30:0e)	1065364.78	25842693.06	0.04	-4.6	1.55073E-05	4.81	0.000412283	1.40256114
PC(31:0)	203748092.9	861805153.6	0.24	-2.08	0.009343383	2.03	0.024693228	1.165135843
PC(31:0e)	2854901.9	37941858.49	0.08	-3.73	0.002498411	2.6	0.009692823	1.30902061
PC(32:0e)	258342.07	33157350.41	0.01	-7	2.25534E-06	5.65	0.000161986	1.439489267
PC(32:2e)	422950.13	6114793.73	0.07	-3.85	0.000529959	3.28	0.003555388	1.367553298
PC(32:3e)	36716.54	7020000.93	0.01	-7.58	1.38736E-06	5.86	0.000130196	1.44609378
PC(32:5e)	682581.22	62884176.55	0.01	-6.53	4.62902E-06	5.33	0.000252584	1.427315495
PC(32:6e)	15928004.1	57814568.08	0.28	-1.86	0.001098762	2.96	0.005520942	1.242589387
PC(33:1e)	8449181.1	29467060.18	0.29	-1.8	0.001250184	2.9	0.006033499	1.233333096
PC(33:2e)	4191641.53	58208909.11	0.07	-3.8	7.80253E-05	4.11	0.001204251	1.358294306
PC(33:4e)	9951258.92	73759891.11	0.13	-2.89	0.000796879	3.1	0.004702876	1.260228055
PC(34:0)	69784729.9	355280800.4	0.2	-2.35	0.000280868	3.55	0.002521614	1.310177763
PC(34:0e)	831453.99	37715129.33	0.02	-5.5	4.99251E-05	4.3	0.000896449	1.37205455
PC(34:1e)	1925104.84	61137950.38	0.03	-4.99	0.001010079	3	0.005385616	1.340694846
PC(34:3e)	18382528.98	231611868.5	0.08	-3.66	0.000278523	3.56	0.002521614	1.394511015
PC(34:4e)	632204.61	44364935.48	0.01	-6.13	1.70609E-06	5.77	0.000130196	1.443086481
PC(35:2e)	11868847.06	42096283.75	0.28	-1.83	0.014825549	1.83	0.034611846	1.13149281

Accession	YC_Mean	CC_Mean	FC	log2FC	P.value	-log10(P.value)	FDR	VIP
PC(35:3)	5594125.63	178032449.5	0.03	-4.99	2.97875E-05	4.53	0.000586981	1.454207168
PC(35:4)	5776261.58	45773165.67	0.13	-2.99	0.004353867	2.36	0.014214096	1.255549535
PC(35:4e)	225746.38	9381321.73	0.02	-5.38	1.48669E-05	4.83	0.000412283	1.40390437
PC(36:2e)	916861.33	32942413.87	0.03	-5.17	0.000431327	3.37	0.003172593	1.289929387
PC(36:3)	69784729.9	355280800.4	0.2	-2.35	0.000280868	3.55	0.002521614	1.310177763
PC(36:4)	398117731.5	1558146766	0.26	-1.97	0.003728698	2.43	0.012717151	1.159147687
PC(36:4e)	7199715.17	164685952.1	0.04	-4.52	0.000255623	3.59	0.002476967	1.313525327
PC(36:5e)	3143246.04	177946803.2	0.02	-5.82	2.85428E-06	5.54	0.000193615	1.435538673
PC(37:3)	4289108.54	44006768.11	0.1	-3.36	0.000164828	3.78	0.001863468	1.331062761
PC(37:5)	4198703.63	21574220.41	0.19	-2.36	0.01267909	1.9	0.030838981	1.047075399
PC(37:5e)	493109.34	27613888.39	0.02	-5.81	1.01145E-05	5	0.000322273	1.412020326
PC(38:4)	254789177.3	912060881.4	0.28	-1.84	0.023870906	1.62	0.0488214	1.040662111
PC(38:4e)	3036815.98	183605085.6	0.02	-5.92	0.000449755	3.35	0.00320412	1.387744937
PC(38:5e)	2294846.6	92012358.75	0.02	-5.33	0.000923553	3.03	0.005244924	1.358562011
PC(38:6e)	4833492.31	67202510.67	0.07	-3.8	0.000351288	3.45	0.002859485	1.299977745
PC(39:4)	1950836.59	7573097.15	0.26	-1.96	0.026313468	1.58	0.053017731	1.036707166
PC(40:6e)	925661.69	10026376.97	0.09	-3.44	0.018209226	1.74	0.04020518	1.107594669
PC(8:0e_24:1)	931379.24	44429930.23	0.02	-5.58	1.08434E-05	4.96	0.000330995	1.410594444
PE(16:0_20:4)	8320765.35	61532136.89	0.14	-2.89	0.008791726	2.06	0.023801062	1.185563737
PE(16:0e_18:1)	8649088.22	71944923.05	0.12	-3.06	0.001036776	2.98	0.005404529	1.244642922
PE(16:1e_16:0)	397427.41	3254297.33	0.12	-3.03	0.001783127	2.75	0.00750758	1.310311033
PE(16:1e_18:1)	16025050.28	76832852.51	0.21	-2.26	0.017524063	1.76	0.038974282	1.090158216
PE(16:1e_18:2)	7685226.5	93950308.4	0.08	-3.61	0.000607129	3.22	0.003964198	1.358127317
PE(16:1e_20:3)	401611.11	3029972.84	0.13	-2.92	0.000335812	3.47	0.002827766	1.303053873

Accession	YC_Mean	CC_Mean	FC	log2FC	P.value	-log10(P.value)	FDR	VIP
PE(16:1e_20:4)	4907495.16	132020036.2	0.04	-4.75	0.000273873	3.56	0.002521614	1.402892568
PE(16:1e_22:5)	4554624.07	43765166.1	0.1	-3.26	0.002592205	2.59	0.00992189	1.288754827
PE(17:0_20:3)	349603.97	1325884.92	0.26	-1.92	0.030800531	1.51	0.060075796	1.008499879
PE(18:0_16:0)	3262207.61	9412351.61	0.35	-1.53	0.025245961	1.6	0.051119932	1.029495196
PE(18:0_18:0)	468844.76	1308187.61	0.36	-1.48	0.001776139	2.75	0.00750758	1.211329482
PE(18:0_18:2)	147957180.4	383369735.4	0.39	-1.37	0.029325941	1.53	0.057753185	1.01690251
PE(18:0_20:3)	1244798.03	53720798.03	0.02	-5.43	2.89442E-05	4.54	0.000586981	1.455170785
PE(18:0_20:4)	14106370.58	141016521.8	0.1	-3.32	0.001026012	2.99	0.005404529	1.338825793
PE(18:0_22:4)	706207.22	14931906.37	0.05	-4.4	0.000111765	3.95	0.001483316	1.427540018
PE(18:0e_18:1)	739318.68	6318792.89	0.12	-3.1	0.006941765	2.16	0.020037577	1.19873593
PE(18:0e_18:2)	252087.41	34798765.58	0.01	-7.11	7.75364E-05	4.11	0.001204251	1.359379397
PE(18:1_18:2)	200533710.5	842860646.8	0.24	-2.07	0.010761032	1.97	0.027379652	1.153953471
PE(18:1_20:3)	660051.41	24090469.02	0.03	-5.19	6.99762E-05	4.16	0.001154608	1.438142517
PE(18:1_20:4)	14015575.4	58510840.39	0.24	-2.06	0.032213624	1.49	0.062433072	1.017865453
PE(18:1e_16:0)	151489.94	2059990.61	0.07	-3.77	0.001577	2.8	0.007105228	1.317023794
PE(18:1e_18:2)	3284635.35	22711442.31	0.14	-2.79	0.001693214	2.77	0.00730535	1.214044914
PE(18:1e_20:3)	332677.85	17320049.07	0.02	-5.7	4.77002E-06	5.32	0.000252584	1.426573034
PE(18:1e_20:4)	3426196.99	67579027.54	0.05	-4.3	0.000920506	3.04	0.005244924	1.349423034
PE(18:1e_20:5)	1458151.66	7379144.48	0.2	-2.34	0.009170066	2.04	0.024500329	1.181391604
PE(18:1e_22:4)	292532.43	8728672.88	0.03	-4.9	0.000286582	3.54	0.002535631	1.402097401
PE(18:2e_18:2)	2033825.1	18643689.44	0.11	-3.2	0.000303422	3.52	0.002646274	1.307091827
PE(19:1_15:0)	30992609.95	211487573.5	0.15	-2.77	0.012093266	1.92	0.029769914	1.142683623
PE(20:3_18:2)	536379.7	5789979.48	0.09	-3.43	5.78883E-05	4.24	0.000995516	1.367539718
PE(20:3e_18:2)	806224.13	8391781.47	0.1	-3.38	9.4867E-05	4.02	0.001331409	1.351682551

Accession	YC_Mean	CC_Mean	FC	log2FC	P.value	-log10(P.value)	FDR	VIP
PE(31:0e)	1269606.17	37382137.35	0.03	-4.88	1.69649E-05	4.77	0.000440726	1.399963704
PE(33:0e)	4463339.63	33329270.57	0.13	-2.9	0.003344146	2.48	0.011838842	1.274101441
PE(33:2e)	6497313.62	39089619.32	0.17	-2.59	0.001204576	2.92	0.005939996	1.235682354
PE(34:0)	200585329.2	861708746.2	0.23	-2.1	0.008991538	2.05	0.024128942	1.172527103
PE(34:0e)	20891978.45	128438399.4	0.16	-2.62	0.001362279	2.87	0.006397472	1.228527561
PE(34:1p)	64076550.04	232540203	0.28	-1.86	0.030631867	1.51	0.059842416	1.028870253
PE(34:2p)	26414224.89	328743586.9	0.08	-3.64	0.001138979	2.94	0.005699563	1.328720744
PE(36:0)	5691446.5	177667600.4	0.03	-4.96	2.84093E-05	4.55	0.000586981	1.45285012
PE(36:3)	23809018.55	116673739.3	0.2	-2.29	0.016184347	1.79	0.037144901	1.11435535
PE(36:3p)	8956212.84	136506498.3	0.07	-3.93	3.93211E-05	4.41	0.000738632	1.378770901
PE(36:4)	30992609.95	211487573.5	0.15	-2.77	0.012093266	1.92	0.029769914	1.142683623
PE(36:5e)	1949311.29	41341440.44	0.05	-4.41	0.000101503	3.99	0.001383013	1.349183058
PE(37:2e)	4512273.38	172597646.1	0.03	-5.26	1.36178E-05	4.87	0.000395889	1.405970431
PE(38:4)	58687011.66	453627010	0.13	-2.95	0.001627472	2.79	0.007199797	1.297595105
PE(38:5e)	1325774.92	25422763.51	0.05	-4.26	5.76489E-05	4.24	0.000995516	1.367580492
PE(40:4p)	493109.34	27613888.39	0.02	-5.81	1.01145E-05	5	0.000322273	1.412020326
PI(18:0_20:4)	9197463.11	38840347.8	0.24	-2.08	0.00266975	2.57	0.010083296	1.184252462
PI(18:0_22:4)	162194.59	2286540.52	0.07	-3.82	5.28966E-06	5.28	0.000256233	1.424880441
PI(18:1_20:4)	1300614.64	4881540.4	0.27	-1.91	0.006860623	2.16	0.019897437	1.108432512
PI(36:1)	6278072.29	27288887.42	0.23	-2.12	0.003510754	2.45	0.012212625	1.249484369
PI(38:4)	6226196.44	27371435.01	0.23	-2.14	0.003437936	2.46	0.012097174	1.254659547
PI(38:5)	2401674.94	9091067.32	0.26	-1.92	0.002742309	2.56	0.010271041	1.18267084
PIP(19:0_18:2)	1118949.7	6479454.19	0.17	-2.53	0.00100774	3	0.005385616	1.247489854
PS(16:0_18:2)	23595466.92	151431525.9	0.16	-2.68	0.005439264	2.26	0.016357984	1.219548246

Accession	YC_Mean	CC_Mean	FC	log2FC	P.value	-log10(P.value)	FDR	VIP
PS(18:0_18:0)	3695449.33	155326993.8	0.02	-5.39	3.11142E-06	5.51	0.00019995	1.434229753
PS(18:0_18:2)	26465282.61	124531058.6	0.21	-2.23	0.003668498	2.44	0.012617566	1.160368759
PS(18:0_20:3)	1294897.62	58092041.32	0.02	-5.49	1.67305E-06	5.78	0.000130196	1.443606603
PS(18:0_22:4)	2534160.1	112863845.9	0.02	-5.48	6.10102E-06	5.21	0.000256233	1.422074278
PS(18:0_22:5)	1531723.84	6829944.93	0.22	-2.16	0.009515451	2.02	0.024932114	1.173613084
PS(18:1_18:1)	30755827.8	137397456.5	0.22	-2.16	0.00057419	3.24	0.003769282	1.276666776
PS(18:1_18:2)	17183811.02	272873369.1	0.06	-3.99	0.000152982	3.82	0.001785781	1.423710041
PS(18:1_20:4)	11077359.12	39763043.11	0.28	-1.84	0.007172957	2.14	0.020656086	1.103928977
PS(18:2_20:4)	1051299.86	6579681.05	0.16	-2.65	0.013006941	1.89	0.031543391	1.143548795
PS(20:3_20:3)	481161.14	12193759.52	0.04	-4.66	0.000138961	3.86	0.001749189	1.413480088
PS(22:0_18:2)	4947550.46	17948189.24	0.28	-1.86	0.01009067	2	0.026134657	1.070823285
PS(22:4_18:2)	1249148.69	13890839.96	0.09	-3.48	0.000655575	3.18	0.004147448	1.373367759
PS(34:0)	17728359.55	274907498.5	0.06	-3.95	0.000189213	3.72	0.001995907	1.414926105
PS(36:1)	6275591.05	37362772.39	0.17	-2.57	0.0048956	2.31	0.015445809	1.2226902
PS(36:2)	122781805.6	432086739	0.28	-1.82	0.001171812	2.93	0.005816189	1.238075214
PS(36:2e)	31696.43	7519262.52	0	-7.89	1.8072E-07	6.74	7.11336E-05	1.469793337
PS(36:5e)	227786.56	3535554.76	0.06	-3.96	0.000183329	3.74	0.001995907	1.407298179
PS(38:4)	5807828.42	37377692.09	0.16	-2.69	0.001283331	2.89	0.006144888	1.232422626
PS(38:5)	3090034.61	16225384.47	0.19	-2.39	0.00513655	2.29	0.015679318	1.218916205
PS(40:7e)	382283.02	8655795.72	0.04	-4.5	0.000811969	3.09	0.004721019	1.362690268
PS(40:8e)	385078.76	4521114.74	0.09	-3.55	0.000567295	3.25	0.00374415	1.278787581
SM(d19:0_19:1)	52456975.57	1131168808	0.05	-4.43	0.000984281	3.01	0.005363444	1.354875406
SM(d19:1_23:0)	28382576.77	113496334.5	0.25	-2	0.029704306	1.53	0.058404118	1.029165973
SM(d28:1)	94429.46	1776068.98	0.05	-4.23	0.002830945	2.55	0.010442853	1.295510801

Accession	YC_Mean	CC_Mean	FC	log2FC	P.value	-log10(P.value)	FDR	VIP
SM(d30:0)	193748.97	1448286.72	0.13	-2.9	0.00076517	3.12	0.004579769	1.355631088
SM(d32:0)	4662740.91	19166853.5	0.24	-2.04	0.009231471	2.03	0.024503535	1.183729596
SM(d32:1)	12859462.23	260433810.8	0.05	-4.34	0.000153225	3.81	0.001785781	1.430113389
SM(d33:1)	13687114.14	116251231.6	0.12	-3.09	0.001917087	2.72	0.007907986	1.312665413
SM(d34:0)	14055350.43	93892597.58	0.15	-2.74	0.005387215	2.27	0.016241456	1.220368207
SM(d34:1)	234145643.4	2403576577	0.1	-3.36	0.000627339	3.2	0.004043776	1.352759787
SM(d34:2)	2997016.8	28155284.09	0.11	-3.23	0.002200179	2.66	0.008750547	1.313456918
SM(d34:4)	12407004.53	504277593.1	0.02	-5.34	7.78118E-05	4.11	0.001204251	1.437095863
SM(d35:1)	4230346.64	43097566.94	0.1	-3.35	0.001242946	2.91	0.006033499	1.341050562
SM(d35:4)	2689337.76	9625298.68	0.28	-1.84	0.020534419	1.69	0.044141771	1.068787657
SM(d36:0)	2645536.91	14404378.16	0.18	-2.44	0.00379006	2.42	0.012783599	1.253253159
SM(d36:1)	10359103.28	258485909	0.04	-4.64	0.000166467	3.78	0.001864732	1.426822646
SM(d36:3)	14055350.43	93892597.58	0.15	-2.74	0.005387215	2.27	0.016241456	1.220368207
SM(d36:4)	234145643.4	2403576577	0.1	-3.36	0.000627339	3.2	0.004043776	1.352759787
SM(d37:1)	26592945.65	166757969.9	0.16	-2.65	0.00387366	2.41	0.01295819	1.264463977
SM(d37:4)	7499223.75	118472972.4	0.06	-3.98	0.000975222	3.01	0.005360305	1.34149935
SM(d38:2)	4363096.89	40435321.32	0.11	-3.21	0.000349122	3.46	0.002859485	1.300881359
SM(d38:3)	5641412.93	38151377.52	0.15	-2.76	0.001240659	2.91	0.006033499	1.314435639
SM(d39:1)	91923045.17	304174821.5	0.3	-1.73	0.014685052	1.83	0.034457224	1.131046073
SM(d39:2)	11191528.23	53232689.37	0.21	-2.25	0.003345127	2.48	0.011838842	1.26223309
SM(d40:1)	153847593.7	1166401819	0.13	-2.92	0.001317348	2.88	0.006251149	1.340931429
SM(d40:2)	13314513.82	234610143.6	0.06	-4.14	0.001040555	2.98	0.005404529	1.339581196
SM(d41:1)	213127124.2	644241671.9	0.33	-1.6	0.024003472	1.62	0.049010434	1.053264474
SM(d41:2)	7512817.17	36255207.11	0.21	-2.27	0.006034838	2.22	0.017928314	1.213506427

Accession	YC_Mean	CC_Mean	FC	log2FC	P.value	-log10(P.value)	FDR	VIP
SM(d42:3)	1754260	40230105.5	0.04	-4.52	0.000388761	3.41	0.00302412	1.397837214
SM(d42:5)	13314513.82	234610143.6	0.06	-4.14	0.001040555	2.98	0.005404529	1.339581196
SM(d43:1)	9460650.68	44435834.93	0.21	-2.23	0.009422326	2.03	0.024794526	1.078922305
SM(d43:2)	3115314.72	14482399	0.22	-2.22	0.009146896	2.04	0.024492017	1.178912528
SQDG(31:1)	392675.54	2772914.47	0.14	-2.82	0.010076787	2	0.026134657	1.164247571
StE(24:7)	45598577.81	229100867.3	0.2	-2.33	0.006313641	2.2	0.018575798	1.192166716
TG(12:1e_10:0_18:3)	1040359	10806595.19	0.1	-3.38	4.26885E-05	4.37	0.000789737	1.376850184
TG(14:0_14:0_20:4)	505350004	1082931350	0.47	-1.1	0.005691297	2.24	0.016990399	1.124751231
TG(14:0e_18:0_18:0)	1681654.32	17475031.11	0.1	-3.38	2.98058E-05	4.53	0.000586981	1.386703086
TG(14:0e_18:1_18:1)	11031852.36	61827981.47	0.18	-2.49	9.34526E-05	4.03	0.001331409	1.352219901
TG(15:0_17:0_18:3)	162407851.2	774136988.2	0.21	-2.25	0.008717802	2.06	0.023759903	1.086107879
TG(16:0_14:0_20:4)	485225399.4	1224693701	0.4	-1.34	0.001841326	2.73	0.007647139	1.209505179
TG(16:0_16:0_20:3)	325939160	891082101	0.37	-1.45	0.017214345	1.76	0.038450326	1.091345369
TG(16:0_17:1_20:5)	320698828.5	852836715.7	0.38	-1.41	0.001690297	2.77	0.00730535	1.214964207
TG(16:0_18:2_20:4)	119191185.1	788379756.8	0.15	-2.73	0.000176254	3.75	0.001956414	1.32970609
TG(16:0_20:2_20:4)	129443867.7	297282850	0.44	-1.2	0.00025953	3.59	0.002476967	1.313184404
TG(16:0_20:4_20:4)	71845757.66	283268115.6	0.25	-1.98	0.000365016	3.44	0.002939959	1.380418306
TG(16:0_8:0_17:0)	677664.76	2628039.15	0.26	-1.96	0.008044482	2.09	0.022272819	1.091784636
TG(16:0e_16:0_20:4)	1887746.14	31379105.54	0.06	-4.06	0.000873912	3.06	0.005009611	1.33969748
TG(16:0e_6:0_20:4)	756688.31	3147846.81	0.24	-2.06	0.00116313	2.93	0.005796659	1.240517795
TG(16:1_17:0_18:3)	370979535	761790574.9	0.49	-1.04	0.000186526	3.73	0.001995907	1.326658169
TG(16:1_18:2_18:3)	38613590.38	102547439.8	0.38	-1.41	0.024130512	1.62	0.049187571	1.062396042
TG(16:1e_16:0_16:0)	10073445.5	96967408.34	0.1	-3.27	0.000344563	3.46	0.002842642	1.404939183
TG(17:0_14:0_18:3)	251875897.4	532611177.4	0.47	-1.08	0.002508544	2.6	0.009692823	1.187194779

Accession	YC_Mean	CC_Mean	FC	log2FC	P.value	-log10(P.value)	FDR	VIP
TG(18:0_14:0_20:5)	407994780.4	1260011767	0.32	-1.63	0.00843987	2.07	0.023157486	1.086509376
TG(18:0_18:0_22:4)	9144913.64	35106170.38	0.26	-1.94	0.01169155	1.93	0.029015005	1.16419229
TG(18:0_18:1_20:3)	87396116.33	726598790.6	0.12	-3.06	6.25853E-06	5.2	0.000256233	1.420676349
TG(18:0_18:1_20:4)	261019720.6	574165725.5	0.45	-1.14	7.57779E-05	4.12	0.001204251	1.359049654
TG(18:0_18:1_22:4)	78311860.54	317324437.9	0.25	-2.02	0.005044579	2.3	0.01563605	1.232273851
TG(18:0_18:2_20:5)	145310153.9	376893313.7	0.39	-1.38	0.000403943	3.39	0.00308259	1.293249407
TG(18:0_20:3_20:5)	27298207.96	119568838.3	0.23	-2.13	0.001206486	2.92	0.005939996	1.354593263
TG(18:0_23:1_24:0)	1463749.07	5507755.46	0.27	-1.91	0.004477596	2.35	0.014540279	1.144950309
TG(18:1_14:3_18:1)	389269071	956847405.5	0.41	-1.3	1.42933E-05	4.84	0.000405864	1.404664078
TG(18:1_18:1_20:3)	504628176.8	1530602833	0.33	-1.6	0.019881014	1.7	0.043270442	1.101306137
TG(18:1_18:1_22:4)	7900838.84	36730992.39	0.22	-2.22	0.001381484	2.86	0.006438139	1.333526067
TG(18:1_18:1_22:6)	36719875.69	110136938.7	0.33	-1.58	0.01472459	1.83	0.034457224	1.032459933
TG(18:1_18:3_22:5)	47415237.3	193727504.1	0.24	-2.03	0.002829284	2.55	0.010442853	1.289883569
TG(18:1e_14:0_14:0)	2878473.24	34712265.89	0.08	-3.59	0.000285624	3.54	0.002535631	1.309534476
TG(18:1e_16:0_16:0)	22854183.79	169413360.3	0.13	-2.89	0.000706962	3.15	0.004381729	1.377503704
TG(18:1e_18:1_18:1)	1297696.57	17823908.47	0.07	-3.78	7.89026E-05	4.1	0.001204251	1.359425239
TG(18:2e_8:0_10:1)	12157423.37	27727576.88	0.44	-1.19	0.021439964	1.67	0.045448257	1.053432774
TG(19:1_18:1_20:3)	21178505.35	52233417.38	0.41	-1.3	0.013599899	1.87	0.032435594	1.121755551
TG(20:0_20:4_20:4)	3227853.25	18901371.3	0.17	-2.55	0.004962562	2.3	0.015539538	1.238073233
TG(20:1e_16:0_16:0)	20164194.67	110515789.7	0.18	-2.45	0.000727615	3.14	0.004430438	1.371345585
TG(20:2e_16:0_16:0)	18649508.64	102326903.3	0.18	-2.46	0.000367584	3.43	0.002939959	1.37591167
TG(20:3e_16:0_16:0)	2372370.42	29268636.06	0.08	-3.62	9.31069E-06	5.03	0.000322273	1.413607728
TG(20:3e_6:0_6:0)	1508223.94	4699292.34	0.32	-1.64	0.016979346	1.77	0.038364472	1.082626381
TG(22:2_14:1_14:1)	2065084.1	7441952.81	0.28	-1.85	0.008201821	2.09	0.022555007	1.090549052

Accession	YC_Mean	CC_Mean	FC	log2FC	P.value	-log10(P.value)	FDR	VIP
TG(24:1_12:2_24:1)	25152537.83	54438281.81	0.46	-1.11	0.017049309	1.77	0.038364472	1.01670836
TG(25:0_18:1_24:0)	459562.79	5579549.86	0.08	-3.6	0.001937309	2.71	0.007937766	1.206023529
TG(25:0_6:0_18:4)	6774963.56	17802513.2	0.38	-1.39	0.001545516	2.81	0.006989166	1.221442653
TG(26:0_6:0_20:4)	19474633.97	730763823.7	0.03	-5.23	0.014731098	1.83	0.034457224	1.128781498
TG(26:1_18:1_18:2)	8280579.71	29046667.55	0.29	-1.81	0.004257015	2.37	0.013972621	1.149474962
TG(27:1_18:1_18:1)	1672828.57	4357687.75	0.38	-1.38	0.010958948	1.96	0.027655799	1.063189329
TG(28:0_18:0_18:1)	7279166.7	21137647.66	0.34	-1.54	0.004073146	2.39	0.013441381	1.152395595
TG(28:1_18:0_18:1)	5790617.63	15761874.75	0.37	-1.44	0.00610102	2.21	0.018080935	1.118888946
TG(28:1_18:1_18:1)	2865508.15	10366638.79	0.28	-1.86	0.007416034	2.13	0.020987083	1.100880884
TG(28:1_6:0_20:3)	671939.6	3146362.74	0.21	-2.23	0.000699039	3.16	0.004354726	1.265339416
TG(29:0_18:1_18:1)	783459.85	3495520.55	0.22	-2.16	0.010763499	1.97	0.027379652	1.065576314
TG(30:0_16:0_18:0)	4176734.6	13088342.58	0.32	-1.65	0.001735001	2.76	0.007407121	1.213214769
TG(30:0_18:0_18:1)	2140917.53	8942796.26	0.24	-2.06	0.026259102	1.58	0.052995642	1.023226867
TG(30:0_18:1_18:1)	1610541.79	7276290.78	0.22	-2.18	0.000732964	3.13	0.004430438	1.26488223
TG(30:1_18:1_18:1)	732036.24	3961386.99	0.18	-2.44	0.009393562	2.03	0.024772222	1.078387208
TG(30:1_19:0_19:0)	645356.77	4022715.37	0.16	-2.64	0.004558188	2.34	0.0147293	1.144095531
TG(38:0e)	969781.22	5818175.5	0.17	-2.58	0.000101942	3.99	0.001383013	1.348948307
TG(38:1e)	1867632.55	7842387.56	0.24	-2.07	0.027428763	1.56	0.054869737	1.016263774
TG(38:4e)	3805547.03	24331399.12	0.16	-2.68	1.16669E-05	4.93	0.000347447	1.408880682
TG(44:1)	96578016.96	289533228.6	0.33	-1.58	0.023606085	1.63	0.048442068	1.040108309
TG(44:4)	851066.87	1720007.78	0.49	-1.02	0.017561754	1.76	0.038987094	1.015127067
TG(46:4)	96578016.96	289533228.6	0.33	-1.58	0.023606085	1.63	0.048442068	1.040108309
TG(48:0e)	7306718.8	107639721.4	0.07	-3.88	1.75538E-05	4.76	0.000446524	1.400460853
TG(4:0_16:1_20:5)	19979041.25	86262653.08	0.23	-2.11	0.010498876	1.98	0.026987638	1.068178553

Accession	YC_Mean	CC_Mean	FC	log2FC	P.value	-log10(P.value)	FDR	VIP
TG(4:0_17:0_18:1)	1896588.89	6484702.31	0.29	-1.77	0.015992695	1.8	0.036843548	1.022731308
TG(4:0_18:0_20:4)	60456767.65	139419140.4	0.43	-1.21	0.002300021	2.64	0.00911794	1.295625571
TG(4:0_18:3_20:4)	47363658	175964471.1	0.27	-1.89	0.001836397	2.74	0.007647139	1.321994847
TG(52:0)	71421200.61	177827803.2	0.4	-1.32	0.003495545	2.46	0.01219446	1.164545351
TG(52:7e)	19859392.03	41038273.66	0.48	-1.05	0.003944353	2.4	0.013122767	1.154756194
TG(54:1e)	4777432.63	15576882.55	0.31	-1.71	0.001610771	2.79	0.007193503	1.327388013
TG(54:5)	70171157.5	170863554.6	0.41	-1.28	0.000502961	3.3	0.00346958	1.283132092
TG(59:3)	27639510.22	57193390.8	0.48	-1.05	0.012328245	1.91	0.030165906	1.052500146
TG(62:1)	481724.6	2011739.36	0.24	-2.06	0.01098049	1.96	0.027655799	1.06248543
ZyE(18:1)	2621852.31	469640028.6	0.01	-7.48	5.1186E-07	6.29	7.11336E-05	1.458545472
ZyE(20:4)	2071909.82	394678329.2	0.01	-7.57	0.030431936	1.52	0.059619603	1.01855501
ZyE(23:1)	3150913.41	44349285.56	0.07	-3.82	0.001053463	2.98	0.005404529	1.243321131
ZyE(26:6)	64185267.11	170257507.7	0.38	-1.41	0.016290293	1.79	0.037317913	1.020114028
ZyE(33:6)	28772998.87	58859678.62	0.49	-1.03	0.002090156	2.68	0.008367476	1.283047487
dMePE(16:0_16:0)	3150739.85	13119464.86	0.24	-2.06	0.009222601	2.04	0.024503535	1.181142618
dMePE(16:0_18:2)	836940.23	8021255.21	0.1	-3.26	0.000149595	3.83	0.001785781	1.335785678
dMePE(16:0_18:3)	45101011.11	210169630.6	0.21	-2.22	0.007394846	2.13	0.020987083	1.19763653
dMePE(18:0_16:0)	831890	3176917.05	0.26	-1.93	0.000746415	3.13	0.004489523	1.263050527
dMePE(18:2_18:2)	14106370.58	141016521.8	0.1	-3.32	0.001026012	2.99	0.005404529	1.338825793
dMePE(36:3)	820042.58	3533115.43	0.23	-2.11	0.008610522	2.06	0.023520015	1.172326809

Table S3. The differential lipids identified between buffalo colostrum (BC) and cow colostrum (CC) (VIP > 1, p value < 0.05, FC > 2 or FC < 0.5).

Accession	BC_Mean	CC_Mean	FC	log2FC	P.value	-log10(P.value)	FDR	VIP
Cer(d18:1_24:0)	706137.33	218742.38	3.23	1.69	0.007087869	2.15	0.058363617	1.452427477
Cer(d19:1_24:0)	9866560.93	2556518.73	3.86	1.95	0.025940801	1.59	0.103508884	1.268094664
Cer(d19:1_24:1)	4886598.96	610261.33	8.01	3	0.003777181	2.42	0.048292441	1.521658331
Cer(d39:0)	3313575.85	1092331.12	3.03	1.6	0.011559447	1.94	0.071774317	1.389902513
DG(18:0e)	37979129.16	10605584.02	3.58	1.84	0.010362691	1.98	0.069455654	1.402624967
DG(18:1e)	9250957.34	2957643.29	3.13	1.65	0.025232457	1.6	0.102617844	1.271794059
DG(20:0e)	1665798034	730634062.5	2.28	1.19	0.002032648	2.69	0.035323552	1.580156945
DG(20:1e)	291566170.2	120633856	2.42	1.27	0.028276712	1.55	0.110369128	1.252016799
DG(24:0e)	57532095.68	12214850.42	4.71	2.24	0.005489555	2.26	0.05132252	1.481076145
DG(24:3e)	43806739.12	7098037.75	6.17	2.63	0.010348215	1.99	0.069455654	1.403544809
DG(25:0e)	2364148.06	436142.04	5.42	2.44	0.025636878	1.59	0.102631568	1.270081247
DG(26:0e)	77937129.91	18445333.7	4.23	2.08	0.014012655	1.85	0.077728723	1.3623574
DG(28:0e)	225653904.7	61052927.94	3.7	1.89	0.007769716	2.11	0.061602746	1.439657654
DG(28:3e)	77969781.87	18502983.64	4.21	2.08	0.014160178	1.85	0.077728723	1.360866096
DG(30:1e)	89806625.52	24829000.86	3.62	1.85	0.00558084	2.25	0.051622767	1.47933173
DG(32:1)	29654731.35	10465104.04	2.83	1.5	0.020444463	1.69	0.093144361	1.30504056
DG(34:0)	23691750.51	10800956.83	2.19	1.13	0.015926963	1.8	0.083106074	1.345800414
DG(34:1)	232361055.6	59180426.65	3.93	1.97	0.001828065	2.74	0.035323552	1.588610634
DG(34:2e)	166436560.1	68959485.61	2.41	1.27	0.010414908	1.98	0.069455654	1.402692015
DG(36:1)	60518336.98	24007585.14	2.52	1.33	0.005063954	2.3	0.051175884	1.490798605

Accession	BC_Mean	CC_Mean	FC	log2FC	P.value	-log10(P.value)	FDR	VIP
DG(36:2)	231072526.8	46450026.98	4.97	2.31	0.000920605	3.04	0.028967109	1.643859475
DG(36:3)	61478137.6	27727576.88	2.22	1.15	0.030919029	1.51	0.116126091	1.236030389
DG(36:4e)	456410606.8	185056702.2	2.47	1.3	0.004792719	2.32	0.051175884	1.496119504
DG(38:1)	24466188.8	7842387.56	3.12	1.64	0.027735062	1.56	0.109240357	1.254675017
Hex1Cer(d35:1)	2198737.35	586824.46	3.75	1.91	0.034286589	1.46	0.119953942	1.218136236
Hex1Cer(d37:1)	3345302.25	268589.39	12.46	3.64	0.006711185	2.17	0.057781244	1.457615786
Hex1Cer(m39:0+O)	2028926.17	917980.35	2.21	1.14	0.043875997	1.36	0.138443067	1.171239885
Hex2Cer(d38:0)	14689933.07	3558330.57	4.13	2.05	0.006927052	2.16	0.057781244	1.455442008
Hex2Cer(d39:0)	7616339.58	1863587.46	4.09	2.03	0.017103214	1.77	0.085938371	1.333930435
Hex2Cer(d40:0+O)	7372313.51	1731532.07	4.26	2.09	0.011423517	1.94	0.071774317	1.392130483
Hex2Cer(d42:2)	1066634.74	235761.11	4.52	2.18	0.012499712	1.9	0.072676899	1.37979188
Hex2Cer(m32:0+O)	1564487.8	537687.52	2.91	1.54	0.024973355	1.6	0.102323711	1.273148439
Hex2Cer(m38:0+O)	14689933.07	3558330.57	4.13	2.05	0.006927052	2.16	0.057781244	1.455442008
Hex2Cer(m39:0+O)	7616339.58	1863587.46	4.09	2.03	0.017103214	1.77	0.085938371	1.333930435
Hex2Cer(t34:0)	7499785.01	1550854.04	4.84	2.27	0.012080266	1.92	0.072676899	1.3830892
Hex2Cer(t40:0)	7372643.01	1731839.82	4.26	2.09	0.01142532	1.94	0.071774317	1.39210942
MGDG(40:0e)	5927055.77	1464872.1	4.05	2.02	0.045940174	1.34	0.140232382	1.163581977
PE(16:0_12:0)	3513382.96	1711571.74	2.05	1.04	0.048041961	1.32	0.143260719	1.22352587
PE(18:1_12:0)	6154648.61	1683683.23	3.66	1.87	0.019203832	1.72	0.091710427	1.452595078
PE(18:1e_18:2)	60967604.62	22711442.31	2.68	1.42	0.025549406	1.59	0.102617844	1.269633534
TG(15:0_6:0_18:1)	2447478971	832811716.3	2.94	1.56	0.013454471	1.87	0.076408881	1.367958006
TG(16:1_6:0_18:2)	7990858612	3036642532	2.63	1.4	0.022612894	1.65	0.096203287	1.288760429
TG(16:1_6:0_6:0)	191098025	47921245.33	3.99	2	0.002217002	2.65	0.036580538	1.572365781
TG(16:1_8:0_16:1)	1156777033	492236128.8	2.35	1.23	0.003792982	2.42	0.048292441	1.520305979

Accession	BC_Mean	CC_Mean	FC	log2FC	P.value	-log10(P.value)	FDR	VIP
TG(18:0e_6:0_10:1)	233472679.5	59104444.99	3.95	1.98	0.00189697	2.72	0.035323552	1.585361067
TG(18:2e_8:0_10:1)	61478137.6	27727576.88	2.22	1.15	0.030919029	1.51	0.116126091	1.236030389
TG(18:4_6:0_14:1)	1020252095	464629672.4	2.2	1.13	8.01709E-05	4.1	0.01062212	1.783023033
TG(18:4_6:0_9:0)	36643047.62	14605964.61	2.51	1.33	0.022200328	1.65	0.096122696	1.292768903
TG(19:1_18:1_18:1)	908543104.9	383862695.1	2.37	1.24	0.005750497	2.24	0.052792157	1.475866908
TG(20:1e_6:0_6:0)	29654731.35	10465104.04	2.83	1.5	0.020444463	1.69	0.093144361	1.30504056
TG(20:1e_8:0_8:0)	60518336.98	24007585.14	2.52	1.33	0.005063954	2.3	0.051175884	1.490798605
TG(20:2e_6:0_10:0)	10456270.43	1392567.67	7.51	2.91	0.000359289	3.44	0.020442879	1.705847147
TG(22:2_14:1_14:1)	19379562.74	7441952.81	2.6	1.38	0.031143882	1.51	0.116126091	1.235386147
TG(24:0_13:0_13:0)	31656978.81	12353557.32	2.56	1.36	0.000385083	3.41	0.020442879	1.70149039
TG(27:1_6:0_6:0)	292772558.9	125574073.4	2.33	1.22	0.000996412	3	0.028967109	1.63842661
TG(29:2)	1321292.13	150972.82	8.75	3.13	0.002510483	2.6	0.039198354	1.561994846
TG(33:1)	158805424.5	34252161.21	4.64	2.21	0.017892326	1.75	0.087737069	1.326574466
TG(38:1e)	24466188.8	7842387.56	3.12	1.64	0.027735062	1.56	0.109240357	1.254675017
TG(39:6)	2687327.15	1013248.34	2.65	1.41	0.044289593	1.35	0.138443067	1.169600315
TG(40:2e)	12878953.88	2465177.19	5.22	2.39	0.031299395	1.5	0.116126091	1.233772076
TG(41:3)	13824786.17	5030386.11	2.75	1.46	0.038000528	1.42	0.127119575	1.275904445
TG(41:6)	9328042.82	3047398.1	3.06	1.61	0.001136665	2.94	0.029529115	1.626302522
TG(44:6e)	76979772.17	8401907.3	9.16	3.2	0.000410933	3.39	0.020906228	1.801153315
TG(4:0_12:0_17:0)	120537526.9	41287447.62	2.92	1.55	0.029028278	1.54	0.111645144	1.247897215
TG(4:0_12:0_18:2)	43937310.01	3110767.49	14.12	3.82	0.000113094	3.95	0.01062212	1.767005874
TG(4:0_12:0_22:1)	13902079.83	4562897.69	3.05	1.61	0.047084357	1.33	0.141324806	1.155652226
TG(4:0_13:0_18:2)	57763468.09	11278620.91	5.12	2.36	0.032508245	1.49	0.116155927	1.227942263
TG(4:0_13:0_20:5)	21740006.45	8592326.71	2.53	1.34	0.046635754	1.33	0.141324806	1.159882784

Accession	BC_Mean	CC_Mean	FC	log2FC	P.value	-log10(P.value)	FDR	VIP
TG(4:0_14:0_17:0)	468908836	151364982.5	3.1	1.63	0.032060139	1.49	0.116126091	1.230566835
TG(4:0_14:0_18:3)	112154657.8	14977785.37	7.49	2.9	0.002930585	2.53	0.042096991	1.545717877
TG(4:0_14:1_17:0)	544042928	115760425.1	4.7	2.23	0.001389215	2.86	0.032885903	1.611126726
TG(4:0_14:1_18:1)	275172313.5	21068353.53	13.06	3.71	0.002054031	2.69	0.035323552	1.578298392
TG(4:0_14:2_20:4)	104510733.5	14977785.37	6.98	2.8	0.01165258	1.93	0.071774317	1.387141883
TG(4:0_14:4_18:1)	26027701.93	8167110.51	3.19	1.67	0.003923569	2.41	0.048292441	1.515039753
TG(4:0_16:0_17:1)	3242582693	1059593292	3.06	1.61	0.01763071	1.75	0.087154237	1.328762083
TG(4:0_16:0_19:1)	3132021665	865624432.5	3.62	1.86	0.006956464	2.16	0.057781244	1.453940783
TG(4:0_16:1_16:1)	1020252095	464629672.4	2.2	1.13	8.01709E-05	4.1	0.01062212	1.783023033
TG(4:0_16:1_17:1)	646992685.8	131525556.9	4.92	2.3	0.018410102	1.73	0.08991494	1.321667391
TG(4:0_16:1_18:3)	22360122.54	8292489.22	2.7	1.43	0.013233512	1.88	0.075505225	1.370935188
TG(4:0_18:1_18:2)	7990858612	3036642532	2.63	1.4	0.022612894	1.65	0.096203287	1.288760429
TG(4:0_18:1_18:3)	1445892666	394599441.6	3.66	1.87	0.017256136	1.76	0.086351402	1.332191679
TG(4:0_18:2_18:3)	136107412.2	63873373.79	2.13	1.09	0.02036518	1.69	0.093144361	1.307820325
TG(4:0_18:2_19:0)	189860402.8	61106822.88	3.11	1.64	0.012444745	1.91	0.072676899	1.379175683
TG(4:0_18:2_19:1)	25827631.38	7472148.72	3.46	1.79	0.033889739	1.47	0.118906241	1.220493609
TG(4:0_18:3_18:4)	34660812.75	13623096.65	2.54	1.35	0.004237291	2.37	0.048352636	1.628425952
TG(4:0_6:0_14:1)	16373467.43	2800559.62	5.85	2.55	0.025468303	1.59	0.102617844	1.269792313
TG(4:0_6:0_18:2)	28702036.37	6946363.54	4.13	2.05	0.021289469	1.67	0.09502269	1.299580399
TG(4:0_6:0_19:1)	14563874.26	3258493.9	4.47	2.16	0.036663088	1.44	0.125746154	1.205753179
TG(4:0_6:0_20:0)	147127916.6	48406059.54	3.04	1.6	0.002032823	2.69	0.035323552	1.580700588
TG(4:0_6:0_20:5)	11748266.94	3201395.1	3.67	1.88	0.032378634	1.49	0.116155927	1.228951233
TG(4:0_8:0_18:1)	1076542119	259946796.7	4.14	2.05	0.00656479	2.18	0.057781244	1.460550372
TG(4:0_8:0_20:4)	336391759.6	134762937.7	2.5	1.32	0.009043202	2.04	0.066516566	1.421316917

Accession	BC_Mean	CC_Mean	FC	log2FC	P.value	-log10(P.value)	FDR	VIP
TG(6:0_10:0_18:2)	46173889.45	14704740.1	3.14	1.65	0.036635082	1.44	0.125746154	1.206676265
TG(6:0_10:1_10:1)	13126481.78	3290480.61	3.99	2	0.044333529	1.35	0.138443067	1.169382859
TG(6:0_12:0_12:3)	268150661.1	131158261.4	2.04	1.03	0.027903622	1.55	0.109550876	1.256175697
TG(6:0_17:1_17:1)	19150995999	7436820946	2.58	1.36	0.003367787	2.47	0.046226585	1.532738512
TG(6:0_17:1_18:1)	1013984813	316665246.7	3.2	1.68	0.02438735	1.61	0.100597819	1.277478676
TG(6:0_17:1_18:3)	10996585.24	2863895	3.84	1.94	0.04623845	1.33	0.140790891	1.160598516
TG(6:0_6:0_10:0)	9960704.53	1047704.7	9.51	3.25	0.01407997	1.85	0.077728723	1.361362374
TG(6:0_6:0_12:0)	95014861.87	15993197.8	5.94	2.57	0.005239136	2.28	0.051175884	1.583288683
TG(6:0_6:0_14:1)	55047257.05	8901534.08	6.18	2.63	0.003774985	2.42	0.048292441	1.520533649
TG(6:0_6:0_17:1)	1781708.15	326335.56	5.46	2.45	0.011616665	1.93	0.071774317	1.389614119
TG(6:0_6:0_20:5)	44632887.96	14300590.4	3.12	1.64	0.036985588	1.43	0.126496927	1.205005738
TG(6:0_8:0_10:1)	2083802.61	707653.86	2.94	1.56	0.02365545	1.63	0.098511886	1.282194116
TG(6:0_8:0_17:1)	75984000.55	17475717.81	4.35	2.12	0.0174205	1.76	0.086818085	1.330514842
TG(6:0_8:0_18:1)	111962509.8	38113338.46	2.94	1.55	0.000624395	3.2	0.024421337	1.67183768
TG(6:0_8:0_20:5)	1749060.99	544530.39	3.21	1.68	0.037923645	1.42	0.127119575	1.303419364
TG(6:0_8:0_8:0)	1749560.27	412696.85	4.24	2.08	0.023389011	1.63	0.098108184	1.284407864
TG(8:0_8:0_10:0)	274644439.8	60321840.24	4.55	2.19	0.009215804	2.04	0.066979147	1.418897369
TG(8:0_8:0_11:1)	9574469.48	1425736.16	6.72	2.75	0.029077114	1.54	0.111645144	1.247031389
TG(8:0_8:0_14:1)	63268774.41	11196090.54	5.65	2.5	0.003503208	2.46	0.047004575	1.528107435
TG(8:0_8:0_20:3)	151084292.7	23628793.19	6.39	2.68	0.012498152	1.9	0.072676899	1.377453115
TG(8:0_8:0_24:1)	2500571281	1053378287	2.37	1.25	0.002592284	2.59	0.039454059	1.661676626
TG(8:0_8:0_8:0)	64059058.57	13355289.67	4.8	2.26	0.005132189	2.29	0.051175884	1.488502917
ZyE(23:6)	79182784.9	21580158.83	3.67	1.88	0.000656141	3.18	0.024421337	1.668112665
ZyE(24:7)	26966462.99	984676.82	27.39	4.78	0.014259815	1.85	0.077728723	1.359186345

Accession	BC_Mean	CC_Mean	FC	log2FC	P.value	-log10(P.value)	FDR	VIP
CL(80:7)	3366268.49	9131790.77	0.37	-1.44	0.042501781	1.37	0.136564933	1.177470134
CL(83:10)	468575.49	4699128.63	0.1	-3.33	0.000146718	3.83	0.011196382	1.75455838
Cer(m36:0)	1640400.86	6530744.49	0.25	-1.99	0.003907157	2.41	0.048292441	1.516610013
Cer(m40:0)	1472086.84	4076836.66	0.36	-1.47	0.01641316	1.78	0.084558936	1.339309168
Cer(m41:0)	30632032.18	68695334.14	0.45	-1.17	0.048610898	1.31	0.144413397	1.151070732
Hex1Cer(d16:0_22:4)	2364578.03	17074105.51	0.14	-2.85	0.002032143	2.69	0.035323552	1.579212232
Hex1Cer(d36:3)	7983893.76	52156615.38	0.15	-2.71	0.001577078	2.8	0.034569916	1.601663727
Hex1Cer(m20:1_18:2)	3703964.55	14202580.53	0.26	-1.94	0.009477447	2.02	0.06807037	1.414967696
Hex1Cer(t18:0_20:4)	87527.1	5388953.26	0.02	-5.94	3.8895E-06	5.41	0.000949816	1.87916772
Hex1Cer(t20:0_20:4)	155556.13	1501419.39	0.1	-3.27	0.023379278	1.63	0.098108184	1.363111676
Hex1Cer(t36:1)	333778.61	1531636.38	0.22	-2.2	0.012668314	1.9	0.073308112	1.37683929
LPC(16:0e)	6936685.88	24448650.32	0.28	-1.82	0.033543394	1.47	0.118371342	1.222299404
LPC(16:1e)	2770043.74	15233031.07	0.18	-2.46	0.011697276	1.93	0.071774317	1.3875179
LPC(18:0)	79863574.89	256865465.1	0.31	-1.69	0.046963465	1.33	0.141324806	1.15863057
LPC(18:0e)	4344548.17	32304068.16	0.13	-2.89	0.02108405	1.68	0.09502269	1.300647191
LPC(18:1)	3331823.95	12211813.73	0.27	-1.87	0.016816067	1.77	0.085551743	1.337458782
LPC(18:1e)	4945831.66	28192639.74	0.18	-2.51	0.044333137	1.35	0.138443067	1.170502143
LPC(18:2)	7596568.7	299705736.9	0.03	-5.3	5.96869E-05	4.22	0.010411103	1.795886029
LPC(18:3)	774932.36	9891454.99	0.08	-3.67	0.001243764	2.91	0.030992561	1.62222719
LPC(20:1)	891302.32	5097160.59	0.17	-2.52	0.024262024	1.62	0.100420105	1.279580692
LPC(20:3)	186654.56	45964058.79	0	-7.94	3.63381E-06	5.44	0.000949816	1.880589113
LPC(20:4)	3331823.95	13357309.31	0.25	-2	0.014635887	1.83	0.078379029	1.357880182
LPC(22:5)	753405.36	12373511.1	0.06	-4.04	0.034635353	1.46	0.120827901	1.215544376
LPC(30:0)	6967994.69	25624912.79	0.27	-1.88	0.015864814	1.8	0.083106074	1.34479803

Accession	BC_Mean	CC_Mean	FC	log2FC	P.value	-log10(P.value)	FDR	VIP
LPC(32:1)	10063160.56	44364935.48	0.23	-2.14	0.012487904	1.9	0.072676899	1.378845239
LPE(18:0)	146078.21	8976335.76	0.02	-5.94	8.28726E-07	6.08	0.000337292	1.908605064
LPE(20:2)	239221.59	4848814.46	0.05	-4.34	0.000177828	3.75	0.012772215	1.746038441
LPE(20:3)	84805.16	3538444.73	0.02	-5.38	5.71012E-06	5.24	0.001162009	1.869974323
LPE(20:4)	609843.76	13273660.72	0.05	-4.44	0.000103417	3.99	0.01062212	1.772257575
LdMePE(18:0)	357343.92	1869187.52	0.19	-2.39	0.009203039	2.04	0.066979147	1.419489738
LdMePE(18:1)	180920.63	1637859.05	0.11	-3.18	0.005183963	2.29	0.051175884	1.488732079
MGDG(16:0_16:0)	264285.74	2649151.72	0.1	-3.33	0.004821212	2.32	0.051175884	1.494675604
MGDG(29:0e)	4409099.24	13865276.19	0.32	-1.65	0.032146289	1.49	0.116126091	1.230756082
MGDG(30:0e)	469808596.6	1383331311	0.34	-1.56	0.031606627	1.5	0.116126091	1.233590004
MGDG(35:1e)	1767799.6	4667919.78	0.38	-1.4	0.042873401	1.37	0.137037755	1.176290307
MGDG(36:2e)	2103750.08	9090588.59	0.23	-2.11	0.019901557	1.7	0.09230868	1.310650626
MGDG(37:1e)	10703285.77	45121779.43	0.24	-2.08	0.007947733	2.1	0.062206292	1.437509376
MGDG(38:2e)	11553371.59	39145979	0.3	-1.76	0.032737675	1.48	0.116199714	1.226691198
MGDG(38:5)	56066330.15	150000454.3	0.37	-1.42	0.037895641	1.42	0.127119575	1.199838615
PC(12:1e_20:3)	4033182.06	18219971.94	0.22	-2.18	0.011682115	1.93	0.071774317	1.38838272
PC(14:1e_20:4)	12163809.42	61076634.83	0.2	-2.33	0.019198057	1.72	0.091710427	1.315258723
PC(15:0_20:4)	8058263.86	21670989.02	0.37	-1.43	0.044659036	1.35	0.138749829	1.168237618
PC(16:0_18:3)	9865700.39	25551574.24	0.39	-1.37	0.045661536	1.34	0.140082249	1.164025454
PC(16:0_20:4)	7964510.56	53935466.91	0.15	-2.76	0.005080201	2.29	0.051175884	1.48978863
PC(16:0_22:4)	949418.64	18145908.59	0.05	-4.26	0.001122676	2.95	0.029529115	1.629291472
PC(16:0e_16:0)	5881857.08	17434239.03	0.34	-1.57	0.033037923	1.48	0.116925519	1.225420543
PC(16:1e_18:1)	1881551.33	8030639.83	0.23	-2.09	0.013132729	1.88	0.075281984	1.371749167
PC(17:0_18:3)	2757896.69	8711014.22	0.32	-1.66	0.008759177	2.06	0.065914876	1.424467683

Accession	BC_Mean	CC_Mean	FC	log2FC	P.value	-log10(P.value)	FDR	VIP
PC(17:0_20:4)	341574.03	1318977.1	0.26	-1.95	0.02063643	1.69	0.093669447	1.303897405
PC(17:1_18:1)	2494153.27	11995045.69	0.21	-2.27	0.004421664	2.35	0.049902405	1.504064812
PC(17:1_20:5)	2846042.63	15901814.94	0.18	-2.48	0.002175959	2.66	0.036395148	1.573711861
PC(18:0_18:3)	66377334.45	222017304.3	0.3	-1.74	0.021134448	1.68	0.09502269	1.300964266
PC(18:0_20:3)	20583107.06	163116292.4	0.13	-2.99	0.002034086	2.69	0.035323552	1.579811982
PC(18:0_20:4)	12809545.51	54827405.12	0.23	-2.1	0.009785249	2.01	0.069087127	1.410895053
PC(18:0_22:4)	1243547.64	7406596.11	0.17	-2.57	0.004862175	2.31	0.051175884	1.493861704
PC(18:0_22:5)	2615342.11	7845564.24	0.33	-1.58	0.03877508	1.41	0.128803063	1.193985717
PC(18:0_22:6)	129538.48	598908.95	0.22	-2.21	0.032088378	1.49	0.116126091	1.229214406
PC(18:0e_18:2)	6486078.19	18942378.06	0.34	-1.55	0.036585927	1.44	0.125746154	1.206595092
PC(18:0e_20:4)	1855742.62	7348213.8	0.25	-1.99	0.031675705	1.5	0.116126091	1.23264641
PC(18:1_20:3)	8438143.16	57590983.81	0.15	-2.77	0.00194497	2.71	0.035323552	1.584014603
PC(18:1_20:4)	19072402.47	66847774.93	0.29	-1.81	0.03579442	1.45	0.124161894	1.210803619
PC(18:2_18:2)	14348031.55	41331209.73	0.35	-1.53	0.012200043	1.91	0.072676899	1.381935715
PC(18:2e_15:0)	1881551.33	8030639.83	0.23	-2.09	0.013132729	1.88	0.075281984	1.371749167
PC(20:0_18:2)	11364839.72	58745438.15	0.19	-2.37	0.005506347	2.26	0.05132252	1.48096097
PC(20:0_18:3)	101061.65	382892.22	0.26	-1.92	0.017730788	1.75	0.087295532	1.430251797
PC(20:0_20:4)	313745.06	911972.27	0.34	-1.54	0.039294915	1.41	0.129323697	1.294556056
PC(20:3_18:2)	320033.63	3656186.81	0.09	-3.51	0.000132293	3.88	0.010768666	1.759458105
PC(20:4e_18:0)	3478317.26	15058422.78	0.23	-2.11	0.01874666	1.73	0.090473013	1.319139902
PC(30:0e)	6953833.67	25842693.06	0.27	-1.89	0.014561682	1.84	0.078325169	1.357247271
PC(32:0e)	9685071.05	33157350.41	0.29	-1.78	0.023462399	1.63	0.098108184	1.284128465
PC(32:2e)	1103516.03	6114793.73	0.18	-2.47	0.00532641	2.27	0.051209025	1.484557419
PC(32:3e)	80061.26	7020000.93	0.01	-6.45	7.0966E-07	6.15	0.000337292	1.91075197

Accession	BC_Mean	CC_Mean	FC	log2FC	P.value	-log10(P.value)	FDR	VIP
PC(32:5e)	2638816.8	62884176.55	0.04	-4.57	0.000257511	3.59	0.017467804	1.726358408
PC(32:6e)	22970997.29	57814568.08	0.4	-1.33	0.015169752	1.82	0.080531597	1.349958837
PC(33:1e)	4677809.43	29467060.18	0.16	-2.66	0.001585516	2.8	0.034569916	1.601265305
PC(34:0e)	9618376.72	37715129.33	0.26	-1.97	0.022048065	1.66	0.095803157	1.294181212
PC(34:3e)	36829414.52	231611868.5	0.16	-2.65	0.001645987	2.78	0.034650854	1.59877118
PC(34:4e)	10063160.56	44364935.48	0.23	-2.14	0.012487904	1.9	0.072676899	1.378845239
PC(35:2e)	7594254.17	42096283.75	0.18	-2.47	0.001763661	2.75	0.035323552	1.592129876
PC(35:3)	31897930.33	178032449.5	0.18	-2.48	0.002000258	2.7	0.035323552	1.581047943
PC(35:4)	7260829.64	45773165.67	0.16	-2.66	0.004540154	2.34	0.050395708	1.501815766
PC(35:4e)	1357005.73	9381321.73	0.14	-2.79	0.005289718	2.28	0.051209025	1.485066504
PC(36:2e)	12852337.85	32942413.87	0.39	-1.36	0.045577563	1.34	0.140082249	1.164027069
PC(36:4e)	27104042.88	164685952.1	0.16	-2.6	0.004946274	2.31	0.051175884	1.492438502
PC(36:5e)	15285037.68	177946803.2	0.09	-3.54	0.001723526	2.76	0.035323552	1.594701971
PC(37:3)	14135874.23	44006768.11	0.32	-1.64	0.028716743	1.54	0.111645144	1.249411198
PC(38:2)	34972617.87	123350730.5	0.28	-1.82	0.022390706	1.65	0.096203287	1.291257168
PC(38:4e)	41825142.11	183605085.6	0.23	-2.13	0.023056853	1.64	0.097564377	1.286558606
PC(38:5e)	32535690.65	92012358.75	0.35	-1.5	0.041939564	1.38	0.135114004	1.180699019
PC(38:6e)	9589056.94	67202510.67	0.14	-2.81	0.004132185	2.38	0.048352636	1.511723281
PC(8:0e_24:1)	10063893.99	44429930.23	0.23	-2.14	0.012440227	1.91	0.072676899	1.379377192
PE(16:0_20:4)	15448274.41	61532136.89	0.25	-1.99	0.031109081	1.51	0.116126091	1.23661789
PE(16:0_22:6)	156221.65	989877.84	0.16	-2.66	0.044261523	1.35	0.138443067	1.17086899
PE(16:1e_20:4)	19592370.68	132020036.2	0.15	-2.75	0.003838537	2.42	0.048292441	1.519892501
PE(16:1e_22:5)	14914018.2	43765166.1	0.34	-1.55	0.047108269	1.33	0.141324806	1.158490099
PE(18:0_20:3)	8100861.25	53720798.03	0.15	-2.73	0.001419083	2.85	0.032885903	1.610453608

Accession	BC_Mean	CC_Mean	FC	log2FC	P.value	-log10(P.value)	FDR	VIP
PE(18:0_22:4)	2327144	14931906.37	0.16	-2.68	0.002617345	2.58	0.039454059	1.556261286
PE(18:0e_18:2)	2703364.13	34798765.58	0.08	-3.69	0.009009381	2.05	0.066516566	1.421581417
PE(18:0e_20:3)	17127.49	1151949.75	0.01	-6.07	0.00010642	3.97	0.01062212	1.770605497
PE(18:1_20:3)	3491231.25	24090469.02	0.14	-2.79	0.001435953	2.84	0.032885903	1.609744323
PE(18:1e_20:3)	4386275.7	17320049.07	0.25	-1.98	0.022454769	1.65	0.096203287	1.290798834
PE(18:1e_22:6)	90782.55	761101.66	0.12	-3.07	0.004068009	2.39	0.048352636	1.514000663
PE(19:1_15:0)	50951571.4	211487573.5	0.24	-2.05	0.032119542	1.49	0.116126091	1.230905583
PE(20:2_18:2)	8058263.86	21670989.02	0.37	-1.43	0.044659036	1.35	0.138749829	1.168237618
PE(20:3_18:2)	1856389.22	5789979.48	0.32	-1.64	0.025170591	1.6	0.102617844	1.272634079
PE(20:3e_18:2)	2952996.02	8391781.47	0.35	-1.51	0.038237399	1.42	0.127562472	1.198867661
PE(22:5_18:2)	7994392.41	21809271.62	0.37	-1.45	0.043475137	1.36	0.138328102	1.17351465
PE(31:0e)	9871284.22	37382137.35	0.26	-1.92	0.010231528	1.99	0.069455654	1.405488438
PE(33:2e)	10557035.18	39089619.32	0.27	-1.89	0.014336836	1.84	0.077801229	1.35940119
PE(34:3p)	8361838.33	24130839.26	0.35	-1.53	0.045314908	1.34	0.139720967	1.165728661
PE(36:0)	32980159.88	177667600.4	0.19	-2.43	0.00216124	2.67	0.036395148	1.574172501
PE(36:4)	50951571.4	211487573.5	0.24	-2.05	0.032119542	1.49	0.116126091	1.230905583
PE(36:5e)	7232502.96	41341440.44	0.17	-2.52	0.007429556	2.13	0.060076075	1.446120134
PE(37:2e)	16404797.13	172597646.1	0.1	-3.4	0.000950525	3.02	0.028967109	1.641871817
PE(40:6)	2696767.79	15718142.13	0.17	-2.54	0.001783991	2.75	0.035323552	1.59117829
PI(16:0_18:2)	1539302.86	6077230.15	0.25	-1.98	0.021645266	1.66	0.09502269	1.296734212
PI(18:0_22:4)	861270.26	2286540.52	0.38	-1.41	0.021691466	1.66	0.09502269	1.296051019
PI(18:0_22:5)	710916.71	1707980.95	0.42	-1.26	0.033670867	1.47	0.118478759	1.221271649
PI(18:1_20:4)	1871443.7	4881540.4	0.38	-1.38	0.029242275	1.53	0.111927328	1.246771577
PI(38:5)	3429878.65	9091067.32	0.38	-1.41	0.021664366	1.66	0.09502269	1.296643664

Accession	BC_Mean	CC_Mean	FC	log2FC	P.value	-log10(P.value)	FDR	VIP
PS(18:0_18:0)	20762702.49	155326993.8	0.13	-2.9	0.001195713	2.92	0.030415955	1.623886603
PS(18:0_18:1)	38443689.72	103633901.4	0.37	-1.43	0.025343452	1.6	0.102617844	1.27015352
PS(18:0_20:2)	37054230.25	97078039.04	0.38	-1.39	0.037935341	1.42	0.127119575	1.199176541
PS(18:0_20:3)	8012463.72	58092041.32	0.14	-2.86	0.001454413	2.84	0.032885903	1.607973196
PS(18:0_22:4)	21972686.67	112863845.9	0.19	-2.36	0.006648119	2.18	0.057781244	1.458271071
PS(18:0_22:5)	1715643.56	6829944.93	0.25	-1.99	0.010333005	1.99	0.069455654	1.403070303
PS(18:0_22:6)	1124158.27	8169276.47	0.14	-2.86	0.0104667	1.98	0.069455654	1.403253867
PS(18:1_22:0)	519782.89	3437290.27	0.15	-2.73	0.00085362	3.07	0.028224422	1.648810988
PS(18:2_20:4)	1637740.73	6579681.05	0.25	-2.01	0.029512562	1.53	0.11212534	1.245824896
PS(19:0_18:1)	306751.59	1011684.11	0.3	-1.72	0.04481327	1.35	0.138875641	1.166872735
PS(20:0_18:1)	2472502.76	11387955.57	0.22	-2.2	0.004207853	2.38	0.048352636	1.509259208
PS(20:3_20:3)	1927575.38	12193759.52	0.16	-2.66	0.002536175	2.6	0.039198354	1.559781702
PS(22:0_18:2)	2654220.6	17948189.24	0.15	-2.76	0.002674968	2.57	0.03983093	1.554778209
PS(22:4_18:2)	5078953.56	13890839.96	0.37	-1.45	0.037571949	1.43	0.127119575	1.201672098
PS(36:2e)	76673.54	7519262.52	0.01	-6.62	3.83922E-07	6.42	0.000337292	1.919974603
PS(38:3)	19079458.68	49688368.43	0.38	-1.38	0.038880352	1.41	0.128803063	1.194817659
PS(40:5)	37054230.25	97078039.04	0.38	-1.39	0.037935341	1.42	0.127119575	1.199176541
PS(40:6)	19065048.5	49390427.21	0.39	-1.37	0.039551169	1.4	0.129817145	1.191518432
SM(d19:0_19:1)	368673372.2	1131168808	0.33	-1.62	0.049417367	1.31	0.146098319	1.148647949
SM(d28:1)	279148.88	1776068.98	0.16	-2.67	0.016912111	1.77	0.085683353	1.33768742
SM(d30:0)	325861.82	1448286.72	0.22	-2.15	0.003369505	2.47	0.046226585	1.533981979
SM(d32:0)	5883443.64	19166853.5	0.31	-1.7	0.016238422	1.79	0.084013194	1.341979798
SM(d32:1)	55107582.49	260433810.8	0.21	-2.24	0.004655107	2.33	0.051175884	1.499645292
SM(d34:2)	2704327.45	28155284.09	0.1	-3.38	0.000745434	3.13	0.026769838	1.660098423

Accession	BC_Mean	CC_Mean	FC	log2FC	P.value	-log10(P.value)	FDR	VIP
SM(d34:4)	64827864.27	504277593.1	0.13	-2.96	0.001380732	2.86	0.032885903	1.613758741
SM(d35:4)	3329578.88	9625298.68	0.35	-1.53	0.034923755	1.46	0.121486907	1.215232669
SM(d42:3)	10817710.14	40230105.5	0.27	-1.89	0.024640053	1.61	0.101297996	1.275887288
SQDG(31:1)	632188.92	2772914.47	0.23	-2.13	0.023720307	1.62	0.098511886	1.282836158
TG(10:0_18:3_20:4)	18019858.84	63117974.09	0.29	-1.81	0.001613987	2.79	0.034573295	1.601093583
TG(10:0_20:5_23:1)	20061985.96	43387636.26	0.46	-1.11	0.005232739	2.28	0.051175884	1.486574071
TG(14:0_14:0_20:4)	527350216.8	1082931350	0.49	-1.04	0.0295695	1.53	0.11212534	1.244546287
TG(14:0_14:0_20:5)	18284260.9	40906388.07	0.45	-1.16	0.02538595	1.6	0.102617844	1.271156062
TG(14:0e_18:0_18:0)	5638845.51	17475031.11	0.32	-1.63	0.011816635	1.93	0.07214056	1.387540585
TG(15:0_14:0_18:3)	129633091.7	423220893.1	0.31	-1.71	0.009788758	2.01	0.069087127	1.504492887
TG(15:0_17:0_18:3)	159232352.7	774136988.2	0.21	-2.28	0.011432659	1.94	0.071774317	1.390665091
TG(16:0_10:0_17:0)	94495426.94	292986406.6	0.32	-1.63	0.007169978	2.14	0.058363617	1.569270923
TG(16:0_10:0_22:5)	12777588.96	28552306.72	0.45	-1.16	0.02149776	1.67	0.09502269	1.297564582
TG(16:0_12:3_14:0)	569003146.1	1848519789	0.31	-1.7	0.006931841	2.16	0.057781244	1.553928151
TG(16:0_12:3_16:0)	417205986.2	1286119340	0.32	-1.62	0.003260328	2.49	0.045757014	1.53537457
TG(16:0_14:0_14:0)	4711679061	14949560746	0.32	-1.67	0.000125777	3.9	0.010768666	1.762555411
TG(16:0_14:0_17:0)	1794519046	4636585312	0.39	-1.37	0.004171658	2.38	0.048352636	1.616267018
TG(16:0_14:0_17:1)	54987596.7	158751700.8	0.35	-1.53	0.000275557	3.56	0.017708192	1.722560383
TG(16:0_14:0_20:4)	559176836.8	1224693701	0.46	-1.13	0.027428434	1.56	0.10873415	1.257468276
TG(16:0_16:0_20:3)	321953005.4	891082101	0.36	-1.47	0.021317681	1.67	0.09502269	1.299089963
TG(16:0_16:0_24:0)	238592239.9	624306926.9	0.38	-1.39	0.010888955	1.96	0.07148072	1.396567651
TG(16:0_17:0_18:3)	140553895.5	419614480.5	0.33	-1.58	0.00829793	2.08	0.063323581	1.527760022
TG(16:0_19:0_20:3)	47434440.92	97334784.32	0.49	-1.04	0.019677648	1.71	0.092266387	1.310574233
TG(16:0_20:4_20:4)	104047607.3	283268115.6	0.37	-1.44	0.000595984	3.22	0.024421337	1.675438624

Accession	BC_Mean	CC_Mean	FC	log2FC	P.value	-log10(P.value)	FDR	VIP
TG(16:0_20:4_22:5)	11235084.04	50591109.8	0.22	-2.17	0.040046099	1.4	0.130727352	1.190919108
TG(16:0_22:5_24:0)	2816499.85	16262316.35	0.17	-2.53	0.007158422	2.15	0.058363617	1.45033864
TG(16:0_6:0_22:6)	3925374.53	28059606.19	0.14	-2.84	0.007575182	2.12	0.060850637	1.443589046
TG(16:1e_16:0_16:0)	29104085.58	96967408.34	0.3	-1.74	0.00357943	2.45	0.047505265	1.52798919
TG(17:0_12:0_14:3)	118347431.5	294746316.7	0.4	-1.32	0.000113014	3.95	0.01062212	1.767101751
TG(17:0_14:0_18:3)	137588595	532611177.4	0.26	-1.95	0.009969262	2	0.069455654	1.522169058
TG(17:0_6:0_14:3)	5937836.52	17331514.17	0.34	-1.55	0.005501587	2.26	0.05132252	1.479083662
TG(17:0_6:0_17:0)	3532838.15	14845269.36	0.24	-2.07	0.011697862	1.93	0.071774317	1.387131261
TG(18:0_10:0_18:0)	78023227.11	167317130.6	0.47	-1.1	0.000369463	3.43	0.020442879	1.704712068
TG(18:0_14:3_16:0)	5861609.65	14972116.98	0.39	-1.35	0.004881639	2.31	0.051175884	1.492247443
TG(18:0_16:0_17:0)	112567508.3	269017692.7	0.42	-1.26	0.002856241	2.54	0.041774307	1.548311192
TG(18:0_16:0_23:0)	78172052.53	174302851.9	0.45	-1.16	0.014229095	1.85	0.077728723	1.359508884
TG(18:0_16:0_24:0)	85205082.59	259210844.9	0.33	-1.61	0.008435401	2.07	0.063972822	1.429531387
TG(18:0_18:0_22:4)	10696227.1	35106170.38	0.3	-1.71	0.012382169	1.91	0.072676899	1.379583108
TG(18:0_18:0_22:6)	125846638.9	316607022	0.4	-1.33	0.01930334	1.71	0.091710427	1.314541604
TG(18:0_18:0_24:0)	25054530.3	104126891.7	0.24	-2.06	0.004996741	2.3	0.051175884	1.491071168
TG(18:0_18:1_20:3)	210784952.2	726598790.6	0.29	-1.79	0.000855286	3.07	0.028224422	1.649030285
TG(18:0_18:1_22:4)	89497923.62	317324437.9	0.28	-1.83	0.004131101	2.38	0.048352636	1.511689774
TG(18:0_18:3_23:0)	5183506.43	12038426.22	0.43	-1.22	0.031002692	1.51	0.116126091	1.235790034
TG(18:0_18:3_24:0)	4709218.95	15247923.73	0.31	-1.7	0.011690806	1.93	0.071774317	1.387147478
TG(18:0_20:0_22:5)	7490018.52	34009229.76	0.22	-2.18	0.002368732	2.63	0.037561324	1.566488988
TG(18:0_20:1_22:5)	6143507.5	21623241.17	0.28	-1.82	0.028292823	1.55	0.110369128	1.252195822
TG(18:0_20:3_20:5)	39795391.82	119568838.3	0.33	-1.59	0.002873908	2.54	0.041774307	1.547730688
TG(18:0_20:3_23:0)	1375636.11	5310374.21	0.26	-1.95	0.032079463	1.49	0.116126091	1.230208197

Accession	BC_Mean	CC_Mean	FC	log2FC	P.value	-log10(P.value)	FDR	VIP
TG(18:0_23:1_24:0)	1808267.95	5507755.46	0.33	-1.61	0.021712801	1.66	0.09502269	1.297577435
TG(18:0_6:0_22:5)	14034240.77	45125118.89	0.31	-1.68	0.00054265	3.27	0.024421337	1.681185453
TG(18:1_10:4_20:4)	6766891.09	14224630.63	0.48	-1.07	0.015843329	1.8	0.083106074	1.344463295
TG(18:1_12:3_20:4)	14472406.24	45294566.47	0.32	-1.65	0.009909908	2	0.069455654	1.410125817
TG(18:1_17:1_18:2)	160368775.8	353331277.3	0.45	-1.14	0.004094363	2.39	0.048352636	1.512538277
TG(18:1_18:1_22:6)	33403284.99	110136938.7	0.3	-1.72	0.011685834	1.93	0.071774317	1.3880864
TG(18:1e_14:0_14:0)	9863810.3	34712265.89	0.28	-1.82	0.022498254	1.65	0.096203287	1.290570546
TG(18:4_8:0_19:0)	2549227.98	11052022.95	0.23	-2.12	0.005128771	2.29	0.051175884	1.591195028
TG(19:0_9:0_21:0)	13159054.21	31636226.37	0.42	-1.27	0.000515542	3.29	0.024210622	1.683247979
TG(19:1_18:1_20:3)	12662103.03	52233417.38	0.24	-2.04	0.000335441	3.47	0.020442879	1.709988124
TG(20:0_20:4_20:4)	2609718.01	18901371.3	0.14	-2.86	0.000995651	3	0.028967109	1.638043238
TG(20:3_20:3_20:3)	6145650.71	31130833.07	0.2	-2.34	0.011536741	1.94	0.071774317	1.389062817
TG(20:4_14:1_20:4)	9215565.21	23403283.99	0.39	-1.34	0.039694808	1.4	0.129939306	1.192766499
TG(25:0_16:0_18:0)	23994011.23	66343075.4	0.36	-1.47	0.019798357	1.7	0.092266387	1.310567965
TG(25:0_18:0_18:0)	5392491.44	20732733.47	0.26	-1.94	0.0010923	2.96	0.029529115	1.631406784
TG(25:0_18:1_24:0)	680412.42	5579549.86	0.12	-3.04	0.00634652	2.2	0.057400744	1.464325899
TG(25:0_6:0_18:4)	4559816.09	17802513.2	0.26	-1.97	0.000614387	3.21	0.024421337	1.673021003
TG(26:0_16:0_23:0)	1295597.15	4442069.91	0.29	-1.78	0.019762428	1.7	0.092266387	1.310574264
TG(26:0_18:0_18:0)	8003690.29	36691840.08	0.22	-2.2	0.002256116	2.65	0.036729569	1.570939682
TG(26:1_18:1_18:2)	12120185.69	29046667.55	0.42	-1.26	0.045882782	1.34	0.140232382	1.16338457
TG(27:0_18:0_18:1)	3860675.07	11099759.59	0.35	-1.52	0.008799447	2.06	0.065914876	1.425688248
TG(30:0_16:0_18:0)	3324898.39	13088342.58	0.25	-1.98	0.001906574	2.72	0.035323552	1.587178643
TG(30:0_18:0_18:0)	1066370.14	5123114.7	0.21	-2.26	0.037091371	1.43	0.126504368	1.205158546
TG(44:1)	118708695.8	289533228.6	0.41	-1.29	0.046803273	1.33	0.141324806	1.159173649

Accession	BC_Mean	CC_Mean	FC	log2FC	P.value	-log10(P.value)	FDR	VIP
TG(44:6)	9119777.08	18669591.95	0.49	-1.03	0.036492736	1.44	0.125746154	1.206991825
TG(46:4)	118708695.8	289533228.6	0.41	-1.29	0.046803273	1.33	0.141324806	1.159173649
TG(48:0e)	18783404.5	107639721.4	0.17	-2.52	0.000970645	3.01	0.028967109	1.64149177
TG(4:0_14:0_20:3)	762133875.9	1774697430	0.43	-1.22	0.019740351	1.7	0.092266387	1.310865294
TG(4:0_15:0_20:4)	1961028.4	5705904.31	0.34	-1.54	0.019303505	1.71	0.091710427	1.315852348
TG(4:0_18:0_20:4)	56929791.62	139419140.4	0.41	-1.29	0.000660036	3.18	0.024421337	1.6682615
TG(4:0_18:3_20:4)	81278378.77	175964471.1	0.46	-1.11	0.013650033	1.86	0.076805024	1.367951144
TG(4:0_20:3_20:3)	34006376.49	96722333.79	0.35	-1.51	0.006866738	2.16	0.057781244	1.57373683
TG(4:0_20:3_20:5)	4204736.1	14234671.72	0.3	-1.76	0.007722817	2.11	0.061602746	1.442655552
TG(52:0)	48712893.04	177827803.2	0.27	-1.87	0.013727794	1.86	0.076888242	1.453013744
TG(52:7e)	17359154.46	41038273.66	0.42	-1.24	0.016583794	1.78	0.085079043	1.33789375
TG(56:6e)	3034080	10541891.11	0.29	-1.8	0.00894712	2.05	0.066516566	1.421686713
TG(6:0_14:0_22:5)	10405910.77	48010915.45	0.22	-2.21	0.020110683	1.7	0.092660921	1.307756556
TG(6:0_18:3_20:4)	88470363.68	211200851.8	0.42	-1.26	0.010766043	1.97	0.071055887	1.495199217
TG(8:0_18:2_18:3)	9480597.14	29623740.12	0.32	-1.64	0.019958633	1.7	0.09230868	1.308683127
TG(8:0_18:3_22:5)	14394445.43	43041655.14	0.33	-1.58	0.013634202	1.87	0.076805024	1.366083958
TG(8:0_8:0_14:0)	3577992.04	7287626.76	0.49	-1.03	0.014043974	1.85	0.077728723	1.362869039
TG(9:0_18:2_18:2)	5719151.28	14371723.66	0.4	-1.33	0.002303223	2.64	0.0370031	1.567927764
ZyE(18:1)	215067635.3	469640028.6	0.46	-1.13	0.045202842	1.34	0.139720967	1.2346304
ZyE(23:1)	18005001.9	44349285.56	0.41	-1.3	0.043962455	1.36	0.138443067	1.170682905
dMePE(16:0_18:2)	2720688.2	8021255.21	0.34	-1.56	0.031390654	1.5	0.116126091	1.234216109

Table S4. The differential lipids identified between yak colostrum (YC) and buffalo colostrum (BC) (VIP > 1， p value < 0.05, FC > 2 or FC < 0.5).

Accession	YC_Mean	BC_Mean	FC	log2FC	P.value	-log10(P.value)	FDR	VIP
BisMePA(4:0_22:0)	4507504.55	1131294.77	3.98	1.99	0.004286958	2.37	0.020285441	1.396456595
BisMePA(4:0_24:0)	10607754.86	1592451.83	6.66	2.74	0.000133491	3.87	0.001752604	1.497689163
Cer(d16:0_23:0)	424884.33	87025.87	4.88	2.29	0.003524687	2.45	0.01756589	1.420406247
Cer(d39:0)	8442504.3	3313575.85	2.55	1.35	0.00560828	2.25	0.023694496	1.342523916
Cer(d39:1+O)	827973.05	27883.29	29.69	4.89	8.81299E-07	6.05	0.000153724	1.623807997
Cer(t18:1_23:0)	1304298.16	251415.26	5.19	2.38	0.000692986	3.16	0.005958699	1.417073099
Cer(t39:1)	811763.38	36442.8	22.27	4.48	1.21894E-05	4.91	0.000549147	1.574261172
Cer(t41:1)	558732.26	44569.02	12.54	3.65	0.000166119	3.78	0.002112823	1.488505054
DG(14:0e)	2753961.32	1241907.46	2.22	1.15	0.006167025	2.21	0.025353325	1.249361032
DG(22:1e)	22105094.39	10554255.31	2.09	1.07	0.040001707	1.4	0.097372248	1.086646294
DG(22:2e)	1602689902	682556634.3	2.35	1.23	0.006296567	2.2	0.025559072	1.37040182
DG(24:0)	15168899.84	4949200.89	3.06	1.62	0.005417973	2.27	0.023375776	1.3737934
DG(25:2e)	25997586.69	4729055.67	5.5	2.46	0.002389435	2.62	0.013761791	1.442306181
DG(26:3)	15545621.16	5073255.92	3.06	1.62	0.004741659	2.32	0.021442836	1.387595205
DG(27:2e)	23990483.58	9305736.02	2.58	1.37	0.010301406	1.99	0.036040161	1.195647868
DG(38:2e)	12643539.57	3090723.2	4.09	2.03	1.36448E-05	4.87	0.000573889	1.571415545
Hex2Cer(d39:0)	19198745.38	7616339.58	2.52	1.33	0.013716434	1.86	0.043227821	1.250657616
Hex2Cer(m39:0+O)	19198745.38	7616339.58	2.52	1.33	0.013716434	1.86	0.043227821	1.250657616
LPC(12:0)	6700608.07	2282085.07	2.94	1.55	0.002738795	2.56	0.014862528	1.321766488
LPC(18:3)	7755821.7	774932.36	10.01	3.32	0.00138551	2.86	0.009557669	1.501564387

Accession	YC_Mean	BC_Mean	FC	log2FC	P.value	-log10(P.value)	FDR	VIP
LPC(20:0)	1486519.22	265232.44	5.6	2.49	0.003301407	2.48	0.016724454	1.41211762
LPE(20:2)	1153530.83	239221.59	4.82	2.27	0.004332621	2.36	0.020346537	1.379945333
PC(16:0_18:3)	28531068.26	9865700.39	2.89	1.53	0.012933218	1.89	0.042547098	1.28163213
PC(18:0_18:1)	3059681.44	1002907.79	3.05	1.61	0.016928346	1.77	0.050910126	1.24327641
PC(18:0_22:6)	993222.51	129538.48	7.67	2.94	0.004355702	2.36	0.020346537	1.419070036
PC(19:0_18:1)	3182784.19	1382840.81	2.3	1.2	0.031206969	1.51	0.081592526	1.148255913
PC(20:0_18:3)	926132.78	101061.65	9.16	3.2	0.006311914	2.2	0.025559072	1.382987457
PC(20:0_20:4)	873806.65	313745.06	2.79	1.48	0.039255309	1.41	0.096440105	1.112725808
PC(29:0)	2170984.56	852762.87	2.55	1.35	0.013736605	1.86	0.043227821	1.253020869
PC(31:1)	80180171.08	25224285.21	3.18	1.67	0.002665953	2.57	0.014531823	1.43118389
PC(33:4)	80180171.08	25224285.21	3.18	1.67	0.002665953	2.57	0.014531823	1.43118389
PC(36:5)	763556.55	270441.63	2.82	1.5	0.009769237	2.01	0.034877891	1.201493241
PC(38:2)	79593753.6	34972617.87	2.28	1.19	0.033207595	1.48	0.085181668	1.121440726
PE(15:0_16:0)	978398.53	316223.98	3.09	1.63	0.000591904	3.23	0.005237068	1.426040762
PE(16:0_12:0)	7859105.51	3513382.96	2.24	1.16	0.001178939	2.93	0.008629407	1.383568807
PE(16:0_18:3)	7536840.22	1362804.84	5.53	2.47	0.002008453	2.7	0.012455602	1.472135297
PE(16:0_22:6)	2824586.59	156221.65	18.08	4.18	9.96215E-05	4	0.001465516	1.610990798
PE(18:0_22:6)	3096017.11	376311.2	8.23	3.04	0.000183479	3.74	0.002262904	1.588256437
PE(18:0e_20:3)	170870.82	17127.49	9.98	3.32	0.031024864	1.51	0.081409374	1.05212194
PE(18:1_18:3)	81096367.79	25655668.33	3.16	1.66	0.002776198	2.56	0.014867272	1.427954998
PE(18:1_22:6)	7681272.82	864091.25	8.89	3.15	0.006243105	2.2	0.025494418	1.381262525
PE(18:1e_22:6)	1051059.83	90782.55	11.58	3.53	0.00015577	3.81	0.00200205	1.585538043
PE(32:1)	1485707.99	128945.27	11.52	3.53	0.000323809	3.49	0.003533553	1.57898367
PE(34:4)	1485707.99	128945.27	11.52	3.53	0.000323809	3.49	0.003533553	1.57898367

Accession	YC_Mean	BC_Mean	FC	log2FC	P.value	-log10(P.value)	FDR	VIP
PI(16:0_16:0)	2060300.92	530998.54	3.88	1.96	0.005004032	2.3	0.021978139	1.359677299
PI(18:0_18:0)	992250.76	220919.76	4.49	2.17	0.01069236	1.97	0.037089123	1.296413601
PS(18:0_22:6)	17553295.68	1124158.27	15.61	3.96	0.001703971	2.77	0.010975898	1.498775926
PS(18:1_20:5)	2716915.87	1137603.29	2.39	1.26	0.014588489	1.84	0.045673192	1.255093807
PS(18:1_22:0)	1646150.29	519782.89	3.17	1.66	0.001979847	2.7	0.012455602	1.34724647
PS(19:0_18:1)	1079420.6	306751.59	3.52	1.82	0.000120831	3.92	0.001695802	1.502025263
PS(39:1)	5394520.98	2252925.6	2.39	1.26	0.004888641	2.31	0.02182142	1.271897197
PS(40:7)	1803904.04	532738.06	3.39	1.76	0.002284735	2.64	0.013547039	1.336349358
SM(d39:0)	28603492.99	10893879.78	2.63	1.39	0.016153947	1.79	0.049186956	1.24079961
TG(10:0_12:0_12:0)	1470848021	477061226.9	3.08	1.62	9.70182E-05	4.01	0.001453502	1.61531721
TG(10:0_12:2_12:3)	305877347.2	101694132.9	3.01	1.59	0.02865225	1.54	0.077058143	1.1641095
TG(10:0_18:1_18:3)	421267700.3	138496287.8	3.04	1.6	0.007238175	2.14	0.027479455	1.351568178
TG(10:0_18:3_18:3)	209541658.3	90061934.46	2.33	1.22	0.000406903	3.39	0.004210407	1.571108144
TG(10:0_20:5_23:1)	48136286.25	20061985.96	2.4	1.26	6.93831E-05	4.16	0.001193194	1.522725402
TG(11:0_18:2_18:2)	28740786.85	9267536.76	3.1	1.63	0.029430314	1.53	0.078459418	1.155958087
TG(11:0_8:0_12:3)	145447284.4	37282271.31	3.9	1.96	0.001249308	2.9	0.008920498	1.496564975
TG(14:0_14:0_20:5)	51927107.53	18284260.9	2.84	1.51	0.006562803	2.18	0.026359153	1.361359948
TG(15:0_10:0_16:0)	3642918631	898604363.3	4.05	2.02	5.23903E-05	4.28	0.001084213	1.616281523
TG(15:0_10:0_20:4)	276027047	136483480.1	2.02	1.02	1.78779E-06	5.75	0.000171184	1.61288887
TG(15:0_14:1_20:5)	33412445.03	14384953.29	2.32	1.22	0.002901888	2.54	0.015259942	1.454534714
TG(15:0_18:2_18:2)	505700484.4	251893859.9	2.01	1.01	0.000955136	3.02	0.007622357	1.397415377
TG(15:0_18:2_18:3)	6544306.58	2202099.02	2.97	1.57	0.009384526	2.03	0.033900905	1.309714003
TG(15:0_18:2_20:5)	45070440.46	21912973.01	2.06	1.04	0.000109634	3.96	0.001574865	1.505861803
TG(15:0_6:0_14:3)	211736502.2	77335851.56	2.74	1.45	8.46794E-05	4.07	0.001325558	1.605777628

Accession	YC_Mean	BC_Mean	FC	log2FC	P.value	-log10(P.value)	FDR	VIP
TG(15:0_6:0_6:0)	38524989.32	5505156.71	7	2.81	0.002234261	2.65	0.013438583	1.441790584
TG(15:0_6:0_8:0)	358541105.6	118969736.5	3.01	1.59	0.004300998	2.37	0.020285441	1.28328883
TG(15:0_8:0_20:3)	163378148.4	51059123.89	3.2	1.68	0.04334632	1.36	0.1034472	1.088184153
TG(16:0_10:0_17:0)	297620784.4	94495426.94	3.15	1.66	0.007184206	2.14	0.027412238	1.359332632
TG(16:0_10:0_22:5)	40807545.16	12777588.96	3.19	1.68	0.00247399	2.61	0.014115618	1.427372521
TG(16:0_10:0_22:6)	26151856.93	2247689.53	11.63	3.54	0.00122804	2.91	0.00882022	1.517795358
TG(16:0_12:0_22:6)	25690453.95	3953259.47	6.5	2.7	0.00010216	3.99	0.001484968	1.508393187
TG(16:0_12:3_14:0)	1339930033	569003146.1	2.35	1.24	0.021055659	1.68	0.060067662	1.191038827
TG(16:0_14:0_14:0)	11555560344	4711679061	2.45	1.29	0.001453815	2.84	0.009916807	1.493151274
TG(16:0_14:0_17:0)	4583859630	1794519046	2.55	1.35	0.005306754	2.28	0.023058886	1.402022269
TG(16:0_14:0_17:1)	129471366.5	54987596.7	2.35	1.24	0.002912012	2.54	0.015259942	1.44349023
TG(16:0_16:0_23:0)	636604795.4	175079821	3.64	1.86	2.51454E-06	5.6	0.000191891	1.607157477
TG(16:0_16:0_24:0)	819461156.8	238592239.9	3.43	1.78	1.8226E-06	5.74	0.000171184	1.612671744
TG(16:0_18:3_23:0)	35471932.18	13376642.87	2.65	1.41	1.17103E-05	4.93	0.000549147	1.575281387
TG(16:0_19:0_20:3)	103459131.3	47434440.92	2.18	1.13	1.55484E-05	4.81	0.000593269	1.568218125
TG(16:0_6:0_22:6)	15384126.34	3925374.53	3.92	1.97	0.017377975	1.76	0.052133924	1.253731684
TG(16:0_8:0_14:0)	18804189157	5635210877	3.34	1.74	6.47622E-05	4.19	0.001159773	1.525186944
TG(16:0_8:0_22:5)	79358578.3	16164333.78	4.91	2.3	0.001325923	2.88	0.009304322	1.477557185
TG(17:0_12:0_14:3)	440219901.7	118347431.5	3.72	1.9	8.16048E-06	5.09	0.000433215	1.58352066
TG(17:0_18:1_22:5)	15326692.98	7283855.92	2.1	1.07	0.00419327	2.38	0.020078364	1.285806322
TG(17:0_6:0_14:3)	65402344.99	5937836.52	11.01	3.46	0.000174357	3.76	0.002194745	1.601622063
TG(17:0_6:0_17:0)	16269382.34	3532838.15	4.61	2.2	0.004582147	2.34	0.020954312	1.412842392
TG(18:0_10:0_18:0)	186575502.4	78023227.11	2.39	1.26	3.71807E-05	4.43	0.000926482	1.543402951
TG(18:0_14:3_16:0)	11854432.76	5861609.65	2.02	1.02	0.015577055	1.81	0.048273056	1.235947854

Accession	YC_Mean	BC_Mean	FC	log2FC	P.value	-log10(P.value)	FDR	VIP
TG(18:0_16:0_19:0)	484214086.9	237550127.1	2.04	1.03	0.000422967	3.37	0.004303688	1.444238613
TG(18:0_16:0_23:0)	264987375.2	78172052.53	3.39	1.76	2.18467E-05	4.66	0.000687321	1.559105845
TG(18:0_16:0_24:0)	281121766	85205082.59	3.3	1.72	5.18553E-05	4.29	0.001084213	1.532864767
TG(18:0_18:0_24:0)	77965558.3	25054530.3	3.11	1.64	0.000119174	3.92	0.001691993	1.502617778
TG(18:0_18:3_23:0)	17553484.97	5183506.43	3.39	1.76	9.72631E-05	4.01	0.001453502	1.510437881
TG(18:0_18:3_24:0)	15406928.19	4709218.95	3.27	1.71	4.2331E-05	4.37	0.001013455	1.539494078
TG(18:0_20:3_23:0)	4123799.22	1375636.11	3	1.58	0.000397094	3.4	0.004144037	1.447728302
TG(18:0_6:0_22:5)	54362017.76	14034240.77	3.87	1.95	0.000310031	3.51	0.003472918	1.459934468
TG(18:1_12:3_20:4)	53140821.58	14472406.24	3.67	1.88	0.00455199	2.34	0.020894659	1.415351556
TG(18:1_18:2_23:1)	29177848.6	12652954.96	2.31	1.21	0.028205861	1.55	0.076193267	1.137277741
TG(18:1_18:3_18:3)	51378070.28	15666132.38	3.28	1.71	0.010845546	1.96	0.037487614	1.296556103
TG(18:2_12:3_18:2)	70049690.7	17581815.85	3.98	1.99	0.001333815	2.87	0.009306217	1.510832199
TG(18:3_11:1_18:2)	29047513.67	7046580.6	4.12	2.04	0.002640984	2.58	0.014531823	1.44516436
TG(18:4_8:0_19:0)	8019462.8	2549227.98	3.15	1.65	0.01198782	1.92	0.04029009	1.271707627
TG(19:0_6:0_6:0)	462193173.3	98548810.94	4.69	2.23	0.002595138	2.59	0.014531823	1.412891069
TG(19:0_9:0_21:0)	52557182.81	13159054.21	3.99	2	5.13629E-06	5.29	0.000313571	1.593604984
TG(19:1_6:0_14:3)	33185793.45	11243913	2.95	1.56	0.03919171	1.41	0.096440105	1.100449053
TG(19:1_6:0_20:5)	29930244.28	4959319.6	6.04	2.59	0.005489476	2.26	0.023538034	1.401192698
TG(19:1_8:0_16:0)	93087967.92	26472188.19	3.52	1.81	0.000133433	3.87	0.001752604	1.590981551
TG(19:1_8:0_16:1)	29344352.25	11231773.97	2.61	1.39	0.006941767	2.16	0.026907612	1.328519743
TG(25:0_16:0_18:0)	77564203.46	23994011.23	3.23	1.69	0.00013248	3.88	0.001752604	1.498306898
TG(25:0_18:0_18:0)	15120360.11	5392491.44	2.8	1.49	0.0002586	3.59	0.003036063	1.468959841
TG(26:0_18:0_18:0)	18191330.19	8003690.29	2.27	1.18	0.004925622	2.31	0.021860435	1.271276593
TG(26:0_6:0_8:0)	25808774015	7260249643	3.55	1.83	0.000478608	3.32	0.004601422	1.572397411

Accession	YC_Mean	BC_Mean	FC	log2FC	P.value	-log10(P.value)	FDR	VIP
TG(29:0_6:0_8:0)	15512865.82	6619880.41	2.34	1.23	0.003343345	2.48	0.016799278	1.412768469
TG(29:2)	4337947.25	1321292.13	3.28	1.72	0.013244147	1.88	0.042749852	1.24211775
TG(30:5)	3038744.31	1325495.37	2.29	1.2	0.010145081	1.99	0.035800994	1.197423904
TG(34:3)	16792846.46	2913718.13	5.76	2.53	0.0029406	2.53	0.015343899	1.435436695
TG(38:3)	23005277.74	8071279.87	2.85	1.51	0.021535615	1.67	0.061151129	1.187006715
TG(42:6e)	57100420.33	25899806.22	2.2	1.14	4.57016E-06	5.34	0.000310009	1.595836648
TG(44:3)	38660361.33	16482662.43	2.35	1.23	0.004038728	2.39	0.019646562	1.424013945
TG(44:6)	66369543.71	9119777.08	7.28	2.86	1.09876E-05	4.96	0.000536632	1.576751994
TG(46:7e)	22323689.21	10902851.04	2.05	1.03	4.58256E-05	4.34	0.001052313	1.53662379
TG(4:0_10:0_16:0)	4095513.63	1189574.96	3.44	1.78	0.001737449	2.76	0.01110694	1.356805231
TG(4:0_10:0_18:1)	291563080	114517243.2	2.55	1.35	0.005707786	2.24	0.023943169	1.3907804
TG(4:0_10:1_18:3)	4712101.61	1153272.39	4.09	2.03	0.003961	2.4	0.019361169	1.387873891
TG(4:0_10:3_20:4)	2610478.15	450593.75	5.79	2.53	0.005269373	2.28	0.022978229	1.383148297
TG(4:0_12:0_18:3)	25998355.41	11291957.02	2.3	1.2	0.047698091	1.32	0.111569673	1.042259318
TG(4:0_12:0_20:3)	1470848021	477061226.9	3.08	1.62	9.70182E-05	4.01	0.001453502	1.61531721
TG(4:0_12:3_16:0)	68163431.28	8243659	8.27	3.05	0.001358192	2.87	0.009422458	1.491247241
TG(4:0_13:0_20:3)	85781323.35	16926488.55	5.07	2.34	0.014714332	1.83	0.045949359	1.267402078
TG(4:0_14:0_19:1)	123663132.6	58775451.88	2.1	1.07	0.002318735	2.63	0.013547039	1.426069546
TG(4:0_14:0_22:6)	11195925.71	1772768.29	6.32	2.66	0.002633654	2.58	0.014531823	1.416377747
TG(4:0_14:3_16:0)	738975590.1	249169026.6	2.97	1.57	0.000973163	3.01	0.007715794	1.395841873
TG(4:0_16:0_22:6)	98002384.11	18127558.66	5.41	2.43	0.004896863	2.31	0.02182142	1.373634636
TG(4:0_16:0_23:0)	3003753351	1060075366	2.83	1.5	0.000526509	3.28	0.004838255	1.432499363
TG(4:0_17:0_22:5)	29056048.84	10751181.78	2.7	1.43	0.002375887	2.62	0.013748614	1.33314644
TG(4:0_18:1_22:6)	12304138.16	5912587.54	2.08	1.06	0.030655405	1.51	0.080683781	1.054090012

Accession	YC_Mean	BC_Mean	FC	log2FC	P.value	-log10(P.value)	FDR	VIP
TG(4:0_18:2_18:2)	204607297.3	63640112.51	3.22	1.68	3.00343E-05	4.52	0.000833451	1.63327486
TG(4:0_18:2_20:5)	204607297.3	60614881.76	3.38	1.76	2.85528E-08	7.54	3.4863E-05	1.658684353
TG(4:0_18:3_18:3)	769637916.3	243117289.9	3.17	1.66	4.88949E-05	4.31	0.001066083	1.643541861
TG(4:0_20:3_20:5)	8670111.44	4204736.1	2.06	1.04	0.006592409	2.18	0.026391251	1.242601896
TG(4:0_6:0_18:3)	20675955.88	3521100.04	5.87	2.55	0.000353285	3.45	0.003783863	1.540319048
TG(4:0_6:0_22:2)	352126619.9	85967378.26	4.1	2.03	0.006210722	2.21	0.025447288	1.372070193
TG(4:0_8:0_18:2)	124461726.7	15653628.31	7.95	2.99	0.006428716	2.19	0.025905815	1.32850554
TG(4:0_9:0_22:0)	9017252.02	3020029.7	2.99	1.58	0.023844116	1.62	0.066774462	1.190001737
TG(56:6e)	8568749.79	3034080	2.82	1.5	0.00045186	3.34	0.004413766	1.440917936
TG(6:0_10:1_18:3)	6721762.35	818281.3	8.21	3.04	0.000860556	3.07	0.007147881	1.49326713
TG(6:0_12:0_12:3)	1064525377	268150661.1	3.97	1.99	0.000543774	3.26	0.004881968	1.549638363
TG(6:0_13:0_14:3)	130316447.8	36613768.17	3.56	1.83	0.000924205	3.03	0.007502102	1.511256069
TG(6:0_14:0_22:5)	94221821.53	10405910.77	9.05	3.18	0.001832842	2.74	0.011655728	1.464669045
TG(6:0_18:1_18:3)	844666460.9	296710809.9	2.85	1.51	0.002770648	2.56	0.014867272	1.407527295
TG(6:0_6:0_13:0)	20651912.49	8142451.94	2.54	1.34	0.024611648	1.61	0.068609184	1.177568667
TG(6:0_6:0_14:3)	3688120.51	743276.73	4.96	2.31	0.002189099	2.66	0.01323213	1.443896977
TG(6:0_6:0_17:1)	4290523.13	1781708.15	2.41	1.27	0.029160097	1.54	0.078054149	1.131473001
TG(6:0_6:0_18:2)	311994997.4	117656545.1	2.65	1.41	0.020455371	1.69	0.058767077	1.111514902
TG(6:0_6:0_18:3)	90934947.17	11167574.46	8.14	3.03	0.002766388	2.56	0.014867272	1.43658831
TG(6:0_6:0_20:2)	418201723.5	161129336.6	2.6	1.38	0.008700389	2.06	0.031849286	1.214212945
TG(6:0_6:0_20:5)	116216896.3	44632887.96	2.6	1.38	0.019754093	1.7	0.057427971	1.225578215
TG(6:0_6:0_22:6)	109105280.4	10368424.34	10.52	3.4	0.001224902	2.91	0.00882022	1.499630724
TG(6:0_8:0_14:0)	1360068997	241712878.4	5.63	2.49	2.19537E-05	4.66	0.000687321	1.55874829
TG(6:0_8:0_18:3)	42331891.09	15297462.74	2.77	1.47	0.008209775	2.09	0.030561388	1.220373804

Accession	YC_Mean	BC_Mean	FC	log2FC	P.value	-log10(P.value)	FDR	VIP
TG(6:0_8:0_20:4)	6985199.2	1557367.05	4.49	2.17	0.001138584	2.94	0.008535702	1.385889776
TG(6:0_8:0_20:5)	9790253.62	1749060.99	5.6	2.48	1.49866E-06	5.82	0.000171184	1.615715739
TG(6:0_8:0_22:5)	7394133.78	1083856.14	6.82	2.77	0.01231122	1.91	0.041071038	1.263363509
TG(6:0_8:0_22:6)	71567950.33	4213865.42	16.98	4.09	0.001036155	2.98	0.008162225	1.522288515
TG(6:0_8:0_8:0)	14641208.51	1749560.27	8.37	3.06	0.001149965	2.94	0.00856163	1.486742897
TG(8:0_10:0_10:0)	156596750.4	63193902.69	2.48	1.31	0.01020532	1.99	0.035909786	1.318686843
TG(8:0_12:0_12:3)	818604285.1	227977627.3	3.59	1.84	0.000440217	3.36	0.004369961	1.557301381
TG(8:0_14:0_17:1)	55973309.87	18693516.83	2.99	1.58	0.004302972	2.37	0.020285441	1.417751118
TG(8:0_17:1_18:3)	19249913.54	5507364.27	3.5	1.81	0.000533521	3.27	0.004838255	1.431687303
TG(8:0_18:1_18:3)	346465845.5	71385301.09	4.85	2.28	8.3196E-05	4.08	0.00131925	1.595199886
TG(8:0_18:1_22:5)	42130286.51	12016157.63	3.51	1.81	0.000913169	3.04	0.007483087	1.400008433
TG(8:0_18:2_18:3)	51371218.2	9480597.14	5.42	2.44	6.09965E-05	4.21	0.001141594	1.527167091
TG(8:0_18:3_22:5)	49191885.89	14394445.43	3.42	1.77	5.94422E-05	4.23	0.001141594	1.52819664
TG(8:0_8:0_10:0)	621710186.7	274644439.8	2.26	1.18	0.033409569	1.48	0.085341178	1.114723189
TG(8:0_8:0_18:3)	129152892.4	25456591.16	5.07	2.34	0.005895113	2.23	0.024482767	1.359458554
TG(8:0_8:0_8:0)	131195430.1	64059058.57	2.05	1.03	0.043255869	1.36	0.1034472	1.054726212
TG(8:0_8:0_9:0)	18467762.07	5107150.73	3.62	1.85	0.00705049	2.15	0.027034963	1.235755951
TG(9:0_18:1_18:2)	136406838	51766375.73	2.64	1.4	0.032869768	1.48	0.084670858	1.132341715
TG(9:0_18:2_18:2)	22372018.5	5719151.28	3.91	1.97	0.00032702	3.49	0.003533553	1.457595143
TG(9:0_9:0_23:0)	44212670.1	10737695.81	4.12	2.04	0.002328152	2.63	0.013547039	1.441257375
TG(9:0_9:0_9:0)	212532845.6	79312375.12	2.68	1.42	0.010868645	1.96	0.037487614	1.189561581
ZyE(32:6)	18198238.88	5297800.85	3.44	1.78	0.012549809	1.9	0.041639449	1.173401552
ZyE(37:6)	7424497.63	2961373.73	2.51	1.33	0.018243612	1.74	0.053935715	1.240150753
BisMePA(38:8e)	934832.2	3313462.26	0.28	-1.83	0.013067291	1.88	0.042547098	1.168450712

Accession	YC_Mean	BC_Mean	FC	log2FC	P.value	-log10(P.value)	FDR	VIP
CL(70:1)	3718937.06	9131385.28	0.41	-1.3	0.011044947	1.96	0.037775575	1.187453055
CL(72:4)	2253103.07	8250938.92	0.27	-1.87	0.004991314	2.3	0.021978139	1.26950187
CL(74:2)	5686276.66	21682687.86	0.26	-1.93	0.012212783	1.91	0.040854267	1.176169862
CL(74:5)	2171757.39	19293399.56	0.11	-3.15	5.18958E-05	4.28	0.001084213	1.532684259
CL(78:11)	728732.39	6389693.27	0.11	-3.13	0.000126922	3.9	0.001752604	1.499829522
CL(80:5)	2926558.02	23815896.43	0.12	-3.02	0.006836455	2.17	0.02676847	1.239270446
CarE(18:0)	558482.52	71195592.24	0.01	-6.99	0.016707465	1.78	0.050619887	1.245112509
CarE(20:0)	77308.08	5813901.08	0.01	-6.23	0.012425411	1.91	0.041339038	1.271545781
Cer(d16:1_16:0)	967454.62	10700650.34	0.09	-3.47	0.020298142	1.69	0.058452903	1.218291596
Cer(d16:1_22:0)	10206423.87	41923125.04	0.24	-2.04	0.006134777	2.21	0.025305954	1.341628536
Cer(d16:1_24:1)	1265800.26	9162868.77	0.14	-2.86	0.013623142	1.87	0.043227821	1.269063407
Cer(d18:0_16:0)	1173341.35	6693475.87	0.18	-2.51	0.042675937	1.37	0.102775778	1.074397694
Cer(d18:1_16:0)	9257030.37	87962744.56	0.11	-3.25	0.01517352	1.82	0.047262419	1.260233596
Cer(d18:1_18:0)	978799.97	34193771.47	0.03	-5.13	0.000507677	3.29	0.004768258	1.559412656
Cer(d18:1_22:0)	36030538.57	102760201.4	0.35	-1.51	0.020926859	1.68	0.059919841	1.19366519
Cer(d18:1_24:1)	4132148.46	22656776.28	0.18	-2.45	0.003211307	2.49	0.01640588	1.413587803
Cer(d18:1_24:2)	306855.07	3905823.57	0.08	-3.67	0.001096947	2.96	0.008403232	1.388268582
Cer(d19:1_24:0)	1752724.97	9866560.93	0.18	-2.49	0.009884486	2.01	0.03514023	1.300786817
Cer(d19:1_24:1)	389436.48	4886598.96	0.08	-3.65	0.002154394	2.67	0.01308714	1.457974924
Cer(d20:1_24:0)	466931	2275371.39	0.21	-2.28	0.011290342	1.95	0.038507006	1.288803057
DG(18:1_18:1)	35013863.46	335276911.5	0.1	-3.26	2.10842E-06	5.68	0.000171626	1.610306016
DG(20:0e)	732727972.4	1665798034	0.44	-1.18	0.000140943	3.85	0.001830764	1.581958794
DG(32:1)	7746172.16	29654731.35	0.26	-1.94	0.006727338	2.17	0.026669088	1.324281889
DG(34:1)	43471125.31	232361055.6	0.19	-2.42	0.001991199	2.7	0.012455602	1.474182074

Accession	YC_Mean	BC_Mean	FC	log2FC	P.value	-log10(P.value)	FDR	VIP
DG(34:2e)	52887948.41	166436560.1	0.32	-1.65	0.002614442	2.58	0.014531823	1.325312164
DG(36:1)	19987569.43	60518336.98	0.33	-1.6	0.001593359	2.8	0.010516173	1.457724024
DG(36:2)	23856817.39	231072526.8	0.1	-3.28	5.99558E-05	4.22	0.001141594	1.527948035
DG(36:3)	12157423.37	61478137.6	0.2	-2.34	0.001707961	2.77	0.010975898	1.358267668
DG(36:4e)	178296843.4	456410606.8	0.39	-1.36	0.000324387	3.49	0.003533553	1.548059075
DG(38:1)	1867632.55	24466188.8	0.08	-3.71	2.70296E-05	4.57	0.000767514	1.553030406
DG(38:4)	1702362.8	11388318.18	0.15	-2.74	1.90138E-05	4.72	0.000629238	1.562923438
DG(52:1)	22885684.65	112562399.3	0.2	-2.3	0.000952474	3.02	0.007622357	1.513575595
DG(52:2)	18595634.97	124887605.8	0.15	-2.75	1.28911E-06	5.89	0.000171184	1.618118496
DGDG(30:5)	497131.32	1535326.21	0.32	-1.63	0.033859814	1.47	0.086130902	1.113436093
DGDG(32:5)	337149.55	1724781.81	0.2	-2.35	0.000789559	3.1	0.006709431	1.409086
GD3(d42:1)	92834.01	775900.55	0.12	-3.06	0.01326961	1.88	0.042749852	1.166804338
GM3(d40:1)	8511.09	1407001.42	0.01	-7.37	6.20367E-05	4.21	0.001141594	1.526640572
GM3(d42:1)	3619.94	1747440.76	0	-8.92	0.00025005	3.6	0.002964187	1.585166359
Hex1Cer(d15:0_18:1)	343917.79	2472719.74	0.14	-2.85	0.006022595	2.22	0.02492742	1.371040897
Hex1Cer(d18:1_16:0)	6229657.07	68490331.2	0.09	-3.46	0.001631623	2.79	0.010653539	1.488509856
Hex1Cer(d23:1_18:3)	10638184.33	81341554.91	0.13	-2.93	0.015459021	1.81	0.048029172	1.246230676
Hex1Cer(d32:0)	1639333.81	11099757.48	0.15	-2.76	0.002468263	2.61	0.014115618	1.453796008
Hex1Cer(d32:1)	131711.55	2888572.81	0.05	-4.45	0.000130319	3.88	0.001752604	1.588903323
Hex1Cer(d35:1)	94077.23	2198737.35	0.04	-4.55	3.31548E-05	4.48	0.000880045	1.546877799
Hex1Cer(d36:1)	384673.99	39004218.58	0.01	-6.66	7.22644E-05	4.14	0.001208697	1.621576286
Hex1Cer(d36:2)	6130.9	1306788.26	0	-7.74	1.60973E-07	6.79	4.9137E-05	1.644091227
Hex1Cer(d36:3)	2093711.94	7983893.76	0.26	-1.93	0.021223352	1.67	0.060404925	1.106750673
Hex1Cer(d37:1)	14863.16	3345302.25	0	-7.81	1.31885E-06	5.88	0.000171184	1.617503574

Accession	YC_Mean	BC_Mean	FC	log2FC	P.value	-log10(P.value)	FDR	VIP
Hex1Cer(d38:1)	2739929.66	36557112.34	0.07	-3.74	0.001180271	2.93	0.008629407	1.517601015
Hex1Cer(d39:1)	4812820.42	27370614.59	0.18	-2.51	0.007851102	2.11	0.029678625	1.34314692
Hex1Cer(d40:2)	131707.07	3122577.98	0.04	-4.57	5.99258E-05	4.22	0.001141594	1.52754681
Hex1Cer(d42:0+O)	1740844.8	12630937.09	0.14	-2.86	0.006862024	2.16	0.02676847	1.238867703
Hex1Cer(t36:0)	1661796.23	19154673.21	0.09	-3.53	0.015742212	1.8	0.048441103	1.258224478
Hex2Cer(d37:1)	61585.71	4461062.64	0.01	-6.18	3.31413E-05	4.48	0.000880045	1.546827683
Hex2Cer(m36:0+O)	1701508.92	10287988.12	0.17	-2.6	0.002545815	2.59	0.014324608	1.327425298
Hex2Cer(t34:0)	231776.14	7499785.01	0.03	-5.02	1.41005E-05	4.85	0.000573889	1.57058438
LBPA(16:0_18:0)	58693.01	645184.97	0.09	-3.46	0.015750301	1.8	0.048441103	1.231086848
LPC(32:1)	632204.61	10063160.56	0.06	-3.99	0.013015184	1.89	0.042547098	1.252913849
LPE(18:0)	41281.4	146078.21	0.28	-1.82	0.002642368	2.58	0.014531823	1.324393023
MGDG(18:1_22:4)	31082626.93	88919469.54	0.35	-1.52	0.023140237	1.64	0.065252262	1.177273136
MGDG(18:1_22:5)	9881846.91	38410170.28	0.26	-1.96	0.031070244	1.51	0.081409374	1.138837153
MGDG(30:0e)	112154709.2	469808596.6	0.24	-2.07	0.043378351	1.36	0.1034472	1.083346775
MGDG(31:0e)	4230346.64	36994472.53	0.11	-3.13	0.009730801	2.01	0.034842547	1.318682469
MGDG(32:0e)	11320491.48	183673112.2	0.06	-4.02	0.001610354	2.79	0.010571196	1.489030023
MGDG(33:0e)	2630872.59	20667054.67	0.13	-2.97	0.010589705	1.98	0.036942942	1.279455681
MGDG(36:2e)	87984.65	2103750.08	0.04	-4.58	0.000427919	3.37	0.004318095	1.443205047
MGDG(38:2e)	1481682.55	11553371.59	0.13	-2.96	0.030230139	1.52	0.08035784	1.151752752
MGDG(38:3)	18834070.23	47517227.25	0.4	-1.34	0.040840263	1.39	0.0989404	1.085712246
MGDG(40:0e)	391857.23	5927055.77	0.07	-3.92	0.002096653	2.68	0.012850752	1.441025881
MGDG(40:1e)	251895.84	4782578.49	0.05	-4.25	0.000273802	3.56	0.003153887	1.466155203
MGDG(42:3)	280506.56	2090236.87	0.13	-2.9	0.000605761	3.22	0.005321107	1.424738136
PC(14:1e_16:0)	1018546.69	9300005.5	0.11	-3.19	0.001574568	2.8	0.010505727	1.457070794

Accession	YC_Mean	BC_Mean	FC	log2FC	P.value	-log10(P.value)	FDR	VIP
PC(15:0_14:0)	23974499.74	89853399.11	0.27	-1.91	0.010964877	1.96	0.037713	1.187973745
PC(16:0e_16:0)	232585.08	5881857.08	0.04	-4.66	0.00993159	2	0.035149194	1.308871653
PC(16:0e_18:2)	118755.96	5451014.22	0.02	-5.52	0.038838718	1.41	0.095802172	1.112148677
PC(16:1e_18:0)	260172.48	4176676.05	0.06	-4	0.000791284	3.1	0.006709431	1.535066166
PC(17:1_16:0)	58687011.66	206192646	0.28	-1.81	0.02921437	1.53	0.078054149	1.124669025
PC(18:0_16:0)	39346491.09	96510571.51	0.41	-1.29	0.043369967	1.36	0.1034472	1.071700592
PC(18:0e_16:0)	196287.77	4153714.64	0.05	-4.4	0.01186884	1.93	0.040143639	1.274978699
PC(18:0e_18:2)	180347.43	6486078.19	0.03	-5.17	0.04284827	1.37	0.102987673	1.096561323
PC(18:1_18:1)	103898658.5	350033883.8	0.3	-1.75	0.013646982	1.86	0.043227821	1.162964373
PC(18:1_18:3)	78199.43	4201044.96	0.02	-5.75	0.000475312	3.32	0.004601422	1.43760052
PC(18:1e_18:2)	79430.54	3803018.72	0.02	-5.58	0.020186777	1.69	0.058407713	1.22099394
PC(20:4e_18:0)	376738.83	3478317.26	0.11	-3.21	0.035253195	1.45	0.088709573	1.03261783
PC(31:0)	203748092.9	952949352.5	0.21	-2.23	0.007246834	2.14	0.027479455	1.332215204
PC(31:0e)	2854901.9	15359210.67	0.19	-2.43	0.002009626	2.7	0.012455602	1.466844156
PC(31:3)	25402968.33	79901797.9	0.32	-1.65	0.017910567	1.75	0.053208764	1.2233409
PC(32:0e)	258342.07	9685071.05	0.03	-5.23	0.01104138	1.96	0.037775575	1.271407624
PC(33:2e)	4191641.53	29703225.12	0.14	-2.83	0.004286073	2.37	0.020285441	1.283574201
PC(33:4e)	9951258.92	122473942.9	0.08	-3.62	4.51144E-05	4.35	0.001052313	1.537467578
PC(34:0)	69784729.9	214490322.7	0.33	-1.62	0.048900224	1.31	0.11372795	1.036320378
PC(34:1e)	1925104.84	32046876.04	0.06	-4.06	0.014571892	1.84	0.045673192	1.251297087
PC(34:4e)	632204.61	10063160.56	0.06	-3.99	0.013015184	1.89	0.042547098	1.252913849
PC(35:3)	5594125.63	31897930.33	0.18	-2.51	0.005499269	2.26	0.023538034	1.360833139
PC(35:4e)	225746.38	1357005.73	0.17	-2.59	0.001463088	2.83	0.009924612	1.368944388
PC(36:3)	69784729.9	214490322.7	0.33	-1.62	0.048900224	1.31	0.11372795	1.036320378

Accession	YC_Mean	BC_Mean	FC	log2FC	P.value	-log10(P.value)	FDR	VIP
PC(37:5e)	493109.34	12397986.65	0.04	-4.65	0.000502573	3.3	0.004756915	1.435268947
PC(8:0e_24:1)	931379.24	10063893.99	0.09	-3.43	0.016787503	1.78	0.050736488	1.13747771
PE(16:0e_18:1)	8649088.22	119170442.7	0.07	-3.78	6.56614E-05	4.18	0.001159773	1.524845468
PE(16:1e_18:1)	16025050.28	64257664.28	0.25	-2	0.019350195	1.71	0.056658484	1.196738765
PE(16:1e_18:2)	7685226.5	64072190.65	0.12	-3.06	0.005559423	2.25	0.023569636	1.360326913
PE(18:0_18:0)	468844.76	1281977.82	0.37	-1.45	0.020088768	1.7	0.058262198	1.113667131
PE(18:0_20:3)	1244798.03	8100861.25	0.15	-2.7	0.004136897	2.38	0.019886424	1.385523747
PE(18:0_20:4)	14106370.58	57066553.9	0.25	-2.02	0.025455382	1.59	0.070319054	1.161203858
PE(18:0e_18:1)	739318.68	3187811.98	0.23	-2.11	0.018384273	1.74	0.054220282	1.201550451
PE(18:0e_18:2)	252087.41	2703364.13	0.09	-3.42	0.023397479	1.63	0.065825627	1.093594685
PE(18:1_18:2)	200533710.5	952877509	0.21	-2.25	0.006630855	2.18	0.026458412	1.345979205
PE(18:1_20:3)	660051.41	3491231.25	0.19	-2.4	0.00056105	3.25	0.005000309	1.428876876
PE(18:1e_16:0)	151489.94	1591872.52	0.1	-3.39	0.002296773	2.64	0.013547039	1.439820156
PE(18:1e_17:1)	1435452.6	4546098.14	0.32	-1.66	0.042453647	1.37	0.102442495	1.061262079
PE(18:1e_18:1)	10701304.94	59144800.17	0.18	-2.47	0.002104955	2.68	0.012850752	1.431144743
PE(18:1e_18:2)	3284635.35	60967604.62	0.05	-4.21	2.5492E-05	4.59	0.000767514	1.554688196
PE(18:1e_20:3)	332677.85	4386275.7	0.08	-3.72	0.000209202	3.68	0.002554355	1.478645605
PE(18:1e_20:4)	3426196.99	24706039.82	0.14	-2.85	0.013351302	1.87	0.042787243	1.258349017
PE(18:1e_22:4)	292532.43	4334803.66	0.07	-3.89	0.010297539	1.99	0.036040161	1.306095756
PE(18:2e_18:2)	2033825.1	29470755.1	0.07	-3.86	1.90176E-05	4.72	0.000629238	1.562831308
PE(31:0e)	1269606.17	9871284.22	0.13	-2.96	0.002966866	2.53	0.01534976	1.315141369
PE(32:0)	27150321.55	78220295.73	0.35	-1.53	0.025659184	1.59	0.070404188	1.159927241
PE(33:0e)	4463339.63	34802658.17	0.13	-2.96	0.008668846	2.06	0.031849286	1.330583877
PE(34:0)	200585329.2	954266807.2	0.21	-2.25	0.00689358	2.16	0.026805926	1.341407014

Accession	YC_Mean	BC_Mean	FC	log2FC	P.value	-log10(P.value)	FDR	VIP
PE(34:0e)	20891978.45	291193047.8	0.07	-3.8	0.001108043	2.96	0.008403232	1.48384076
PE(34:2p)	26414224.89	222709544.7	0.12	-3.08	0.004638305	2.33	0.021053424	1.374932027
PE(34:3)	25402968.33	79901797.9	0.32	-1.65	0.017910567	1.75	0.053208764	1.2233409
PE(36:0)	5691446.5	32980159.88	0.17	-2.53	0.004600258	2.34	0.020958638	1.374164594
PE(36:3)	23809018.55	56593222.4	0.42	-1.25	0.034306854	1.46	0.086905952	1.105560812
PE(36:3e)	19463596.18	94855629.44	0.21	-2.28	2.67884E-05	4.57	0.000767514	1.553243093
PE(36:3p)	8956212.84	90737950.83	0.1	-3.34	0.000534785	3.27	0.004838255	1.431516714
PE(38:4)	58687011.66	206192646	0.28	-1.81	0.02921437	1.53	0.078054149	1.124669025
PE(38:5e)	1325774.92	8662004.09	0.15	-2.71	0.003314757	2.48	0.016724454	1.306191031
PE(40:4p)	493109.34	12397986.65	0.04	-4.65	0.000502573	3.3	0.004756915	1.435268947
PE(52:1)	5626477.91	14376925.69	0.39	-1.35	0.022191034	1.65	0.062866016	1.100483354
PE(54:1)	1173398.32	4165862.42	0.28	-1.83	0.011999705	1.92	0.04029009	1.178335877
PS(18:0_18:0)	3695449.33	20762702.49	0.18	-2.49	0.015970906	1.8	0.04875119	1.143524294
PS(18:0_18:2)	26465282.61	128444636.3	0.21	-2.28	0.03739364	1.43	0.092989071	1.023783061
PS(18:0_20:3)	1294897.62	8012463.72	0.16	-2.63	0.030274043	1.52	0.08035784	1.128215646
PS(18:1_18:1)	30755827.8	145668051.1	0.21	-2.24	0.043821415	1.36	0.103785068	1.071389026
PS(18:1_18:2)	17183811.02	267421166.8	0.06	-3.96	0.000295432	3.53	0.003371233	1.569615236
PS(18:1_20:4)	11077359.12	36162240.86	0.31	-1.71	0.03852192	1.41	0.095213085	1.018820782
PS(20:3_20:3)	481161.14	1927575.38	0.25	-2	0.037951795	1.42	0.094185249	1.084006779
PS(22:4_18:2)	1249148.69	5078953.56	0.25	-2.02	0.047406941	1.32	0.111101487	1.064394561
PS(34:0)	17728359.55	308294232.2	0.06	-4.12	0.000384858	3.41	0.004050958	1.558024561
PS(36:1)	6275591.05	52206885.86	0.12	-3.06	0.003946159	2.4	0.019361169	1.39313495
PS(36:5e)	227786.56	3711469.44	0.06	-4.03	0.002505426	2.6	0.014187831	1.447162176
PS(38:4)	5807828.42	52293788	0.11	-3.17	0.00084969	3.07	0.007147881	1.40451388

Accession	YC_Mean	BC_Mean	FC	log2FC	P.value	-log10(P.value)	FDR	VIP
PS(38:4e)	1474598.16	8061575.18	0.18	-2.45	0.000308851	3.51	0.003472918	1.460306496
PS(38:5)	3090034.61	23860190.25	0.13	-2.95	0.001066052	2.97	0.008290758	1.484673264
PS(38:5e)	639192.09	3734766.31	0.17	-2.55	5.54693E-05	4.26	0.001110296	1.530541853
PS(40:7e)	382283.02	2839992.49	0.13	-2.89	0.013267592	1.88	0.042749852	1.266461671
SM(d32:1)	12859462.23	55107582.49	0.23	-2.1	0.031400453	1.5	0.081922976	1.141973361
SM(d34:0)	14055350.43	62895289.9	0.22	-2.16	0.04391236	1.36	0.103785068	1.070497074
SM(d34:4)	12407004.53	64827864.27	0.19	-2.39	0.013138707	1.88	0.042665853	1.253915392
SM(d35:1)	4230346.64	37119744.31	0.11	-3.13	0.009605461	2.02	0.034494906	1.320301412
SM(d36:0)	2645536.91	15368568.93	0.17	-2.54	0.007979529	2.1	0.029886519	1.331130408
SM(d36:1)	10359103.28	177154965.8	0.06	-4.1	0.00127283	2.9	0.008983382	1.50392837
SM(d36:3)	14055350.43	62895289.9	0.22	-2.16	0.04391236	1.36	0.103785068	1.070497074
SM(d37:1)	26592945.65	202861717.3	0.13	-2.93	0.008770523	2.06	0.031966594	1.33252875
SM(d37:4)	7499223.75	120472164	0.06	-4.01	0.003469997	2.46	0.017364208	1.413792774
SM(d38:3)	5641412.93	49946681.59	0.11	-3.15	0.004848863	2.31	0.021766401	1.378864053
SM(d43:1)	9460650.68	88378762.56	0.11	-3.22	0.003038521	2.52	0.015588381	1.400786651
SM(d43:2)	3115314.72	24476446	0.13	-2.97	0.008712254	2.06	0.031849286	1.332334282
StE(24:7)	45598577.81	199106851.3	0.23	-2.13	0.027334039	1.56	0.07416636	1.146213041
TG(12:1e_10:0_18:3)	1040359	12879426.81	0.08	-3.63	1.31643E-07	6.88	4.9137E-05	1.646077655
TG(14:0e_18:0_18:0)	1681654.32	5638845.51	0.3	-1.75	0.001555998	2.81	0.010438869	1.364808616
TG(14:0e_18:1_18:1)	11031852.36	41321769.33	0.27	-1.91	6.43109E-06	5.19	0.000373922	1.588812473
TG(16:0e_16:0_20:4)	1887746.14	11429895.52	0.17	-2.6	4.22735E-05	4.37	0.001013455	1.539495713
TG(16:0e_6:0_20:4)	756688.31	3204597.17	0.24	-2.08	0.002509887	2.6	0.014187831	1.329296582
TG(16:1_8:0_16:1)	548627311.7	1156777033	0.47	-1.08	0.001419162	2.85	0.009734815	1.371018591
TG(16:1e_16:0_16:0)	10073445.5	29104085.58	0.35	-1.53	4.9794E-06	5.3	0.000313571	1.594079738

Accession	YC_Mean	BC_Mean	FC	log2FC	P.value	-log10(P.value)	FDR	VIP
TG(18:0_14:0_20:5)	407994780.4	1050224186	0.39	-1.36	0.007897666	2.1	0.029752199	1.224070198
TG(18:0_18:1_18:1)	7002456228	16123903481	0.43	-1.2	1.43685E-07	6.84	4.9137E-05	1.645048814
TG(18:0_18:1_20:3)	87396116.33	210784952.2	0.41	-1.27	0.001272016	2.9	0.008983382	1.378365492
TG(18:0_18:1_20:4)	261019720.6	576172932	0.45	-1.14	0.022571278	1.65	0.063795209	1.192548566
TG(18:0_18:2_20:5)	145310153.9	373139159.8	0.39	-1.36	0.001139492	2.94	0.008535702	1.385730854
TG(18:0e_6:0_10:1)	45792628.62	233472679.5	0.2	-2.35	0.002300076	2.64	0.013547039	1.455895834
TG(18:1_17:1_18:1)	75351834.78	176729468.1	0.43	-1.23	0.005532691	2.26	0.023538034	1.362045858
TG(18:1_17:1_20:4)	75351834.78	176729468.1	0.43	-1.23	0.005532691	2.26	0.023538034	1.362045858
TG(18:1_18:1_22:4)	7900838.84	23367719.89	0.34	-1.56	4.74015E-05	4.32	0.001052313	1.535777249
TG(18:1_20:4_22:1)	3168391.95	9262484.46	0.34	-1.55	0.000272208	3.57	0.003153887	1.46650016
TG(18:1e_14:0_14:0)	2878473.24	9863810.3	0.29	-1.78	0.005725967	2.24	0.023943169	1.256604701
TG(18:1e_16:0_16:0)	22854183.79	93132130.94	0.25	-2.03	2.0598E-06	5.69	0.000171626	1.610571962
TG(18:1e_18:1_18:1)	1297696.57	31622840.6	0.04	-4.61	4.8471E-07	6.31	9.86385E-05	1.631904946
TG(18:2e_6:0_6:0)	3596726.09	15742301.76	0.23	-2.13	0.038181652	1.42	0.094563482	1.020713423
TG(18:2e_8:0_10:1)	12157423.37	61478137.6	0.2	-2.34	0.001707961	2.77	0.010975898	1.358267668
TG(18:4_8:0_14:0)	59382012.8	172773939	0.34	-1.54	0.025588809	1.59	0.070404188	1.15752763
TG(19:1_18:1_18:1)	367102669	908543104.9	0.4	-1.31	0.001166808	2.93	0.008629407	1.501250106
TG(19:1_18:1_20:4)	17592261.19	51106712.78	0.34	-1.54	6.64898E-05	4.18	0.001159773	1.524237651
TG(20:1e_16:0_16:0)	20164194.67	111580295.2	0.18	-2.47	0.000434492	3.36	0.004348486	1.556309845
TG(20:1e_6:0_6:0)	7746172.16	29654731.35	0.26	-1.94	0.006727338	2.17	0.026669088	1.324281889
TG(20:1e_8:0_8:0)	19987569.43	60518336.98	0.33	-1.6	0.001593359	2.8	0.010516173	1.457724024
TG(20:2e_16:0_16:0)	18649508.64	120005671.2	0.16	-2.69	3.85618E-07	6.41	9.4168E-05	1.634531604
TG(20:2e_6:0_10:0)	738983.35	10456270.43	0.07	-3.82	8.03453E-05	4.1	0.001290811	1.517662454
TG(20:2e_6:0_8:0)	20869396.86	52527131.52	0.4	-1.33	0.004107217	2.39	0.019821786	1.288040361

Accession	YC_Mean	BC_Mean	FC	log2FC	P.value	-log10(P.value)	FDR	VIP
TG(20:3e_16:0_16:0)	2372370.42	23597012.17	0.1	-3.31	4.31777E-06	5.36	0.000310009	1.597119639
TG(22:2_14:1_14:1)	2065084.1	19379562.74	0.11	-3.23	0.000239279	3.62	0.002864313	1.471983308
TG(24:0_13:0_13:0)	6569958.46	31656978.81	0.21	-2.27	7.05414E-06	5.15	0.000391505	1.586912709
TG(26:0_6:0_20:4)	19474633.97	367144395.8	0.05	-4.24	0.005759792	2.24	0.02400241	1.362116112
TG(28:1_6:0_20:3)	671939.6	10454807.3	0.06	-3.96	0.001894673	2.72	0.011986506	1.441770858
TG(29:0_18:1_18:1)	783459.85	2457388.29	0.32	-1.65	0.002320882	2.63	0.013547039	1.334511425
TG(30:0_18:0_18:1)	2140917.53	5697729.49	0.38	-1.41	0.002842092	2.55	0.015152546	1.318587894
TG(30:0_18:1_18:1)	1610541.79	7590550.42	0.21	-2.24	1.77122E-05	4.75	0.000629238	1.564494191
TG(30:1_18:1_18:1)	732036.24	2793795.71	0.26	-1.93	0.003542085	2.45	0.017580837	1.300432718
TG(30:1_19:0_19:0)	645356.77	2582516.02	0.25	-2	0.000534942	3.27	0.004838255	1.4316384
TG(38:0e)	969781.22	6312793.11	0.15	-2.7	7.48627E-05	4.13	0.001235235	1.519846668
TG(38:1e)	1867632.55	24466188.8	0.08	-3.71	2.70296E-05	4.57	0.000767514	1.553030406
TG(38:4e)	3805547.03	12898007.36	0.3	-1.76	9.76144E-05	4.01	0.001453502	1.510006215
TG(39:6)	731813.09	2687327.15	0.27	-1.88	0.017681249	1.75	0.052784366	1.200117645
TG(40:2e)	531280.05	12878953.88	0.04	-4.6	0.015697727	1.8	0.048441103	1.146241848
TG(41:6)	2715284.67	9328042.82	0.29	-1.78	0.000179819	3.75	0.002240396	1.485142865
TG(44:4)	851066.87	3458942.25	0.25	-2.02	0.034475114	1.46	0.087151375	1.036673349
TG(44:6e)	15299165	76979772.17	0.2	-2.33	0.004365924	2.36	0.020346537	1.281666131
TG(48:0e)	7306718.8	18783404.5	0.39	-1.36	0.000898417	3.05	0.00741194	1.401446017
TG(48:6e)	14567403.98	41727683.49	0.35	-1.52	0.015828088	1.8	0.048558029	1.230849566
TG(4:0_12:0_18:2)	8609300.31	43937310.01	0.2	-2.35	0.007919299	2.1	0.029752199	1.320298299
TG(4:0_14:4_18:1)	4196116.75	26027701.93	0.16	-2.63	4.66998E-05	4.33	0.001052313	1.536218237
TG(4:0_18:2_19:0)	71803866.98	189860402.8	0.38	-1.4	0.015922469	1.8	0.048725151	1.241264449
TG(52:1)	2446247.52	11602147.89	0.21	-2.25	0.035922613	1.44	0.090033747	1.123745498

Accession	YC_Mean	BC_Mean	FC	log2FC	P.value	-log10(P.value)	FDR	VIP
TG(52:3)	5666311.98	23919950.53	0.24	-2.08	0.049221684	1.31	0.11425794	1.066766591
TG(54:1e)	4777432.63	18149753.59	0.26	-1.93	0.004941425	2.31	0.021860435	1.399484785
TG(54:2)	1222431914	3074228521	0.4	-1.33	1.48438E-05	4.83	0.000584652	1.569044656
TG(54:2e)	7248784.51	44946938.94	0.16	-2.63	0.000411643	3.39	0.004223667	1.562299565
TG(59:3)	27639510.22	78384321.35	0.35	-1.5	0.006811099	2.17	0.02676847	1.239624965
WE(19:0_17:3)	8911392.96	18809736.44	0.47	-1.08	0.026100648	1.58	0.071135919	1.077929886
ZyE(23:6)	26383557.62	79182784.9	0.33	-1.59	0.004532952	2.34	0.020885789	1.386421305
ZyE(24:7)	533716.6	26966462.99	0.02	-5.66	0.004414743	2.36	0.02049582	1.281245099
dMePE(16:0_18:3)	45101011.11	216700452	0.21	-2.26	0.004782078	2.32	0.021545821	1.381546275
dMePE(18:0_16:0)	831890	2141801.17	0.39	-1.36	0.032868442	1.48	0.084670858	1.105843011
dMePE(18:0_18:2)	1119400.44	3462600.68	0.32	-1.63	0.008027857	2.1	0.029975577	1.22276628
dMePE(18:2_18:2)	14106370.58	57066553.9	0.25	-2.02	0.025455382	1.59	0.070319054	1.161203858
dMePE(36:3)	820042.58	2542924.09	0.32	-1.63	0.04487134	1.35	0.105768159	1.062501896
